# Palladium-catalyzed carbon–carbon bond cleavage of primary alcohols: decarbonylative coupling of acetylenic aldehydes with haloarenes†

**DOI:** 10.1039/d5ra00357a

**Published:** 2025-03-11

**Authors:** Zewei Jin, Qiang Li, Maoshuai Zhu, Yanqiong Zhang, Xufei Yan, Xiangge Zhou

**Affiliations:** a College of Chemistry, Sichuan University 29 Wangjiang Road Chengdu 610064 P. R. China zhouxiangge@scu.edu.cn; b West China School of Public Health and West China Fourth Hospital, Sichuan University Chengdu 610041 P. R. China yanxf92@scu.edu.cn

## Abstract

In the current work, a palladium-catalyzed C–C bond cleavage reaction of primary alcohols has been developed. This transformation was characterized by a broad substrate scope, superior functional group tolerance, and high efficiency for selective C–C bond cleavage and was then followed by alkynyl-aryl cross coupling. Mechanism studies indicated that the propargyl alcohols underwent β-H elimination to form aldehydes rather than having undergone β-C elimination. The corresponding aldehyde intermediates then proceeded through a decarbonylation and coupling reaction with haloarenes to yield diarylacetylenes.

## Abstract 2

We report a novel method for the palladium-catalyzed cleavage of C–C bonds in primary alcohols, thereby enabling a new route for the synthesis of diarylacetylenes.

## Graphical abstract

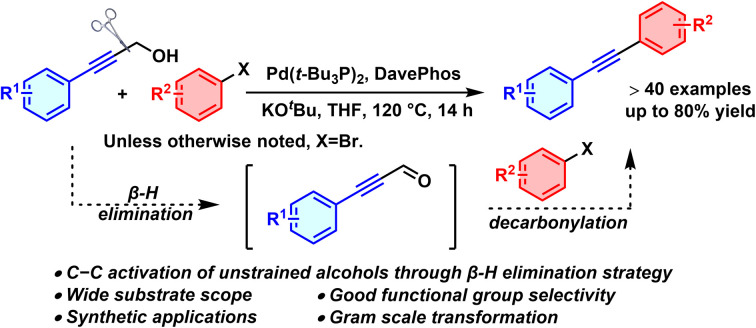

## Introduction

**Scheme 1:**
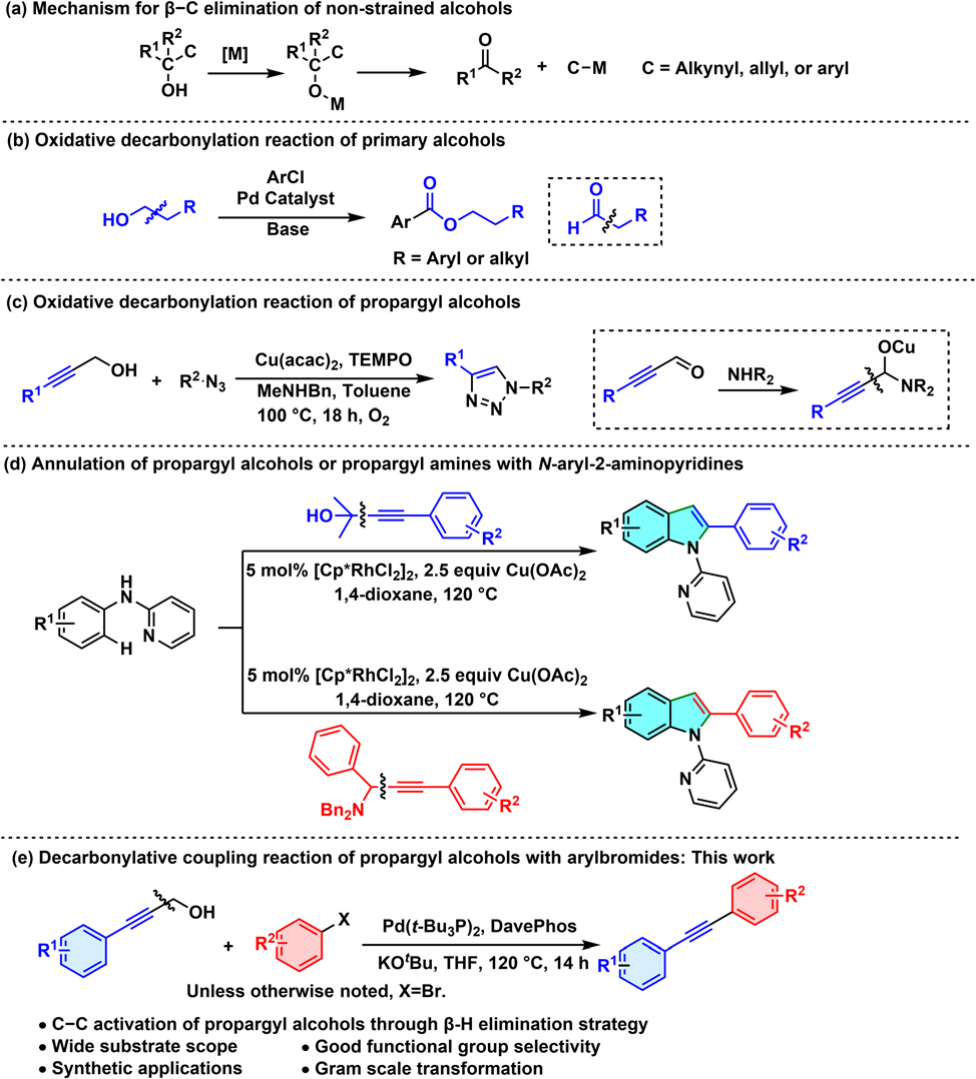
C–C bond cleavage of non-strained alcohols: β-C or β-H elimination strategies.

In recent years, transition-metal-catalyzed C–C bond cleavage reactions have garnered widespread attention. There generally exist two main pathways to achieve the C–C bond cleavage mechanistically: (a) oxidative addition *via* insertion of a low-valence metal into the C–C bonds; (b) β-C elimination driven by the release of small-molecule compounds.^1^ Transition-metal catalysis of β-C elimination of non-strained non-primary alcohols occurs on the metal alkoxide species, thus resulting in the extrusion of carbonyl compounds and formation of C-M species (Scheme 1a).^2^ In contrast, β-H elimination is more favoured than β-C elimination for primary alcohols as a consequence of the more thermodynamically preferred M–H bond forming. Hence, the cleavage of such C–C bonds faces more challenges due to the greater tendency of the more accessible β−H elimination occurring.

Considering that primary alcohols can smoothly undergo β-H elimination under transition-metal catalysis, we envisaged the feasibility of combining β-H elimination with decarbonylation, which would be expected to lead to successful C–C bond cleavage of primary alcohols.^3^ For instance, Jun disclosed a formal dechlorination esterification reaction of aryl chlorides through the cleavage of C–C bonds of primary alcohols under palladium catalysis (Scheme 1b).^4^ The corresponding aldehyde was initially formed *via* β-H elimination on the palladium alkoxide species; then the reaction proceeded through a sequence of decarbonylation and esterification with another alcohol molecule to deliver the ester product. Also, propargyl alcohols have exhibited solid reliability in serving as surrogates in allene and alkyne formation as well as ring expansion reactions.^5^ In this context, Jang reported a copper-catalyzed oxidative decarbonylation reaction of propargyl alcohols for the synthesis of triazole molecules. Including an additional amine was necessary to promote the cleavage of the C(sp^3^)–C(sp) bond *via* nucleophilic addition and the subsequent β-C elimination (Scheme 1c).^6^ Our group has contributed to the field of activation of non-strained C–C bonds.^7^ We have realized such C(sp^3^)–C(sp) bond cleavage in propargyl alcohols and propargyl amines, towards the synthesis of 2-arylindoles, in a rhodium-catalyzed/copper-mediated annulation manner (Scheme 1d).^8^ We have developed a new method for synthesizing diarylalkynes, with our method specifically neither requiring strict control of an inert atmosphere nor needing copper as a co-catalyst—and hence differing from the traditional Sonogashira cross-coupling reaction. In the current work, we attempted to exploit the feasibility of using primary propargyl alcohols as arylacetylene precursors in the coupling with haloarenes, in which a sequence of β-H elimination and decarbonylation would take place,^9^ and it ultimately afforded the corresponding diarylacetylenes (Scheme 1e). This strategy has successfully enabled the efficient synthesis of diarylalkynes, offering a new route for the synthesis of internal alkynes.

## Results and discussion

3-Phenyl-2-propyn-1-ol (1a) and 1-bromo-4-methoxy-benzene (2b) were selected as the model substrates for optimizing conditions (Table 1). We initially screened commercially available metal catalysts, and found that RhCl(PPh_3_)_3_, Cu(OTf)_2_ and Pd(OAc)_2_ were all capable of catalysing the reaction to obtain product 3b, albeit in low yields (entries 1–3). A more electron-rich palladium species, namely Pd(*t*-Bu_3_P)_2_, exhibited a slightly better catalytic efficiency, with the yield increased to 20% with XPhos (2-dicyclohexylphosphino-2′,4′,6′-triisopropylbiphenyl) as the ligand (entry 4). Then, different types of N-ligands, P-ligands and NHC-ligands were investigated, and of them, DavePhos (2-dicyclohexylphosphino-2'-(*N*,*N*-dimethylamino)biphenyl) gave the best results, with a 25% yield for 3b (entries 5–8). The β-H elimination process is more favourable when a bulky monophosphine ligand like DavePhos is coordinated to the palladium catalyst, since an unoccupied coordination site probably exists at the palladium centre.^10^ In addition, inclusion of bases have been found to be necessary to facilitate the cleavage of C–C bonds in some cases.^11^ In our current work, screening different bases revealed KO^*t*^Bu to be the better than Et_3_N, K_2_CO_3_ and K_3_PO_4_, producing 3b in a 30% yield (entries 9–12). Other types of solvents were investigated as well, and using mesitylene instead increased the yield to 43% (entries 13–16). However, it was challenging to purify 3b from residual mesitylene using column chromatography due to the strenuous post-treatment of the high-boiling-point mesitylene and due to the similar polarities of 3b and mesitylene. Therefore, THF was selected as the solvent for further optimizations despite its having given a somewhat lower yield of 40%. In addition, reaction time, temperature, and loading of palladium catalyst were screened systematically in the presence of DavePhos and KO^*t*^Bu as ligand and base (see the ESI† for details). Ultimately, Pd(*t*-Bu_3_P)_2_ (2.5 mol%), DavePhos (10 mol%), and KO^*t*^Bu (2.5 equiv.) in THF (2.0 mL) at 120 °C for 14 h under air were selected as the optimal reaction conditions, delivering 3b in 72% yield (entry 17).

**Table 1:** Optimization of reaction conditions^a^

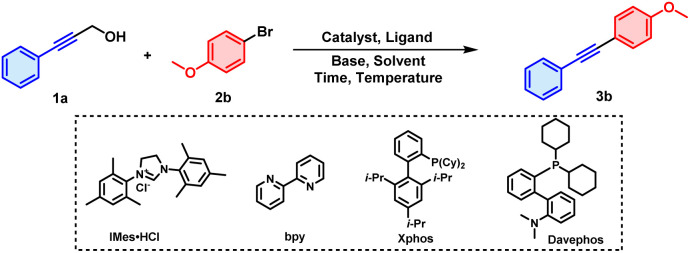					
Entry	Catalyst	Ligand	Base	Solvent	Yields^b^
1	RhCl(PPh_3_)_3_	XPhos	Cs_2_CO_3_	CH_3_CN	<5%
2	Cu(OTf)_2_	XPhos	Cs_2_CO_3_	CH_3_CN	12%
3	Pd(OAc)_2_	XPhos	Cs_2_CO_3_	CH_3_CN	10%
4	Pd(*t*-Bu_3_P)_2_	XPhos	Cs_2_CO_3_	CH_3_CN	20%
5	Pd(*t*-Bu_3_P)_2_	PCy_3_	Cs_2_CO_3_	CH_3_CN	10%
6	Pd(*t*-Bu_3_P)_2_	bpy	Cs_2_CO_3_	CH_3_CN	12%
7	Pd(*t*-Bu_3_P)_2_	IMes·HCl	Cs_2_CO_3_	CH_3_CN	8%
8	Pd(*t*-Bu_3_P)_2_	DavePhos	Cs_2_CO_3_	CH_3_CN	25%
9	Pd(*t*-Bu_3_P)_2_	DavePhos	K_2_CO_3_	CH_3_CN	18%
10	Pd(*t*-Bu_3_P)_2_	DavePhos	K_3_PO_4_	CH_3_CN	10%
11	Pd(*t*-Bu_3_P)_2_	DavePhos	Et_3_N	CH_3_CN	<5%
12	Pd(*t*-Bu_3_P)_2_	DavePhos	KO^*t*^Bu	CH_3_CN	30%
13	Pd(*t*-Bu_3_P)_2_	DavePhos	KO^*t*^Bu	Mesitylene	43%
14	Pd(*t*-Bu_3_P)_2_	DavePhos	KO^*t*^Bu	Toluene	24%
15	Pd(*t*-Bu_3_P)_2_	DavePhos	KO^*t*^Bu	PhCl	35%
16	Pd(*t*-Bu_3_P)_2_	DavePhos	KO^*t*^Bu	THF	40%
17^c^	Pd(*t*-Bu_3_P)_2_	DavePhos	KO^*t*^Bu	THF	72%

a Unless otherwise noted, the reactions were carried out under air atmosphere with 1a (0.3 mmol), 2b (0.2 mmol), catalyst (10 mol%), ligand (20 mol%), and base (2.0 equiv.) in solvent (2.0 mL) at 130 °C for 12 h.

b Isolated yields.

c Pd(*t*-Bu_3_P)_2_ (2.5 mol%), DavePhos (10 mol%), KO^*t*^Bu (2.5 equiv.), 120 °C, 14 h.

**Scheme 2:**
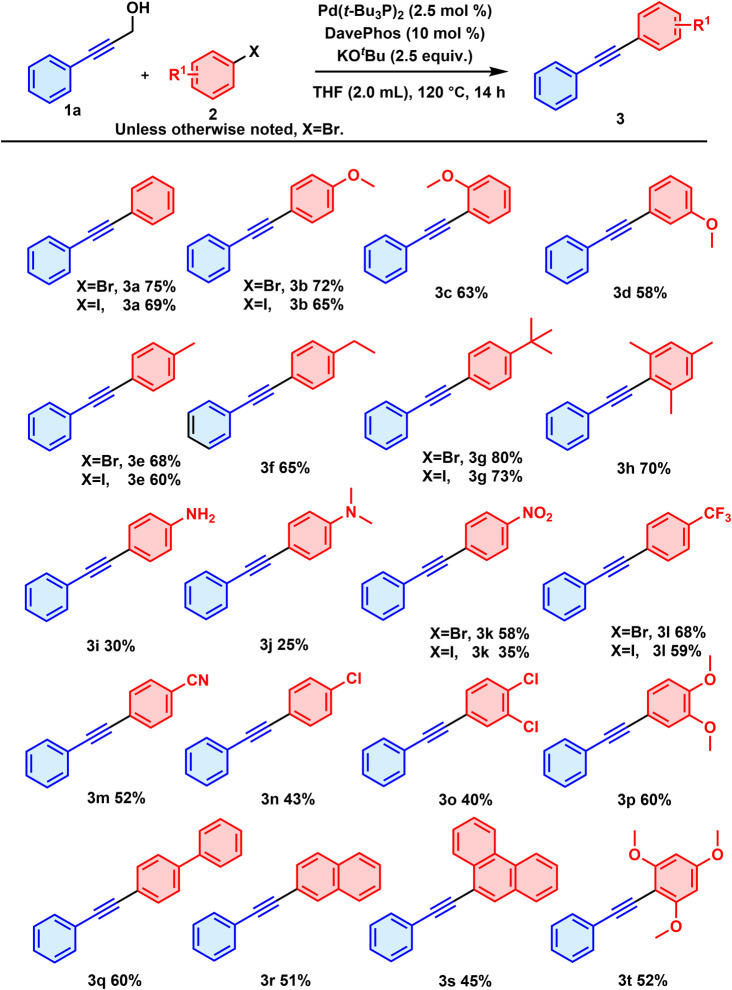
Scope of aryl bromides.^*a a*^1a (0.3 mmol), 2 (0.2 mmol), Pd(*t*-Bu_3_P)_2_ (2.5 mol%), DavePhos (10 mol%) and KO^*t*^Bu (2.5 equiv.) were stirred in THF (2.0 mL) at 120 °C for 14 h under air.

Once the optimal reaction conditions were established, an investigation into the substrate scope for aryl bromides was initiated (Scheme 2). Steric hindrance was found to exert a slight inhibitory effect on the reaction yields—where *para*-OCH_3_-substituted phenyl bromide showed a slightly higher reaction efficiency than did those with the *ortho* and *meta* substituents, and provided a 72% yield for 3b compared to 63% and 58% yields for 3c and 3d, respectively. A more favoured oxidation addition process with palladium catalysis on the less sterically hindered position might account for these variations.^12^ Next, a range of electron-donating substituents were investigated, and the corresponding products were obtained in yields ranging from 25 to 80% (3b, 3e, 3f, 3g, 3h, 3i, 3j). Of them, the strongly electron-donating groups NH_2_ and NH(CH_3_)_2_ apparently caused distinct decreases in the yields, as yields of 30% for 3i and 25% for 3j were observed. These two highly nucleophilic amine substrates are prone to oxidation and overconsumption during the reaction, thus apparently resulting in the severe decrease in the corresponding yield.^13^ Aryl bromides bearing electron-withdrawing substituents, –NO_2_, –CF_3_, –CN, and –Cl for instance, were all viable in the reaction, and moderate yields of the corresponding target products (3k, 3l, 3m, 3n) were observed. In addition, multiply substituted substrates also participated in the reaction successfully, as products 3h, 3o, 3p and 3t were obtained in yields ranging from 40% to 70%. A biphenyl substituent was employed as well, and the product 3q was obtained with a yield of 60%. Polycyclic substrates containing naphthyl and phenanthryl also underwent these transformations to reach the corresponding diarylacetylenes, albeit in moderate yields, specifically of 51% and 45% for 3r and 3s. In addition, we investigated the use of aryl iodides as coupling partners in our substrate studies and found that they afforded moderate to good yields. However, compared to bromobenzene, their performance was slightly inferior.

**Scheme 3:**
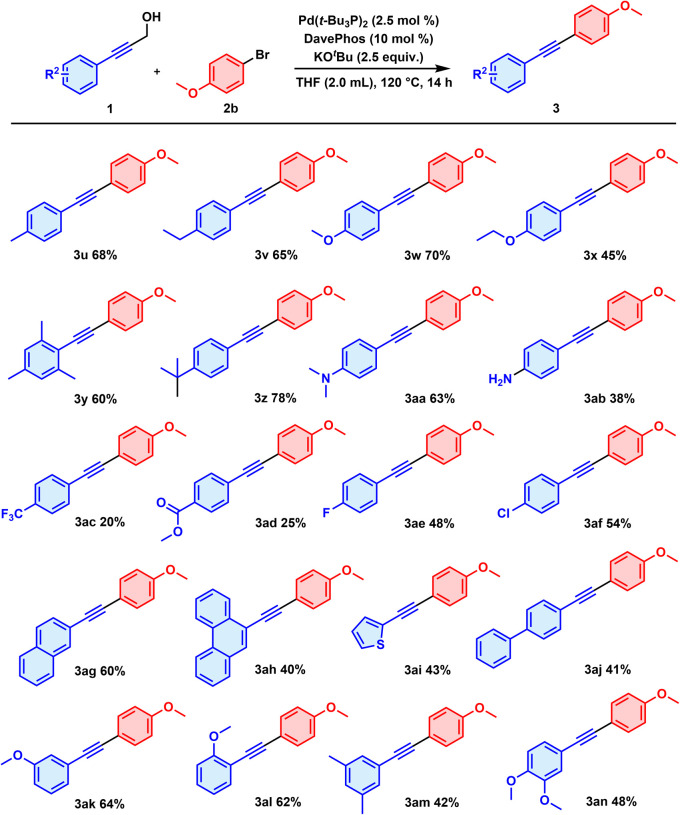
Scope of propargyl alcohols ^*a a*^1 (0.3 mmol), 2b (0.2 mmol), Pd(*t***-**Bu_3_P)_2_ (2.5 mol%), DavePhos (10 mol%) and KO^*t*^Bu (2.5 equiv.) were stirred in THF (2.0 mL) at 120 °C for 14 h under air.

The scope of aryl-substituted propargyl alcohols was subsequently investigated with *para*-methoxy-substituted phenyl bromide as the partner reactant (Scheme 3). Reactions with aryl-substituted propargyl alcohols bearing electron-donating groups, including methyl, ethyl, methoxy, ethoxy, *tert*-butyl, *N*,*N*-dimethyl and amino substituents, produced the target diarylacetylenes in moderate to good yields (3u, 3v, 3w, 3x, 3z, 3aa, 3ab). The amino group, despite being relatively reactive,^14^ was found to be compatible with the reaction, as 3ab was afforded in 38% yield. Substrates containing electron-withdrawing groups, *para*-CF_3_ and –CO_2_Me for instance, delivered the corresponding products 3ac and 3ad—but in relatively low yields, of 20% and 25% yield, respectively, partially due to the competitive homocoupling of arylacetylene detected using gas chromatography-mass spectrometry (GC-MS). As for halogen substituents, fluoro and chloride were tolerated as well, resulting in considerable yields of 3ae and 3af. Consistent with expectations from principles of electronic effects, substrates bearing electron-donating groups performed better than did those bearing electron-withdrawing groups. Steric effects for propargyl alcohols were also examined, and did not notably influence the reaction efficiency, as use of substrates with *ortho*, *meta* and *para*-OCH_3_ substituents led to the products 3w, 3ak and 3al in 70%, 64% and 62% yields, respectively. For polycyclic, heterocyclic and biphenyl substrates, the yields for the corresponding diarylacetylenes 3ag, 3ah, 3ai and 3aj were acceptable, ranging from 40% to 60%. Finally, we demonstrated the capability of multi-substituted substrates to undergo the reactions and the target 3y, 3am and 3an products were obtained in moderate yields.

**Scheme 4:**
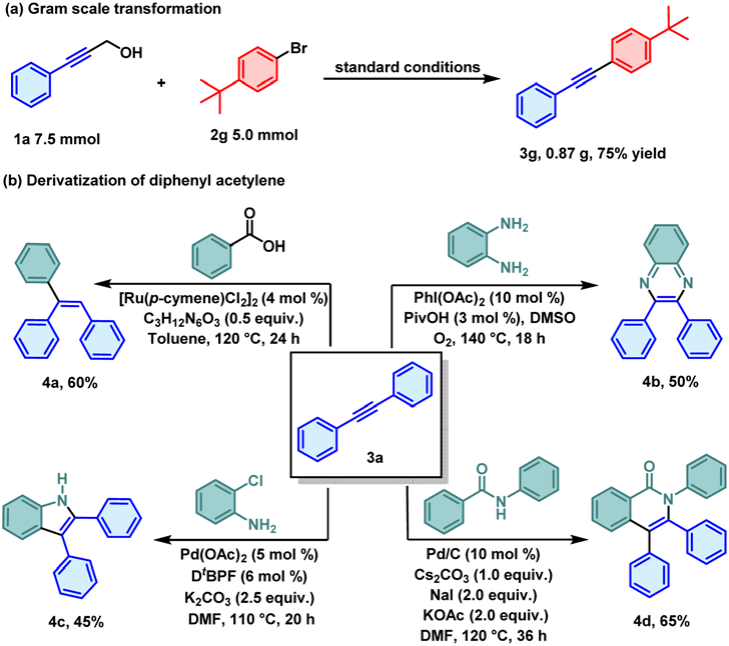
Synthetic applications.

To validate the practicality of the reaction, a series of application studies were conducted. First, this transformation could be successfully scaled up to a gram level, and a mass of 0.87 g of the anticipated product 3g was obtained in 75% yield (Scheme 4a). The derivatizations of diarylacetylene were also implemented (Scheme 4b). Diarylacetylene compounds are widely employed in organic synthesis,^15^ medicinal chemistry,^16^ and materials science,^17^ largely due to their distinctive skeletal rigidity and rich π-electron properties.^18^ The potential of the developed reaction for the synthesis of pharmaceutical molecules was initially demonstrated by the access in 50% yield to 2,3-diphenylquinoxaline 4b,^19^ a precursor to the antituberculosis drug pyrazinamide. Benzamide and *ortho*-chloroaniline underwent cyclization and aromatization reactions with diarylacetylene, resulting in the synthesis of quinolone 4d,^20^ in 65% yield and indole compound 4c,^21^ in 45% yield. In addition, ruthenium-catalyzed decarboxylative hydroarylation of diarylacetylene with benzoic acid was conducted, and led to a 60% yield of tri-aryl-substituted alkene 4a,^22^ which serves as a crucial synthetic intermediate in the fields of fine chemicals and materials.

**Scheme 5:**
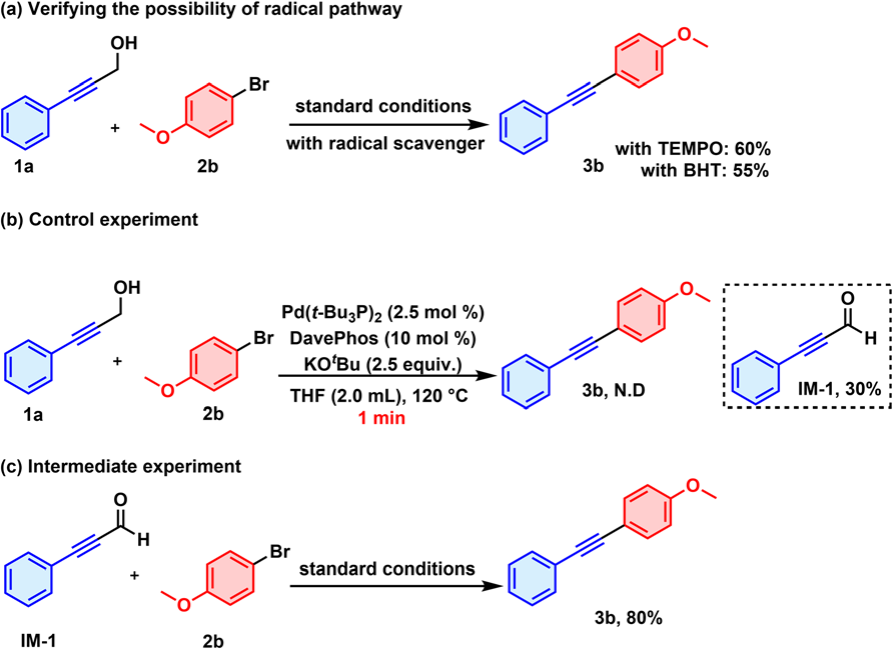
Mechanistic studies.

A series of control experiments were carried out to shed light on the reaction mechanism. Initially, a radical scavenging experiment was conducted in the presence of TEMPO or BHT. The reaction did not give a severely decreased yield of 3b, probably ruling out a radical process (Scheme 5a). Notably, using GC-MS, we could detect 3-phenyl-2-propynal IM-1 with a yield of 30% within the first minute of the reaction (Scheme 5b). The formation of 3-phenyl-2-propynal was consistent with our hypothesis that the reaction involved a β-H elimination process. 3-Phenyl-2-propyn-1-ol was then replaced by 3-phenyl-2-propynal, and an 80% yield of 3b was observed under the standard reaction conditions, thus ultimately validating its role as an intermediate in the reaction (Scheme 5c).

**Scheme 6:**
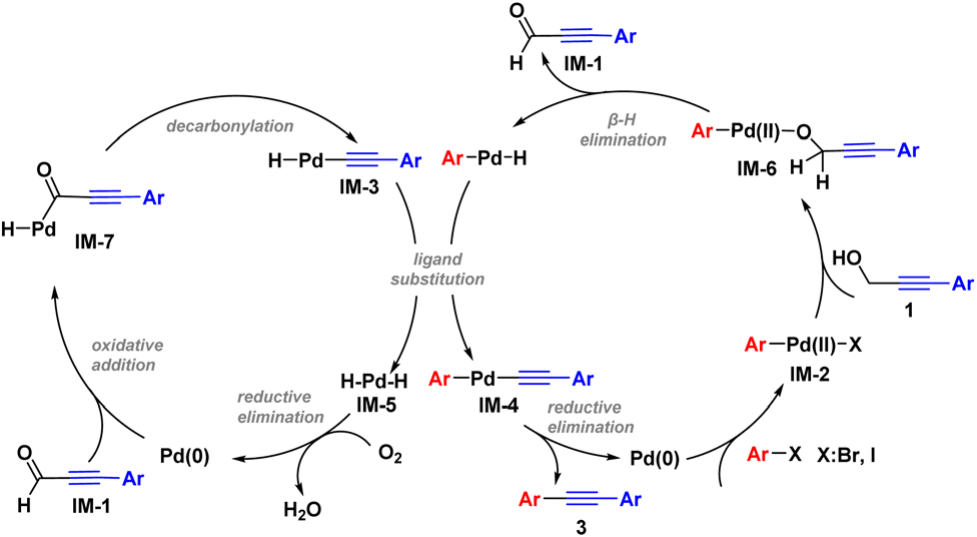
Proposed catalytic cycle.

Based on the mechanism experiments and relevant literature,^3,7^ a catalytic cycle was proposed (Scheme 6). According to this proposal, the reaction was initiated by oxidative addition of Pd(0) with haloarenes to generate intermediate IM-2, followed by ligand exchange of IM-2 with substrate 1 to form IM-6. A β-H elimination of IM-6 occurred, and was accompanied by the generation of IM-1 and the Pd–H species.^23^ Initiated by Pd(0), IM-1 underwent oxidative addition to generate IM-7.^24^IM-3 was obtained as a result of decarbonylation of IM-7, and later underwent ligand exchange with the Pd–H species to yield IM-4 and IM-5, respectively.^25^ Finally, reductive elimination of IM-4 yielded the target diaryl acetylene 3 along with regeneration of the Pd(0) species. Another reductive elimination, of IM-5, also delivered Pd(0), and thus the overall catalytic cycle was realized.

## Conclusions

In summary, a novel method for the palladium-catalyzed C–C bond cleavage of primary propargyl alcohols has been developed. This method, operating through a β-H elimination mechanism, was shown to achieve the decarbonylative coupling of alkynols with haloarenes, offering a new route for the synthesis of diarylacetylenes. The reaction was found to be characterized by a broad substrate scope, good functional group tolerance, and high efficiency for C–C bond cleavage and re-coupling. Furthermore, the practicality of this reaction was further validated by the synthesis of heterocycles derived from diarylacetylenes, including quinoxalines, isoquinolines and indoles. We plan to utilize this strategy in combination with photocatalysis to achieve the cleavage of C–C bonds of primary alcohols in our further studies.

## Data availability

The data underlying this study are available in the published article and its ESI.†

## Conflicts of interest

There are no conflicts to declare.

## Supplementary Material

RA-015-D5RA00357A-s001
