# Supplementary material for: Palladium-catalyzed carbon–carbon bond cleavage of primary alcohols: decarbonylative coupling of acetylenic aldehydes with haloarenes
Source: RSC Adv. 2025 Mar 11;15(10):7826–31. doi: 10.1039/d5ra00357a (PMC11895861; doi:10.1039/d5ra00357a)
Supplement: RA-015-D5RA00357A-s001 [file RA-015-D5RA00357A-s001.pdf]

Supplementary Information (SI) for RSC Advances.
This journal is © The Royal Society of Chemistry 2025

Supporting Information

Palladium-catalyzed carbon-carbon bond cleavage of primary alcohols: decarbonylative coupling of acetylenic aldehydes with haloarenes

Zewei Jin,[a] Qiang Li,[a] Maoshuai Zhu,[a] Yanqiong Zhang,[a] Xufei Yan*[b] and Xiangge Zhou*[a]

[*a] *College of Chemistry, Sichuan University, 29 Wangjiang Road, Chengdu 610064, P. R. China. E-mail: zhouxiangge@scu.edu.cn*

[*b] *West China School of Public Health and West China Fourth Hospital, Sichuan University, Chengdu 610041, P. R. China. E-mail: yanxf92@scu.edu.cn*

Contents

1. General experimental information 3
2. Optimization of reaction conditons 3
   2.1 Screening of catalyst 3
   2.2 Screening of ligand 3
   2.3 Screening of base 4
   2.4 Screening of solvent 5
   2.5 Screening of the loading of $Pd(t\text{-}Bu_3P)_2$, Davephos and KO^tBu 5
   2.6 Screening of the temperature 6
   2.7 Screening of the time 6
3. Synthesis of starting materials 7
   3.1 Synthesis of 3-Phenyl-2-propyn-1-ol 7
4. General procedures of reactions 13
5. Characterization date of the products 13
6. Synthetic applications 26
   6.1 Gram-scale reaction 26
   6.2 Derivatization of diphenylacetylene 3a 26
7. Mechanism experiments 29
   7.1 Verifying the possibility of radical pathway 29
   7.2 Control experiment 29
   7.3 Reaction monitoring experiment 30
   7.4 Intermediate experiment 30
8. References 31
9. Copies of ^{1}H、^{13}C and ^{19}F spectra 33

1. General experimental information

Unless otherwise noted, all reagents were purchased from commercial suppliers and used without further purification. NMR spectra were obtained on an Agilent 400-MR DD2 or a Bruker AV II-400 spectrometer. The ^{1}H NMR (400 MHz) chemical shifts were measured relative to $CDCl_3$ (δ = 7.26 ppm). The ^{13}C NMR (101 MHz) chemical shifts were given using $CDCl_3$ as the internal standard (δ = 77.16 ppm). High resolution mass spectra (HR-MS) were obtained with a Shimadzu LCMS-IT-TOF (ESI). An oil bath was used for the reactions that required high temperature conditions.

2. Optimization of reaction conditons

2.1 Screening of catalyst

Eight types of catalyst were surveyed as shown in Table S1. The results showed that Pd(*t*-$Bu_3P)_2$ gave the best yield, so Pd(*t*-$Bu_3P)_2$ was chosen as optimal catalyst for further evaluation.

Table S1. Screening of catalyst [a]

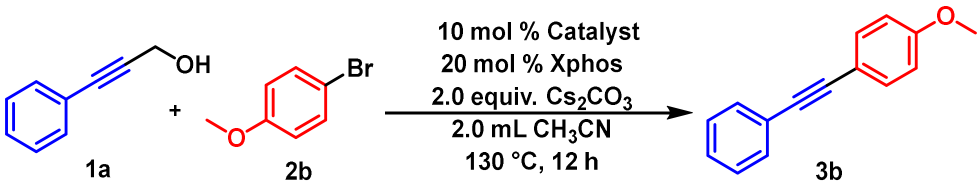

Entry	Catalyst	Yield [b] (%)
1	$RhCl(PPh_3)_3$	<5
2	$Cu(OTf)_2$	12
3	$Ni(acac)_2$	<5
4	$Pd(dppf)Cl_2$	<5
5	$Pd(PPh_3)_4$	8
6	$Pd(OAc)_2$	10
7	$PdCl_2$	10
**8**	**Pd(*t*-$Bu_3P)_2$**	**20**

[a] Unless otherwise noted, the reactions were carried out under air atmosphere with **1a** (0.3 mmol), **2b** (0.2 mmol), catalyst (10 mol %), Xphos (20 mol %), and Cs_2CO_3 (2.0 equiv.) in CH_3CN (2.0 mL) at 130 °C for 12 h. [b] Isolated yields.

2.2 Screening of ligand

Seven types of ligand were surveyed as shown in Table S2. The results showed that Davephos gave the best yield, so we chose it as the optimal ligand for further evaluation.

Table S2. Screening of ligand [a]

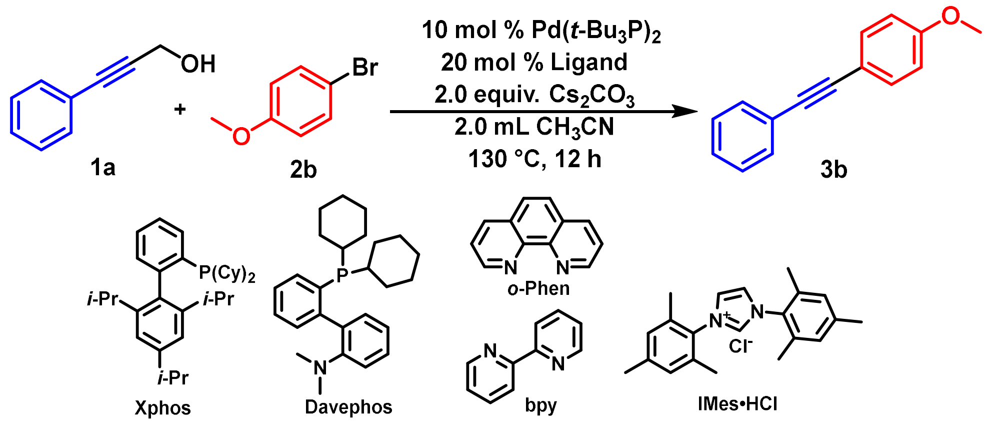

Entry	Ligand	Yield [b] (%)
1	PPh_3	8
2	PCy_3	10
3	XPhos	20
**4**	**DavePhos**	**25**
5	*o*-Phen	<5
6	Bpy	12
7	IMes·HCl	8

[a] Unless otherwise noted, the reactions were carried out under air atmosphere with **1a** (0.3 mmol), **2b** (0.2 mmol), Pd(*t*-$Bu_3P)_2$ (10 mol %), Ligand (20 mol %), and Cs_2CO_3 (2.0 equiv.) in CH_3CN (2.0 mL) at 130 °C for 12 h. [b] Isolated yields.

2.3 Screening of base

Six types of base were surveyed as shown in Table S3. The results showed that KO^{t}Bu gave the best yield of 30%. So KO^{t}Bu was chosen as the optimal base for further evaluation.

Table S3. Screening of base [a]

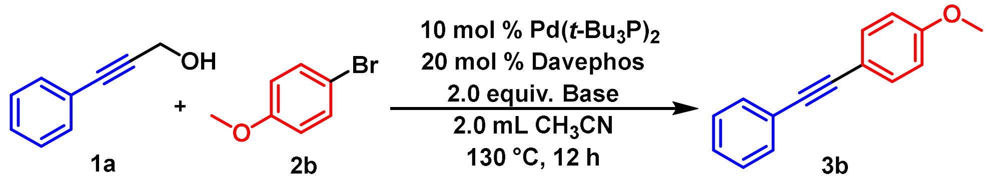

Entry	Base	Yield [b] (%)
1	Cs_2CO_3	25
**2**	**KO^{t}Bu**	**30**
3	Et_3N	<5
4	K_2CO_3	18
5	K_3PO_4	10
6	NaHMDS	18

[a] Unless otherwise noted, the reactions were carried out under air atmosphere with **1a** (0.3 mmol), **2b** (0.2 mmol), Pd(*t*-$Bu_3P)_2$ (10 mol %), Davephos (20 mol %), and Base (2.0 equiv.) in CH_3CN (2.0 mL) at 130 °C for 12 h. [b] Isolated yields.

2.4 Screening of solvent

Seven types of solvent were surveyed as shown in Table S4. The results showed that Mesitylene gave the best yield of 43%. Considering its chemical properties, such as its high boiling point and the difficulty of removing the solvent at high temperatures using rotary evaporation, so we chose THF as the optimal solvent for further evaluation, which gave a 40% yield.

Table S4. Screening of solvent [a]

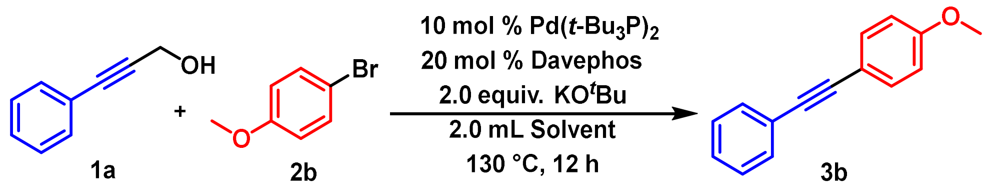

Entry	Solvent	Yield [b] (%)
1	CH_3CN	30
2	Toluene	24
**3**	**THF**	**40**
4	DMSO	<5
5	Mesitylene	43
6	DCE	<5
7	PhCl	35

[a] Unless otherwise noted, the reactions were carried out under air atmosphere with **1a** (0.3 mmol), **2b** (0.2 mmol), Pd(*t*-$Bu_3P)_2$ (10 mol %), Davephos (20 mol %), and KO*t*Bu (2.0 equiv.) in solvent (2.0 mL) at 130 °C for 12 h. [b] Isolated yields.

2.5 Screening of the loading of Pd(*t*-$Bu_3P)_2$, Davephos and KO*t*Bu

It was found that a yield of 61% could be achieved under the conditions of entry 7.

Table S5. Screening of equivalent [a]

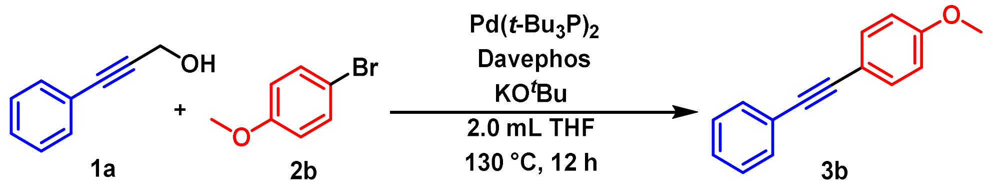

Entry	loading of Pd(*t*-$Bu_3P)_2$	loading of Davephos	loading of base	Yield [b] (%)
1	2.5 mol %	5 mol %	2 equiv.	45
2	2.5 mol %	10 mol %	2 equiv.	55
3	2.5 mol %	20 mol %	2 equiv.	50
4	5 mol %	10 mol %	2 equiv.	30
5	2.5 mol %	10 mol %	1 equiv.	29
6	2.5 mol %	10 mol %	1.5 equiv.	38
**7**	**2.5 mol %**	**10 mol %**	**2.5 equiv.**	**61**

[a] Unless otherwise noted, the reactions were carried out under air atmosphere with **1a** (0.3 mmol), **2b** (0.2 mmol), Pd(*t*-$Bu_3P)_2$ (x mol %), Davephos (y mol %), and KO*t*Bu (z equiv.) in THF (2.0 mL) at 130 °C for 12 h. [b] Isolated yields.

2.6 Screening of the temperature

Screening of the temperature showed that 120 °C gave the best yield of 66% (entry 4).

Table S6. Screening of temperature [a]

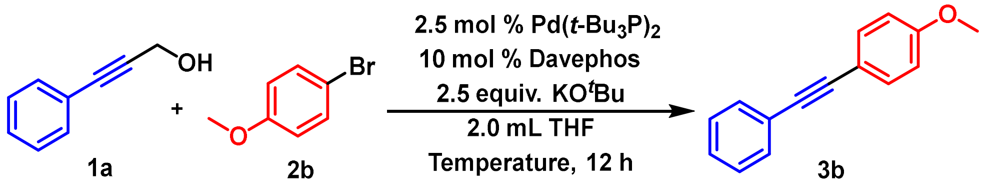

Entry	Temperature (°C)	Yield [b] (%)
1	90	55
2	100	58
3	110	63
**4**	**120**	**66**
5	130	61
6	140	45

[a] Unless otherwise noted, the reactions were carried out under air atmosphere with **1a** (0.3 mmol), **2b** (0.2 mmol), Pd(t-$Bu_3P)_2$ (2.5 mol %), Davephos (10 mol %), and KO^{t}Bu (2.0 equiv.) in THF (2.0 mL) for 12 h. [b] Isolated yields.

2.7 Screening of the time

Screening of the reaction time showed that 14 h gave the best yield of **3b** in 72%, so be as the optimization in this reaction (entry 4).

Table S7. Screening of reaction time [a]

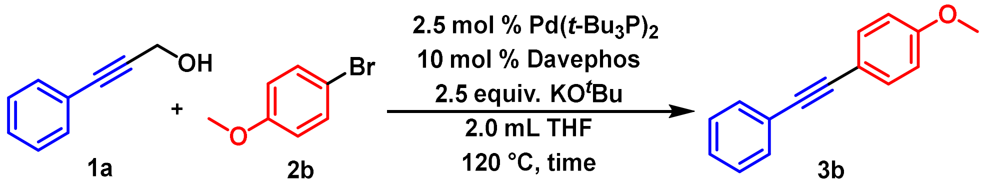

Entry	Time (h)	Yield [b] (%)
1	8	45
2	10	53
3	12	66
**4**	**14**	**72**
5	16	65

[a] Unless otherwise noted, the reactions were carried out under air atmosphere with **1a** (0.3 mmol), **2b** (0.2 mmol), Pd(t-$Bu_3P)_2$ (2.5 mol %), Davephos (10 mol %), and KO^{t}Bu (2.0 equiv.) in THF (2.0 mL) at 120 °C for the indicated time. [b] Isolated yields.

3. Synthesis of starting materials

3.1 Synthesis of 3-Phenyl-2-propyn-1-ol

Procedure A:

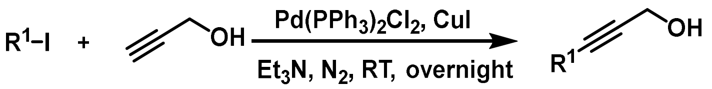

In a 25.0 mL oven-dried round-bottom double-necked flask equipped with a magnetic stir bar, iodobenzene (1.02 g, 5 mmol), prop-2-yn-1-ol (0.34 g, 6 mmol), CuI (28.5 mg, 0.15 mmol), and $Pd(PPh_3)_2Cl_2$ (56.2 mg, 0.08 mmol) were sequentially added. Then, 10.0 mL of triethylamine was added. The mixture was stirred overnight at room temperature under a nitrogen atmosphere. Post-treatment involved diluting the mixture and adding a saturated aqueous solution of ammonium chloride, followed by extraction with ethyl acetate (EtOAc). The combined organic layers were washed with a saturated brine, dried over sodium sulfate (Na_2SO_4), filtered, and the solvents were removed under reduced pressure. The residue was purified by flash column chromatography (silica gel, petroleum ether /ethyl acetate = 5:1) to obtain the product.[1]

Characterization data of starting materials:

3-Phenyl-2-propyn-1-ol (1a)

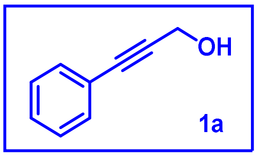

Light yellow oil, 0.56 g, 85% yield, eluting with 20% ethyl acetate: petroleum ether. **^{1}H NMR** (400 MHz, Chloroform-*d*) δ 7.46 – 7.43 (m, 2H), 7.33 – 7.29 (m, 3H), 4.50 (s, 2H), 2.39 (s, 1H). **^{13}C NMR** (101 MHz, Chloroform-*d*) δ 131.7, 128.5, 128.3, 122.6, 87.3, 85.7, 51.5. **HRMS** calculated for C_9H_9O $[M+H]^+$ 133.0648, found 133.0650. The data are in agreement with those previously reported in the literature.[2]

3-*p*-Tolylprop-2-yn-1-ol (1b)

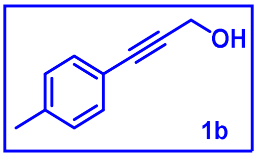

Orange oil, 0.58 g, 80% yield, eluting with 20% ethyl acetate: petroleum ether.**^{1}H NMR** (400 MHz, Chloroform-*d*) δ 7.33 (d, J = 8.1 Hz, 2H), 7.12 (d, J = 7.9 Hz, 2H), 4.49 (s, 2H), 2.35 (s, 3H), 1.79 (s, 1H). **^{13}C NMR** (101 MHz, Chloroform-*d*) δ 138.8, 131.7, 129.2, 119.5, 86.6, 85.9, 51.8, 21.6. **HRMS** calculated for $C_{10}H_{11}O$ $[M+H]^+$ 147.0805, found 147.0810. The data are in agreement with those previously reported in the literature.[2]

3-(4-Ethylphenyl) prop-2-yn-1-ol (1c)

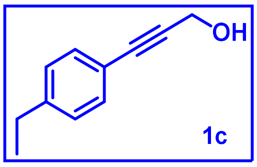

Orange oil, 0.65 g, 82% yield, eluting with 20% ethyl acetate: petroleum ether. ^{1}**H NMR** (400 MHz, Chloroform-*d*) δ 7.36 (d, *J* = 8.2 Hz, 2H), 7.14 (d, *J* = 8.2 Hz, 2H), 4.49 (s, 2H), 2.64 (q, *J* = 7.6 Hz, 2H), 1.88 (s, 1H), 1.23 (t, *J* = 7.6 Hz, 3H).^{13}**C NMR** (101 MHz, Chloroform-*d*) δ 144.2, 131.0, 127.2, 118.9, 85.8, 85.2, 51.0, 28.1, 14.6. **HRMS** calculated for $C_{11}H_{13}O$ $[M+H]^+$ 161.0961, found 161.0963. The data are in agreement with those previously reported in the literature.[2]

4-(4-Methoxyphenyl) prop-2-yn-1-ol (1d)

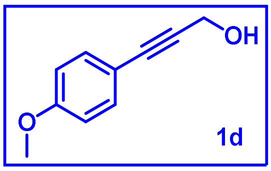

Colorless crystals, 0.65 g, 80% yield, eluting with 20% ethyl acetate: petroleum ether.^{1}**H NMR** (400 MHz, Chloroform-*d*) δ 7.39 – 7.31 (m, 2H), 6.85 – 6.77 (m, 2H), 4.46 (s, 2H), 3.78 (s, 3H), 2.17 (s, 1H). ^{13}**C NMR** (101 MHz, Chloroform-*d*) δ 159.8, 133.3, 114.7, 114.0, 86.0, 85.7, 55.4, 51.7. **HRMS** calculated for $C_{10}H_{11}O_2$ $[M+H]^+$ 163.0754, found 163.0754. The data are in agreement with those previously reported in the literature.[2]

3-(4-Ethoxyphenyl) prop-2-yn-1-ol (1e)

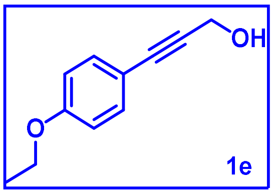

Light yellow solid, 0.66 g, 75% yield, eluting with 20% ethyl acetate: petroleum ether. ^{1}**H NMR** (400 MHz, Chloroform-*d*) δ 7.36 – 7.32 (m, 2H), 6.83 – 6.78 (m, 2H), 4.47 (s, 2H), 4.01 (q, *J* = 7.0 Hz, 2H), 2.13 (s, 1H), 1.40 1.40 (t, *J* = 7.0 Hz, 3H). ^{13}**C NMR** (101 MHz, Chloroform-*d*) δ 158.4, 132.5, 113.7, 113.6, 85.1, 84.9, 62.8, 50.9, 14.0. **HRMS** calculated for $C_{11}H_{13}O_2$ $[M+H]^+$ 177.0911, found 177.0910. The data are in agreement with those previously reported in the literature.[2]

3-Mesitylprop-2-yn-1-ol (1f)

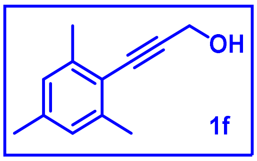

Colorless crystals, 0.52 g, 60% yield, eluting with 20% ethyl acetate: petroleum ether. ^{1}**H NMR** (400 MHz, Chloroform-*d*) δ 6.86 (s, 2H), 4.58 (s, 2H), 2.39 (s, 6H), 2.28 (s, 3H), 1.80 (s, 1H). ^{13}**C NMR** (101 MHz, Chloroform-*d*) δ 140.3, 137.9, 127.6, 119.3, 94.7, 83.5, 51.9, 21.3, 21.0. **HRMS** calculated for $C_{12}H_{15}O$ $[M+H]^+$ 175.1118, found 175.1115.The data are in agreement with

those previously reported in the literature.[1]

3-(4-(*tert*-Butyl) phenyl) prop-2-yn-1-ol (1g)

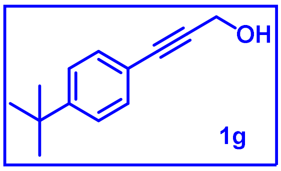

Brown solid, 0.69 g, 74% yield, eluting with 20% ethyl acetate: petroleum ether. **^{1}H NMR** (400 MHz, Chloroform-*d*) δ 7.38 (d, *J* = 8.6 Hz, 2H), 7.33 (d, *J* = 8.5 Hz, 2H), 4.50 (s, 2H), 1.86 (s, 1H), 1.31 (s, 9H). **^{13}C NMR** (101 MHz, Chloroform-*d*) δ 151.0, 130.7, 124.6, 118.7, 85.8, 85.1, 51.0, 34.0, 30.4. **HRMS** calculated for $C_{13}H_{17}O$ $[M+H]^+$ 189.1274, found 189.1276. The data are in agreement with those previously reported in the literature.[1]

3-(4-(*N, N*-dimethylamino) phenyl)prop-2-yn-1-ol (1h)

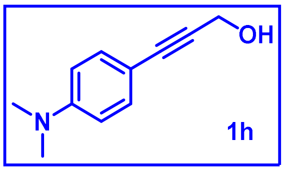

Gray-white crystals, 0.51 g, 58% yield, eluting with 25% ethyl acetate: petroleum ether.**^{1}H NMR** (400 MHz, Chloroform-*d*) δ 7.36 – 7.28 (m, 2H), 6.66 – 6.58 (m, 2H), 4.47 (d, *J* = 5.6 Hz, 2H), 2.96 (s, 6H), 1.99 (s, 1H). **^{13}C NMR** (101 MHz, Chloroform-*d*) δ 150.3, 132.8, 111.8, 109.3, 86.7, 85.1, 51.8, 40.2. **HRMS** calculated for $C_{11}H_{14}NO$ $[M+H]^+$ 176.1070, found 176.1072. The data are in agreement with those previously reported in the literature.[4]

3-(4-Aminophenyl)-2-propyn-1-ol (1i)

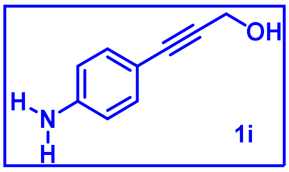

Brown solid, 0.48 g, 65% yield, eluting with 30% ethyl acetate: petroleum ether. **^{1}H NMR** (400 MHz, Chloroform-*d*) δ 7.31 – 7.22 (m, 2H), 6.74 – 6.58 (m, 2H), 4.48 (s, 2H), 3.84 (s, 2H), 1.87 (s, 1H). **^{13}C NMR** (101 MHz, Chloroform-*d*) δ 146.8, 133.1, 114.7, 111.9, 86.3, 85.1, 51.8. **HRMS** calculated for $C_9H_{10}NO$ $[M+H]^+$ 148.0757, found 148.0756. The data are in agreement with those previously reported in the literature.[5]

3-(4-Trifluoromethylphenyl) prop-2-yn-1-ol (1j)

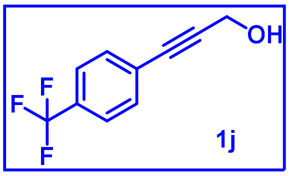

Light yellow solid, 0.65 g, 65% yield, eluting with 20% ethyl acetate: petroleum ether. **^{1}H NMR** (400 MHz, Chloroform-*d*) δ 7.58 – 7.48 (m, 4H), 4.54 (s, 2H), 2.91 (s, 1H).**^{13}C NMR** (101 MHz,

Chloroform-*d*) δ 131.8, 130.2 (q, *J* = 32.7 Hz), 127.8, 126.3 (d, *J* = 1.8 Hz), 125.2 (q, *J* = 3.7 Hz), 123.5 (q, *J* = 272.7 Hz), 122.5, 119.8, 89.7, 84.2, 51.3. ^{19}**F NMR** (376 MHz, Chloroform-*d*) δ -63.0. **HRMS** calculated for $C_{10}H_8F_3O$ $[M+H]^+$ 201.0522, found 201.0521. The data are in agreement with those previously reported in the literature.[2]

Methyl 4-(3-hydroxypropynyl) benzoate (1k)

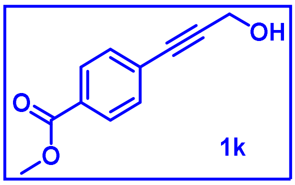

Colorless crystals, 0.72 g, 76% yield, eluting with 20% ethyl acetate: petroleum ether. ^{1}**H NMR** (400 MHz, Chloroform-*d*) δ 7.93 (d, *J* = 8.4 Hz, 2H), 7.42 (d, *J* = 8.5 Hz, 2H), 4.49 (d, *J* = 4.3 Hz, 2H), 3.88 (s, 3H), 2.74 (s, 1H). ^{13}**C NMR** (101 MHz, Chloroform-*d*) δ 166.7, 131.6, 129.6, 129.5, 127.4, 90.5, 84.7, 52.3, 51.4. **HRMS** calculated for $C_{11}H_{11}O_3$ $[M+H]^+$ 191.0703, found 191.0701. The data are in agreement with those previously reported in the literature.[6]

3-(4-Fluoro-phenyl)-prop-2-yn-1-ol (1l)

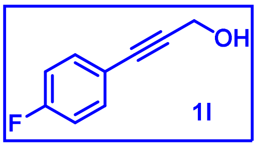

Colorless oil, 0.49 g, 66% yield, eluting with 20% ethyl acetate: petroleum ether. ^{1}**H NMR** (400 MHz, Chloroform-*d*) δ 7.45 – 7.36 (m, 2H), 7.03 – 6.97 (m, 2H), 4.48 (s, 2H), 2.00 (s, 1H). ^{13}**C NMR** (101 MHz, Chloroform-*d*) δ 162.6 (d, *J* = 249.7 Hz), 133.6 (d, *J* = 8.3 Hz), 118.6 (d, *J* = 3.4 Hz), 115.6 (d, *J* = 22.2 Hz), 87.0, 84.6, 51.4. ^{19}**F NMR** (376 MHz, Chloroform-*d*) δ -110.6. **HRMS** calculated for C_9H_8FO $[M+H]^+$ 151.0554, found 151.0556. The data are in agreement with those previously reported in the literature.[2]

3-(4-Chlorophenyl) prop-2-yn-1-ol (1m)

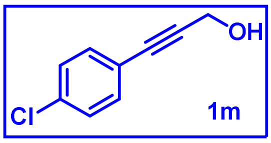

Yellow oil, 0.60 g, 73% yield, eluting with 20% ethyl acetate: petroleum ether. ^{1}**H NMR** (400 MHz, Chloroform-*d*) δ 7.39 – 7.31 (m, 2H), 7.31 – 7.23 (m, 2H), 4.49 (s, 2H), 2.32 (s, 1H). ^{13}**C NMR** (101 MHz, Chloroform-*d*) δ 133.8, 132.2, 128.0, 120.3, 87.4, 83.8, 50.8. **HRMS** calculated for C_9H_8ClO $[M+H]^+$ 167.0259, found 167.0256. The data are in agreement with those previously reported in the literature.[2]

3-(Naphthalen-2-yl) prop-2-yn-1-ol (1n)

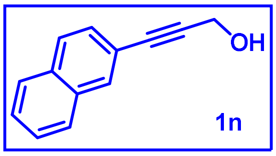

Colorless oil, 0.65 g, 72% yield, eluting with 20% ethyl acetate: petroleum ether. ^{1}**H NMR** (400

MHz, Chloroform-*d*) δ 7.97 (s, 1H), 7.85 – 7.74 (m, 3H), 7.53 – 7.45 (m, 3H), 4.56 (s, 2H), 2.05 (d, *J* = 3.3 Hz, 1H). **^{13}C NMR** (101 MHz, Chloroform-*d*) δ 132.9, 131.7, 128.3, 128.0, 127.8, 127.8, 126.8, 126.6, 119.8, 87.6, 86.1, 51.7. **HRMS** calculated for $C_{13}H_{11}O$ $[M+H]^+$ 183.0805, found 183.0804. The data are in agreement with those previously reported in the literature.[7]

3-(Phenanthren-9-yl) prop-2-yn-1-ol (1o)

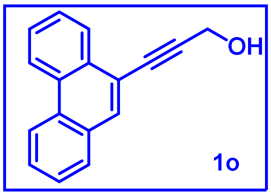

Yellow oil, 0.75 g, 65% yield, eluting with 20% ethyl acetate: petroleum ether.**^{1}H NMR** (400 MHz, Chloroform-*d*) δ 8.72 – 8.61 (m, 2H), 8.48 – 8.38 (m, 1H), 7.99 (s, 1H), 7.87 – 7.54 (m, 5H), 4.69 (d, *J* = 4.8 Hz, 2H), 2.01 (d, *J* = 5.5 Hz, 1H).**^{13}C NMR** (101 MHz, Chloroform-*d*) δ 132.3, 131.1, 131.1, 130.4, 130.1, 128.6, 127.6, 127.1, 127.1, 127.0, 126.8, 122.8, 122.6, 118.9, 91.7, 84.0, 51.9. **HRMS** calculated for $C_{17}H_{13}O$ $[M+H]^+$ 233.0961, found 233.0964. The data are in agreement with those previously reported in the literature.[7]

3-Thiophen-2-yl-prop-2-yn-1-ol (1p)

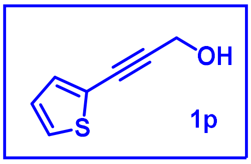

Yellow oil, 0.48 g, 70% yield, eluting with 20% ethyl acetate: petroleum ether. **^{1}H NMR** (400 MHz, Chloroform-*d*) δ 7.22 – 7.12 (m, 2H), 6.91 – 6.89 (m, 1H), 4.44 (d, *J* = 5.7 Hz, 2H), 1.52 (s, 1H). **^{13}C NMR** (101 MHz, Chloroform-*d*) δ 132.4, 127.4, 127.0, 122.4, 91.1, 79.1, 51.7. **HRMS** calculated for C_7H_7OS $[M+H]^+$ 139.0213, found 139.0215. The data are in agreement with those previously reported in the literature.[7]

3-([1,1'-Biphenyl]-4-yl) prop-2-yn-1-ol (1q)

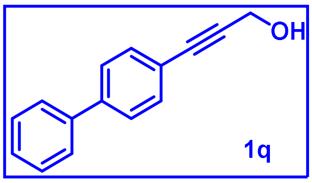

Light yellow solid, 0.78 g, 75% yield, eluting with 20% ethyl acetate: petroleum ether. **^{1}H NMR** (400 MHz, Chloroform-*d*) δ 7.60 – 7.57 (m, 2H), 7.56 (d, *J* = 8.6 Hz, 2H), 7.51 (d, *J* = 8.5 Hz, 2H), 7.46 – 7.43 (m, 2H), 7.40 – 7.33 (m, 1H), 4.53 (d, *J* = 4.4 Hz, 2H), 1.87 (s, 1H). **^{13}C NMR** (101 MHz, Chloroform-*d*) δ 141.3, 140.3, 132.1, 128.9, 127.7, 127.0, 127.0, 121.4, 87.9, 85.6, 51.7. **HRMS** calculated for $C_{15}H_{13}O$ $[M+H]^+$ 209.0961, found 209.0962. The data are in agreement with those previously reported in the literature.[7]

3-(2-Methoxyphenyl) prop-2-yn-1-ol (1r)

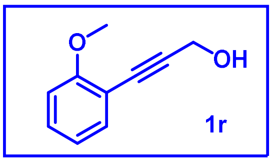

Brown oil, 0.61 g, 75% yield, eluting with 25% ethyl acetate: petroleum ether. ^{1}**H NMR** (400 MHz, Chloroform-*d*) δ 7.43 – 7.40 (m, 1H), 7.35 – 7.26 (m, 1H), 6.96 – 6.85 (m, 2H), 4.56 (s, 2H), 3.90 (s, 3H), 2.40 (s, 1H). ^{13}**C NMR** (101 MHz, Chloroform-*d*) δ 160.0, 133.7, 130.0, 120.5, 111.7, 110.6, 91.5, 81.8, 55.8, 51.8. **HRMS** calculated for $C_{10}H_{11}O_2$ $[M+H]^+$ 163.0754, found 163.0753. The data are in agreement with those previously reported in the literature.[8]

3-(3-Methoxyphenyl) prop-2-yn-1-ol (1s)

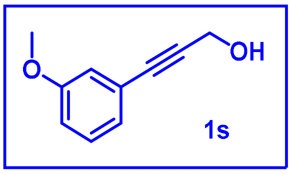

Brown oil, 0.57 g, 70% yield, eluting with 25% ethyl acetate: petroleum ether. ^{1}**H NMR** (400 MHz, Chloroform-*d*) δ 7.23 – 7.19 (m, 1H), 7.04 – 7.02 (m, 1H), 6.98 – 7.96 (m, 1H), 6.89 – 6.86 (m, 1H), 4.49 (d, *J* = 5.1 Hz, 2H), 3.79 (s, 3H), 2.00 (s, 1H). ^{13}**C NMR** (101 MHz, Chloroform-*d*) δ 159.3, 129.4, 124.2, 123.5, 116.6, 115.1, 87.1, 85.6, 55.3, 51.6. **HRMS** calculated for $C_{10}H_{11}O_2$ $[M+H]^+$ 163.0754, found 163.0752. The data are in agreement with those previously reported in the literature.[8]

3-(3,4-Dimethylphenyl) prop-2-yn-1-ol (1t)

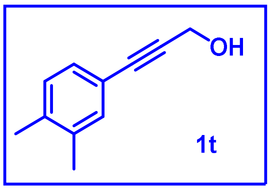

White solid, 0.60 g, 75% yield, eluting with 20% ethyl acetate: petroleum ether. ^{1}**H NMR** (400 MHz, Chloroform-*d*) δ 7.22 (s, 1H), 7.18 (d, *J* = 7.7 Hz, 1H), 7.07 (d, *J* = 7.7 Hz, 1H), 4.48 (d, *J* = 3.8 Hz, 2H), 2.25 (s, 3H), 2.23 (s, 3H), 1.81 (s, 1H). ^{13}**C NMR** (101 MHz, Chloroform-*d*) δ 137.5, 136.7, 132.8, 129.6, 129.1, 119.7, 86.3, 86.0, 51.7, 19.7, 19.5. **HRMS** calculated for $C_{11}H_{13}O$ $[M+H]^+$ 161.0961, found 161.0963. The data are in agreement with those previously reported in the literature.[9]

3-(3,4-Dimethoxyphenyl) prop-2-yn-1-ol (1u)

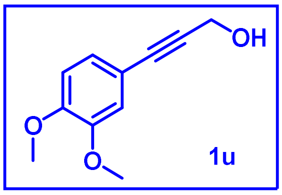

Yellow oil, 0.70 g, 73 % yield, eluting with 30% ethyl acetate: petroleum ether.^{1}**H NMR** (400 MHz, Chloroform-*d*) δ 7.02 (dd, *J* = 8.3, 1.9 Hz, 1H), 6.93 (d, *J* = 1.9 Hz, 1H), 6.77 (d, *J* = 8.3 Hz, 1H), 4.47 (d, *J* = 4.5 Hz, 2H), 3.86 (s, 3H), 3.84 (s, 3H), 2.09 (s, 1H). ^{13}**C NMR** (101 MHz,

Chloroform-*d*) δ 149.6, 148.6, 125.0, 114.7, 114.5, 111.0, 85.8, 85.7, 55.9, 51.6. **HRMS** calculated for $C_{11}H_{13}O_3$ $[M+H]^+$ 193.0860, found 193.0864. The data are in agreement with those previously reported in the literature.[9]

4. General procedures of reactions

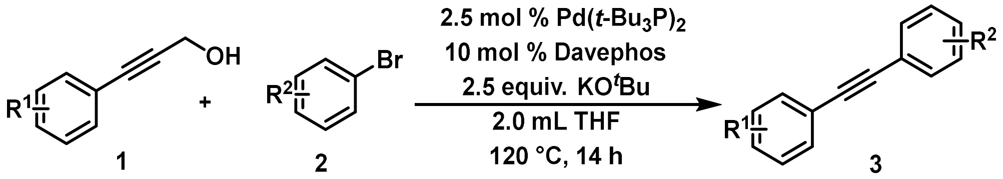

A Schlenk tube (25.0 mL) with a magnetic stir bar was charged with **1** (0.3 mmol, 1.5 equiv.), **2** (0.2 mmol, 1.0 equiv.) , Pd(*t*-$Bu_3P)_2$ (0.005 mmol, 2.5 mol %, 2.5 mg), Davephos (0.02 mmol, 10 mol %, 7.8 mg) and KO*ᵗ*Bu (0.5 mmol, 2.5 equiv, 56.0 mg) and THF (2.0 mL). The Schlenk tube was heated at 120 °C for 14 hours. Then the reaction solution was cooled to ambient temperature and ethyl acetate (2.0 mL) was added. After filtration and evaporation of the solvents under reduced pressure, the residue **3** was purified by column chromatography on silica gel (100% petroleum ether) to provide the desired product.

5. Characterization date of the products

Diphenyl acetylene (3a)

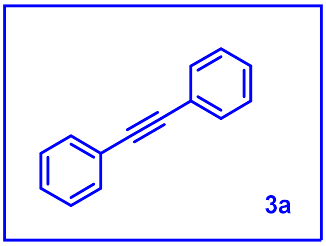

White solid, 26.7 mg, 75% yield, eluting with 100% petroleum ether. **^{1}H NMR** (400 MHz, Chloroform-*d*) δ 7.72 – 7.62 (m, 4H), 7.50 – 7.38 (m, 6H). **^{13}C NMR** (101 MHz, Chloroform-*d*) δ 131.7, 128.5, 128.4, 123.4, 89.6. **HRMS** calculated for $C_{14}H_{11}$ $[M+H]^+$ 179.0856, found 179.0858. The data are in agreement with those previously reported in the literature.[10]

4-Methoxylphenyl phenylacetylene (3b)

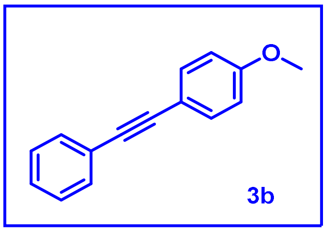

White solid, 29.9 mg, 72% yield, eluting with 100% petroleum ether. **^{1}H NMR** (400 MHz, Chloroform-*d*) δ 7.55 – 7.51 (m, 2H), 7.51 – 7.47 (m, 2H), 7.38 – 7.31 (m, 3H), 6.92 – 6.87 (m, 2H), 3.83 (s, 3H).**^{13}C NMR** (101 MHz, Chloroform-*d*) δ 159.7, 133.1, 131.5, 128.3, 128.0, 123.6, 115.4, 114.0, 89.4, 88.1, 55.3. **HRMS** calculated for $C_{15}H_{13}O$ $[M+H]^+$ 209.0961, found 209.0961. The data are in agreement with those previously reported in the literature.[11]

2-Methoxydiphenylacetylene (3c)

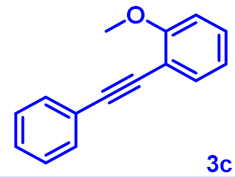

3c

Yellow solid, 26.2 mg, 63% yield, eluting with 100% petroleum ether. **^{1}H NMR** (400 MHz, Chloroform-*d*) δ 7.60 – 7.55 (m, 2H), 7.52 (dd, *J* = 7.6, 1.8 Hz, 1H), 7.38 – 7.29 (m, 4H), 6.95 – 6.91 (m, 2H), 3.92 (s, 3H). **^{13}C NMR** (101 MHz, Chloroform-*d*) δ 160.0, 133.6, 131.7, 129.8, 128.3, 128.1, 123.6, 120.5, 112.5, 110.7, 93.5, 85.8, 55.9. **HRMS** calculated for $C_{15}H_{13}O$ $[M+H]^+$ 209.0961, found 209.0962. The data are in agreement with those previously reported in the literature.[12]

1-Methoxy-3-(phenylethynyl) benzene (3d)

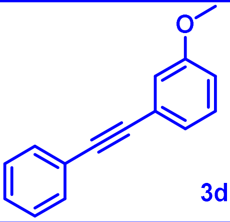

3d

Pale yellow oil, 24.1 mg, 58% yield, eluting with 100% petroleum ether. **^{1}H NMR** (400 MHz, Chloroform-*d*) δ 7.68 – 7.58 (m, 2H), 7.46 – 7.38 (m, 3H), 7.35 – 7.30 (m, 1H), 7.25 – 7.22 (m, 1H), 7.17 – 7.16 (m, 1H), 7.00 – 6.96 (m, 1H), 3.88 (s, 3H). **^{13}C NMR** (101 MHz, Chloroform-*d*) δ 159.5, 131.7, 129.5, 128.5, 128.4, 124.4, 124.3, 123.3,116.5, 116.0, 89.5, 89.3, 55.3. **HRMS** calculated for $C_{15}H_{13}O$ $[M+H]^+$ 209.0961, found 209.0962. The data are in agreement with those previously reported in the literature.[12]

4-Methylphenyl phenylacetylene (3e)

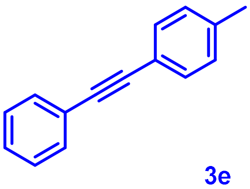

3e

White solid, 26.1 mg, 68% yield, eluting with eluting with 100% petroleum ether. **^{1}H NMR** (400 MHz, Chloroform-*d*) δ 7.59 – 7.49 (m, 2H), 7.44 (d, *J* = 8.0 Hz, 2H), 7.44 – 7.28 (m, 3H), 7.17 (d, *J* = 7.8 Hz, 2H), 2.38 (s, 3H). **^{13}C NMR** (101 MHz, Chloroform-*d*) δ 138.4, 131.6, 131.5, 129.1, 128.3, 128.1, 123.5, 120.2, 89.6, 88.4, 21.5. **HRMS** calculated for $C_{15}H_{13}$ $[M+H]^+$ 193.1012, found 193.1014. The data are in agreement with those previously reported in the literature.[13]

Ethyl-4-(phenylethynyl) benzene (3f)

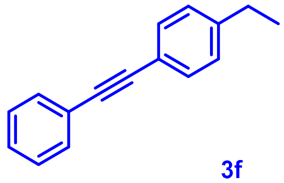

3f

Yellow oil, 26.8 mg, 65% yield, eluting with 100% petroleum ether.^{1}**H NMR** (400 MHz, Chloroform-*d*) δ 7.59 – 7.52 (m, 2H), 7.47 (d, *J* = 8.2 Hz, 2H), 7.37 – 7.33 (m, 3H), 7.20 (d, *J* = 7.9 Hz, 2H), 2.68 (q, *J* = 7.6 Hz, 2H), 1.26 (t, *J* = 7.6 Hz, 3H). ^{13}**C NMR** (101 MHz, Chloroform-*d*) δ 144.8, 131.7, 131.6, 128.4, 128.2, 128.0, 123.6, 120.5, 89.7, 88.8, 28.9, 15.4. **HRMS** calculated for $C_{16}H_{15}$ $[M+H]^+$ 207.1169, found 207.1165. The data are in agreement with those previously reported in the literature.[14]

1-(*tert*-Butyl)-4-(phenylethynyl) benzene (3g)

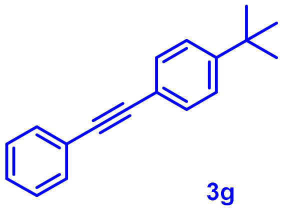

3g

White solid, 37.4 mg, 80% yield, eluting with 100% petroleum ether.^{1}**H NMR** (400 MHz, Chloroform-*d*) δ 7.59 – 7.53 (m, 2H), 7.53 – 7.47 (m, 2H), 7.43 – 7.27 (m, 5H), 1.35 (s, 9H). ^{13}**C NMR** (101 MHz, Chloroform-*d*) δ 151.6, 131.6, 131.4, 128.4, 128.1, 125.4, 123.6, 120.3, 89.6, 88.8, 34.8, 31.2. **HRMS** calculated for $C_{18}H_{19}$ $[M+H]^+$ 235.1482, found 235.1484. The data are in agreement with those previously reported in the literature.[11]

1-(*tert*-Butyl)-4-(phenylethynyl) benzene (3h)

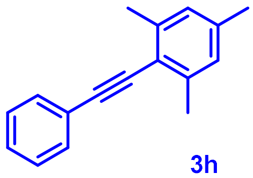

3h

White solid, 30.8 mg, 70% yield, eluting with 100% petroleum ether.^{1}**H NMR** (400 MHz, Chloroform-*d*) δ 7.61 – 7.52 (m, 2H), 7.42 – 7.30 (m, 3H), 6.93 (d, *J* = 8.7 Hz, 2H), 2.52 (d, *J* = 12.6 Hz, 6H), 2.33 (d, *J* = 9.7 Hz, 3H). ^{13}**C NMR** (101 MHz, Chloroform-*d*) δ 140.2, 137.8, 131.4, 128.4, 127.9, 127.6, 127.6, 124.1, 120.0, 97.1, 87.4, 21.4, 21.0. **HRMS** calculated for $C_{17}H_{17}$ $[M+H]^+$ 221.1325, found 221.1327. The data are in agreement with those previously reported in the literature.[13]

4-Aminodiphenylacetylene (3i)

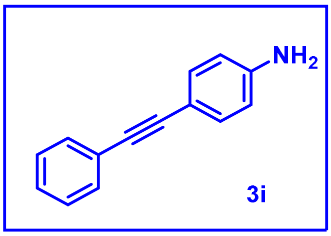

Yellow solid, 11.6 mg, 30% yield, eluting with 20% ethyl acetate: petroleum ether. ^{1}**H NMR** (400 MHz, Chloroform-*d*) δ 7.55 – 7.47 (m, 2H), 7.39 – 7.26 (m, 5H), 6.67 – 6.61 (m, 2H), 3.82 (s, 2H). ^{13}**C NMR** (101 MHz, Chloroform-*d*) δ 146.8, 133.1, 131.5, 128.4, 127.8, 124.0, 114.9, 112.7, 90.2, 87.4. **HRMS** calculated for $C_{14}H_{12}N$ $[M+H]^+$ 194.0965, found 194.0962. The data are in agreement with those previously reported in the literature.[12]

4-*N, N*-dimethyldiphenylacetylene (3j)

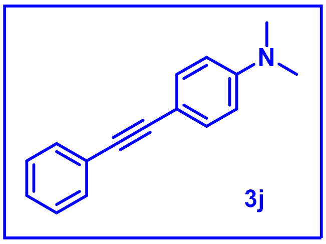

White solid, 11.1 mg, 25% yield, eluting with 30% ethyl acetate: petroleum ether. ^{1}**H NMR** (400 MHz, Chloroform-*d*) δ 7.58 – 7.50 (m, 2H), 7.49 – 7.41 (m, 2H), 7.40 – 7.25 (m, 3H), 6.73 – 6.67 (m, 2H), 3.02 (s, 6H). ^{13}**C NMR** (101 MHz, Chloroform-*d*) δ 150.1, 132.7, 131.3, 128.3, 127.5, 124.2, 111.9, 110.1, 90.6, 87.4, 40.2. **HRMS** calculated for $C_{16}H_{16}N$ $[M+H]^+$ 222.1278, found 222.1276. The data are in agreement with those previously reported in the literature.[14]

4-Nitrodiphenylacetylene (3k)

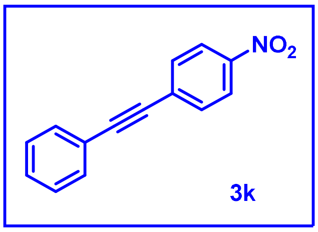

Yellow solid, 25.9 mg, 58% yield, eluting with 2% ethyl acetate: petroleum ether. ^{1}**H NMR** (400 MHz, Chloroform-*d*) δ 8.26 – 8.18 (m, 2H), 7.71 – 7.63 (m, 2H), 7.60 – 7.52 (m, 2H), 7.46 – 7.34 (m, 3H). ^{13}**C NMR** (101 MHz, Chloroform-*d*) δ 147.1, 132.4, 131.9, 130.4, 129.4, 128.6, 123.7, 122.2, 94.8, 87.6. **HRMS** calculated for $C_{14}H_{10}NO_2$ $[M+H]^+$ 224.0707, found 224.0708. The data are in agreement with those previously reported in the literature.[11]

1-(Phenylethynyl)-4-(trifluoromethyl) benzene (3l)

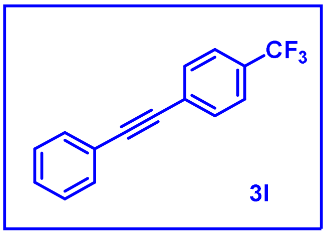

White solid, 33.4 mg, 68% yield, eluting with 100% petroleum ether. ^{1}**H NMR** (400 MHz, Chloroform-*d*) δ 7.70 – 7.59 (m, 4H), 7.60 – 7.54 (m, 2H), 7.45 – 7.33 (m, 3H). ^{13}**C NMR** (101 MHz, Chloroform-*d*) δ 131.8, 131.8, 129.9 (q, *J* = 32.7 Hz), 128.9, 128.5, 127.2, 125.3 (q, *J* = 4.0 Hz), 124.1(q, *J* = 271.7 Hz), 122.6, 91.8, 88.0. ^{19}**F NMR** (376 MHz, Chloroform-*d*) δ -62.7. **HRMS** calculated for $C_{15}H_{10}F_3$ $[M+H]^+$ 247.0730, found 247.0734. The data are in agreement with those previously reported in the literature.[14]

4-(Phenylethynyl) benzonitrile (3m)

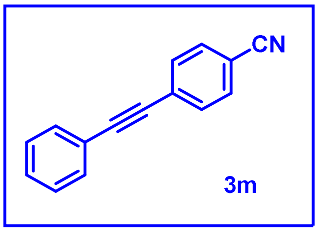

Yellow oil, 21.1 mg, 52% yield, eluting with 2% ethyl acetate: petroleum ether. ^{1}**H NMR** (400 MHz, Chloroform-*d*) δ 7.71 – 7.57 (m, 4H), 7.60 – 7.44 (m, 2H), 7.47 – 7.26 (m, 3H). ^{13}**C NMR** (101 MHz, Chloroform-*d*) δ 132.2, 132.1 132.0, 129.2, 128.6, 128.3, 122.3, 118.6, 111. 6, 93.9, 87.8. **HRMS** calculated for $C_{15}H_{10}N$ $[M+H]^+$ 204.0808, found 204.0807. The data are in agreement with those previously reported in the literature.[12]

4-Chlorodiphenylacetylene (3n)

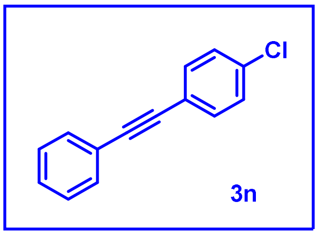

White solid. 18.2 mg, 43% yield, eluting with 100% petroleum ether. ^{1}**H NMR** (400 MHz, Chloroform-*d*) δ 7.57 – 7.52 (m, 2H), 7.49 – 7.44 (m, 2H), 7.38 – 7.35 (m, 3H), 7.35 – 7.31 (m, 2H). ^{13}**C NMR** (101 MHz, Chloroform-*d*) δ 134.4, 132.9, 131.7, 128.8, 128.6, 128.5, 123.0, 121.9, 90.4, 88.4. **HRMS** calculated for $C_{14}H_{10}Cl$ $[M+H]^+$ 213.0466, found 213.0466. The data are in agreement with those previously reported in the literature.[11]

1,2-Dichloro-4-(phenylethynyl) benzene (3o)

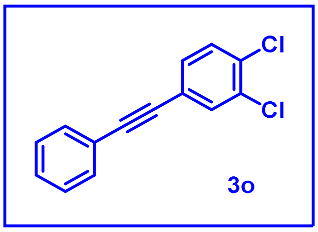

White solid. 19.6 mg, 40% yield, eluting with 100% petroleum ether. ^{1}**H NMR** (400 MHz, Chloroform-*d*) δ 7.63 (d, *J* = 1.9 Hz, 1H), 7.56 – 7.53 (m, 2H), 7.43 – 7.33 (m, 5H). ^{13}**C NMR** (101 MHz, Chloroform-*d*) δ 133.2, 132.6, 132.6, 131.7, 130.7, 130.4, 128.8, 128.5, 123.3, 122.5, 91.4, 87.1. **HRMS** calculated for $C_{14}H_9Cl_2$ $[M+H]^+$ 247.0076, found 247.0078. The data are in agreement with those previously reported in the literature.[15]

1,2-Dimethoxy-4-(phenylethynyl) benzene (3p)

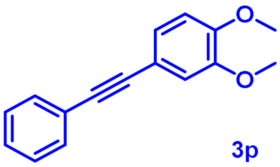

3p

White solid. 28.6 mg, 60% yield, eluting with 1% ethyl acetate: petroleum ether. **^{1}H NMR** (400 MHz, Chloroform-*d*) δ 7.60 – 7.47 (m, 2H), 7.39 – 7.25 (m, 3H), 7.13 (dd, *J* = 8.3, 1.9 Hz, 1H), 7.03 (d, *J* = 1.9 Hz, 1H), 6.83 (d, *J* = 8.3 Hz, 1H), 3.89 (d, *J* = 1.7 Hz, 6H). **^{13}C NMR** (101 MHz, Chloroform-*d*) δ 149.5, 148.7, 131.5, 128.4, 128.1, 124.9, 123.5, 115.5, 114.3, 111.1, 89.5, 89.0, 55.9, 55.9. **HRMS** calculated for $C_{16}H_{15}O_2$ [M+H]$^+$ 239.1067, found 239.1064. The data are in agreement with those previously reported in the literature.[12]

4-(Phenylethynyl)-1,1'-biphenyl (3q)

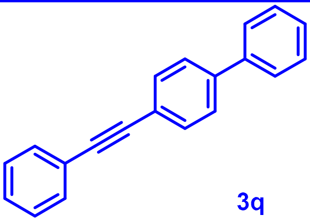

3q

White solid, 30.5 mg, 60% yield, eluting with 3% ethyl acetate: petroleum ether. **^{1}H NMR** (400 MHz, Chloroform-*d*) δ 7.62 (m, 4H), 7.60 – 7.55 (m, 2H), 7.50 – 7.44 (m, 2H), 7.41 – 7.32 (m, 6H). **^{13}C NMR** (101 MHz, Chloroform-*d*) δ 141.1, 140.5, 132.1, 131.7, 129.0, 128.8, 128.5, 128.4, 127.7, 127.1, 123.4, 122.3, 90.2, 89.4. **HRMS** calculated for $C_{20}H_{15}$ [M+H]$^+$ 255.1169, found 255.1165. The data are in agreement with those previously reported in the literature.[12]

2-(Phenylethynyl) naphthalene (3r)

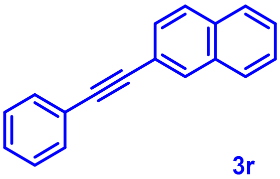

3r

Colorless oil, 23.3 mg, 51% yield, eluting with 1% ethyl acetate: petroleum ether. **^{1}H NMR** (400 MHz, Chloroform-*d*) δ 8.07 (s, 1H), 7.86 – 7.80 (m, 3H), 7.63 – 7.57 (m, 3H), 7.54 – 7.48 (m, 2H), 7.39 – 7.35 (m, 3H). **^{13}C NMR** (101 MHz, Chloroform-*d*) δ 133.1, 132.9, 131.8, 131.5, 128.5, 128.5, 128.4, 128.1, 127.9, 126.8, 126.6, 123.4, 120.7, 89.9, 89.8. **HRMS** calculated for $C_{18}H_{13}$ [M+H]$^+$ 229.1012, found 229.1010. The data are in agreement with those previously reported in the literature.[16]

9-(Phenylethynyl) phenanthrene (3s)

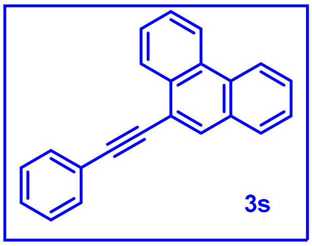

White solid, 25.0 mg, 45% yield, eluting with 1% ethyl acetate: petroleum ether. **^{1}H NMR** (400 MHz, Chloroform-*d*) δ 8.74 – 8.70 (m, 1H), 8.68 (d, *J* = 8.2 Hz, 1H), 8.63 – 8.54 (m, 1H), 8.11 (s, 1H), 7.89 (dd, *J* = 7.8, 1.5 Hz, 1H), 7.75 – 7.70 (m, 4H), 7.70 – 7.65 (m, 1H), 7.64 – 7.60 (m, 1H), 7.53 – 7.37 (m, 3H). **^{13}C NMR** (101 MHz, Chloroform-*d*) δ 132.0, 131.9, 131.4, 131.2, 130.4, 130.2, 128.7, 128.6, 127.6, 127.2, 127.1, 123.5, 122.9, 122.8, 119.7, 94.1, 87.8. **HRMS** calculated for $C_{22}H_{15}$ $[M+H]^{+}$ 279.1169, found 279.1165. The data are in agreement with those previously reported in the literature.[15]

1-(2-(2,4,6-Trimethoxyphenyl) ethynyl)-benzene (3t)

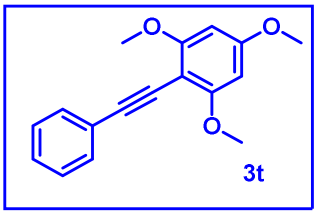

Yellow solid, 27.9 mg, 52% yield, eluting with 3% ethyl acetate: petroleum ether. **^{1}H NMR** (400 MHz, Chloroform-*d*) δ 7.58 – 7.51 (m, 2H), 7.36 – 7.21 (m, 3H), 6.11 (s, 2H), 3.88 (s, 6H), 3.83 (s, 3H). **^{13}C NMR** (101 MHz, Chloroform-*d*) δ 162.3, 161.7, 131.6, 128.2, 127.6, 124.3, 96.4, 94.5, 90.6, 82.1, 56.2, 55.5. **HRMS** calculated for $C_{17}H_{17}O_{3}$ $[M+H]^{+}$ 269.1173, found 269.1174. The data are in agreement with those previously reported in the literature.[17]

1-Methoxy-4-(p-tolylethynyl) benzene (3u)

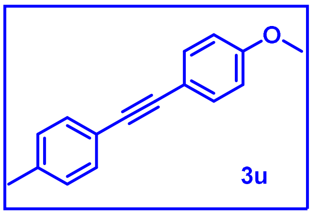

Yello solid, 30.2 mg, 68% yield, eluting with 1% ethyl acetate: petroleum ether. **^{1}H NMR** (400 MHz, Chloroform-*d*) δ 7.48 (d, *J* = 8.9 Hz, 2H), 7.42 (d, *J* = 8.1 Hz, 2H), 7.15 (d, *J* = 7.8 Hz, 2H), 6.88 (d, *J* = 8.8 Hz, 2H), 3.83 (s, 3H), 2.37 (s, 3H). **^{13}C NMR** (101 MHz, Chloroform-*d*) δ 159.5, 138.0, 133.0, 131.4, 129.1, 120.5, 115.6, 114.0, 88.7, 88.2, 55.3, 21.5. **HRMS** calculated for $C_{16}H_{15}O$ $[M+H]^{+}$ 223.1118, found 223.1115. The data are in agreement with those previously reported in the literature.[18]

1-Ethyl-4-((4-methoxyphenyl) ethynyl) benzene (3v)

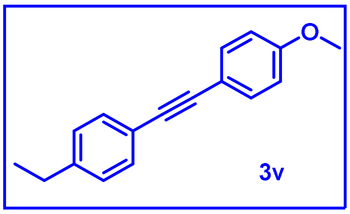

White solid, 30.7 mg, 65% yield, eluting with 1% ethyl acetate: petroleum ether. **^{1}H NMR** (400 MHz, Chloroform-*d*) δ 7.51 – 7.41 (m, 4H), 7.18 (d, *J* = 8.2 Hz, 2H), 6.88 (d, *J* = 8.8 Hz, 2H), 3.83 (s, 3H), 2.67 (q, *J* = 7.6 Hz, 2H), 1.25 (t, *J* = 7.6 Hz, 3H). **^{13}C NMR** (101 MHz, Chloroform-*d*) δ 159.5, 144.3, 133.0, 131.4, 127.9, 120.8, 115.6, 114.0, 88.7, 88.2, 55.3, 28.8, 15.4. **HRMS** calculated for $C_{17}H_{17}O$ $[M+H]^+$ 237.1274, found 237.1275. The data are in agreement with those previously reported in the literature.[19]

Bis (p-methoxyphenyl) acetylene (3w)

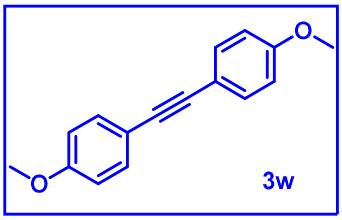

White solid, 33.3 mg, 70% yield, eluting with 1% ethyl acetate: petroleum ether. **^{1}H NMR** (400 MHz, Chloroform-*d*) δ 7.48 (d, *J* = 8.8 Hz, 4H), 6.90 (d, *J* = 8.8 Hz, 4H), 3.85 (s, 6H). **^{13}C NMR** (101 MHz, Chloroform-*d*) δ 159.4, 132.9, 115.7, 114.0, 88.0, 55.3. **HRMS** calculated for $C_{16}H_{15}O_2$ $[M+H]^+$ 239.1067, found 239.1065. The data are in agreement with those previously reported in the literature.[19]

1-Ethoxy-4-((4-methoxyphenyl) ethynyl) benzene (3x)

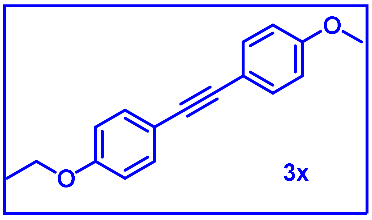

Yello solid, 22.7 mg, 45% yield, eluting with 1% ethyl acetate: petroleum ether. **^{1}H NMR** (400 MHz, Chloroform-*d*) δ 7.49 – 7.40 (m, 4H), 6.91 – 6.80 (m, 4H), 4.05 (q, *J* = 7.0 Hz, 2H), 3.82 (s, 3H), 1.42 (t, *J* = 7.0 Hz, 3H). **^{13}C NMR** (101 MHz, Chloroform-*d*) δ 159.4, 158.8, 132.9, 115.8, 115.5, 114.5, 114.0, 88.0, 87.9, 63.5, 55.3, 14.8. **HRMS** calculated for $C_{17}H_{17}O_2$ $[M+H]^+$ 253.1224, found 253.1226. The data are in agreement with those previously reported in the literature.[20]

2-(2-(4-Methoxyphenyl) ethynyl)-1, 3, 5-trimethylbenzene (3y)

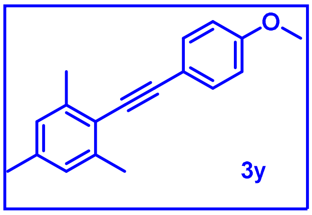

Yellow oil, 30.0 mg, 60% yield, eluting with 1% ethyl acetate: petroleum ether. ^{1}**H NMR** (400 MHz, Chloroform-*d*) δ 7.51 – 7.43 (m, 2H), 6.90 – 6.90 (m, 4H), 3.84 (s, 3H), 2.47 (s, 6H), 2.30 (s, 3H). ^{13}**C NMR** (101 MHz, Chloroform-*d*) δ 159.4, 139.9, 137.4, 132.7, 127.6, 120.3, 116.2, 114.0, 97.0, 86.0, 55.3, 21.3, 21.0. **HRMS** calculated for $C_{18}H_{19}O$ $[M+H]^+$ 251.1431, found 251.1433. The data are in agreement with those previously reported in the literature.[21]

1-(*tert*-Butyl)-4-((4-methoxyphenyl) ethynyl) benzene (3z)

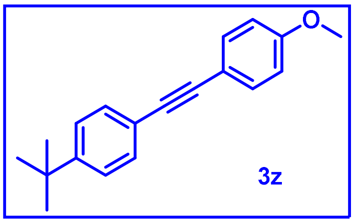

White solid, 41.2 mg, 78% yield, eluting with 1% ethyl acetate: petroleum ether. ^{1}**H NMR** (400 MHz, Chloroform-*d*) δ 7.50 – 7.42 (m, 4H), 7.36 (d, *J* = 8.3 Hz, 2H), 6.88 (d, *J* = 8.7 Hz, 2H), 3.83 (s, 3H), 1.33 (s, 9H). ^{13}**C NMR** (101 MHz, Chloroform-*d*) δ 159.5, 151.2, 133.0, 131.2, 125.3, 120.6, 115.7, 114.0, 88.7, 88.2, 55.3, 34.8, 31.2. **HRMS** calculated for $C_{19}H_{21}O$ $[M+H]^+$ 265.1587, found 265.1584. The data are in agreement with those previously reported in the literature.[11]

4-((4-Methoxyphenyl) ethynyl)-*N, N*-dimethylaniline (3aa)

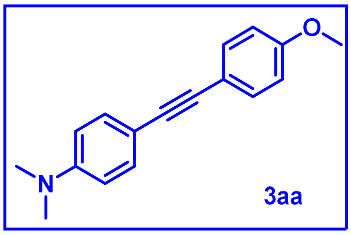

White solid, 31.6 mg, 63% yield, eluting with 10% ethyl acetate: petroleum ether. ^{1}**H NMR** (400 MHz, Chloroform-*d*) δ 7.48 – 7.32 (m, 4H), 6.86 (d, *J* = 8.8 Hz, 2H), 6.66 (d, *J* = 8.9 Hz, 2H), 3.82 (s, 3H), 2.99 (s, 6H). ^{13}**C NMR** (101 MHz, Chloroform-*d*) δ 159.1, 149.9, 132.7, 132.5, 116.3, 113.9, 111.9, 89.0, 87.1, 55.3, 40.3. **HRMS** calculated for $C_{17}H_{18}NO$ $[M+H]^+$ 252.1383, found 252.1384. The data are in agreement with those previously reported in the literature.[22]

4-((4-Methoxyphenyl) ethynyl) aniline (3ab)

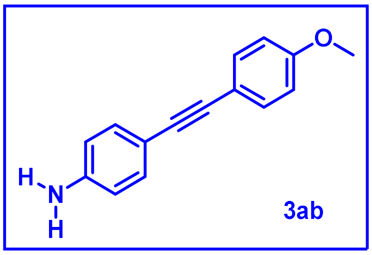

White solid, 16.9 mg, 38% yield, eluting with 10% ethyl acetate: petroleum ether. **^{1}H NMR** (400 MHz, Chloroform-*d*) δ 7.43 (d, *J* = 8.8 Hz, 2H), 7.32 (d, *J* = 8.5 Hz, 2H), 6.86 (d, *J* = 8.8 Hz, 2H), 6.63 (d, *J* = 8.5 Hz, 2H), 3.82 (s, 3H), 3.79 (s, 2H). **^{13}C NMR** (101 MHz, Chloroform-*d*) δ 159.2, 146.4, 132.8, 132.8, 116.1, 114.8, 113.9, 113.0, 88.6, 87.1, 55.3. **HRMS** calculated for $C_{15}H_{14}NO$ $[M+H]^+$ 224.1070, found 224.1074. The data are in agreement with those previously reported in the literature.[23]

1-Methoxy-4-((4-(trifluoromethyl) phenyl) ethynyl) benzene (3ac)

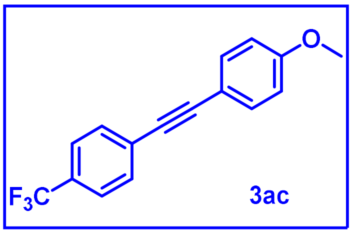

White solid, 11.0 mg, 20% yield, eluting with 1% ethyl acetate: petroleum ether. **^{1}H NMR** (400 MHz, Chloroform-*d*) δ 7.65 – 7.56 (m, 4H), 7.55 – 7.47 (m, 2H), 6.95 – 6.87 (m, 2H), 3.83 (s, 3H). **^{13}C NMR** (101 MHz, Chloroform-*d*) δ 160.1, 133.3, 131.6, 129.5 (q, *J* = 32.6 Hz), 127.5 (d, *J* = 1.6 Hz), 125.2 (q, *J* = 3.8 Hz), 124.1 (q, *J* = 272.1 Hz), 114.6, 114.1, 92.0, 86.9, 55.3. **^{19}F NMR** (376 MHz, Chloroform-*d*) δ -62.7. **HRMS** calculated for $C_{16}H_{12}F_3O$ $[M+H]^+$ 277.0835, found 277.0834. The data are in agreement with those previously reported in the literature.[24]

Methyl 4-((4-methoxyphenyl) ethynyl) benzoate (3ad)

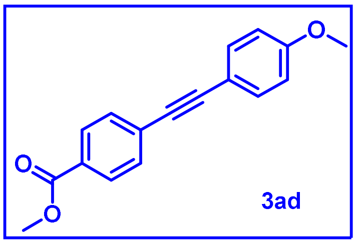

White solid. 13.3 mg, 25% yield, eluting with 1% ethyl acetate: petroleum ether. **^{1}H NMR** (400 MHz, Chloroform-*d*) δ 8.01 (d, *J* = 8.4 Hz, 2H), 7.56 (d, *J* = 8.4 Hz, 2H), 7.48 (d, *J* = 8.8 Hz, 2H), 6.89 (d, *J* = 8.8 Hz, 2H), 3.92 (s, 3H), 3.84 (s, 3H). **^{13}C NMR** (101 MHz, Chloroform-*d*) δ 166.6,160.0, 133.3, 131.3, 129.5, 129.1, 128.4, 114.8, 114.1, 92.6, 87.5, 55.3, 52.2. **HRMS** calculated for $C_{17}H_{15}O_3$ $[M+H]^+$ 267.1016, found 267.1018. The data are in agreement with those previously reported in the literature.[25]

1-Fluoro-4-((4-methoxyphenyl) ethynyl) benzene (3ae)

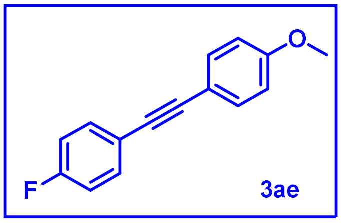

3ae

Pale yellow solid, 21.7 mg, 48% yield, eluting with 1% ethyl acetate: petroleum ether. ^{1}**H NMR** (400 MHz, Chloroform-*d*) δ 7.54 – 7.48 (m, 4H), 7.10 – 7.00 (m, 2H), 6.94 – 6.86 (m, 2H), 3.81 (s, 3H). ^{13}**C NMR** (101 MHz, Chloroform-*d*) δ 162.4 (d, *J* = 249.0 Hz), 160.0, 133.3 (d, *J* = 8.2 Hz), 133.1, 119.8 (d, *J* = 3.6 Hz), 115.6 (d, *J* = 22.1 Hz), 115.2, 114.1, 89.2, 87.1, 55.2. ^{19}**F NMR** (376 MHz, Chloroform-*d*) δ -111.3. **HRMS** calculated for $C_{15}H_{12}FO$ $[M+H]^+$ 227.0867, found 227.0864. The data are in agreement with those previously reported in the literature.[13]

1-Chloro-4-(2-(4-methoxyphenyl) ethynyl) benzene (3af)

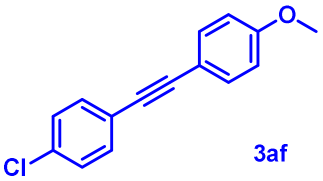

3af

White solid, 26.1 mg, 54% yield, eluting with 1% ethyl acetate: petroleum ether. ^{1}**H NMR** (400 MHz, Chloroform-*d*) δ 7.55 – 7.42 (m, 4H), 7.37 – 7.30 (m, 2H), 6.95 – 6.87 (m, 2H), 3.85 (s, 3H). ^{13}**C NMR** (101 MHz, Chloroform-*d*) δ 159.8, 133.9, 133.1, 132.7, 128.7, 122.2, 115.0, 114.1, 90.4, 87.0, 55.3. **HRMS** calculated for $C_{15}H_{12}ClO$ $[M+H]^+$ 243.0572, found 243.0570. The data are in agreement with those previously reported in the literature.[26]

2-((4-Methoxyphenyl) ethynyl) naphthalene (3ag)

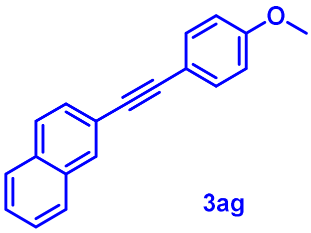

3ag

White solid, 31.0 mg, 60% yield, eluting with 1% ethyl acetate: petroleum ether. ^{1}**H NMR** (400 MHz, Chloroform-*d*) δ 8.04 (s, 1H), 7.86 – 7.77 (m, 3H), 7.60 – 7.44 (m, 5H), 6.91 (d, *J* = 8.9 Hz, 2H), 3.84 (s, 3H). ^{13}**C NMR** (101 MHz, Chloroform-*d*) δ 159.7, 133.1, 133.1, 132.7, 131.1, 128.4, 128.0, 127.8, 127.7, 126.5, 121.0, 115.4, 114.1, 89.8, 88.5, 55.3. **HRMS** calculated for $C_{19}H_{15}O$ $[M+H]^+$ 259.1118, found 259.1116. The data are in agreement with those previously reported in the literature.[21]

9-((4-Methoxyphenyl) ethynyl) phenanthrene (3ah)

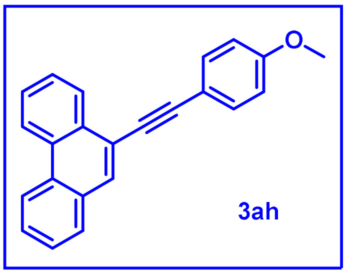

White solid, 24.6 mg, 40% yield, eluting with 1% ethyl acetate: petroleum ether. **^{1}H NMR** (400 MHz, Chloroform-*d*) δ 8.75 – 8.64 (m, 2H), 8.60 – 8.51 (m, 1H), 8.07 (s, 1H), 7.88 (d, *J* = 7.8 Hz, 1H), 7.78 – 7.56 (m, 6H), 6.95 (d, *J* = 8.8 Hz, 2H), 3.87 (s, 3H). **^{13}C NMR** (101 MHz, Chloroform-*d*) δ 159.8, 133.2, 131.4, 131.2, 130.2, 130.1, 128.5, 127.3, 127.0, 127.0, 122.8, 122.6, 120.0, 115.5, 114.1, 94.0, 86.4, 55.4. **HRMS** calculated for $C_{23}H_{17}O$ $[M+H]^+$ 309.1274, found 309.1271. The data are in agreement with those previously reported in the literature.[13]

2-((4-Methoxyphenyl) ethynyl) thiophene (3ai)

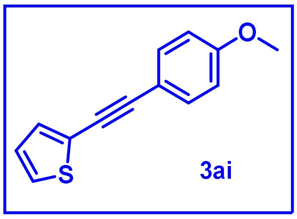

Yellow oil, 18.4 mg, 43% yield, eluting with 5% ethyl acetate: petroleum ether. **^{1}H NMR** (400 MHz, Chloroform-*d*) δ 7.49 (d, *J* = 8.9 Hz, 2H), 7.32 – 7.25 (m, 2H), 7.03 (m, 1H), 6.91 (d, *J* = 8.8 Hz, 2H), 3.85 (s, 3H). **^{13}C NMR** (101 MHz, Chloroform-*d*) δ 159.8, 133.0, 131.5, 127.1, 126.8, 123.7, 115.0, 114.1, 93.0, 81.3, 55.3. **HRMS** calculated for $C_{13}H_{11}OS$ $[M+H]^+$ 215.0526, found 215.0526. The data are in agreement with those previously reported in the literature.[23]

Phenyl 4-((4-methoxyphenyl) ethynyl) benzoate (3aj)

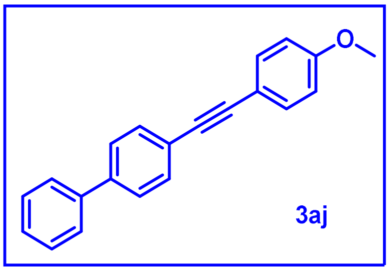

Yellow solid, 23.3 mg, 41% yield, eluting with 5% ethyl acetate: petroleum ether. **^{1}H NMR** (400 MHz, Chloroform-*d*) δ 7.66 – 7.58 (m, 6H), 7.55 – 7.48 (m, 2H), 7.49 – 7.44 (m, 2H), 7.41 – 7.33 (m, 1H), 6.94 – 6.87 (m, 2H), 3.84 (s, 3H). **^{13}C NMR** (101 MHz, Chloroform-*d*) δ 159.7, 140.6, 140.4, 133.1, 131.9, 128.9, 127.6, 127.0, 122.6, 115.4, 114.1, 90.1, 88.1, 55.3. **HRMS** calculated for $C_{21}H_{17}O$ $[M+H]^+$ 285.1274, found 285.1275. M. P: 149.5 – 150 °C.

3,4'-Dimethoxytolan (3ak)

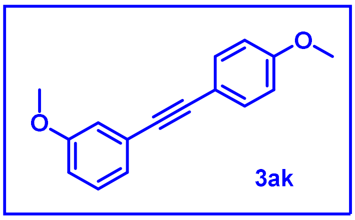

Yellow oil, 30.5 mg, 64% yield, eluting with 3% ethyl acetate: petroleum ether. **^{1}H NMR** (400 MHz, Chloroform-*d*) δ 7.51 – 7.43 (m, 2H), 7.31 – 7.19 (m, 1H), 7.13 – 7.08 (m, 1H), 7.05 – 7.02 (m, 1H), 6.90 – 6.83 (m, 1H), 6.87 – 6.84 (m, 2H), 3.81 (s, 3H), 3.81 (s, 3H). **^{13}C NMR** (101 MHz, Chloroform-*d*) δ 159.7, 159.4, 133.1, 129.4, 124.6, 124.1, 116.2, 115.3, 114.6, 114.0, 89.3, 88.1, 55.3, 55.3. **HRMS** calculated for $C_{16}H_{15}O_2$ $[M+H]^+$ 239.1067, found 239.1065. The data are in agreement with those previously reported in the literature.[21]

1-Methoxy-2-[(4-methoxyphenyl) ethynyl] benzene (3al)

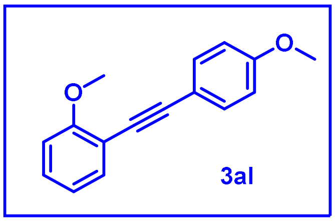

Yellow solid, 29.5 mg, 62% yield, eluting with 3% ethyl acetate: petroleum ether. **^{1}H NMR** (400 MHz, Chloroform-*d*) δ 7.55 – 7.44 (m, 3H), 7.31 – 7.26 (m, 1H), 7.00 – 6.84 (m, 4H), 3.91 (s, 3H), 3.82 (s, 3H). **^{13}C NMR** (101 MHz, Chloroform-*d*) δ 159.8, 159.5, 133.5, 133.1, 129.5, 120.5, 115.7, 113.9, 112.8, 110.7, 93.5, 84.4, 55.8, 55.3. **HRMS** calculated for $C_{16}H_{15}O_2$ $[M+H]^+$ 239.1067, found 239.1065. The data are in agreement with those previously reported in the literature.[21]

1-((4-Methoxyphenyl) ethynyl)-3,5-dimethylbenzene (3am)

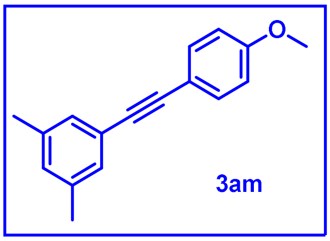

White solid, 19.8 mg, 42% yield, eluting with 3% ethyl acetate: petroleum ether. **^{1}H NMR** (400 MHz, Chloroform-*d*) δ 7.52 – 7.45 (m, 2H), 7.19 (s, 2H), 6.97 (s, 1H), 6.94 – 6.86 (m, 2H), 3.83 (s, 3H), 2.34 (s, 6H). **^{13}C NMR** (101 MHz, Chloroform-*d*) δ 159.6, 137.9, 133.1, 129.9, 129.2, 123.2, 115.6, 114.0, 88.7, 88.5, 55.3, 21.2. **HRMS** calculated for $C_{17}H_{17}O$ $[M+H]^+$ 237.1274, found 237.1276. The data are in agreement with those previously reported in the literature.[27]

1,2-Dimethoxy-4-((4-methoxyphenyl) ethynyl) benzene (3an)

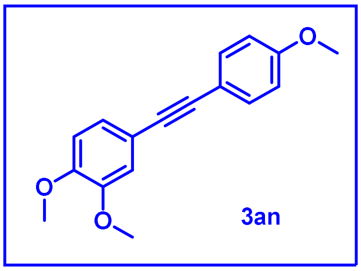

White solid, 25.7 mg, 48% yield, eluting with 3% ethyl acetate: petroleum ether. **^{1}H NMR** (400 MHz, Chloroform-*d*) δ 7.52 – 7.44 (m, 2H), 7.14 (dd, *J* = 8.3, 1.9 Hz, 1H), 7.05 (d, *J* = 1.9 Hz, 1H), 6.92 – 6.87 (m, 2H), 6.85 (d, *J* = 8.3 Hz, 1H), 3.92 (s, 3H), 3.92 (s, 3H), 3.84 (s, 3H). **^{13}C NMR** (101 MHz, Chloroform-*d*) δ 159.5, 149.3, 148.6, 132.9, 124.7, 115.8, 115.6, 114.2, 114.0, 111.0, 88.1, 87.9, 55.9, 55.3. **HRMS** calculated for $C_{17}H_{17}O_3$ $[M+H]^+$ 269.1173, found 269.1174. The data are in agreement with those previously reported in the literature.[28]

6. Synthetic applications

6.1 Gram-scale reaction

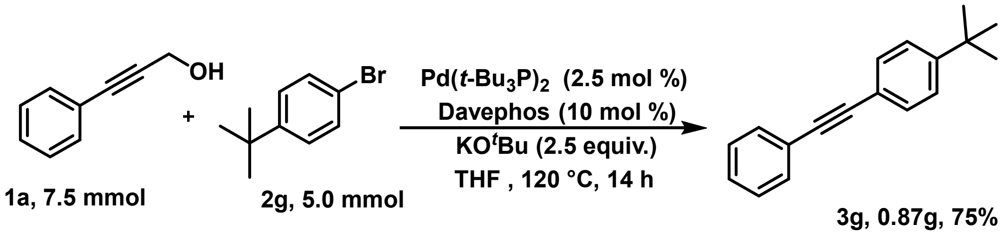

A Schlenk tube (100.0 mL) with a magnetic stir bar was charged with **1a** (7.5 mmol, 1.09 g), **2g** (5.0 mmol, 1.06 g) , Pd(*t*-Bu$_3$P)$_2$ (0.125 mmol, 62.5 mg), Davephos (0.5 mmol, 196.5 mg) , KO^{t}Bu (12.5 mmol, 1.4 g) and THF (30.0 mL). The Schlenk tube was heated at 120 °C for 14 hours. Then the reaction solution was cooled to ambient temperature and ethyl acetate was added. After filtration and evaporation of the solvents under reduced pressure, the residue was purified by column chromatography on silica gel (100% petroleum ether) to provide the desired product **3g** (3.7 mmol, 0.87 g, 75% yield).

6.2 Derivatization of diphenylacetylene 3a

6.2.1 Synthesis of triphenylethylene

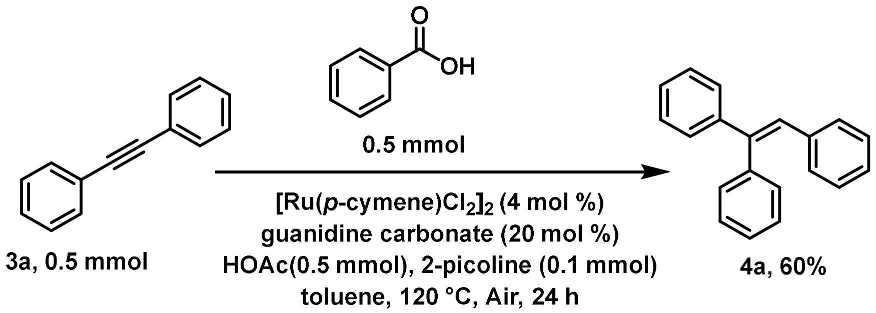

A Schlenk tube (25.0 mL) equipped with a magnetic stir bar was charged with **3a** (0.5 mmol, 89.1 mg), benzoic acid (0.5 mmol, 61.7 mg), [Ru(*p*-cym)Cl$_2$]$_2$ (0.02 mmol, 12.2 mg), guanidine

carbonate (0.1 mmol, 18.2 mg), HOAc (0.5 mmol, 28.9 μL), 2-picoline (0.1 mmol, 9.9 μL) and degassed toluene (2.0 mL) .The Schlenk tube was then heated to 120 °C for 24 hours. After the reaction, the solution was cooled to ambient temperature, brine (20.0 mL) was added and the resulting mixture was extracted with ethyl acetate. The combined organic layers were dried over $MgSO_4$, filtered, and the volatiles were removed under reduced pressure. The purification **4a** was performed by flash column chromatography on silica gel.

Triphenylethylene (4a)

White solid, 0.3 mmol, 76.8 mg, 60% yield, eluting with 100% petroleum ether. **^{1}H NMR** (400 MHz, Chloroform-*d*) δ 7.42 – 7.31 (m, 8H), 7.29 – 7.27 (m, 2H), 7.24 – 7.14 (m, 3H), 7.13 – 7.08 (m, 2H), 7.03 (s, 1H). **^{13}C NMR** (101 MHz, Chloroform-*d*) δ 143.5, 142.7, 140.4, 137.4, 130.5, 130.5, 129.6, 129.6, 128.7, 128.7, 128.3, 128.3, 128.2, 128.0, 128.0, 127.7, 127.7, 127.6, 127.5, 126.8. **HRMS** calculated for $C_{20}H_{17}$ $[M+H]^+$ 257.1325, found 257.1324. The data are in agreement with those previously reported in the literature.[29]

6.2.2 Synthesis of quinoxaline

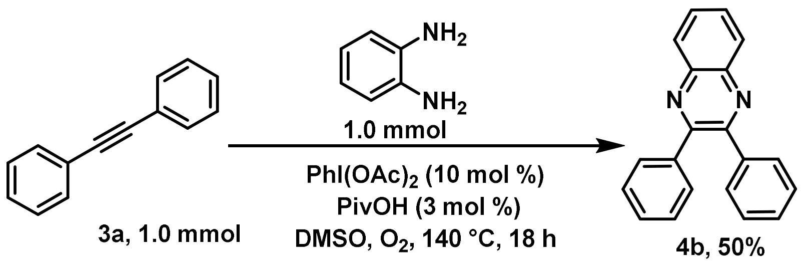

A Schlenk tube (25.0 mL) equipped with a magnetic stir bar was charged with **3a** (1.0 mmol, 178 mg), $PhI(OAc)_2$ (10 mol %, 32.2 mg), 1,2-diaminobenzene derivatives (1.0 mmol, 108 mg), PivOH (3.0 mmol, 678 mg), and dry dimethyl sulfoxide (5.0 mL). The Schlenk tube was then heated to 140 °C and maintained under oxygen atmosphere for 18 hours. After the reaction, the solution was cooled to ambient temperature and poured into a saturated sodium bicarbonate solution (10.0 mL). The aqueous phase was extracted with ethyl acetate, and the combined organic layers were washed with water and brine. The organic layer was dried over Na_2SO_4, filtered, and the volatiles were removed under reduced pressure. The purification **4b** was performed by flash column chromatography on silica gel.

2,3-diphenylquinoxaline (4b)

White solid, 0.5 mmol, 141 mg, 50% yield, eluting with 20% ethyl acetate: petroleum ether. **^{1}H NMR** (400 MHz, Chloroform-*d*) δ 8.22 (dd, *J* = 6.4, 3.4 Hz, 2H), 7.78 (dd, *J* = 6.4, 3.4 Hz, 2H), 7.58 – 7.55 (m, 4H), 7.43 – 7.30 (m, 6H). **^{13}C NMR** (101 MHz, Chloroform-*d*) δ 153.5, 141.3, 139.1, 130.0, 129.9, 129.2, 128.8, 128.3. **HRMS** calculated for $C_{20}H_{14}N_2$ $[M+H]^+$ 283.1230, found 283.1233. The data are in agreement with those previously reported in the literature.[30]

6.2.3 Synthesis of indole compounds

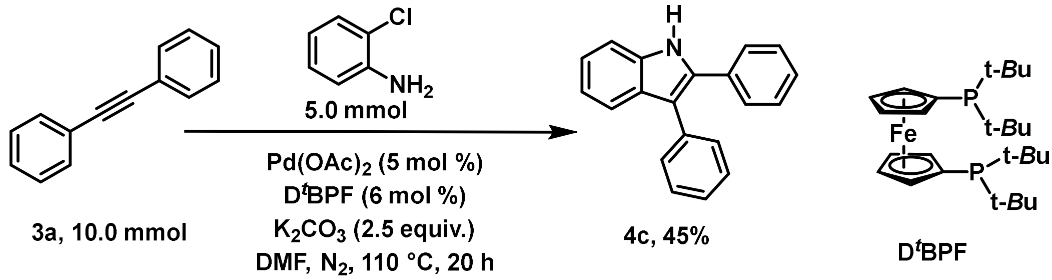

A reaction flask (100.0 mL) equipped with a magnetic stir bar was charged with $Pd(OAc)_2$ (0.25 mmol, 56 mg), D*t*BPF(0.3 mmol, 142 mg), 2-chloro-4-methylaniline (5 mmol, 0.71 g), **3a** (10.0 mmol, 1.78g), K_2CO_3 (12.5 mmol, 1.73 g) and DMF (50.0 mL). The reaction was then heated to 110 °C and maintained under nitrogen atmosphere for 20 hours. The mixture was filtered through a layer of celite and washed with ethyl acetate. The filtrate was diluted with water, extracted with EtOAc. The combined organic phase was washed with water and brine, dried over $MgSO_4$, filtered and concentrated to give a dark brown residue. The purification **4c** was performed by flash column chromatography on silica gel.

2,3-Diphenyl-1H-indole (4c)

Beige solid, 2.2 mmol, 590 mg, 45%, eluting with 20% ethyl acetate: petroleum ether. **^{1}H NMR** (400 MHz, Chloroform-*d*) δ 8.23 (s, 1H), 7.75 (d, *J* = 7.8 Hz, 1H), 7.58 – 7.51 (m, 2H), 7.49 – 7.40 (m, 5H), 7.40 – 7.27 (m, 5H), 7.27 – 7.17 (m, 1H). **^{13}C NMR** (101 MHz, Chloroform-*d*) δ 136.0, 135.1, 134.1, 132.7, 130.2, 128.8, 128.7, 128.6, 128.2, 127.8, 126.3, 122.8, 120.5, 119.8, 115.1, 110.4. **HRMS** calculated for $C_{20}H_{16}N$ $[M+H]^+$ 270.1278, found 270.1276. The data are in agreement with those previously reported in the literature.[31]

6.2.4 Synthesis of isoquinolinone

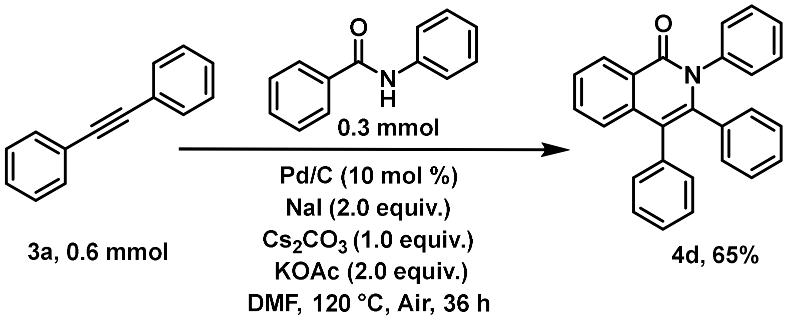

A Schlenk tube (25.0 mL) equipped with a magnetic stir bar was charged with benzamide (0.3 mmol, 59.1 mg), **3a** (0.6 mmol, 106.8 mg), 10 mol % Pd/C (0.03 mmol, 31.8 mg), NaI (0.6 mmol, 111.6 mg), Cs_2CO_3 (0.3 mmol, 97.8 mg) and KOAc (0.6 mmol, 57.6 mg) and DMF (1.0 mL) . The reaction was then heated to 120 °C and maintained under air for 36 hours. The mixture was filtered and washed with H_2O and extracted with CH_2Cl_2. The combined organic phase was dried with anhydrous Na_2SO_4. After removal of solvents under reduced pressure, the residue was absorbed to small amounts of silica. The purification **4d** was performed by flash column chromatography on silica gel.

2,3,4-triphenylisoquinolin-1(2H)-one (4d)

White solid, 0.19 mmol, 70.9 mg, 65%, eluting with 20% ethyl acetate: petroleum ether. **^{1}H NMR** (400 MHz, Chloroform-*d*) δ 8.61 (d, *J* = 9.6 Hz, 1H), 7.67 – 7.52 (m, 2H), 7.33 – 7.12 (m, 11H), 6.93 (s, 5H). **^{13}C NMR** (101 MHz, Chloroform-*d*) δ 162.7, 141.1, 139.5, 137.7, 136.4, 134.8,

132.6, 131.7, 131.1, 129.6, 128.6, 128.3, 128.0, 127.6, 127.3, 127.1, 126.9, 126.9, 125.6, 125.5, 118.8. **HRMS** calculated for $C_{27}H_{20}NO$ $[M+H]^+$ 374.1540, found 374.1540. The data are in agreement with those previously reported in the literature.[32]

7. Mechanism experiments

7.1 Verifying the possibility of radical pathway

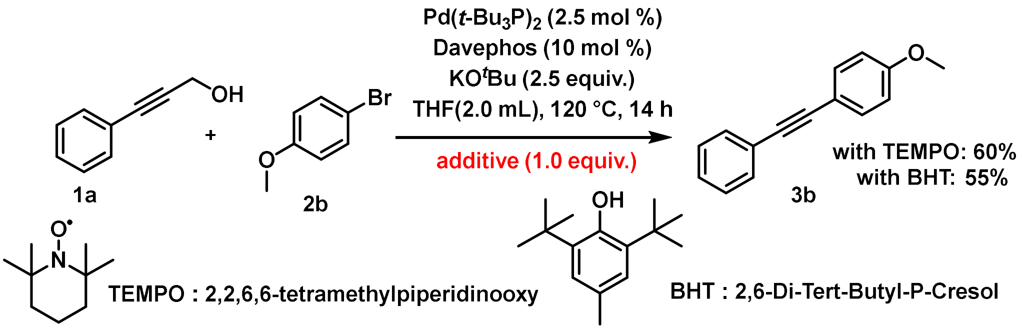

A Schlenk tube (25.0 mL) with a magnetic stir bar was charged with **1a** (0.3 mmol, 1.5 equiv.), **2b** (0.2 mmol, 1.0 equiv.) , Pd(t-Bu_3P)$_2$ (0.005 mmol, 2.5 mol %, 2.5 mg), Davephos (0.02 mmol, 10 mol %, 7.8 mg) , KO^{t}Bu (0.5 mmol, 2.5 equiv, 56.0 mg) and THF (2.0 mL). Then BHT (0.2 mmol, 1.0 equiv, 44.0 mg) or TEMPO (0.2 mmol, 1.0 equiv, 31.2 mg) was added to the reaction. The Schlenk tube was then sealed with a Teflon lined cap and then heated at 120 °C for 14 hours. The reaction solution was then cooled to ambient temperature and EA (3.0 mL) was added. After filtration and evaporation of the solvents under reduced pressure, the residue was purified by column chromatography on silica gel (100% petroleum ether) to provide the desired product **3b** in 24.9 mg (60% yield) and 22.8 mg (55% yield) respectively.

7.2 Control experiment

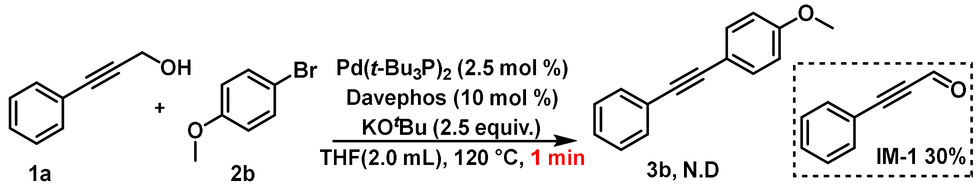

A Schlenk tube (25.0 mL) with a magnetic stir bar was charged with **1a** (0.3 mmol, 1.5 equiv.), **2b** (0.2 mmol, 1.0 equiv.), Pd(t-Bu_3P)$_2$ (0.005 mmol, 2.5 mol %, 2.5 mg), Davephos (0.02 mmol, 10 mol %, 7.8 mg), KO^{t}Bu (0.5 mmol, 2.5 equiv, 56.0 mg) and THF (2.0 mL). The Schlenk tube was then sealed with a Teflon lined cap and then heated at 120 °C for 1min. After the indicated time, the reaction solution was cooled to ambient temperature and EA (3.0 mL) was added. After filtration and evaporation of the solvents under reduced pressure, the residue was purified by column chromatography on silica gel (10% ethyl acetate: petroleum ether) to provide the desired product **IM-1** (0.06 mmol, 7.8 mg, 30% yield)。Subsequently, we characterized the results using NMR and HRMS.

3-phenylpropiolaldehyde(IM-1)

Pale yellow liquid, 0.06 mmol, 7.8 mg, 30%, eluting with 10% ethyl acetate: petroleum ether.
^{1}**H NMR** (400 MHz, Chloroform-d) δ 9.42 (s, 1H), 7.64 – 7.57 (m, 2H), 7.53 – 7.44 (m, 1H), 7.44

– 7.36 (m, 2H).[13]**C NMR** (101 MHz, Chloroform-*d*) δ 176.8, 133.3, 131.3, 128.7, 119.4, 95.1, 88.4. **HRMS** calculated for C_9H_7O $[M+H]^+$ 131.0492, found 131.0491. The data are in agreement with those previously reported in the literature.[33]

7.3 Reaction monitoring experiment

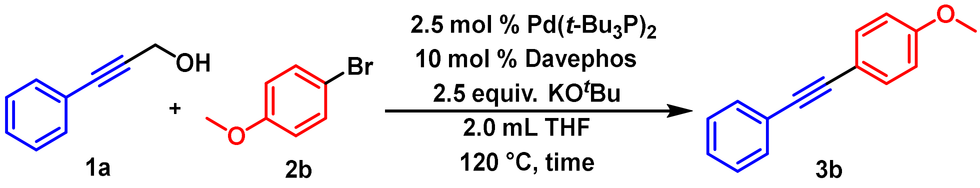

Time (h)	IM-1 (%)	3b (%)
0.017	30	0
2	5	8
4	0	22
8	0	45
14	0	72

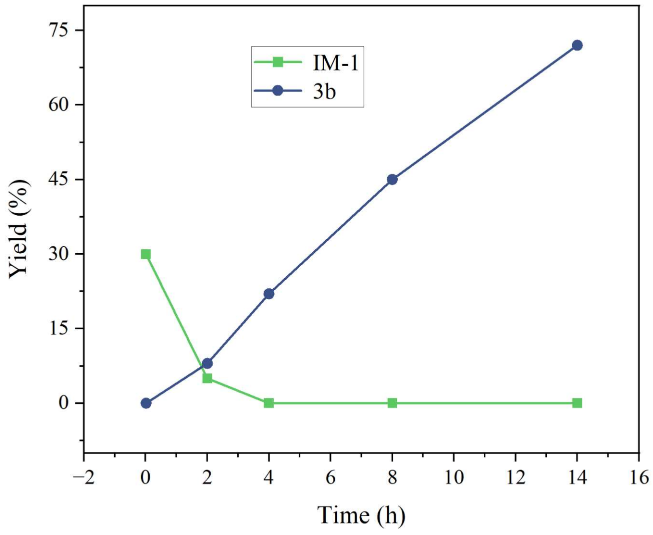

A Schlenk tube (25.0 mL) with a magnetic stir bar was charged with **1a** (0.3 mmol, 1.5 equiv.), **2b** (0.2 mmol, 1.0 equiv.), Pd(*t*-$Bu_3P)_2$ (0.005 mmol, 2.5 mol %, 2.5 mg), Davephos (0.02 mmol, 10 mol %, 7.8 mg), KO*^t^*Bu (0.5 mmol, 2.5 equiv, 56.0 mg) and THF (2.0 mL). The Schlenk tube was then sealed with a Teflon lined cap and then heated at 120 °C, We selected five different time intervals (0.017h, 2h, 4h, 8h, 14h) to monitor the reaction, observing the changes of **IM-1** and **3b** over time.

7.4 Intermediate experiment

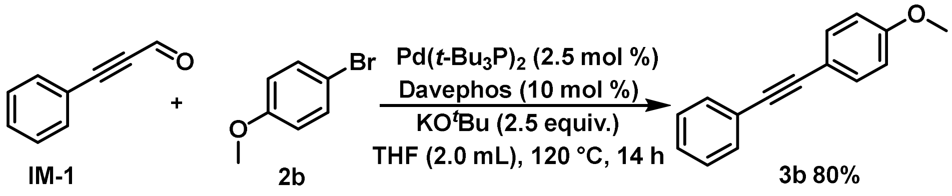

A Schlenk tube (25.0 mL) with a magnetic stir bar was charged with **IM-1** (0.3 mmol, 1.5 equiv.), **2b** (0.2 mmol, 1.0 equiv.), Pd(*t*-$Bu_3P)_2$ (0.005 mmol, 2.5 mol %, 2.5 mg), Davephos

(0.02 mmol, 10 mol %, 7.8 mg) , KO^{t}Bu (0.5 mmol, 2.5 equiv, 56.0 mg) and THF (2.0 mL). The Schlenk tube was then sealed with a Teflon lined cap and then heated at 120 °C for 14 hours. After the indicated time, the reaction solution was cooled to ambient temperature and EA (3.0 mL) was added. After filtration and evaporation of the solvents under reduced pressure, the residue was purified by column chromatography on silica gel (100% petroleum ether) to obtain product **3b** in 80% yield. Considering that we successfully detected substance **IM-1** in the control experiment, it is sufficient to conclude that **IM-1** is indeed the intermediate of the reaction.

8. References

1. T. Sang, J. Liang, S. Guo, J. Yang, X. Bao and C. Huo, *J. Org. Chem.,* 2023, **88**, 10232–10241.
2. M. Wang, S. Jiang, X. Lu, K. Zhang, Z. Yuan, R. Xu, B. Zhao and A. Wu, *Org. Biomol. Chem.,* 2023, **21**, 5949–5952.
3. C. M. Le, P. J. C. Menzies, D. A. Petrone and M. Lautens, *Angew. Chem. Int. Ed*., 2015, **127**, 256–259.
4. M. Klikar, I. V. Kityk, D. Kulwas, T. Mikysek, O. Pytela and F. Bureš, *New J. Chem.*, 2017, **41**, 1459–1472.
5. O. Koniev, S. Kolodych, Z. Baatarkhuu, J. Stojko, J. Eberova, J. Bonnefoy, S. Cianferani, A. V. Dorsselaer and A. Wagner, *Bioconjugate Chem.,* 2015, **26**, 1863–1867.
6. N. Chaisan, S. Ruengsangtongkul, C. Thongsornkleeb, J. Tummatorn and S. Ruchirawat, *Synlett.*, 2022, **33**, 1426–1430.
7. Q. Zhang, Y. Duan, H. Guo, H. Yang, J. Zhai, T. Li, Z. Wang, X. Lu, Y. Wang and Y. Yin, *Chem. Asian. J*., 2021, **16**, 1832–1838.
8. Z. Wang, Y. Sun, Q. Zhang, W. Pan, T. Li and Y. Yin, *J. Org. Chem.,* 2022, **87**, 3329–3340.
9. C. Chen, Y. Huang, Z. Zhang, X. Dong and X. Zhang, *Chem. Commun*., 2017, **53**, 4612–4615.
10. G. Sullivan, Y. Zhang, G. Xu, L. Christianson, F. Luengo, T. Halkoski and P. Gao, *Green Chem.,* 2022, **24**, 7184–7193.
11. S. Kumari, A. K. Dhara, A. Ratnam, K. Mawai, V. K. Chaudhary, A. Mohanty and K. Ghosh, *J. Org. Chem.,* 2020, **928**, 121367.
12. Y. Chen, S. Li, L. Xu and D. Ma, *J. Org. Chem.,* 2023, **88**, 3330–3334.
13. S. Sil, A. U. Krishnapriya, P. Mandal, R. Kuniyil and S. K. Mandal, *Chem. Eur. J.,* 2024, **30**, e202400895.
14. M. Ye, M. Hou, Y. Wang, X. Ma, K. Yang and Q. Song, *Org. Lett.,* 2023, **25**, 1787–1792.
15. T. Truong and O. Daugulis, *Org. Lett.,* 2011, **13**, 4172–4175.
16. Y. Chen, S. Li, L. Xu and D. Ma, *J. Org. Chem.,* 2023, **88**, 3330–3334.
17. T. Haro, C. Nevado, *J. Am. Chem. Soc.,* 2010, **132**, 1512–1513.
18. Y. Ji, N. Zhong, Z. Kang, G. Yan and M. Zhao, *Synlett.,* 2018, **29,** 209–214.
19. L. Liang, H. Niu, R. Li, Y. Wang, J. Yan, C. Li and H. Guo, *Org. Lett.,* 2020, **22**, 6842–6846.
20. A. Tlahuext-Aca, M. N. Hopkinson, B. Sahoo and F. Glorius, *Chem. Sci.,* 2016, **7**, 89.
21. P. K. Mandali, D. K. Chand, *Cat Commun.,* 2014, **47**, 40–44.
22. M. Liu, M. Ye, Y. Xue, G. Yin, D. Wang and J. Huang, *Tetrahedron Letters.,* 2016, **57**, 3137–3139.
23. C. Rossy, J. Majimel, E. Fouquet, C. Delacote, M. Boujtita, C. Labrugere, M. Treguer-

Delapierre, F. X. Felpin, *Chem. Eur. J.,* 2013, **19**, 14024–14029.

24. A. F. Palermo, B. S. Y. Chiu, P. Patel and S. A. L. Rousseaux, *J. Am. Chem. Soc.,* 2023, **145**, 24981–24989.
25. J. Cao, W. Ding and G. Zou, *Org. Lett.,* 2024, **26**, 4576–4580.
26. M. Chourasiya, A. Kumar, V. N. Vikram and N. Tadigopila, *Chem. Commun.,* 2023, **59***,* 9650–9653.
27. Z. Tian, S. Wang, S. Jia, H. Song and C. Zhang, *Org. Lett.,* 2017, **19**, 5454–5457.
28. J. Liang, L. Yang, S. Wu, S. Liu, M. Cushman, J. Tian, N. Li, Q. Yang, H. Zhang, Y. Qiu, L. Xiang, C. Ma, X. Li and H. Qing, *Eur. J. Med. Chem.,* 2017, **136**, 382–392.
29. L. Huang, A. Biafora, G. Zhang, V. Bragoni, L. J. Gooβen, *Angew. Chem. Int. Ed.,* 2016, **55**, 6933–6937.
30. C. Chen, W. Hu, M. Liu, P. Yan, J. Wang, M. Chung, *Tetrahedron.,* 2013, **69**, 9735–9741.
31. M. Shen, G. Li, B. Z. Lu, A. Hossain, F. Roschangar, V. Farina and C. H. Senanayake, *Org. Lett.,* 2004, **6**, 4129–4132.
32. Z. Shu, Y. Guo, W. Li, B. Wang, *Catalysis Today.,* 2017, **297**, 292–297.
33. N. Brach, L. Popek, M. Truong, C. Laurent, V. Bizet, K. P. Kaliappan and N. Blanchard, *Org. Lett.,* 2023, **25**, 7847–7851.

9. Copies of ^{1}H、^{13}C and ^{19}F spectra

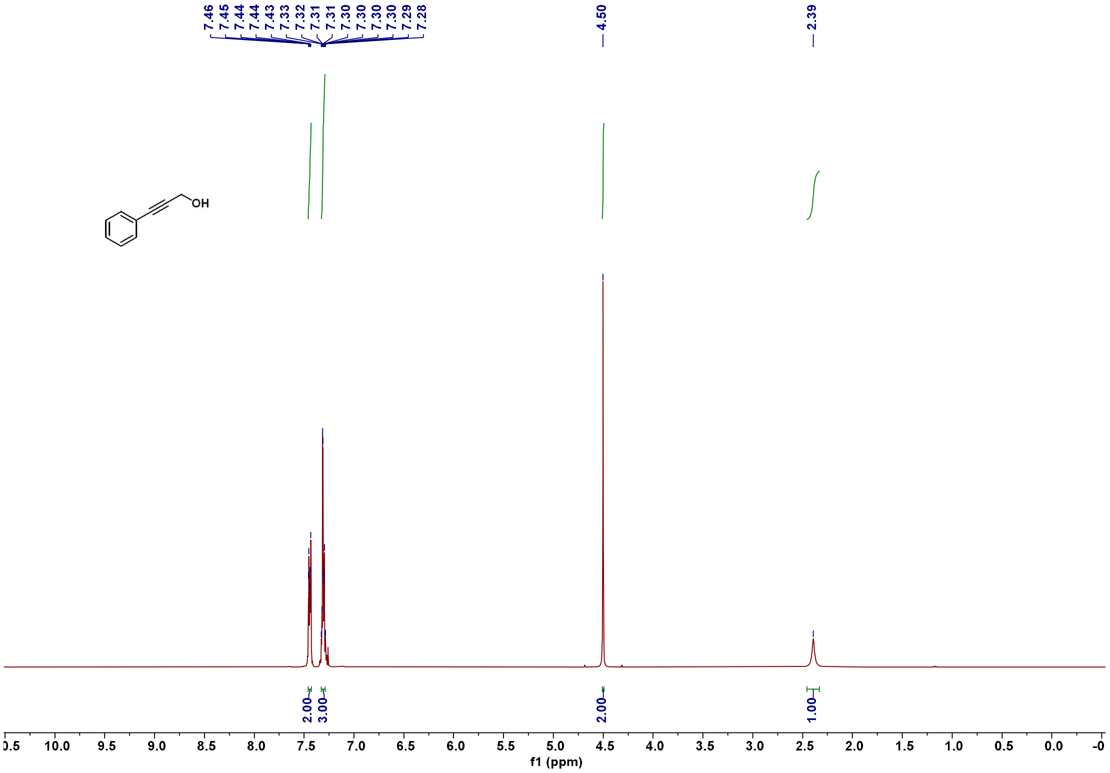

^{1}H NMR (400 MHz, $CDCl_3$) spectrum of 1a

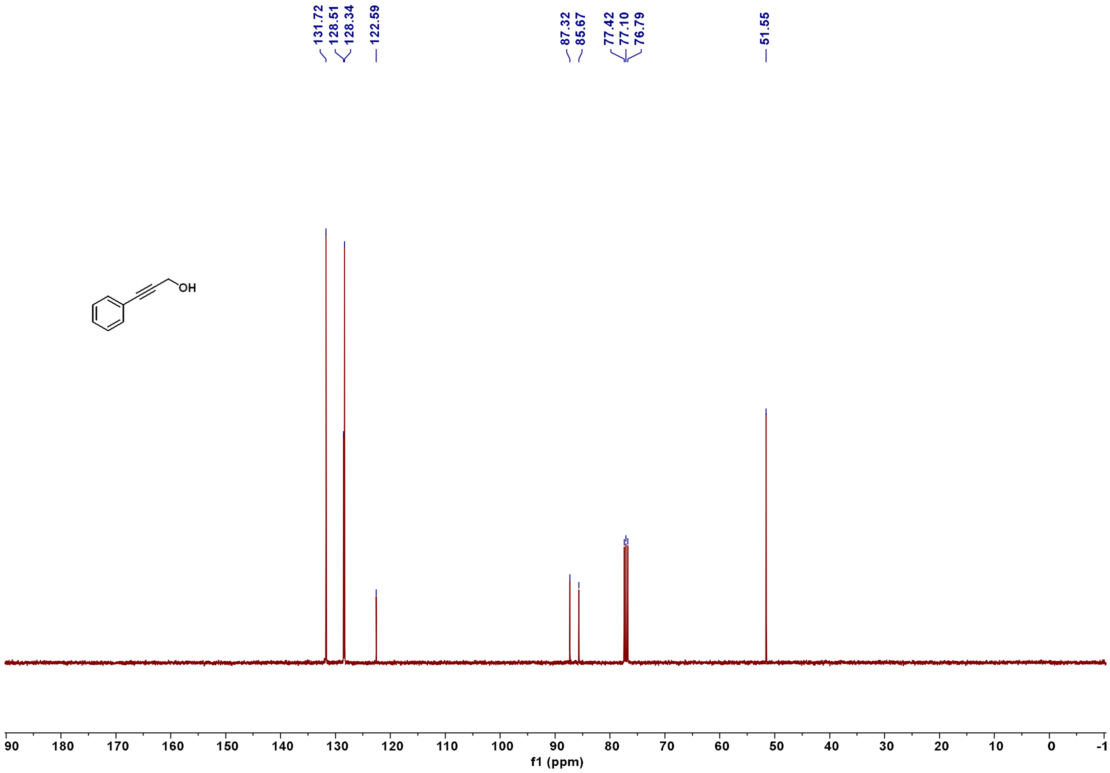

^{13}C NMR (101 MHz, $CDCl_3$) spectrum of 1a

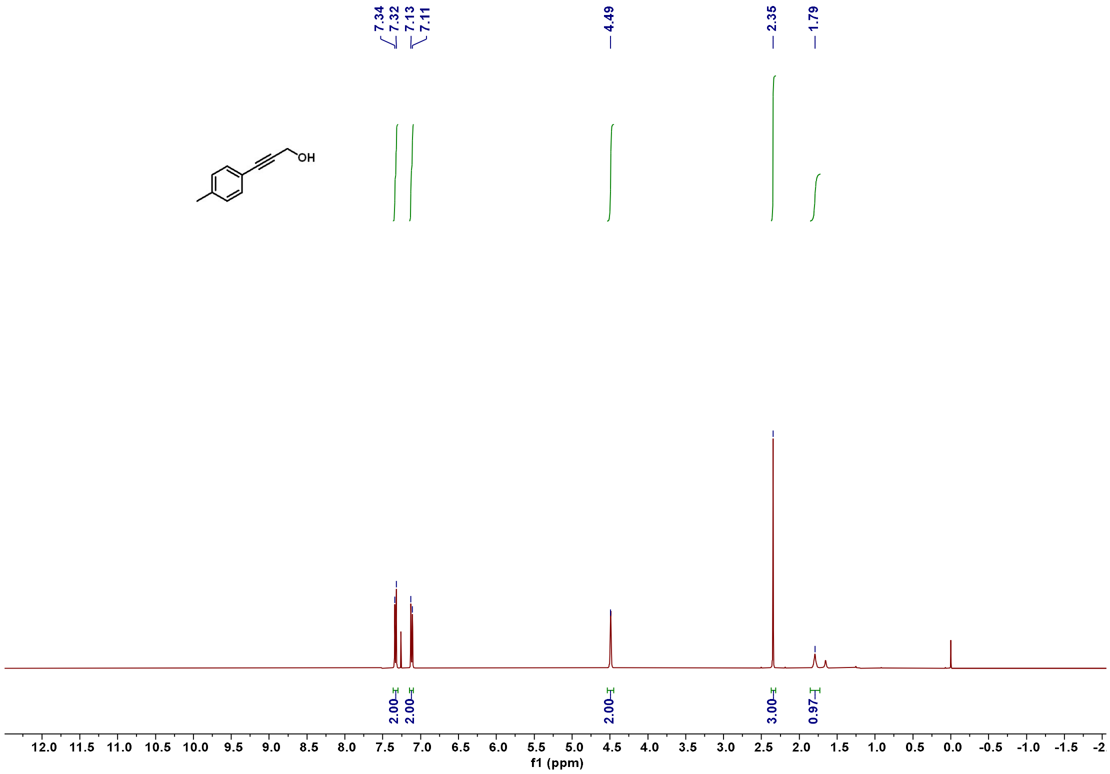

^{1}H NMR (400 MHz, CDCl$_3$) spectrum of 1b

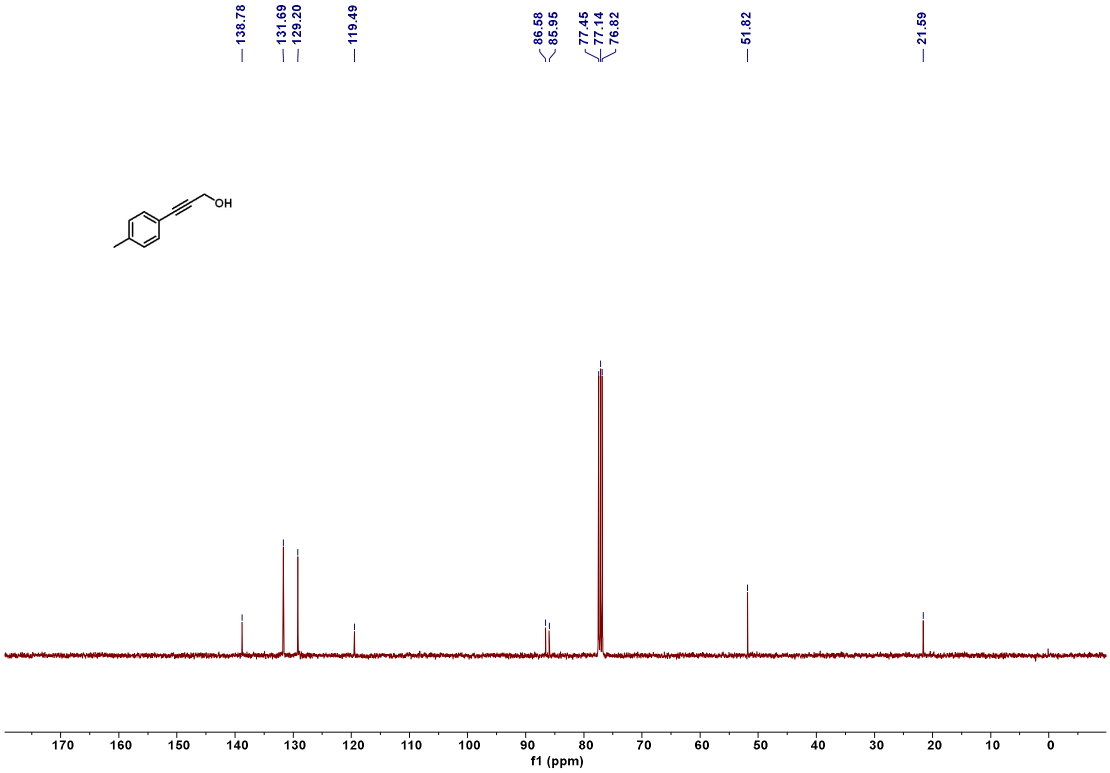

^{13}C NMR (101 MHz, CDCl$_3$) spectrum of 1b

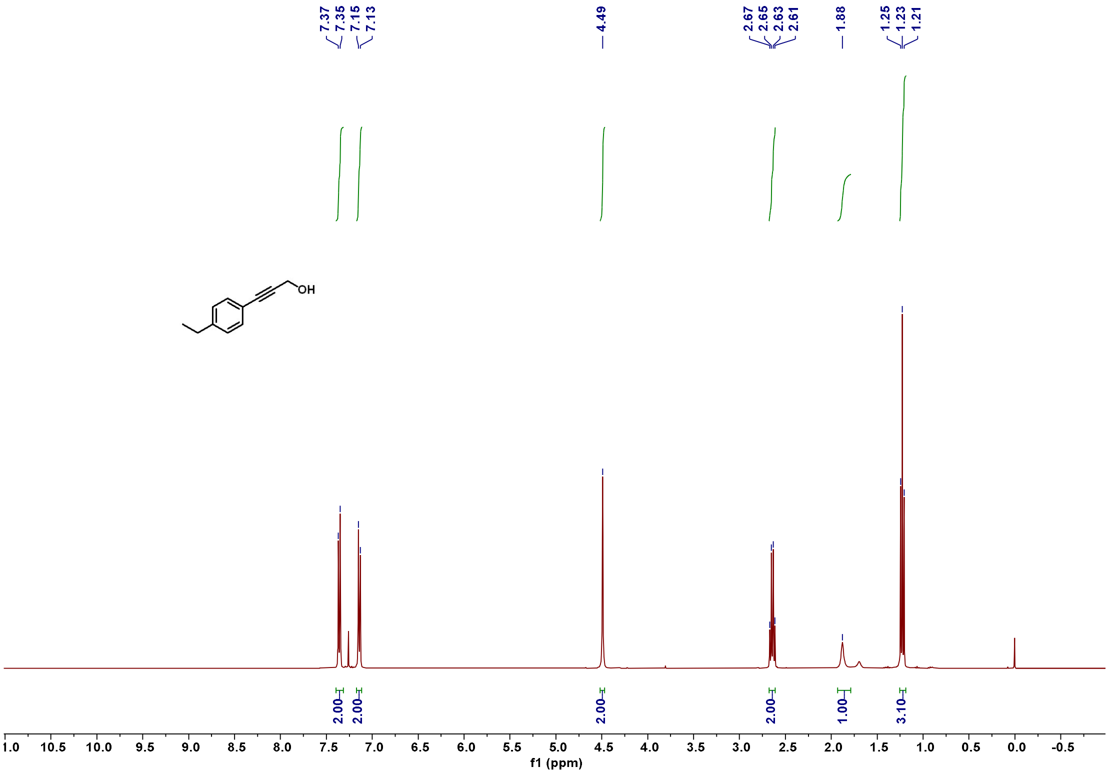

^{1}H NMR (400 MHz, $CDCl_3$) spectrum of 1c

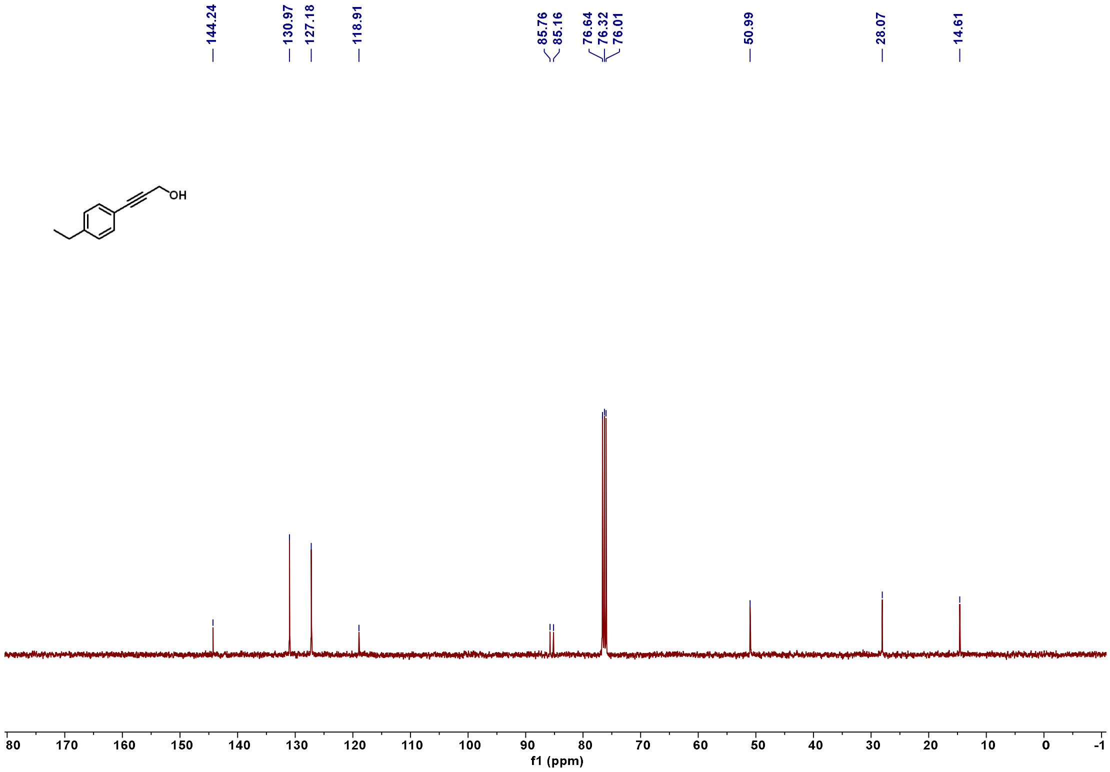

^{13}C NMR (101 MHz, $CDCl_3$) spectrum of 1c

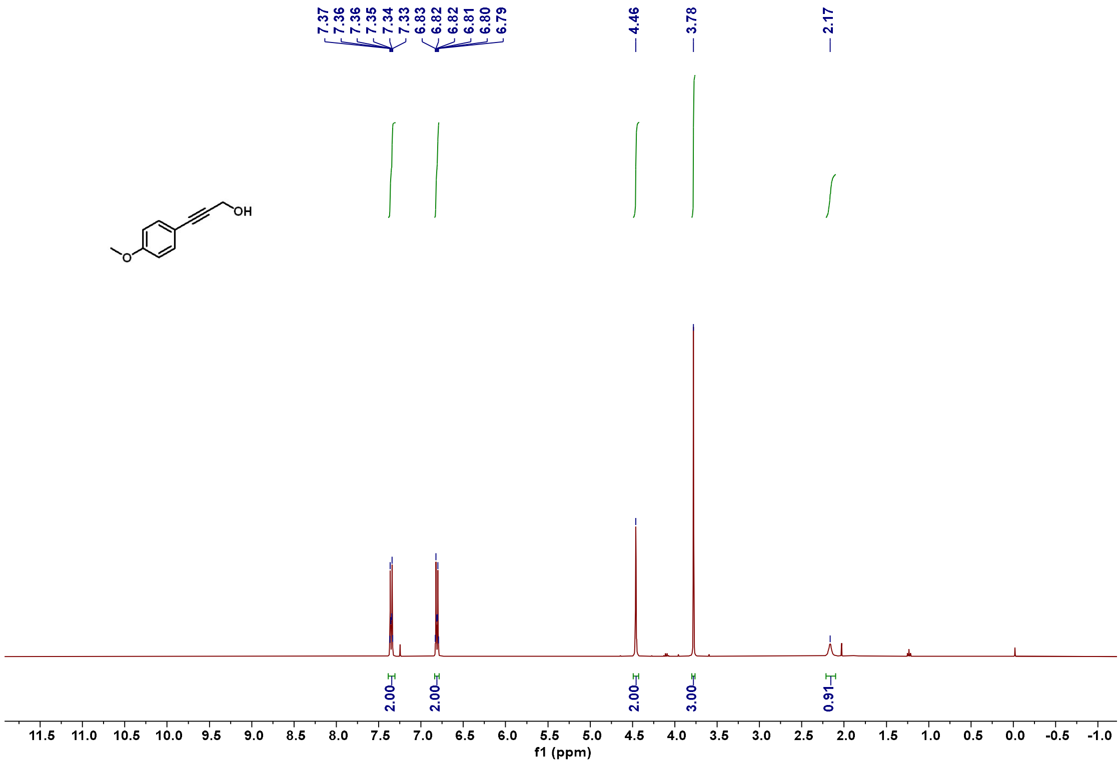

^{1}H NMR (400 MHz, CDCl$_3$) spectrum of 1d

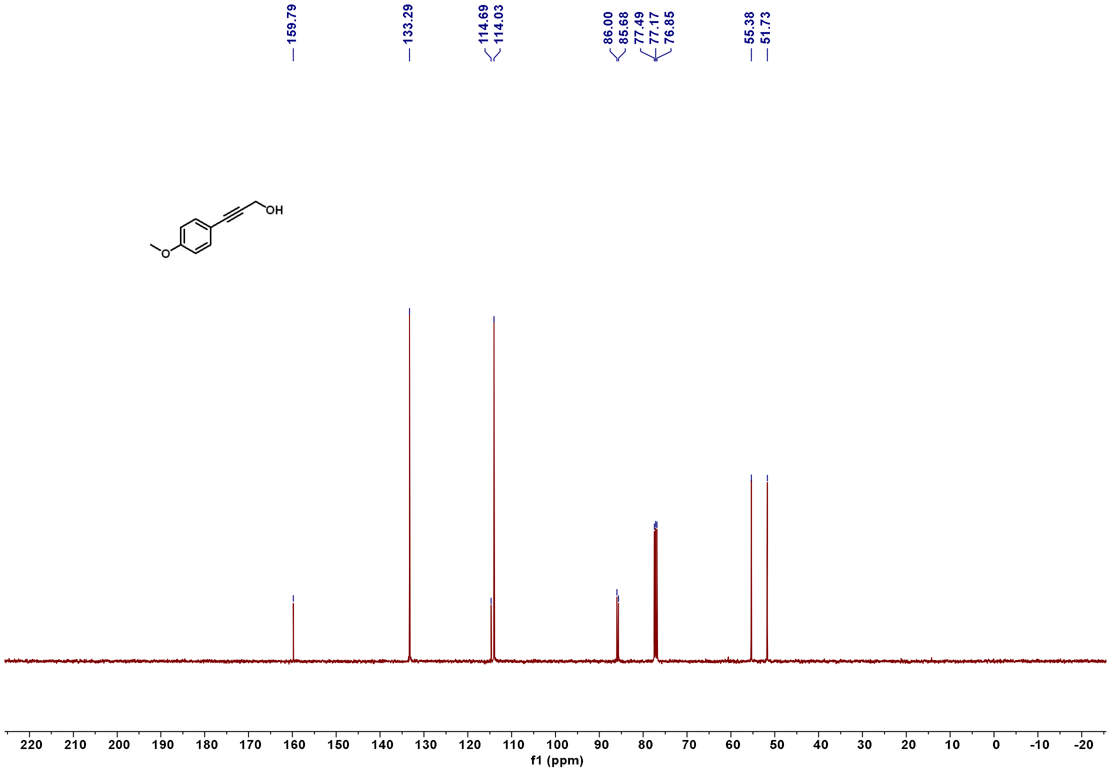

^{13}C NMR (101 MHz, CDCl$_3$) spectrum of 1d

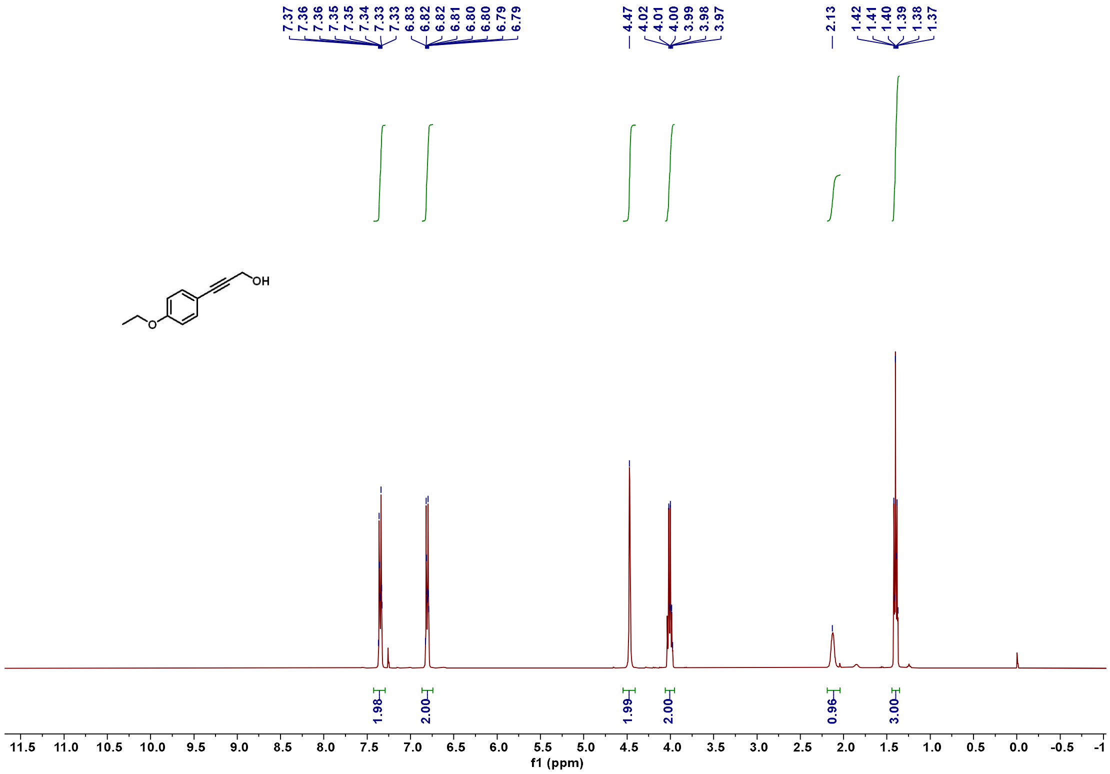

^{1}H NMR (400 MHz, $CDCl_3$) spectrum of 1e

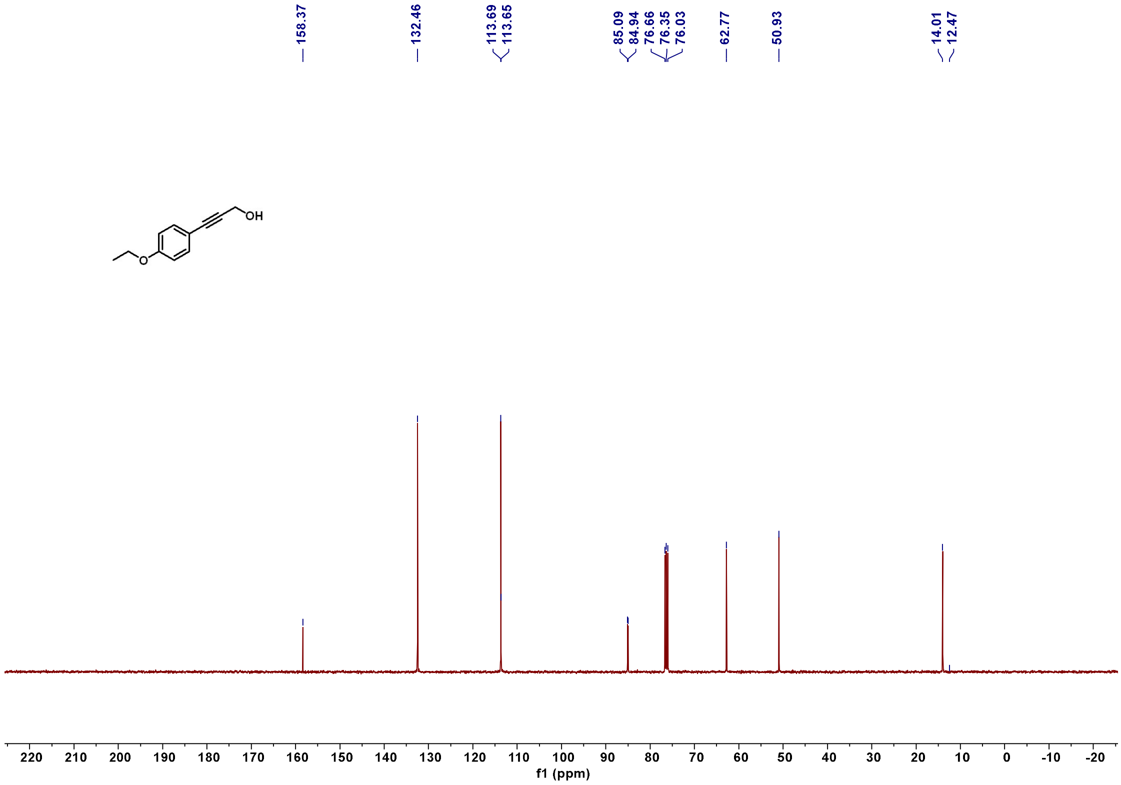

^{13}C NMR (101 MHz, $CDCl_3$) spectrum of 1e

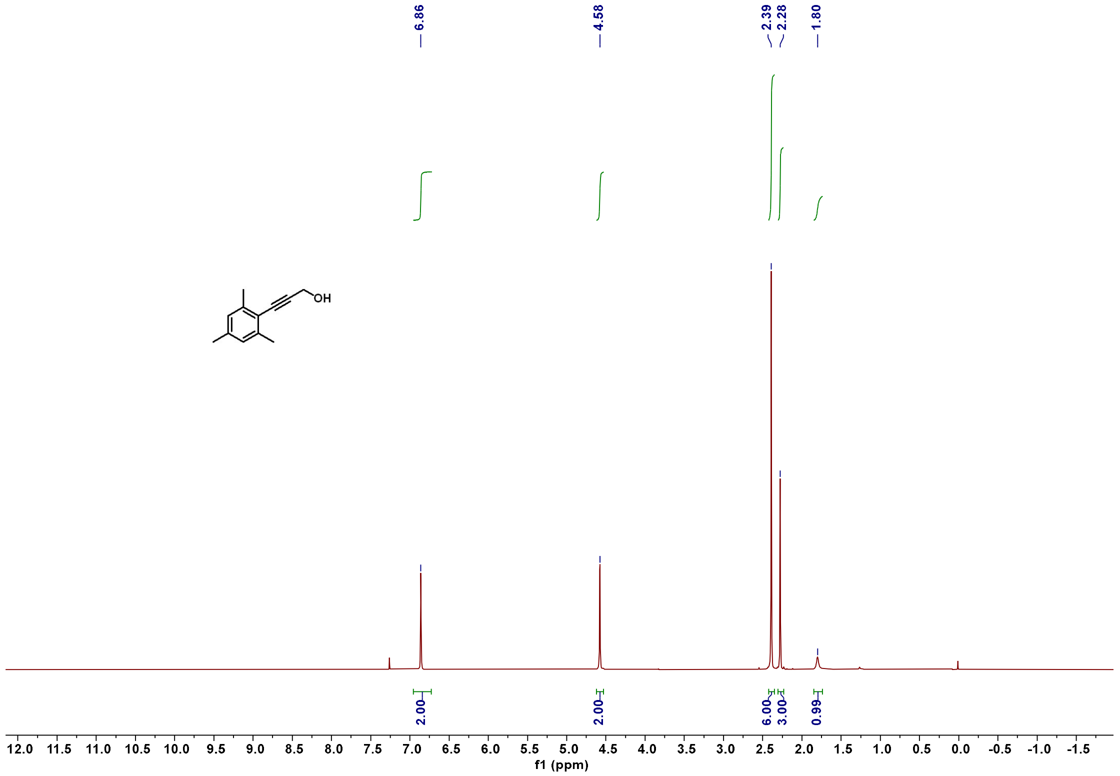

^{1}H NMR (400 MHz, $CDCl_3$) spectrum of 1f

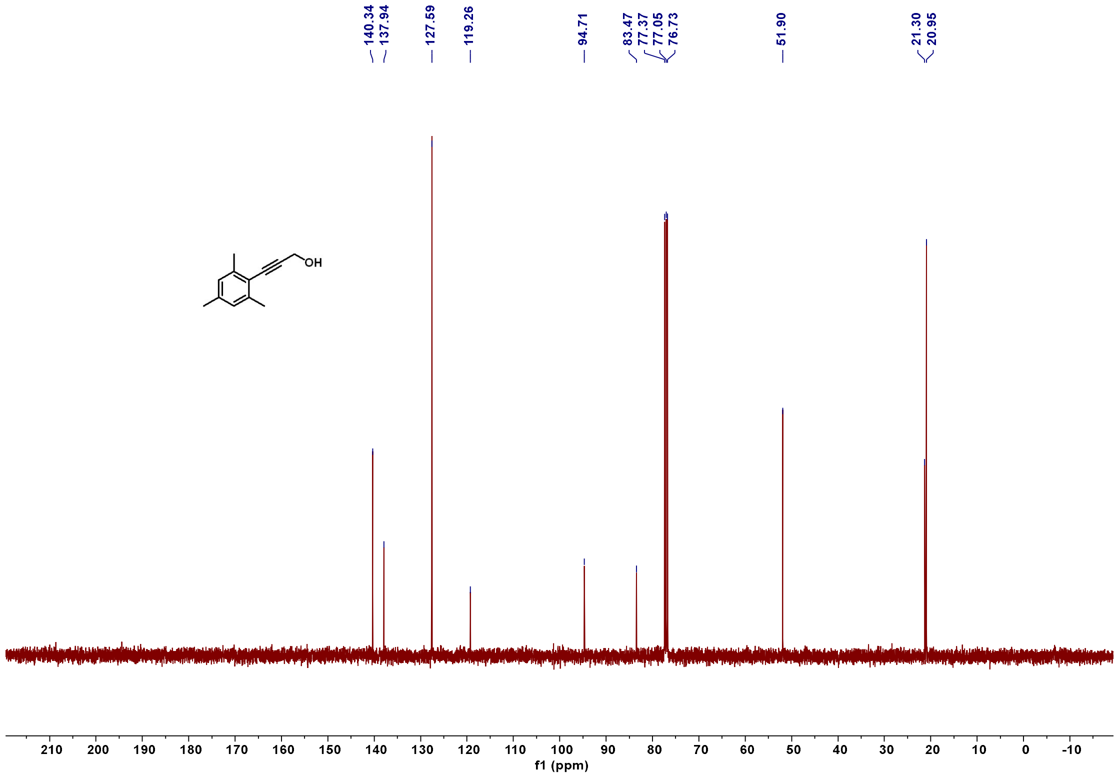

^{13}C NMR (101 MHz, $CDCl_3$) spectrum of 1f

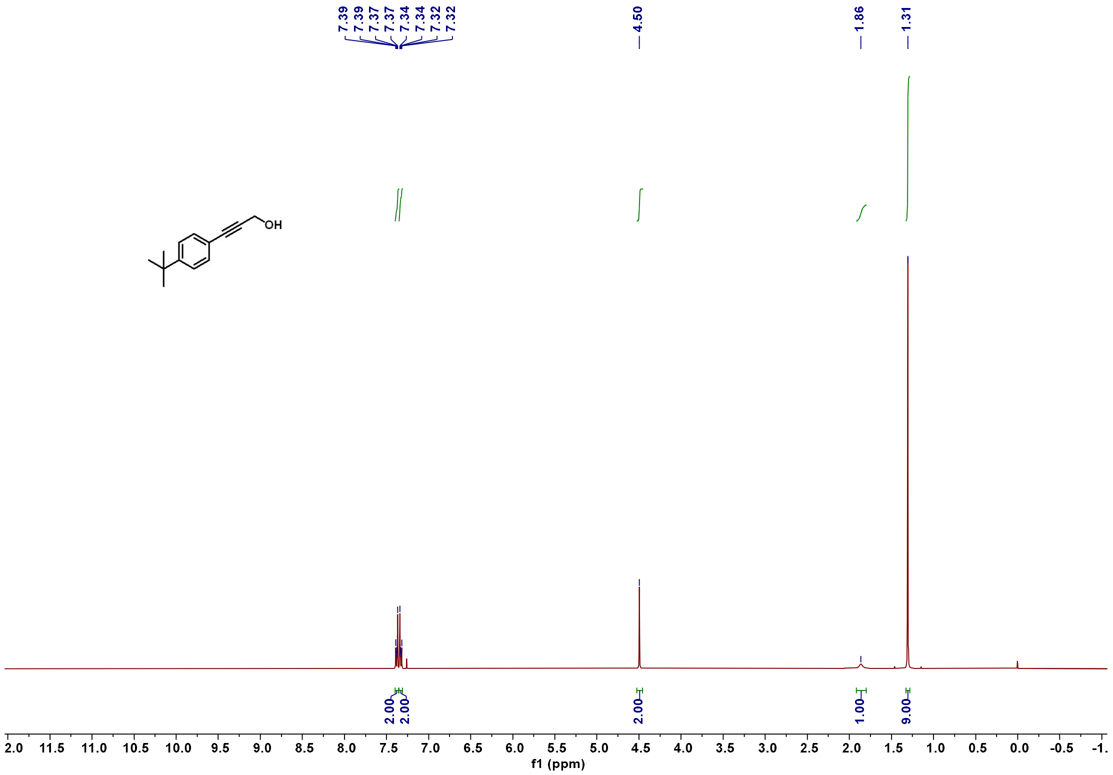

^{1}H NMR (400 MHz, $CDCl_3$) spectrum of 1g

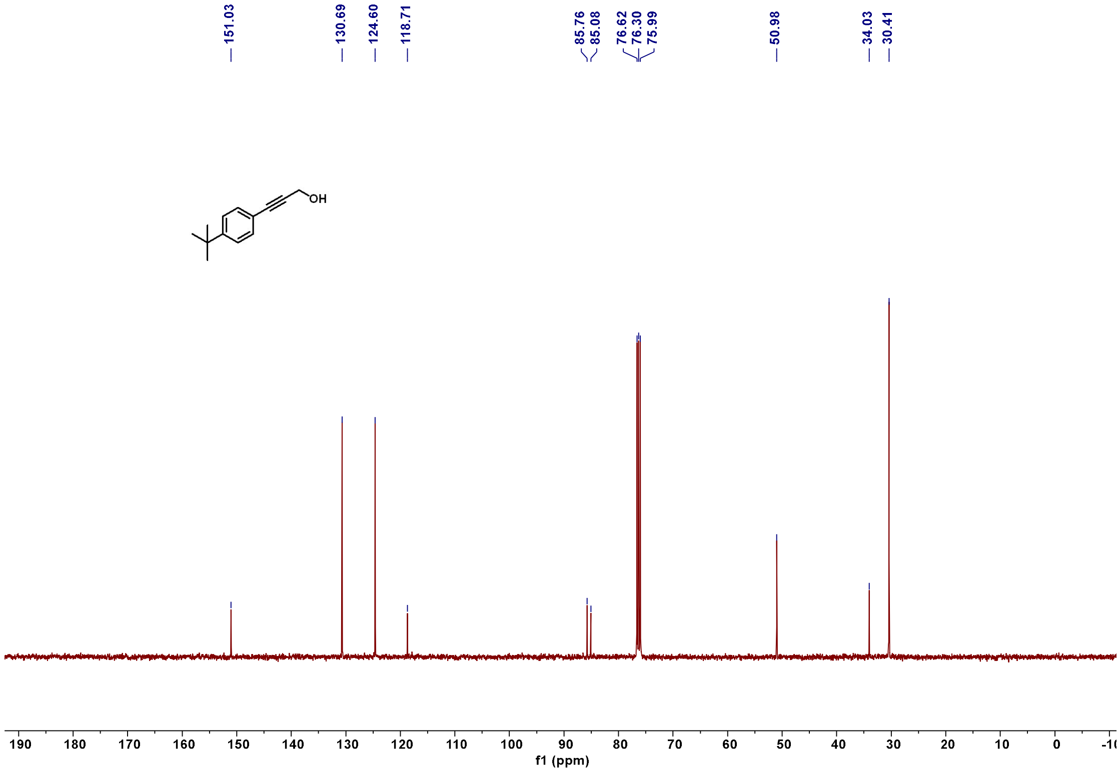

^{13}C NMR (101 MHz, $CDCl_3$) spectrum of 1g

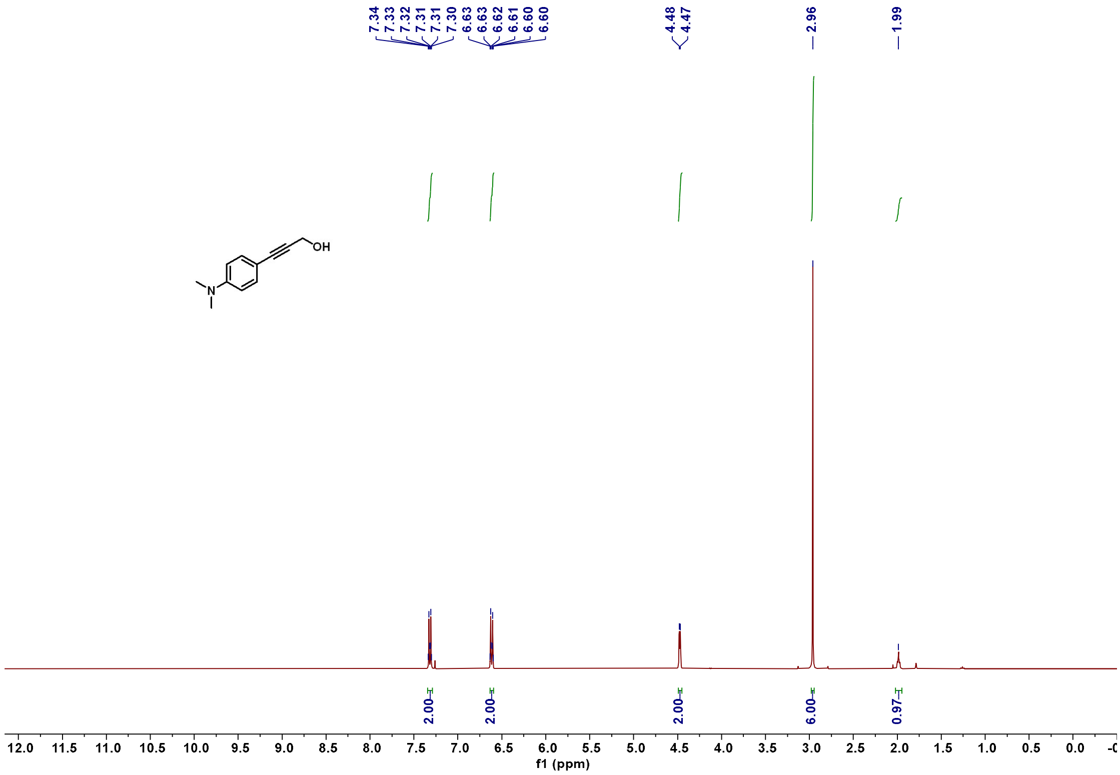

^{1}H NMR (400 MHz, $CDCl_3$) spectrum of 1h

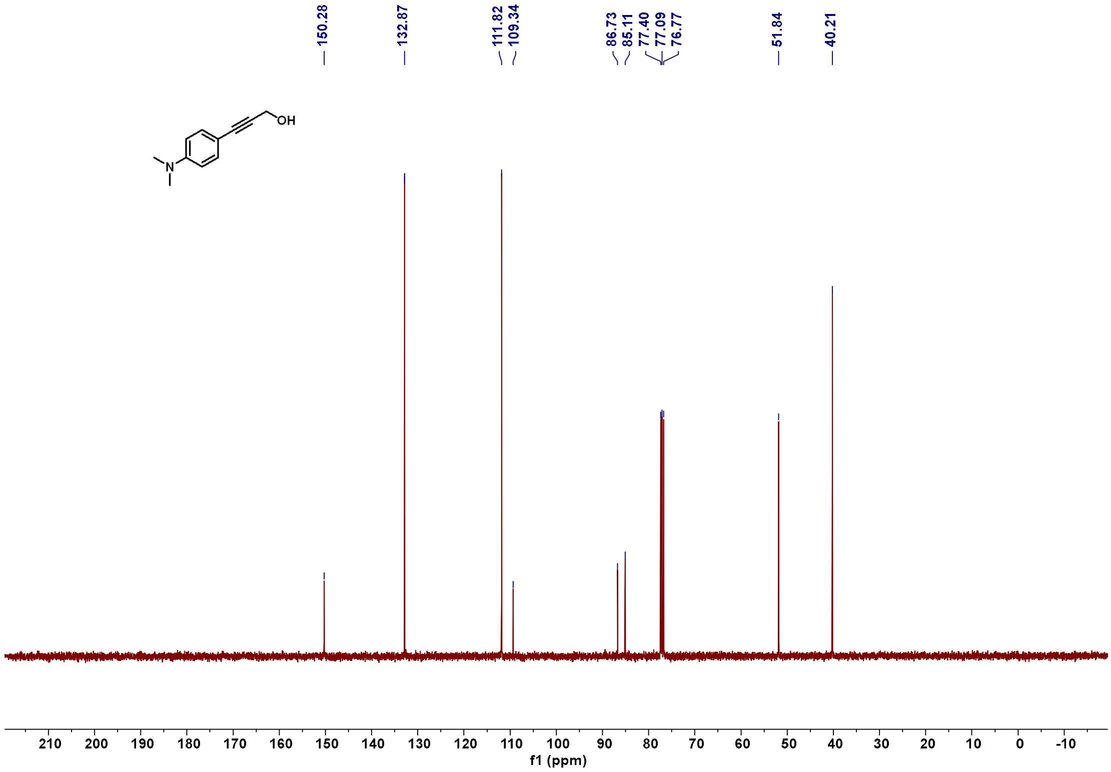

^{13}C NMR (101 MHz, $CDCl_3$) spectrum of 1h

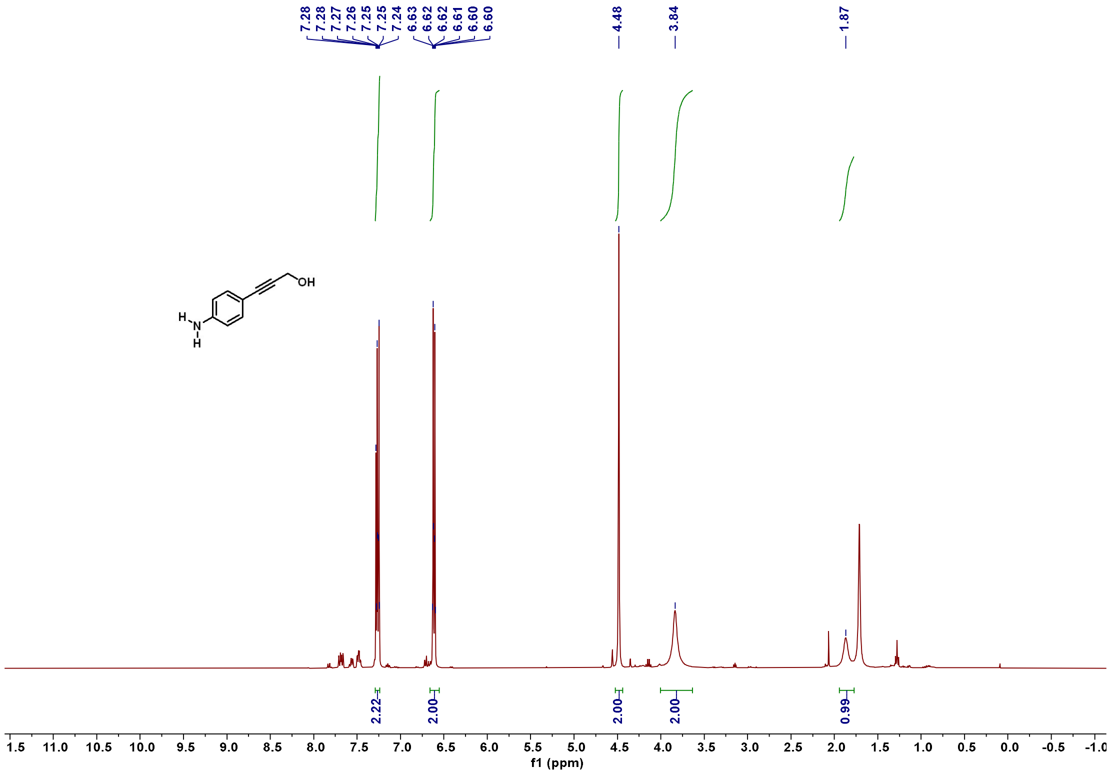

^{1}H NMR (400 MHz, $CDCl_3$) spectrum of 1i

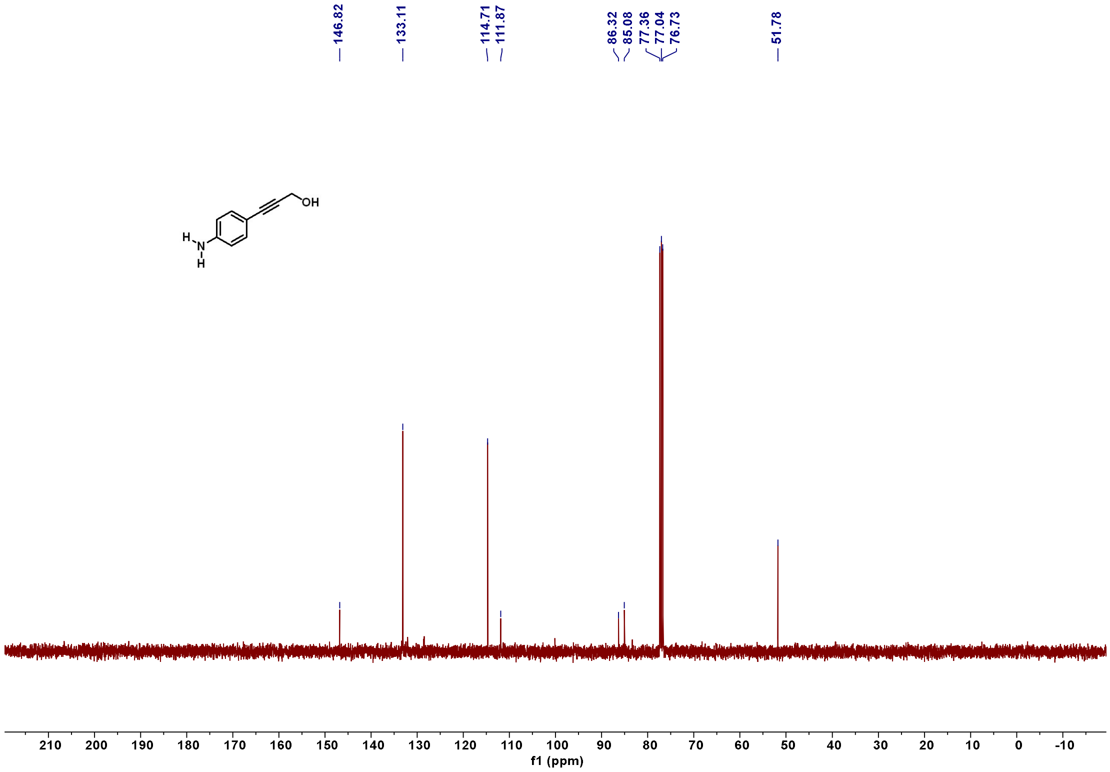

^{13}C NMR (101 MHz, $CDCl_3$) spectrum of 1i

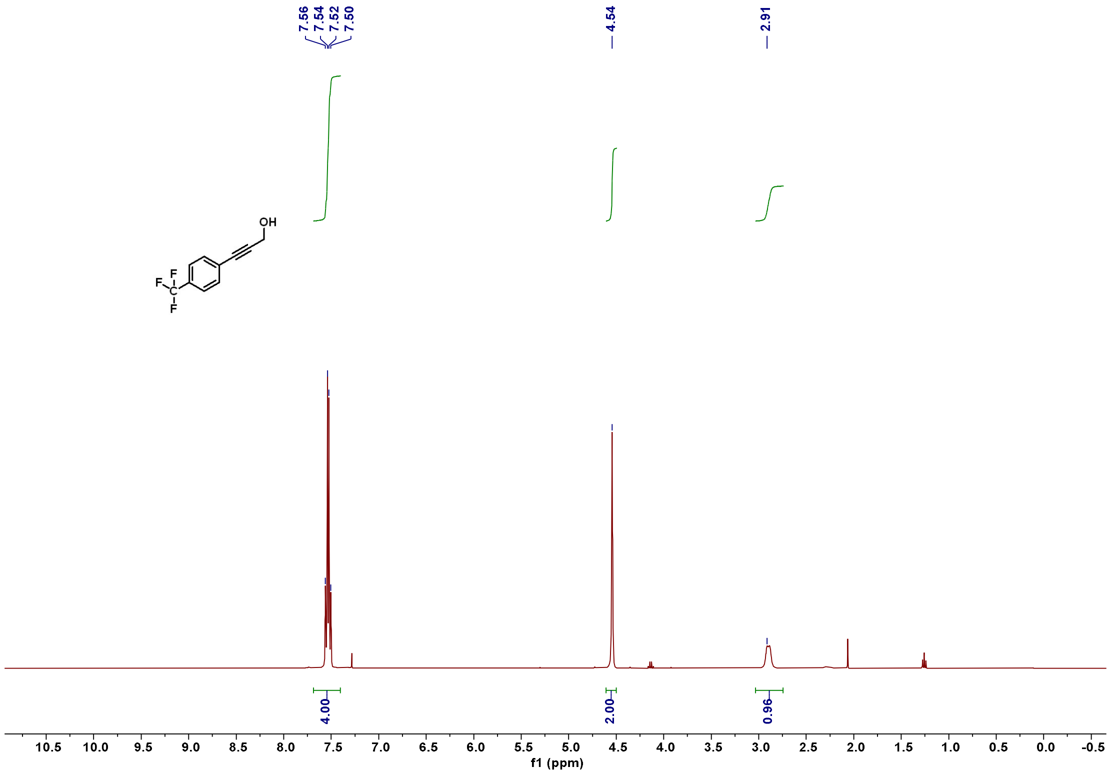

^{1}H NMR (400 MHz, $CDCl_3$) spectrum of 1j

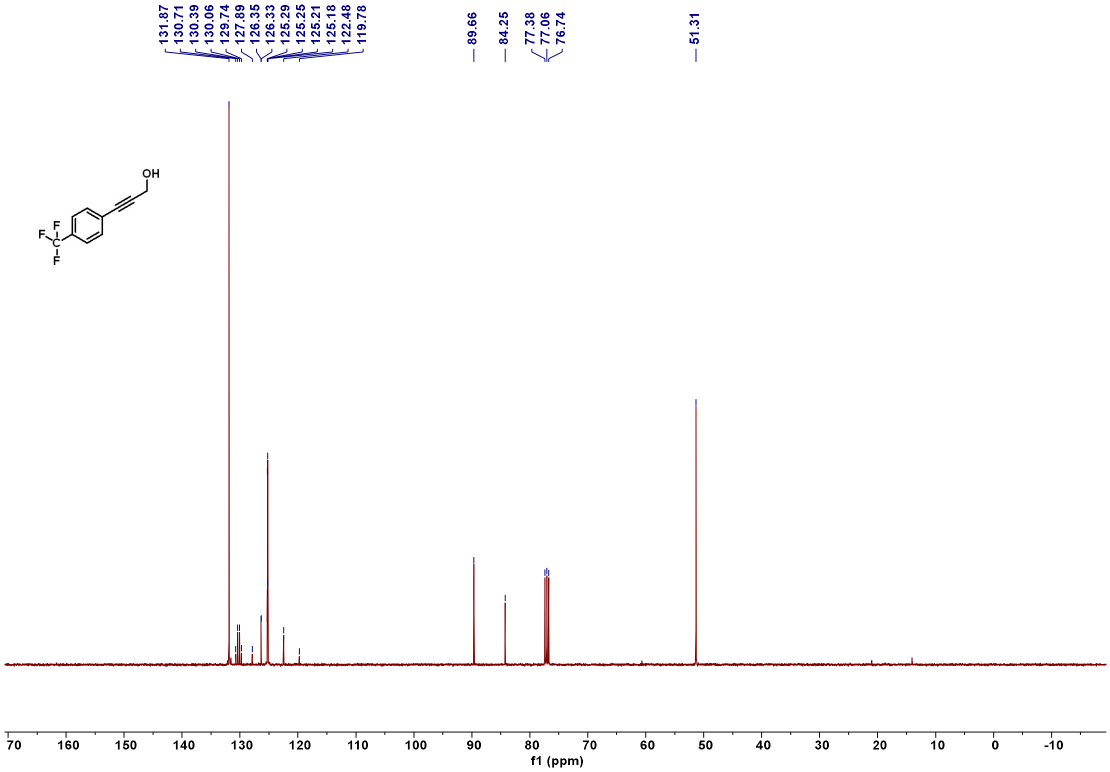

^{13}C NMR (101 MHz, $CDCl_3$) spectrum of 1j

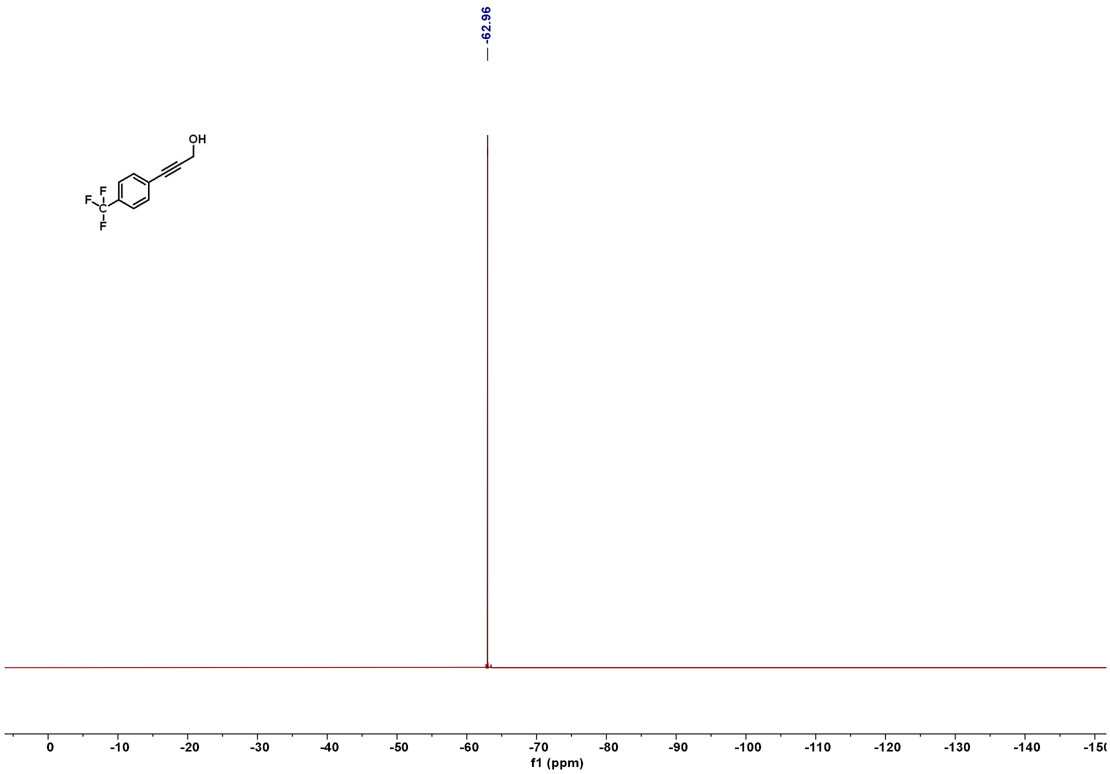

^{19}F NMR (376 MHz, $CDCl_3$) spectrum of 1j

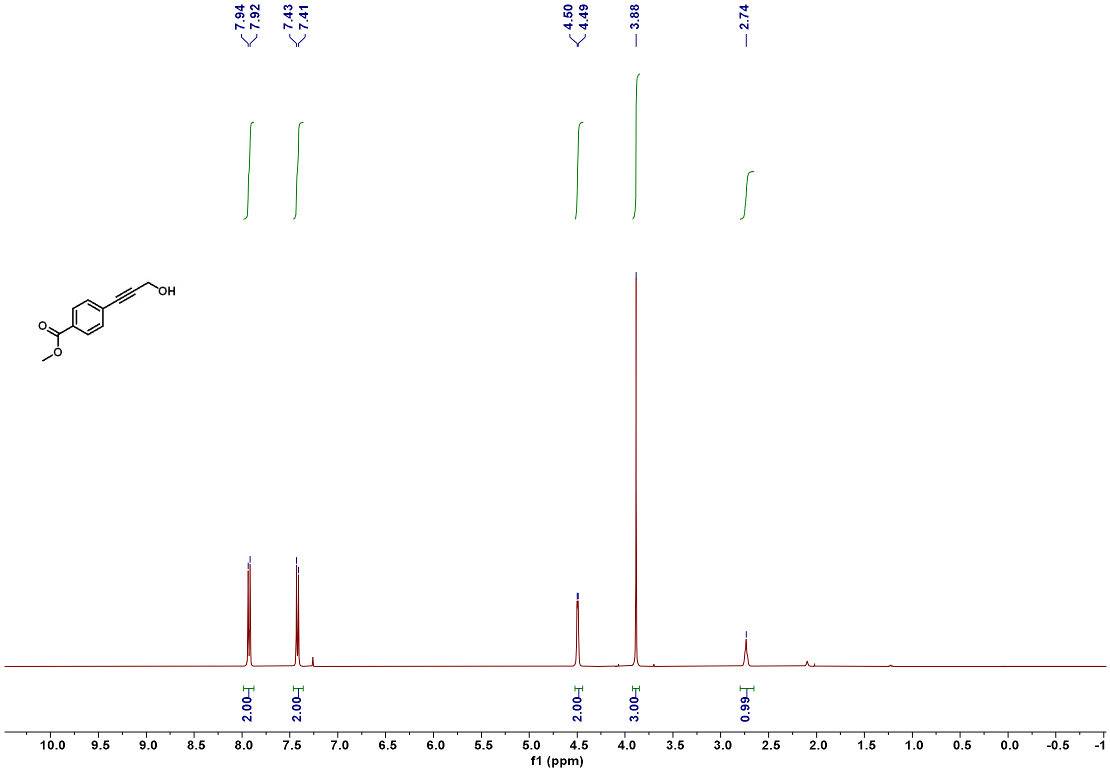

^{1}H NMR (400 MHz, $CDCl_3$) spectrum of 1k

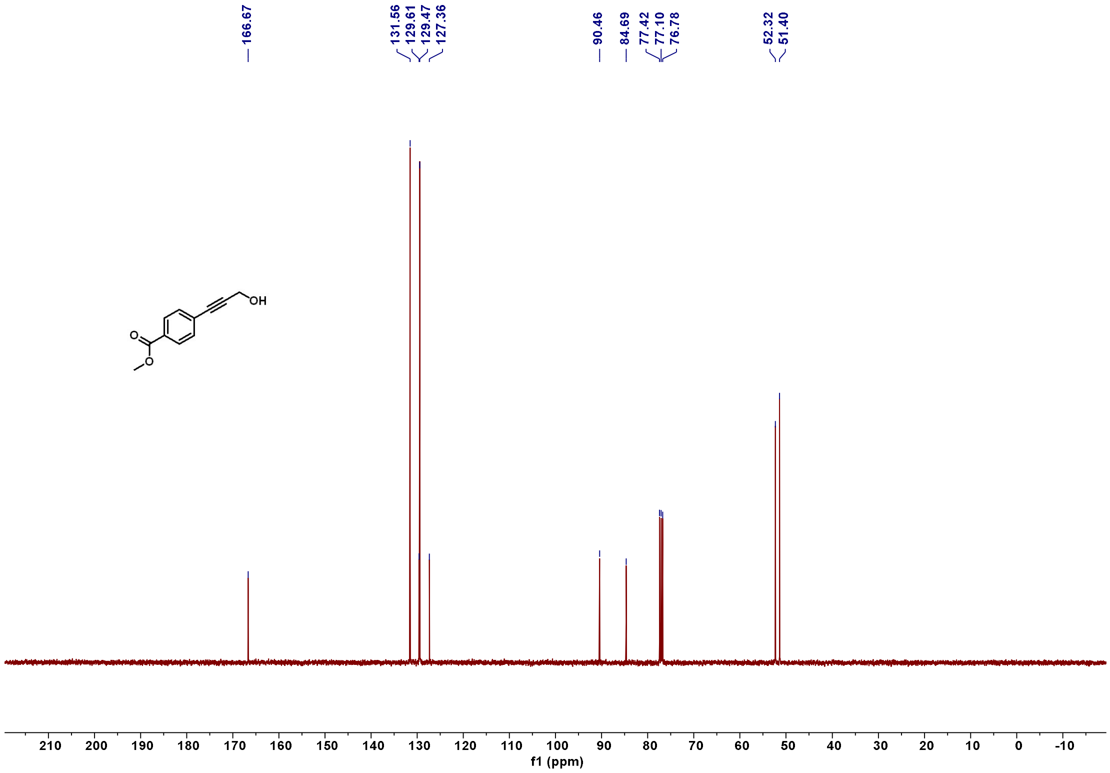

^{13}C NMR (101 MHz, $CDCl_3$) spectrum of 1k

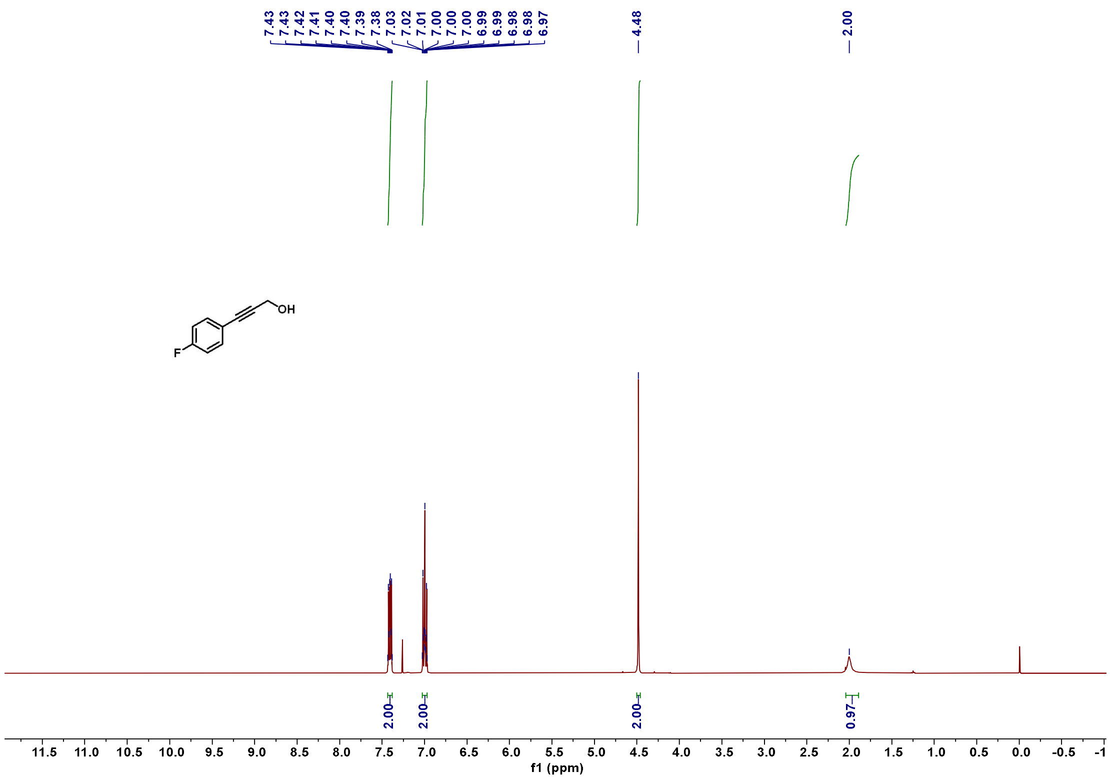

^{1}H NMR (400 MHz, CDCl$_3$) spectrum of 1l

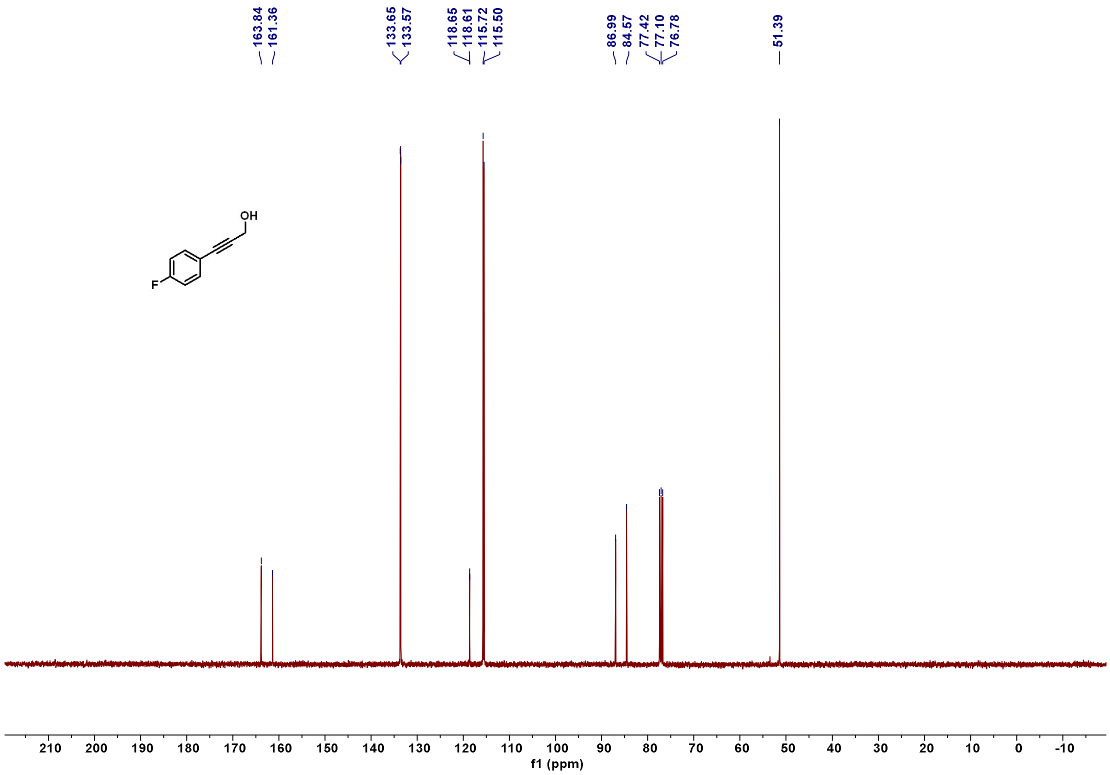

^{13}C NMR (101 MHz, CDCl$_3$) spectrum of 1l

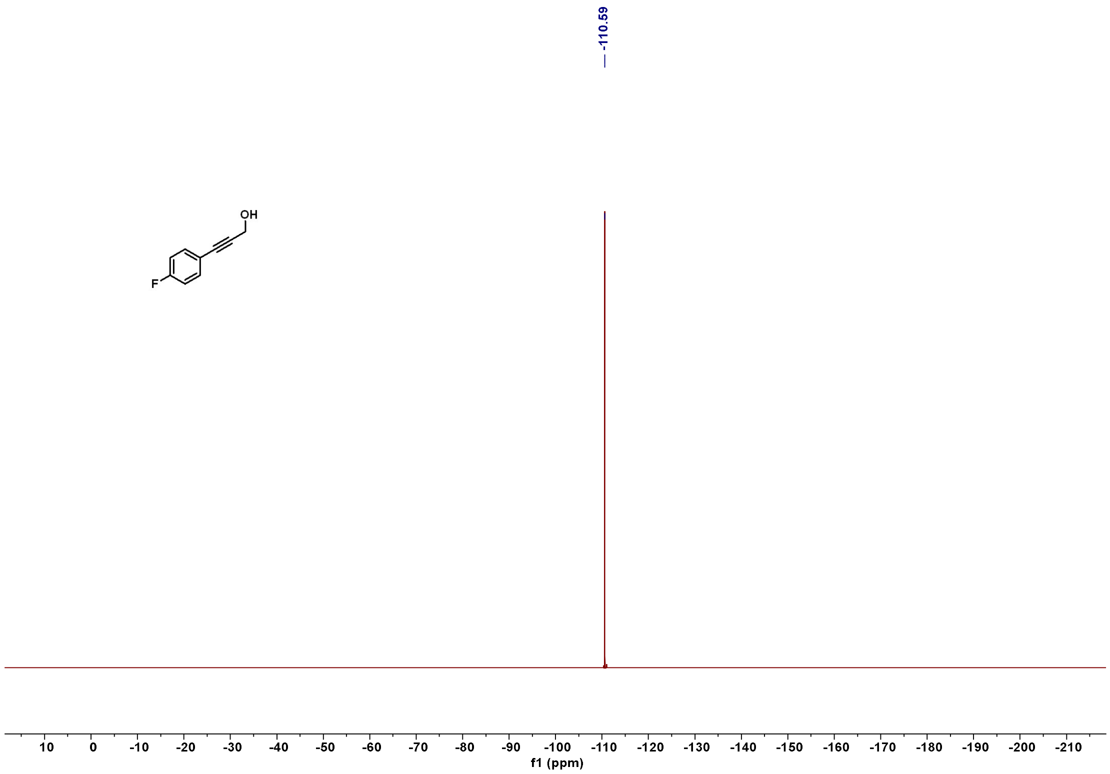

^{19}F NMR (376 MHz, $CDCl_3$) spectrum of 1l

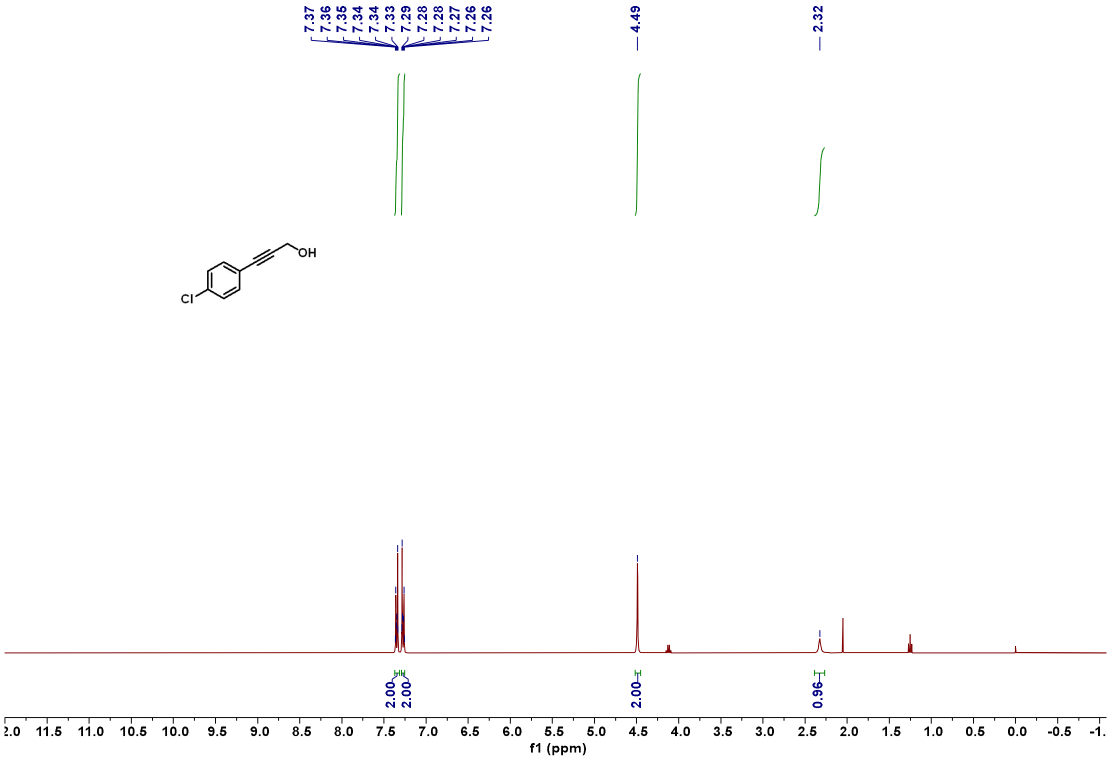

^{1}H NMR (400 MHz, $CDCl_3$) spectrum of 1m

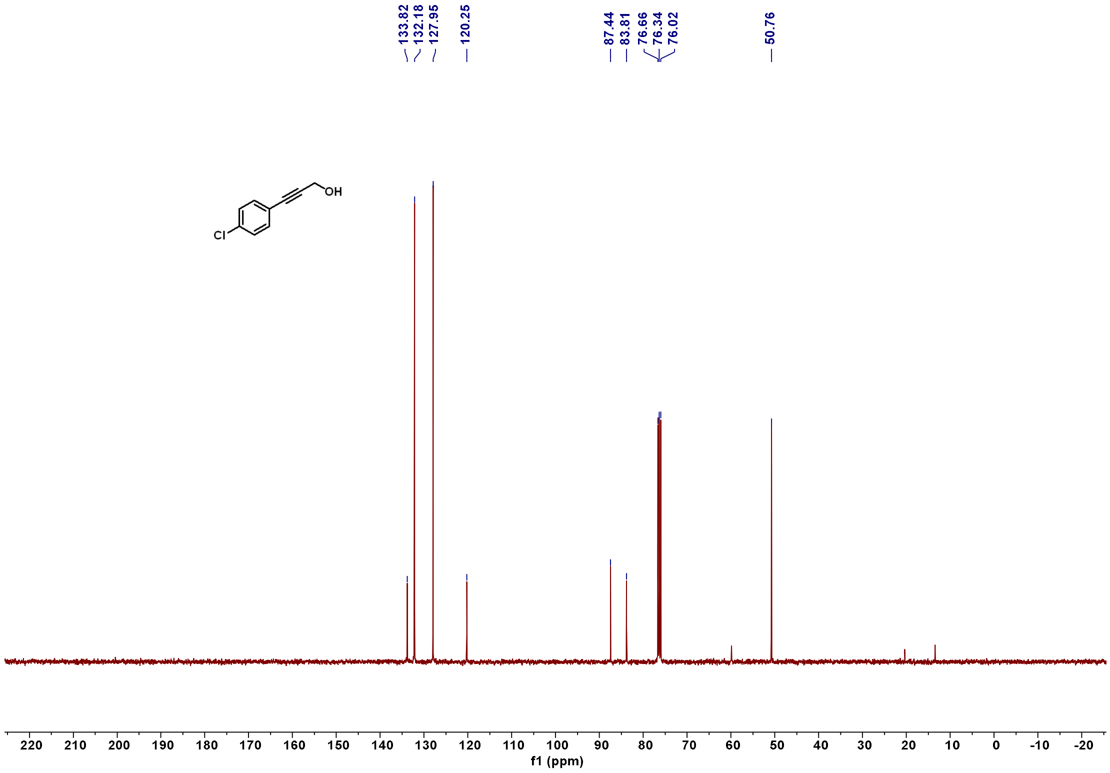

^{13}C NMR (101 MHz, $CDCl_3$) spectrum of 1m

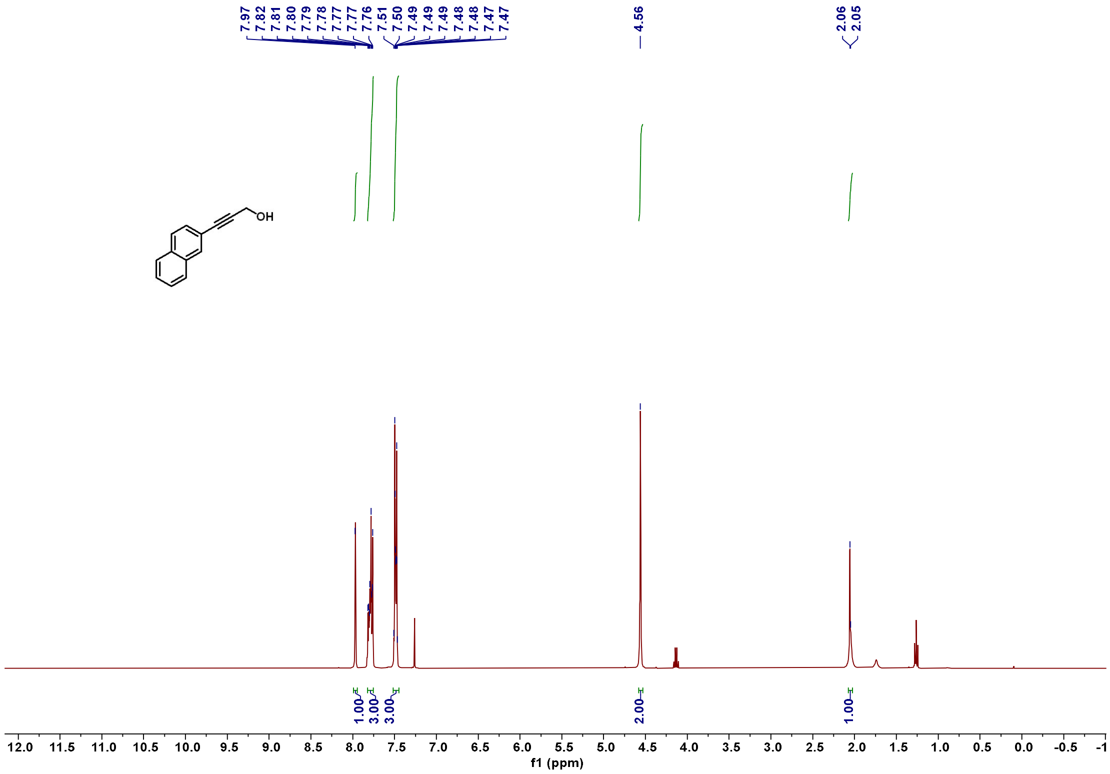

^{1}H NMR (400 MHz, CDCl$_3$) spectrum of 1n

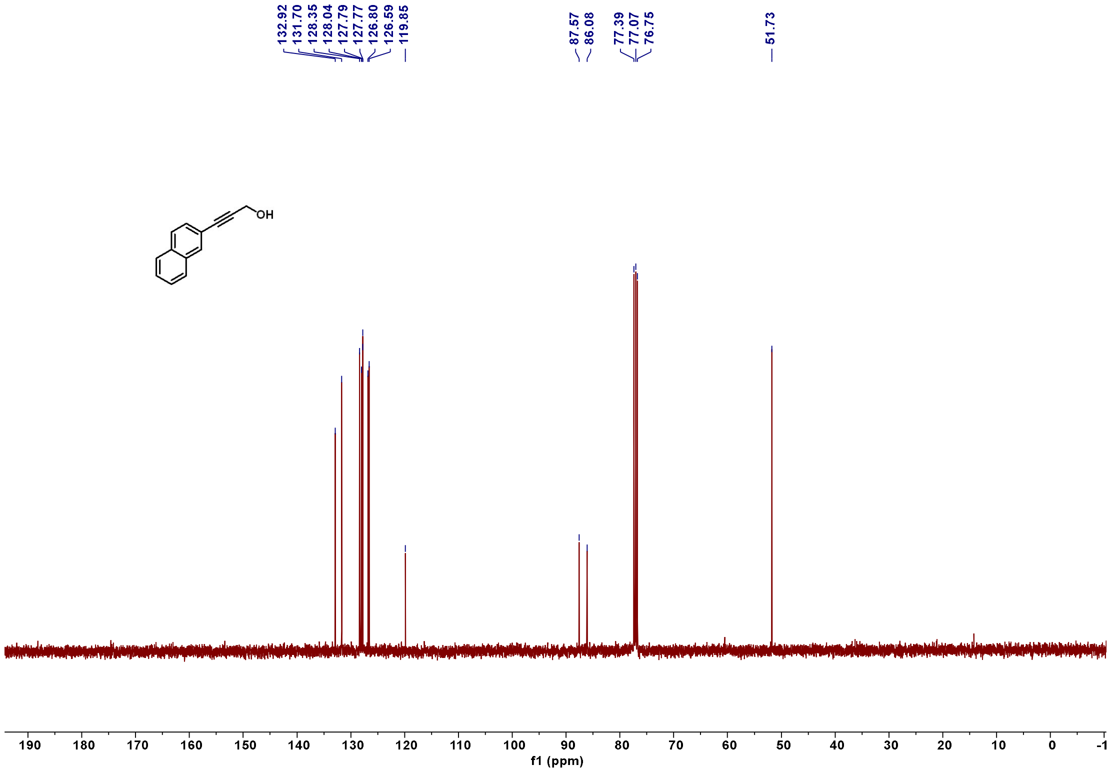

^{13}C NMR (101 MHz, CDCl$_3$) spectrum of 1n

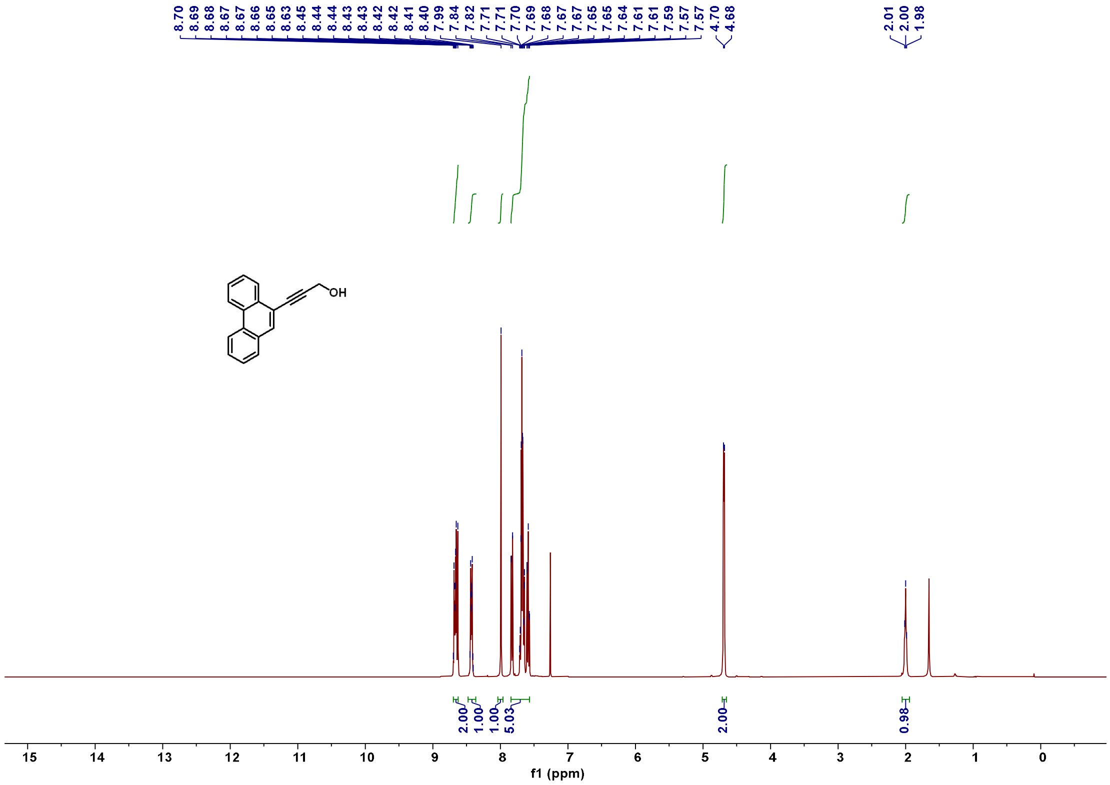

^{1}H NMR (400 MHz, CDCl₃) spectrum of 1o

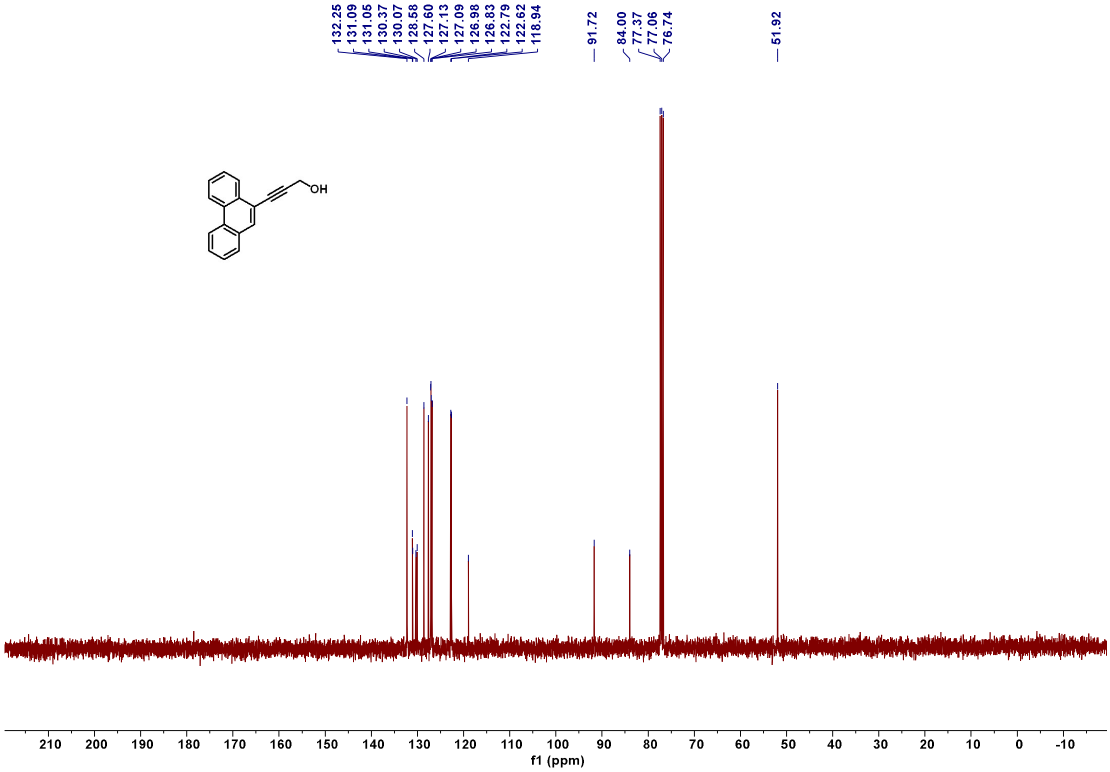

^{13}C NMR (101 MHz, CDCl₃) spectrum of 1o

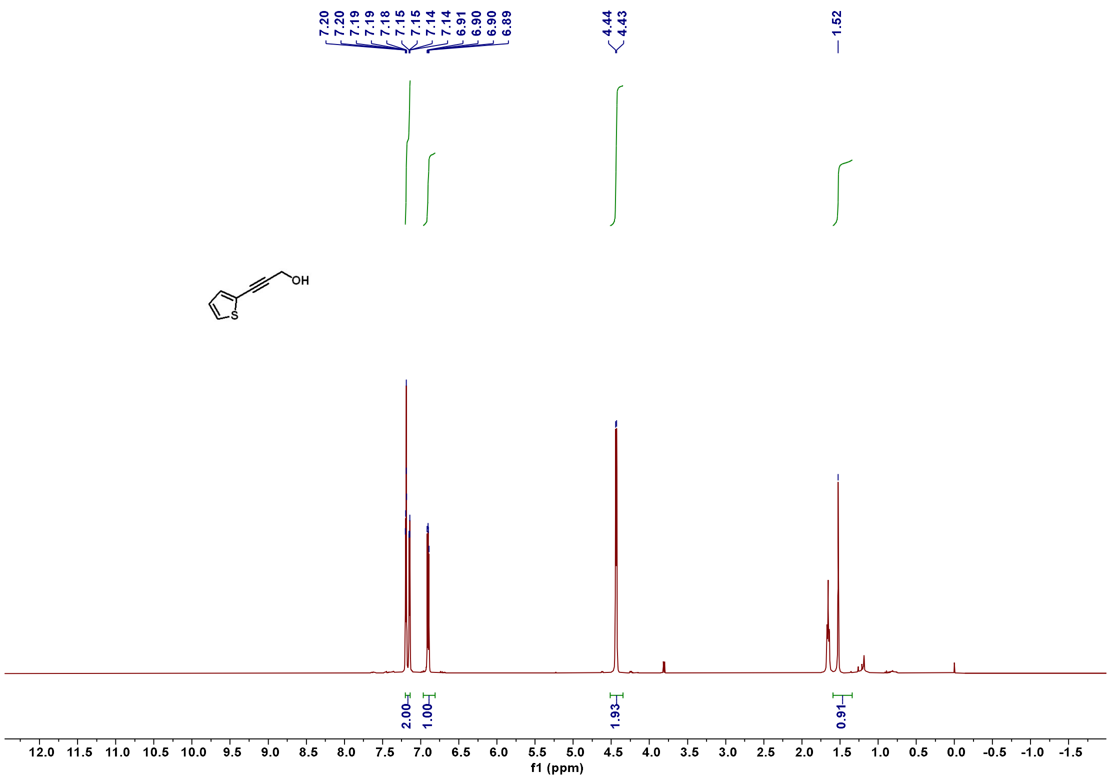

^{1}H NMR (400 MHz, CDCl3) spectrum of 1p

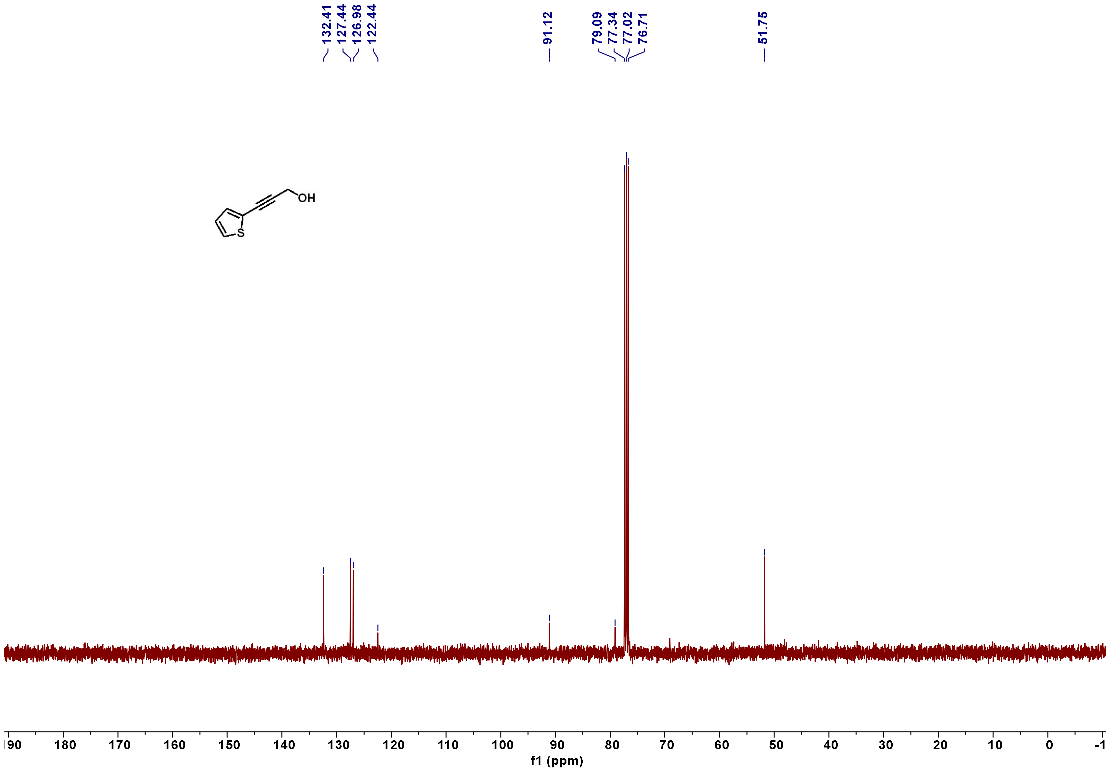

^{13}C NMR (101 MHz, CDCl3) spectrum of 1p

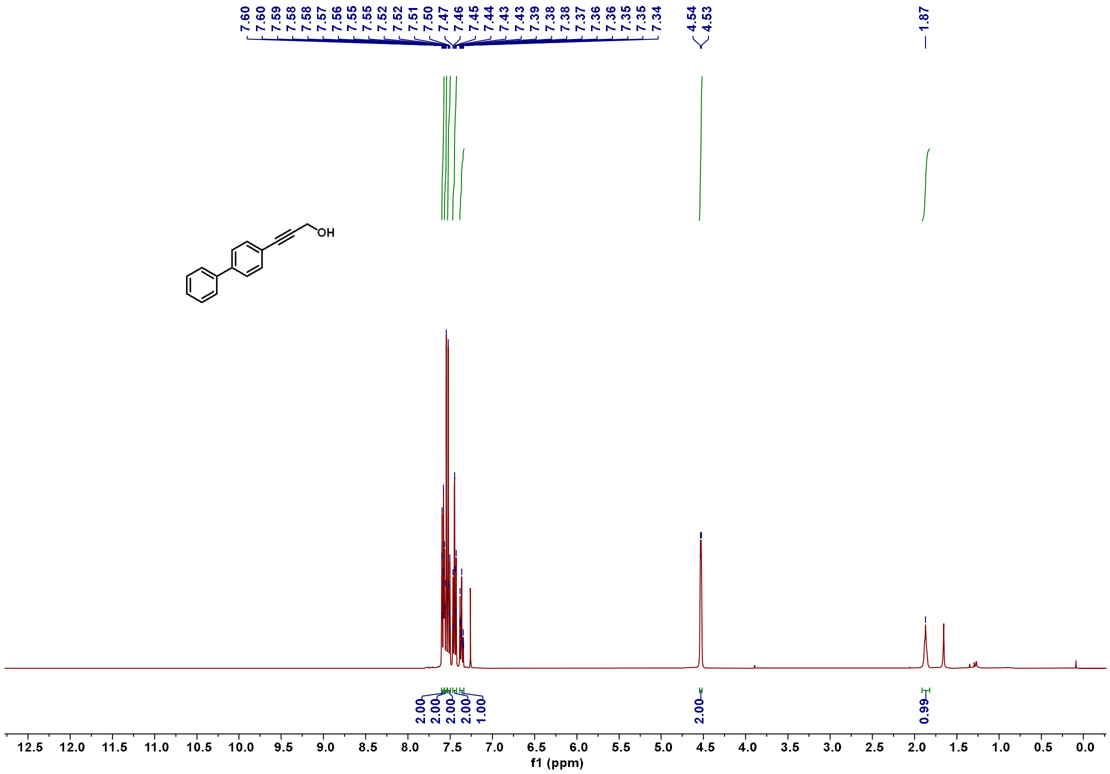

^{1}H NMR (400 MHz, CDCl$_3$) spectrum of 1q

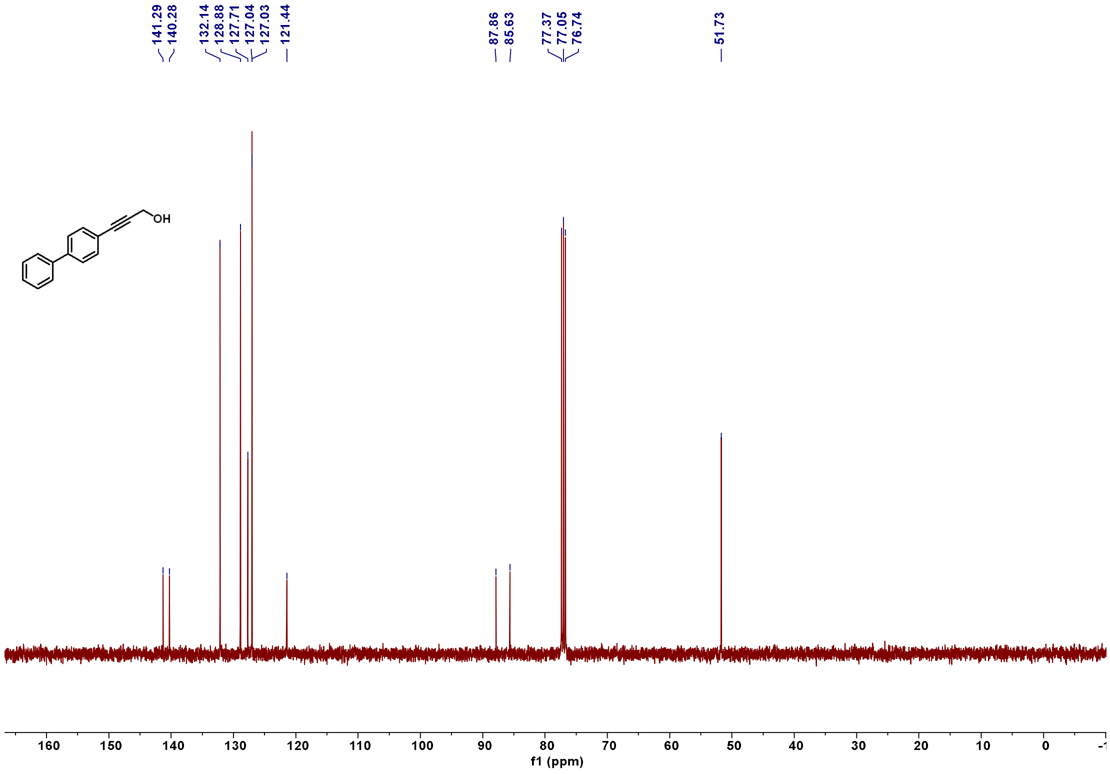

^{13}C NMR (101 MHz, CDCl$_3$) spectrum of 1q

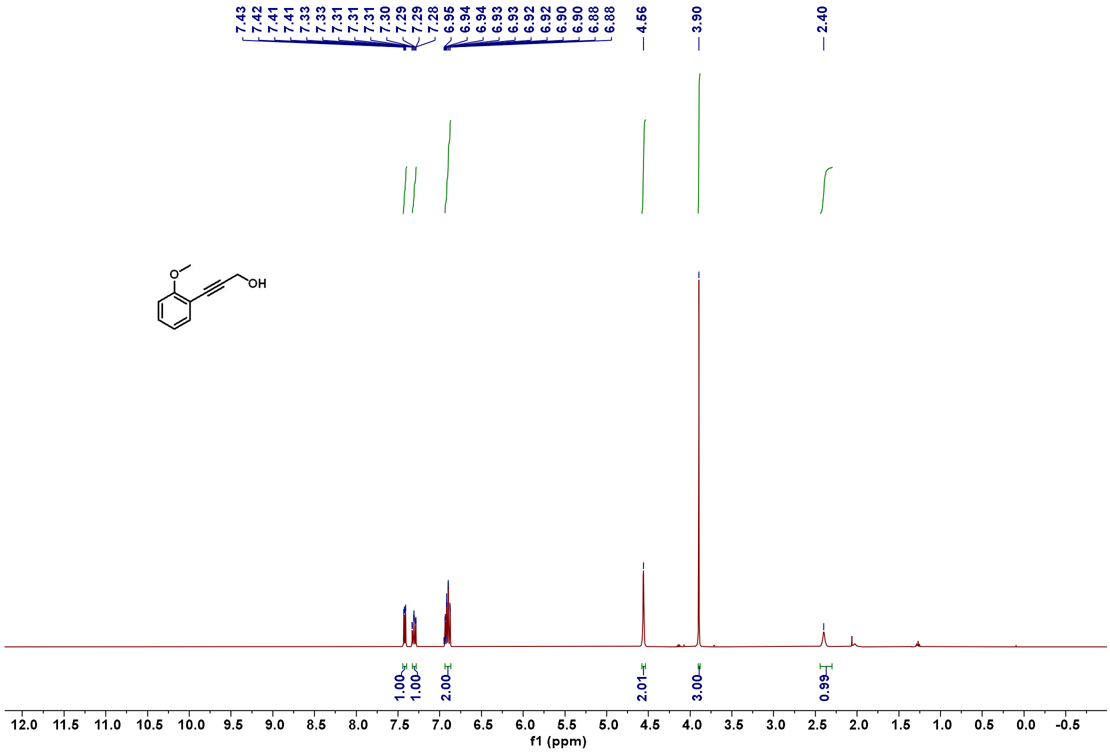

^{1}H NMR (400 MHz, $CDCl_3$) spectrum of 1r

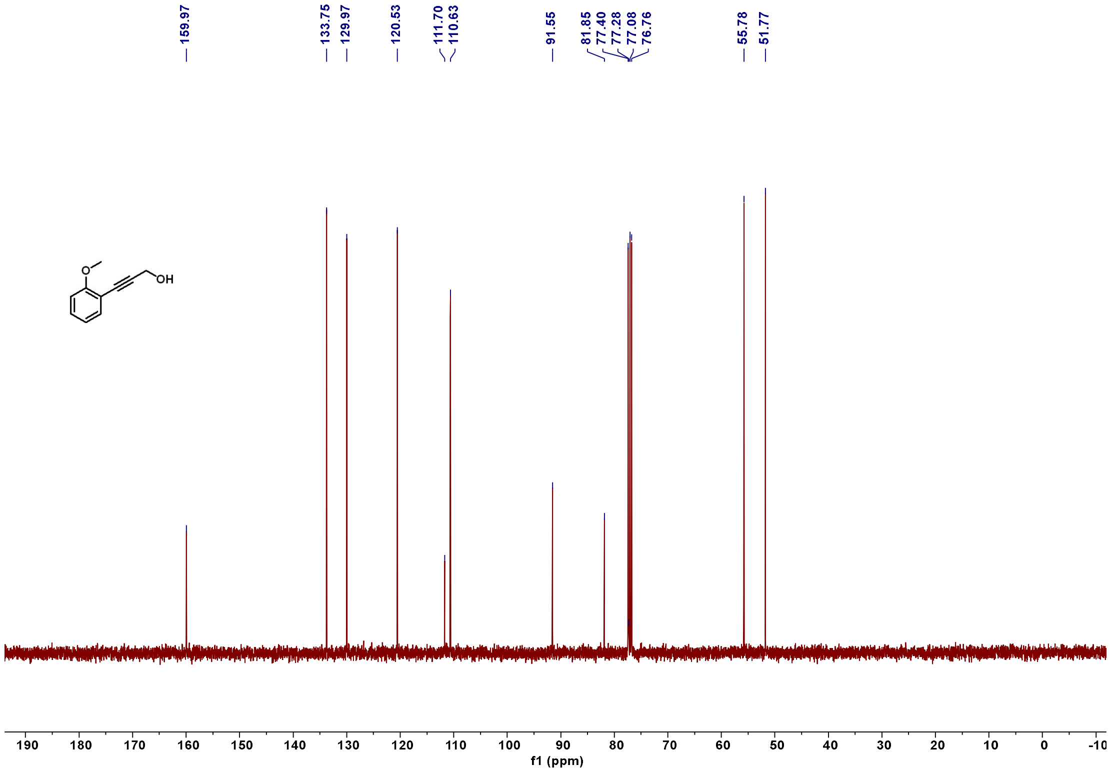

^{13}C NMR (101 MHz, $CDCl_3$) spectrum of 1r

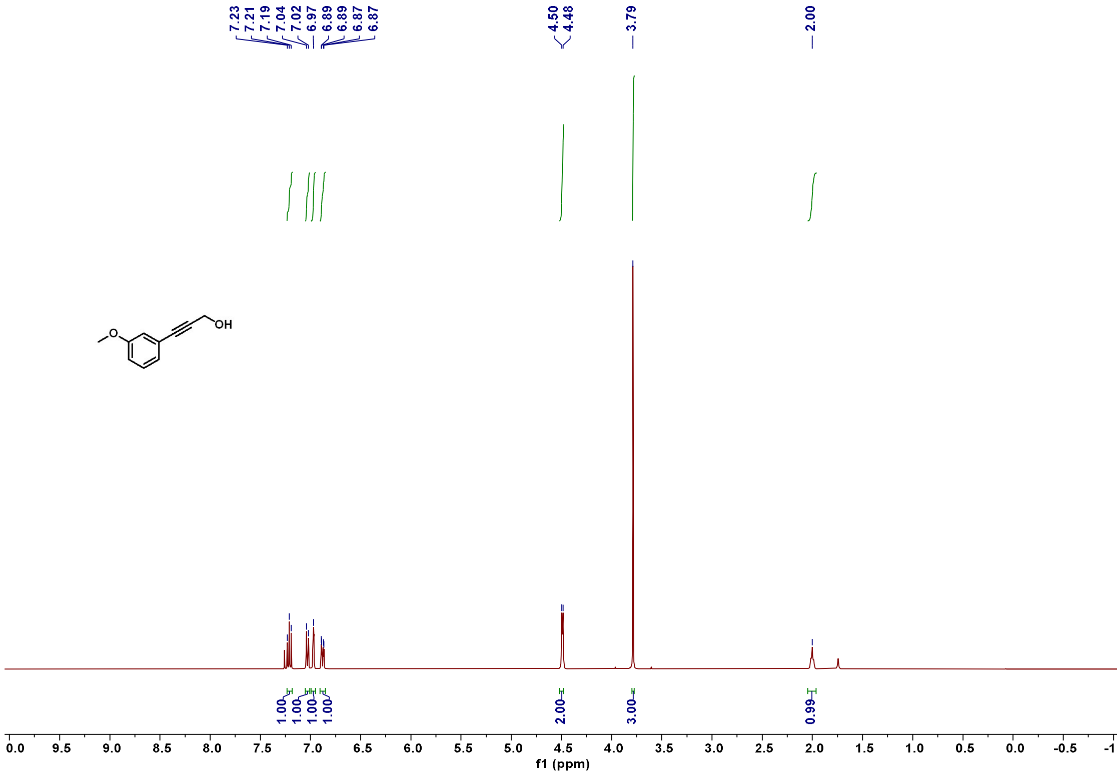

^{1}H NMR (400 MHz, $CDCl_3$) spectrum of 1s

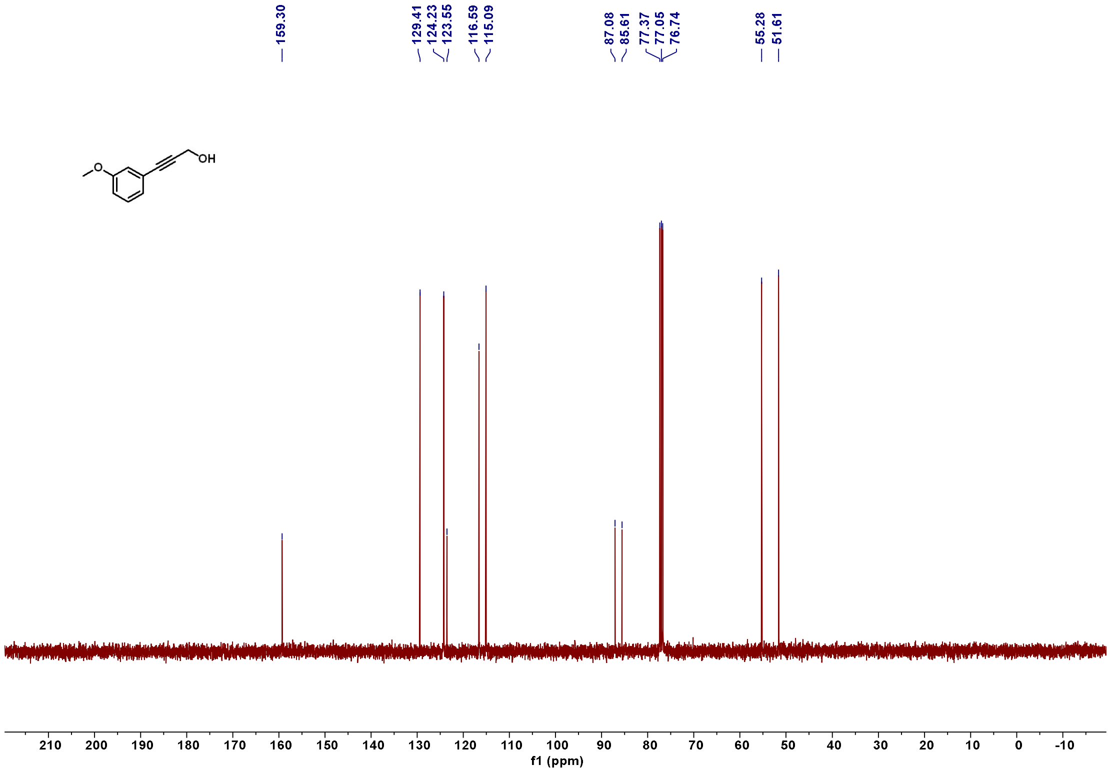

^{13}C NMR (101 MHz, $CDCl_3$) spectrum of 1s

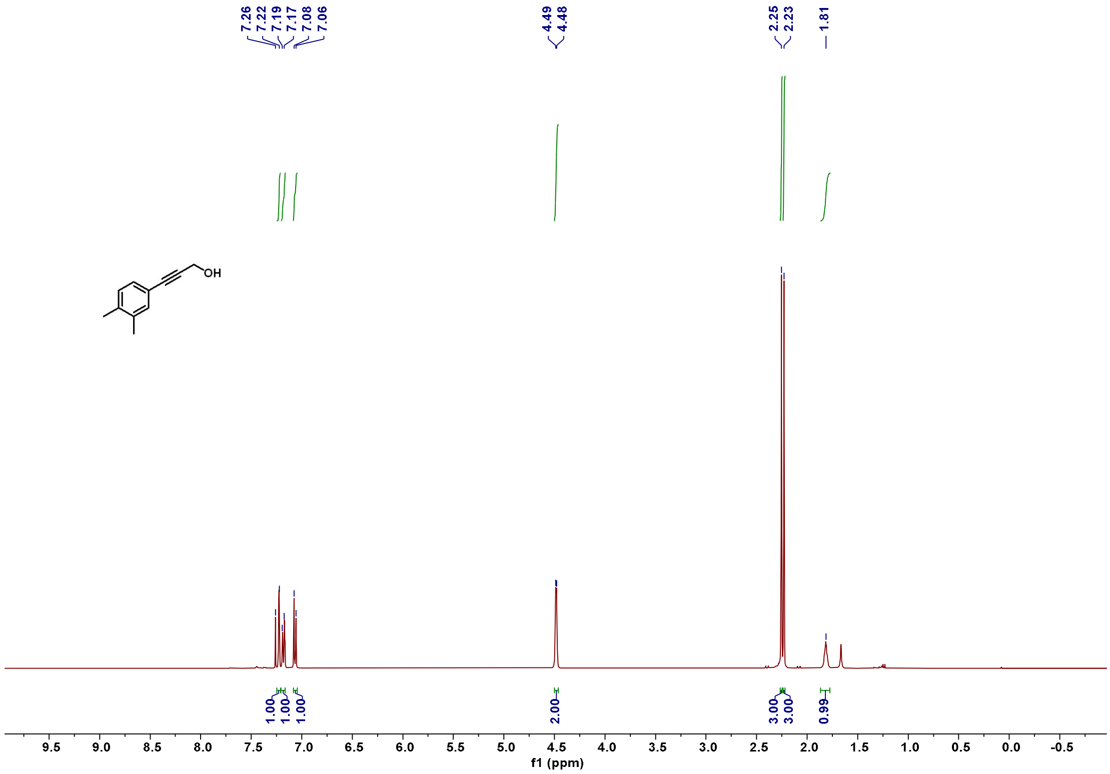

^{1}H NMR (400 MHz, $CDCl_3$) spectrum of 1t

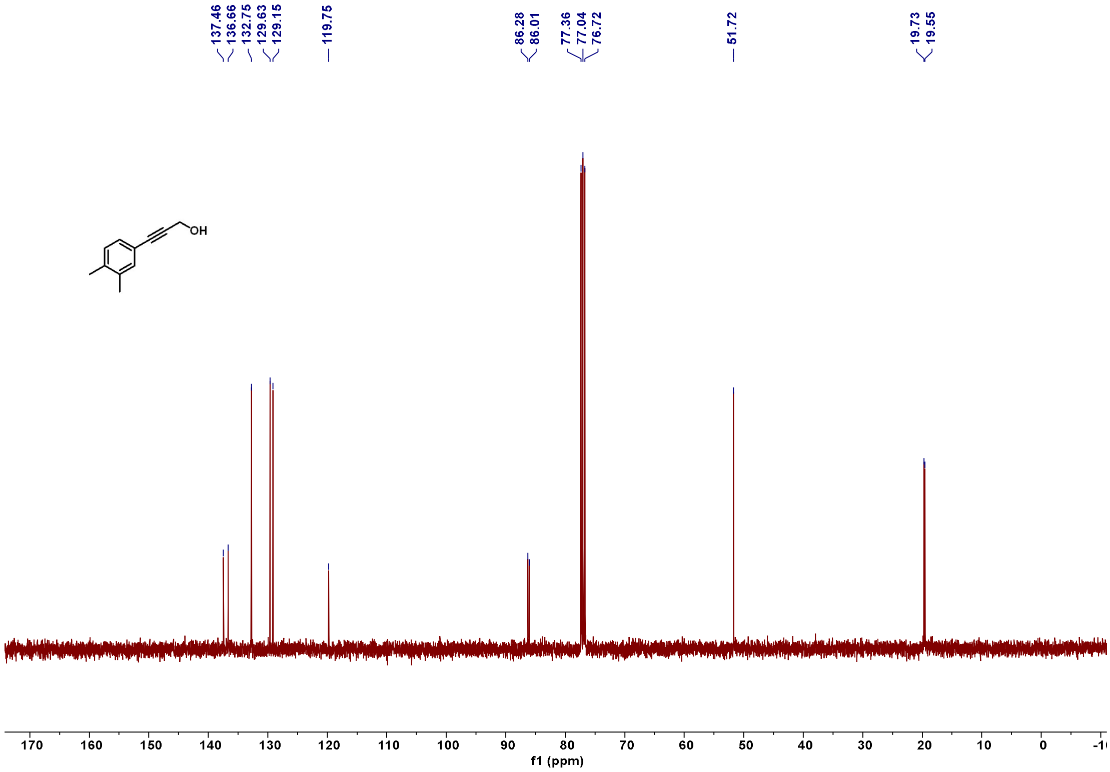

^{13}C NMR (101 MHz, $CDCl_3$) spectrum of 1t

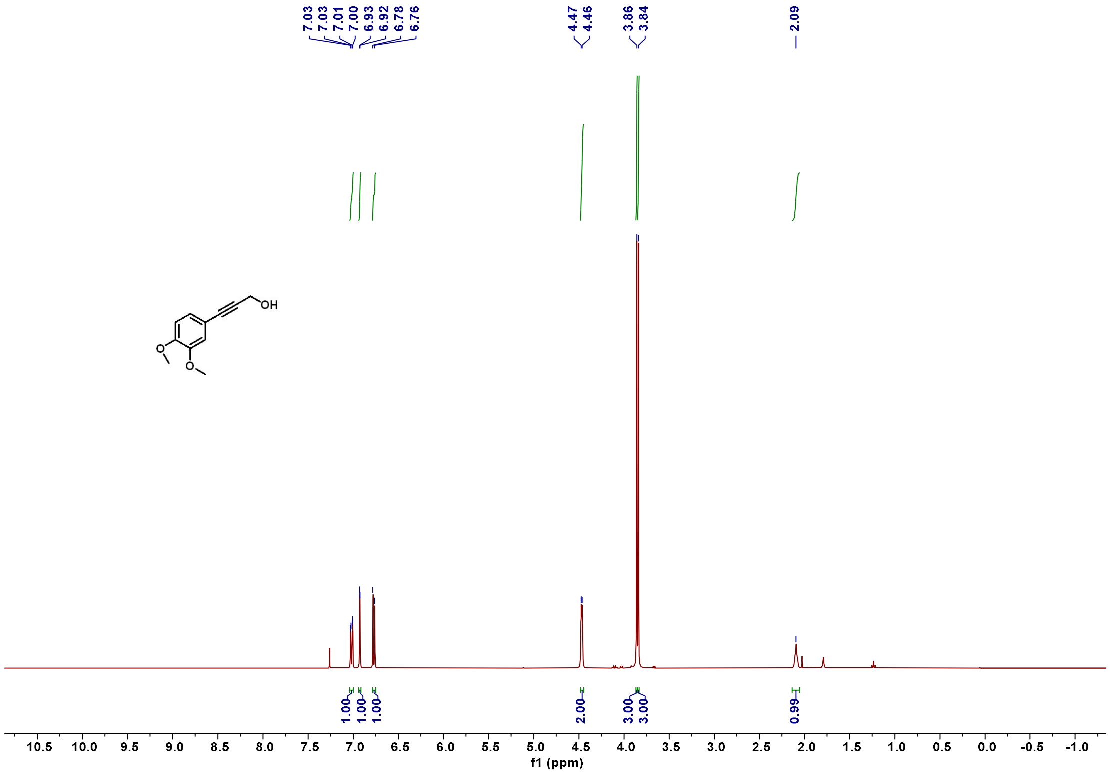

^{1}H NMR (400 MHz, CDCl3) spectrum of 1u

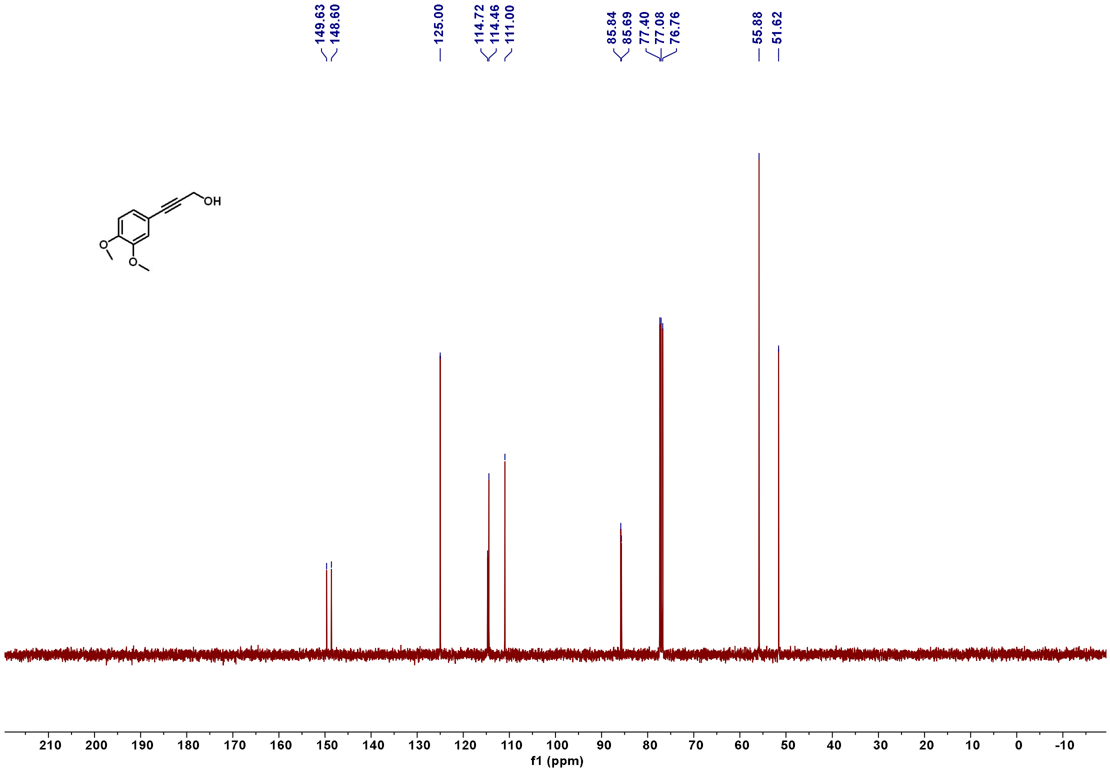

^{13}C NMR (101 MHz, CDCl3) spectrum of 1u

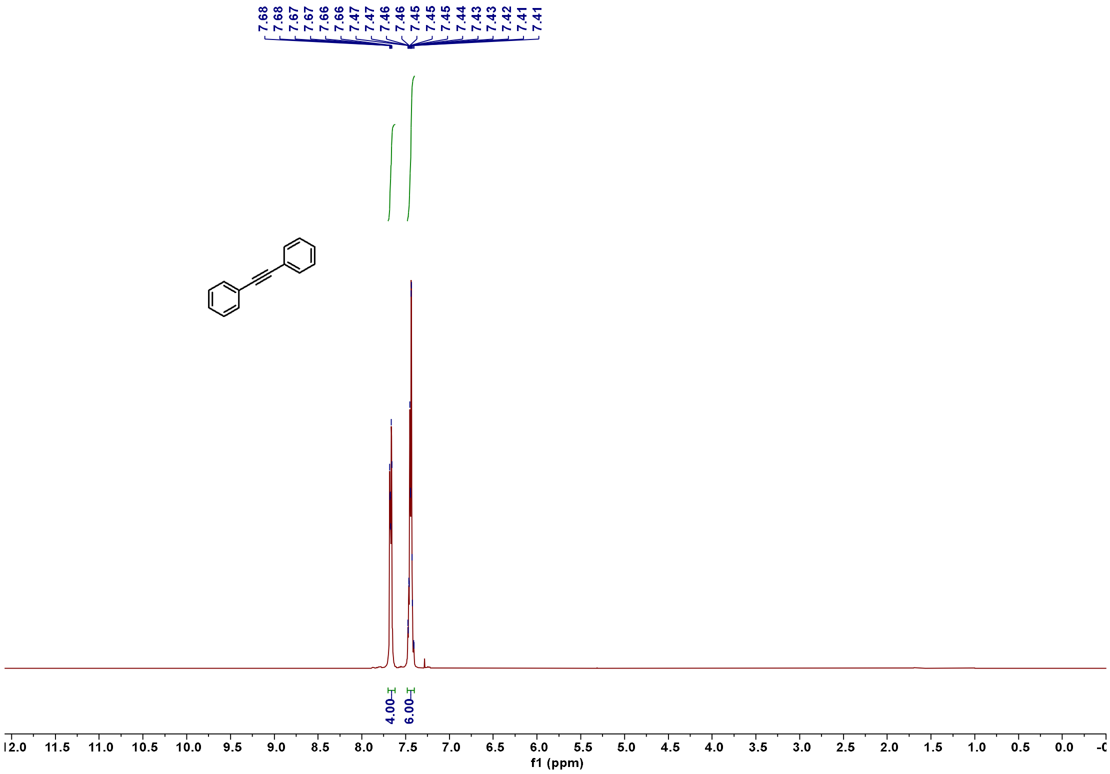

^{1}H NMR (400 MHz, CDCl3) spectrum of 3a

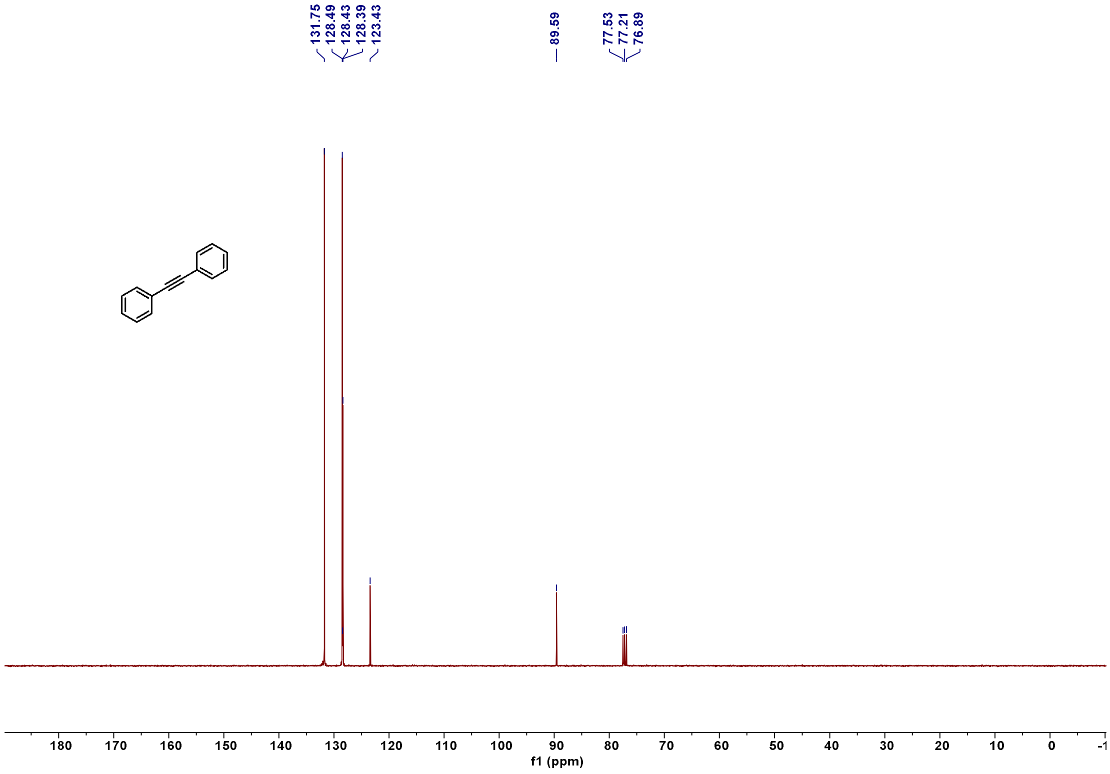

^{13}C NMR (101 MHz, CDCl3) spectrum of 3a

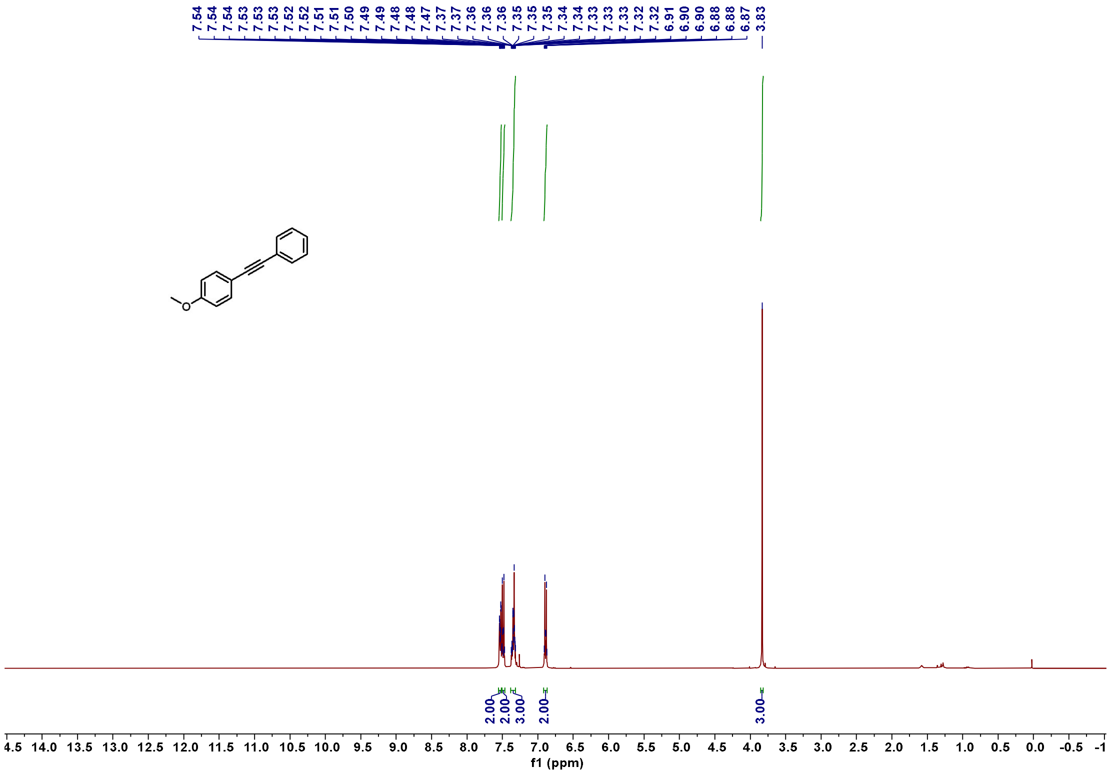

^{1}H NMR (400 MHz, CDCl3) spectrum of 3b

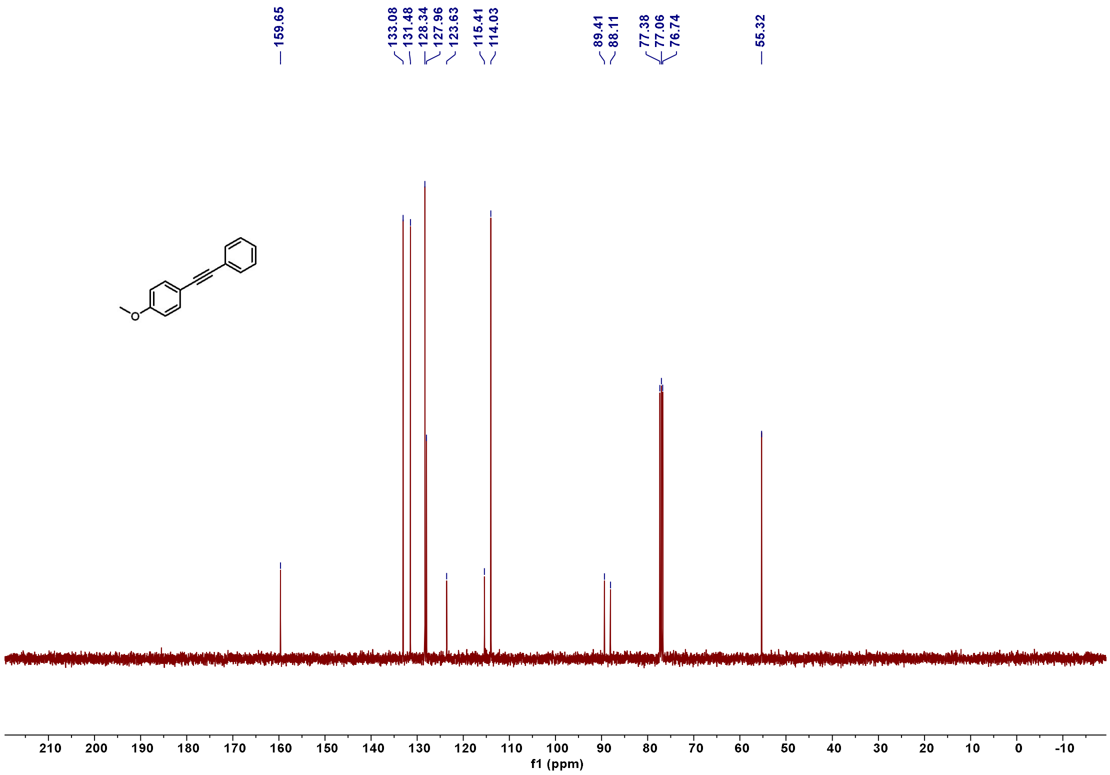

^{13}C NMR (101 MHz, CDCl3) spectrum of 3b

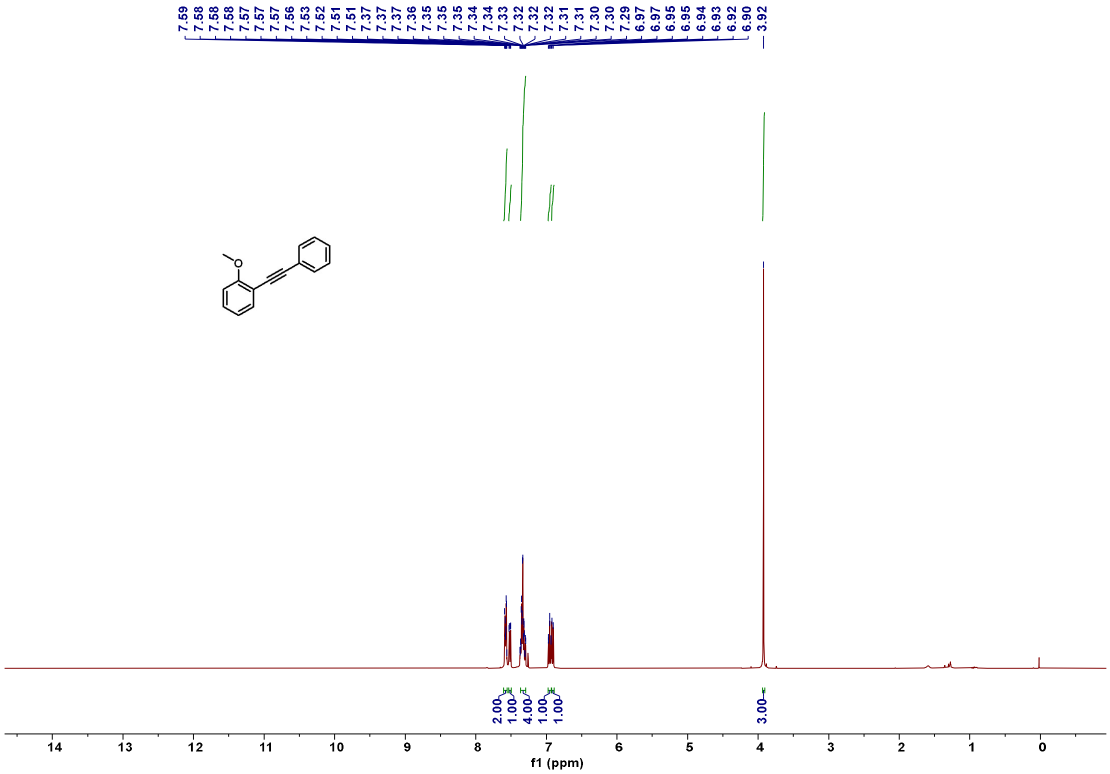

^{1}H NMR (400 MHz, CDCl$_3$) spectrum of 3c

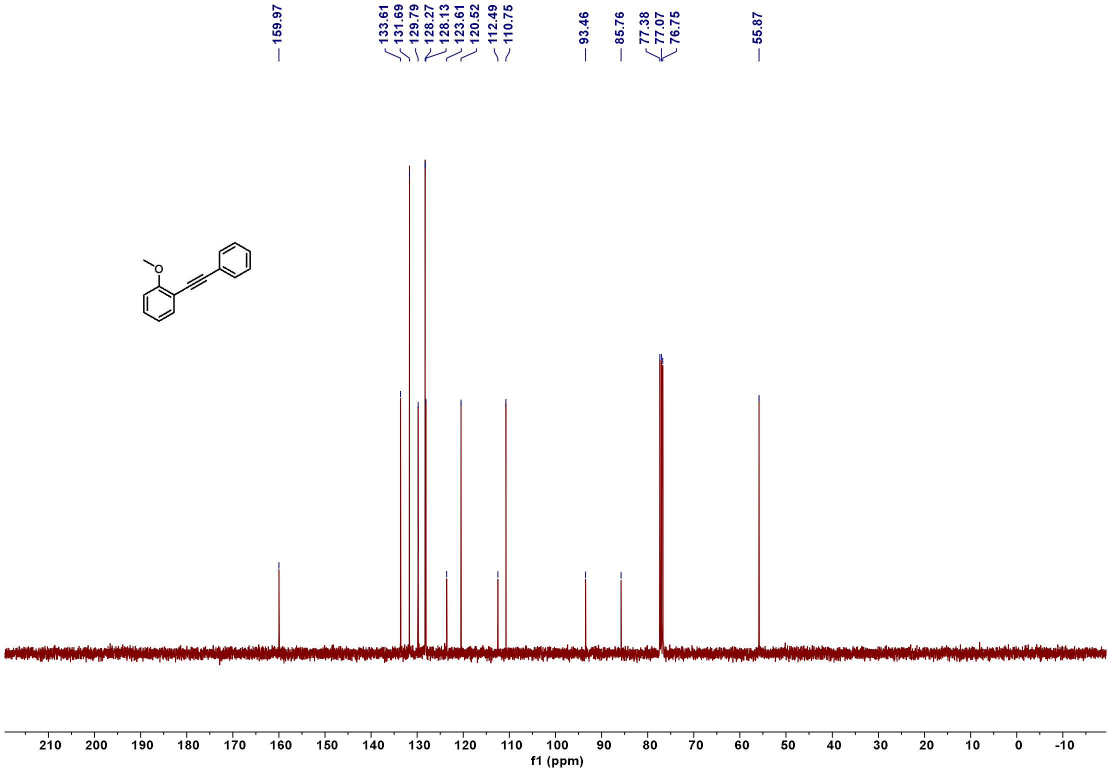

^{13}C NMR (101 MHz, CDCl$_3$) spectrum of 3c

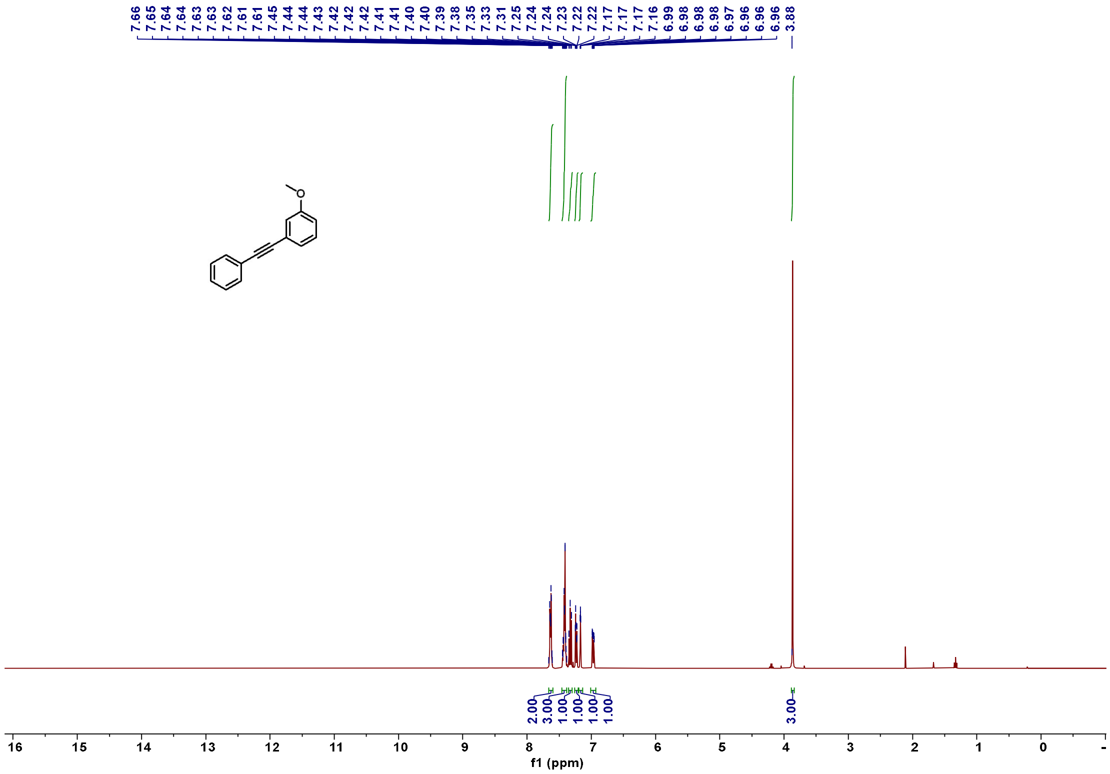

^{1}H NMR (400 MHz, CDCl3) spectrum of 3d

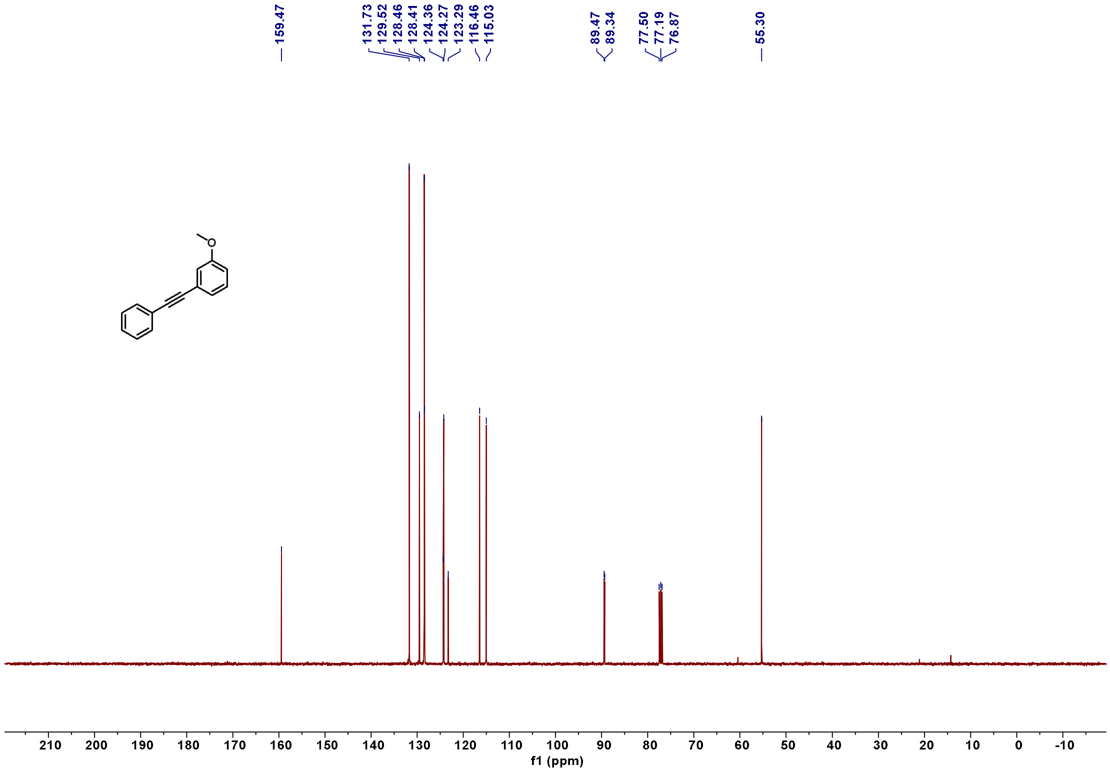

^{13}C NMR (101 MHz, CDCl3) spectrum of 3d

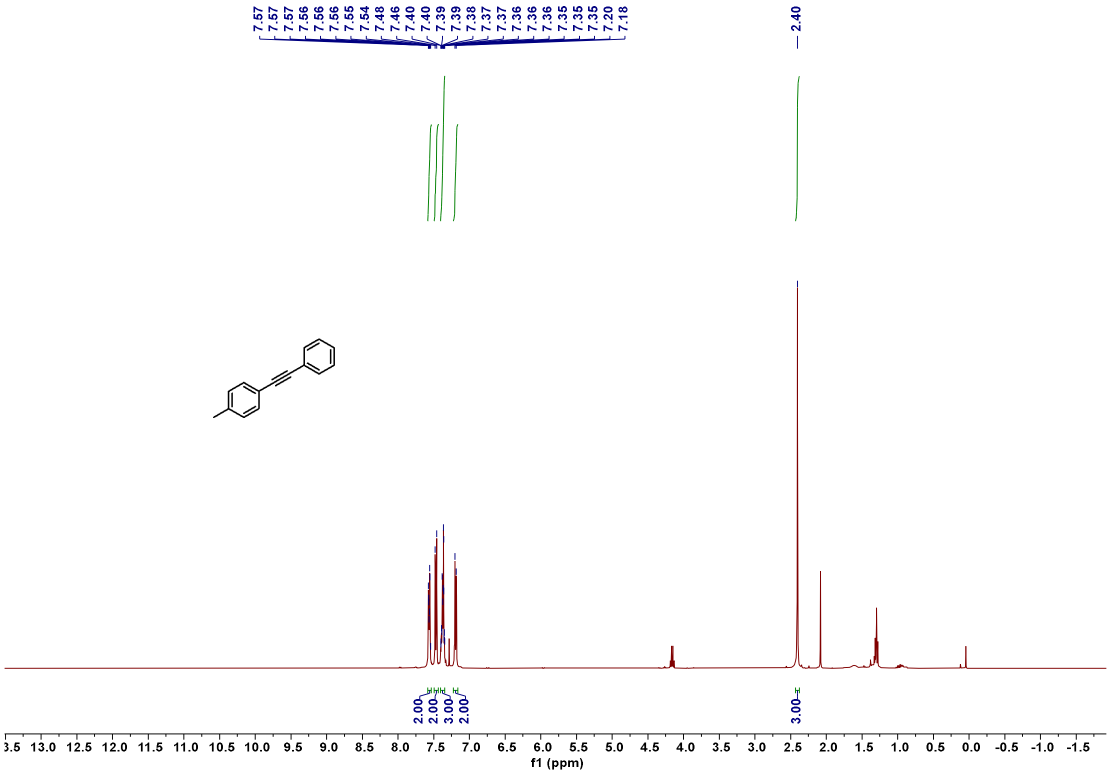

^{1}H NMR (400 MHz, $CDCl_3$) spectrum of 3e

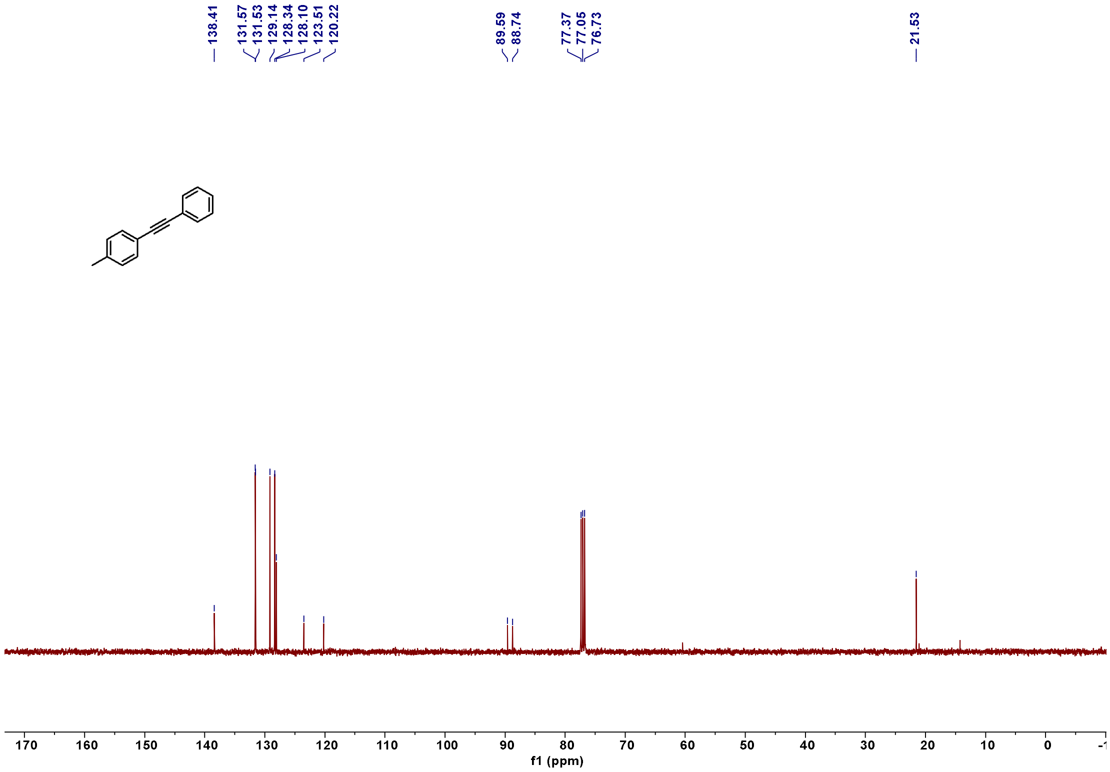

^{13}C NMR (101 MHz, $CDCl_3$) spectrum of 3e

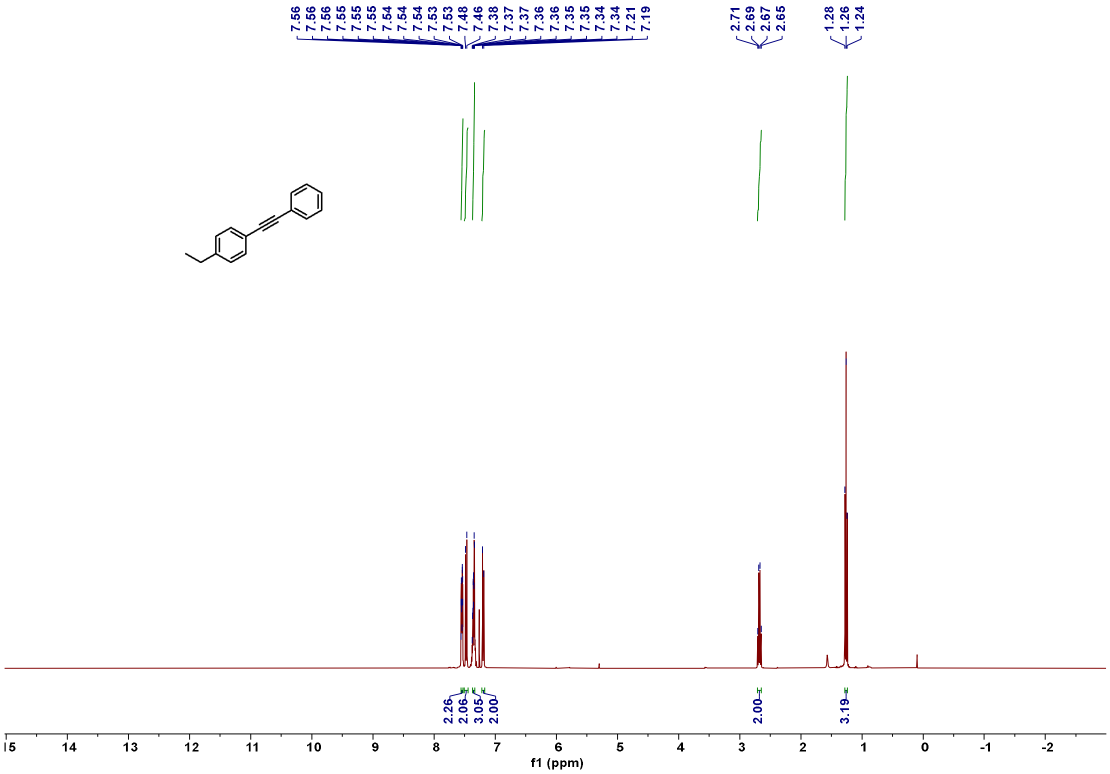

^{1}H NMR (400 MHz, $CDCl_3$) spectrum of 3f

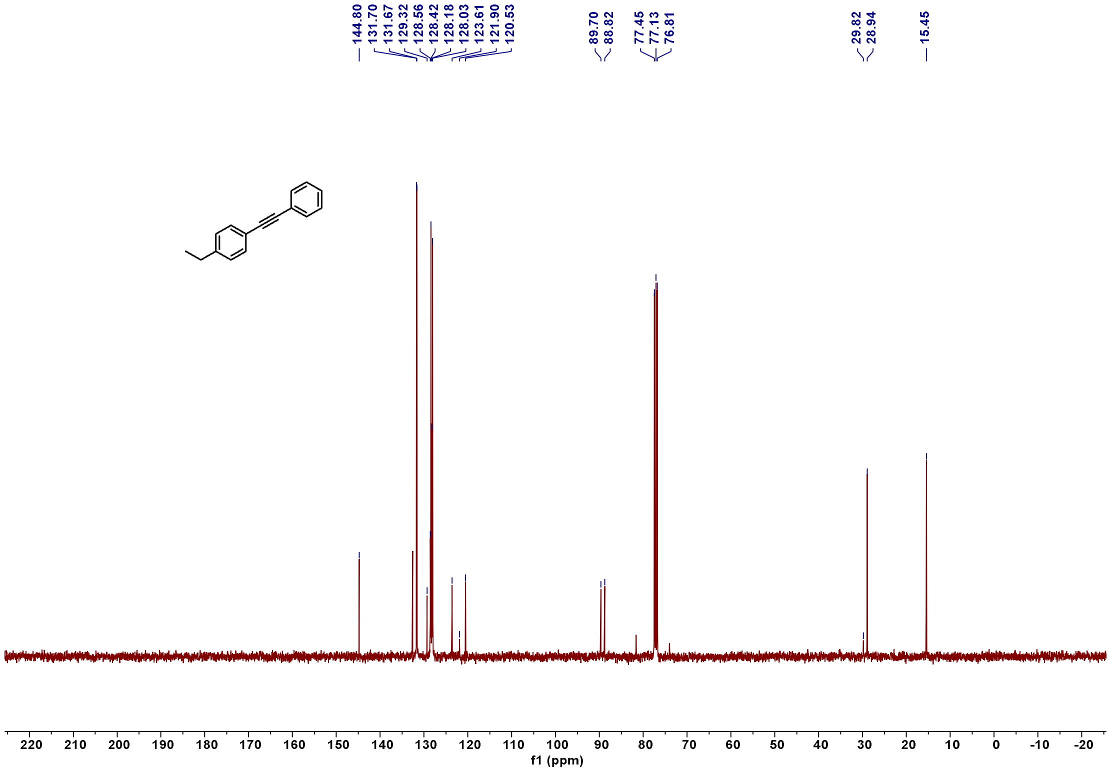

^{13}C NMR (101 MHz, $CDCl_3$) spectrum of 3f

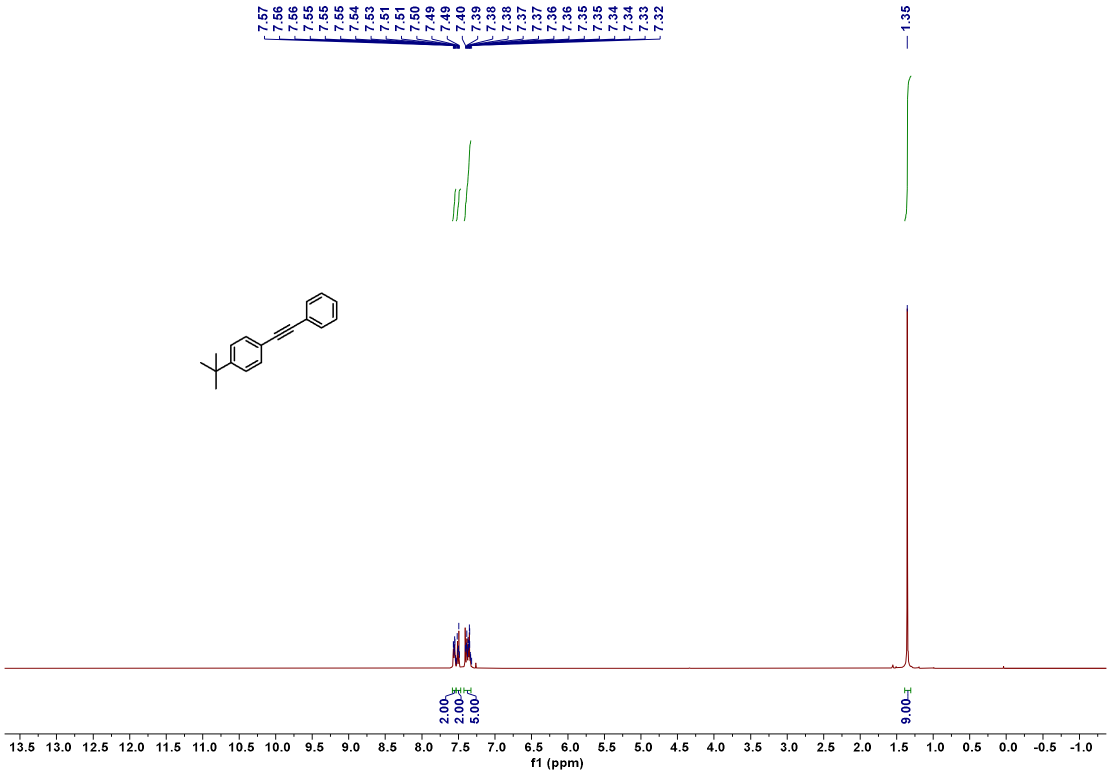

^{1}H NMR (400 MHz, $CDCl_3$) spectrum of 3g

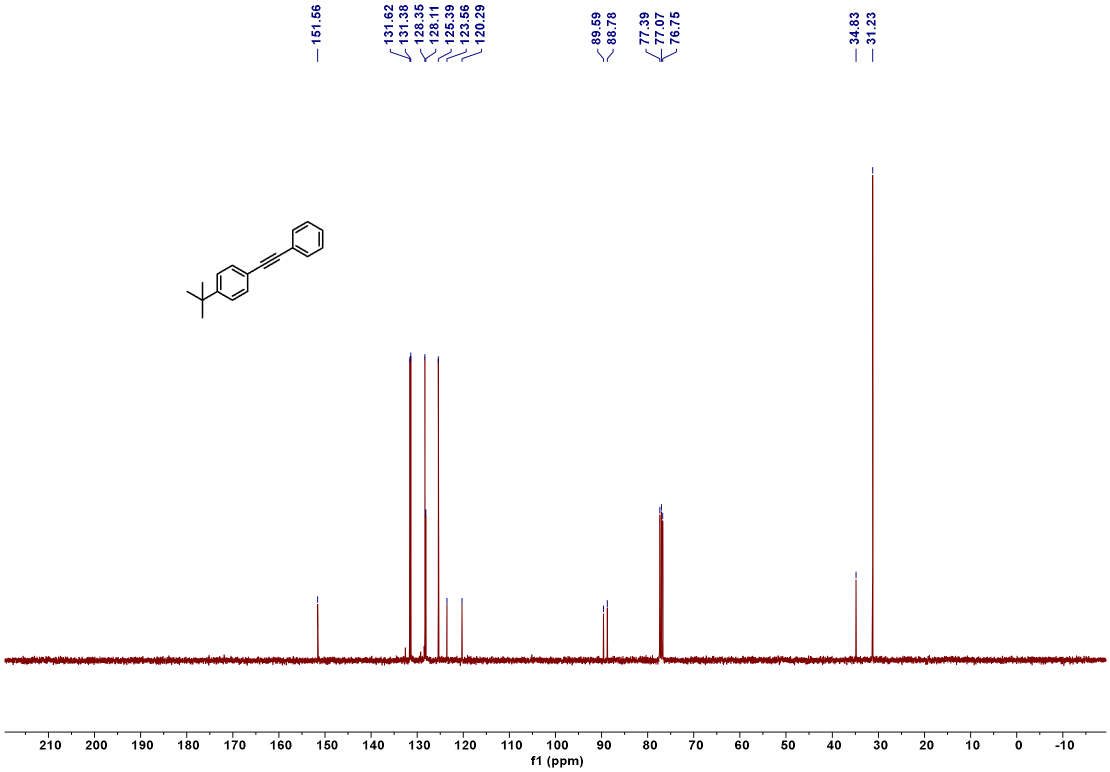

^{13}C NMR (101 MHz, $CDCl_3$) spectrum of 3g

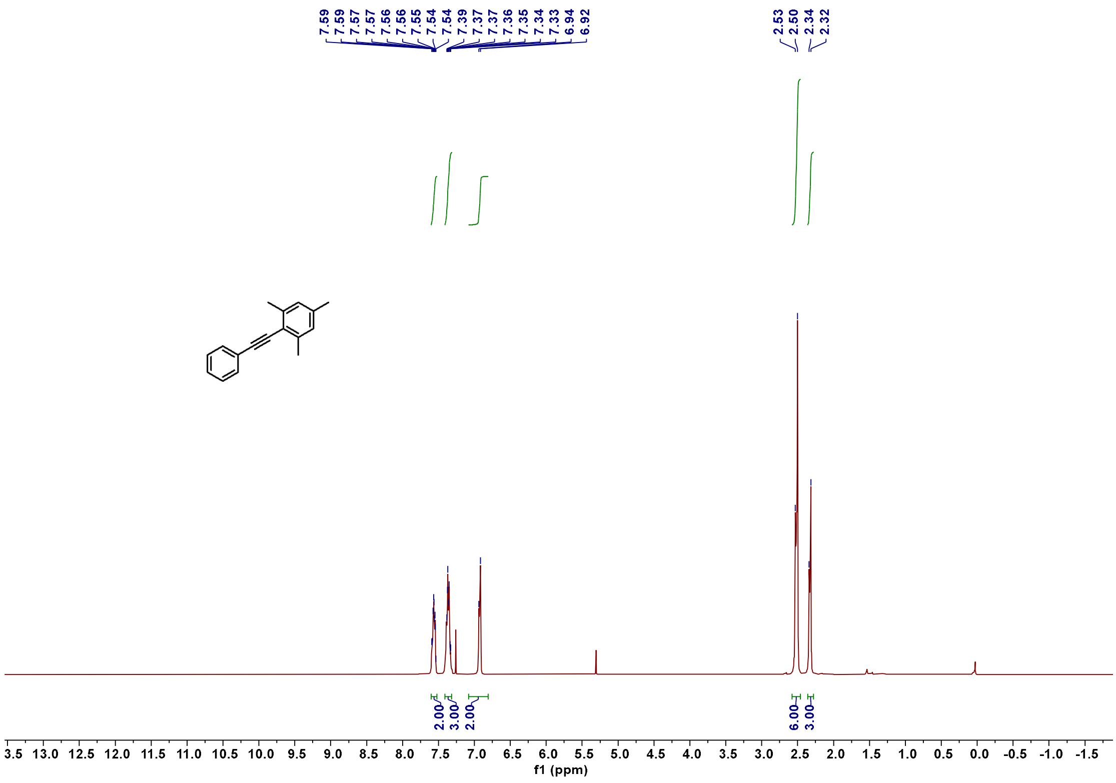

^{1}H NMR (400 MHz, $CDCl_3$) spectrum of 3h

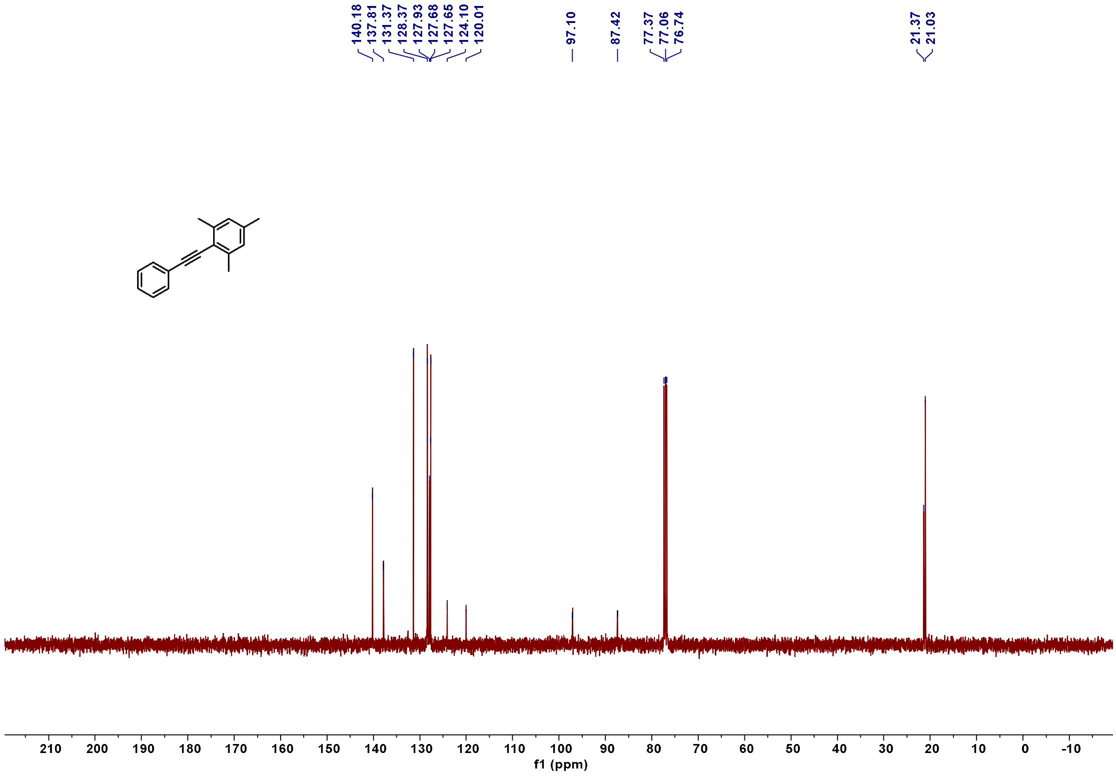

^{13}C NMR (101 MHz, $CDCl_3$) spectrum of 3h

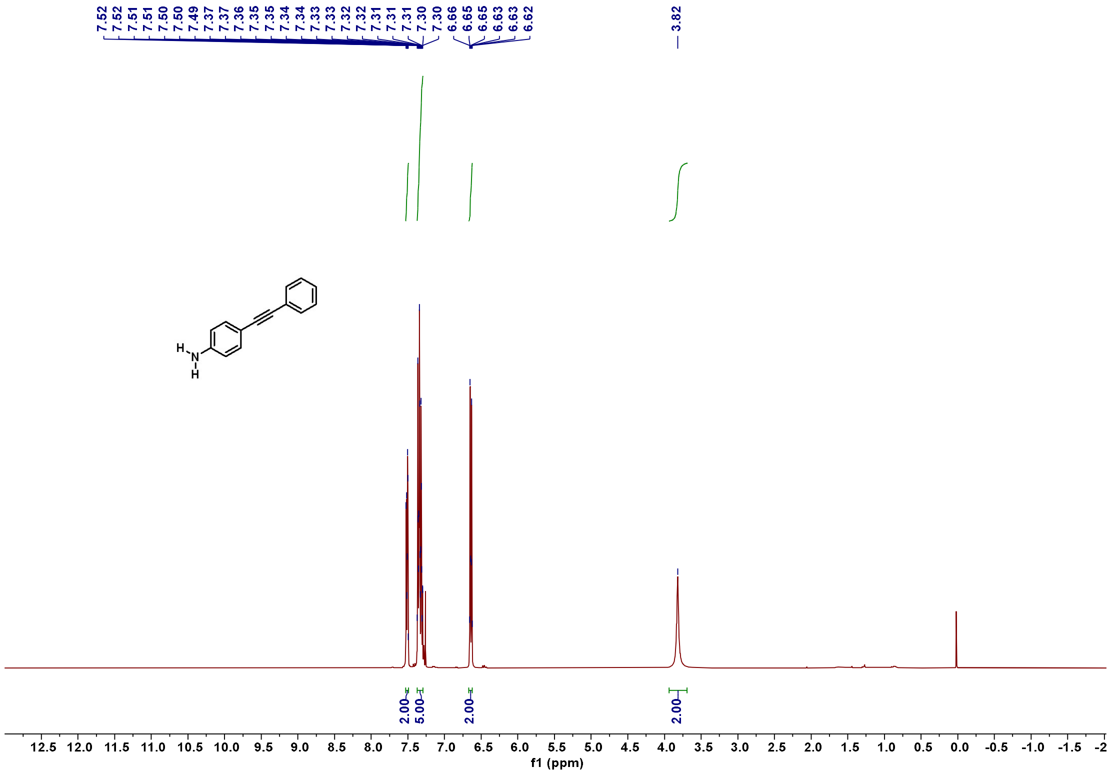

^{1}H NMR (400 MHz, $CDCl_3$) spectrum of 3i

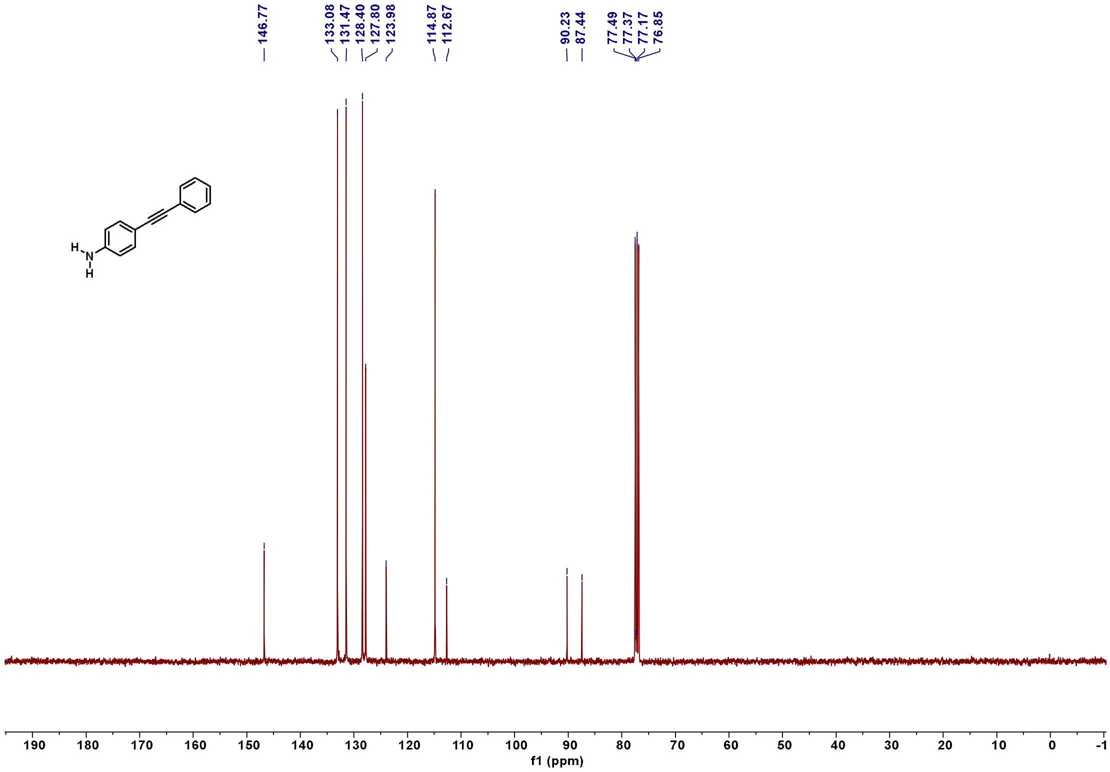

^{13}C NMR (101 MHz, $CDCl_3$) spectrum of 3i

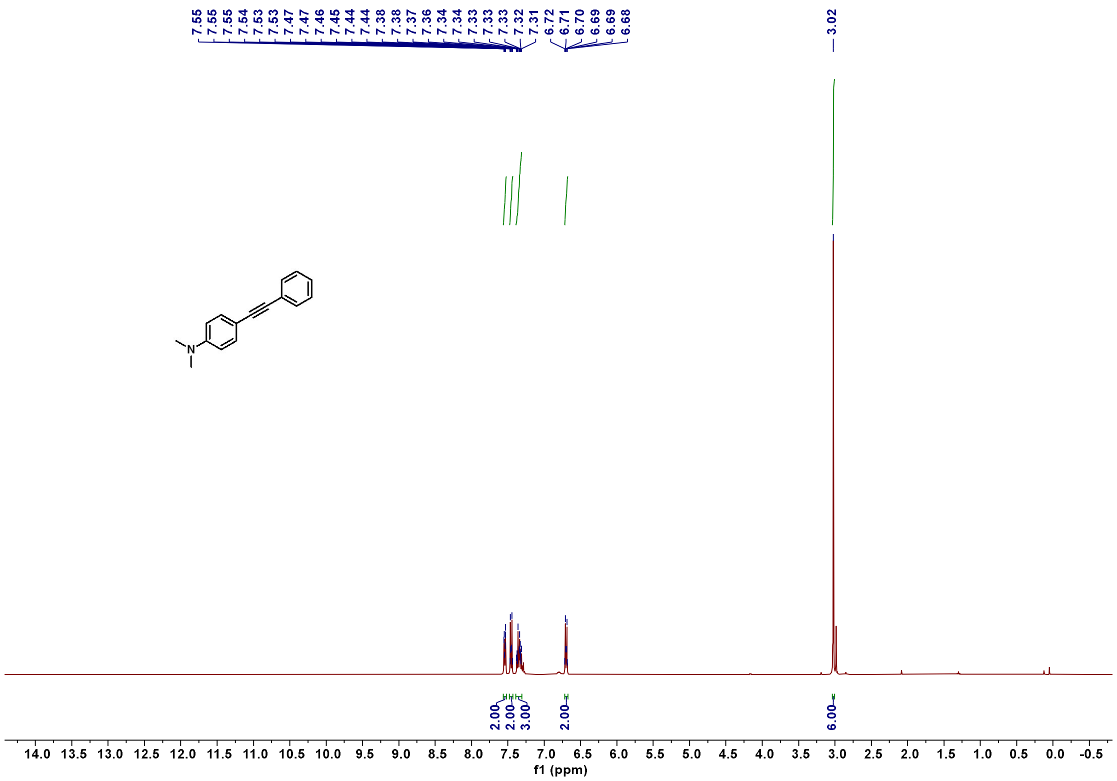

^{1}H NMR (400 MHz, CDCl$_3$) spectrum of 3j

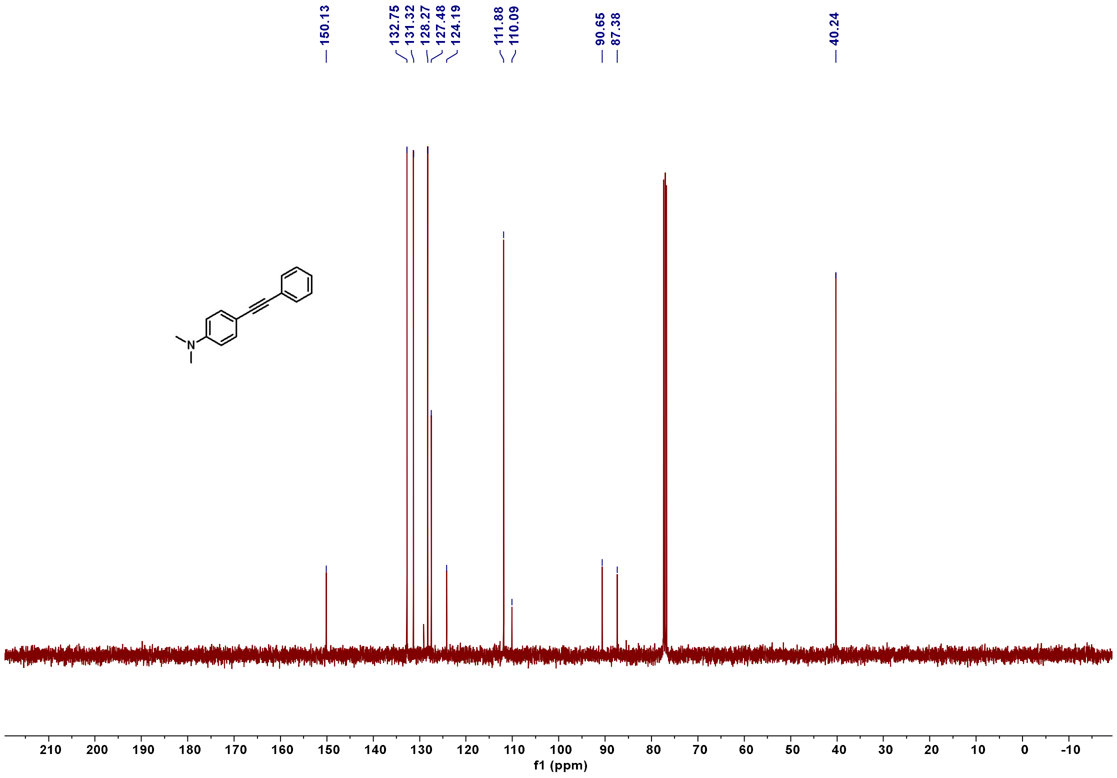

^{13}C NMR (101 MHz, CDCl$_3$) spectrum of 3j

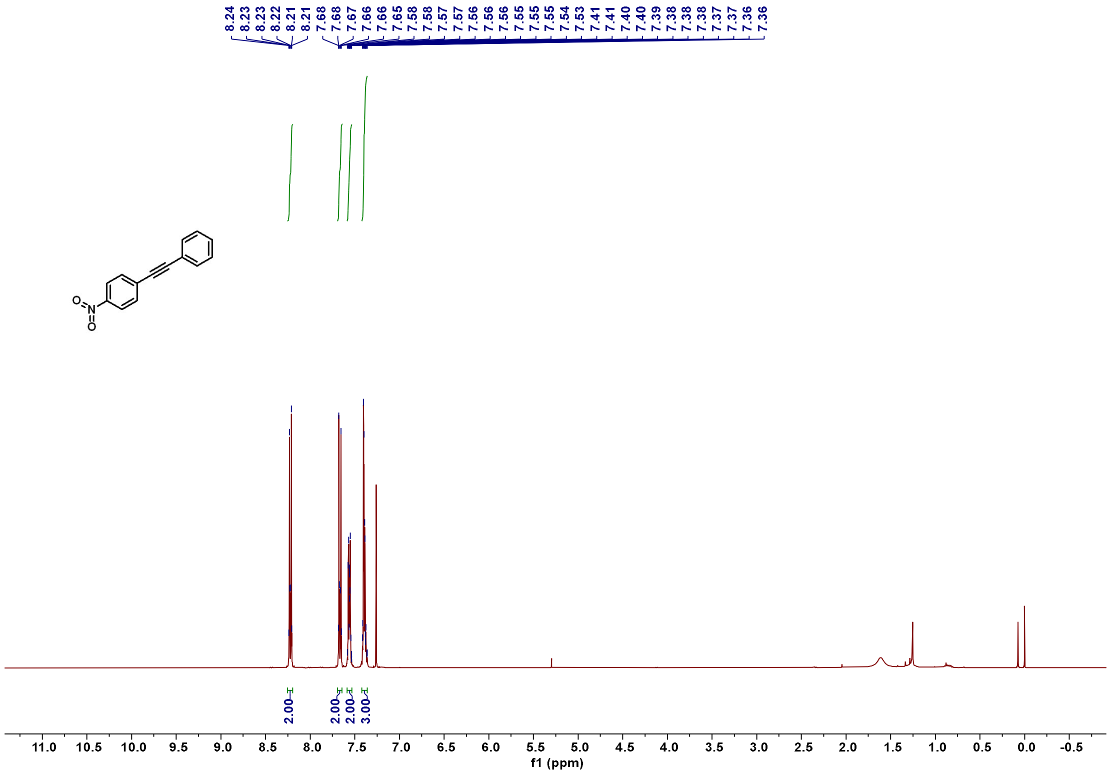

^{1}H NMR (400 MHz, CDCl3) spectrum of 3k

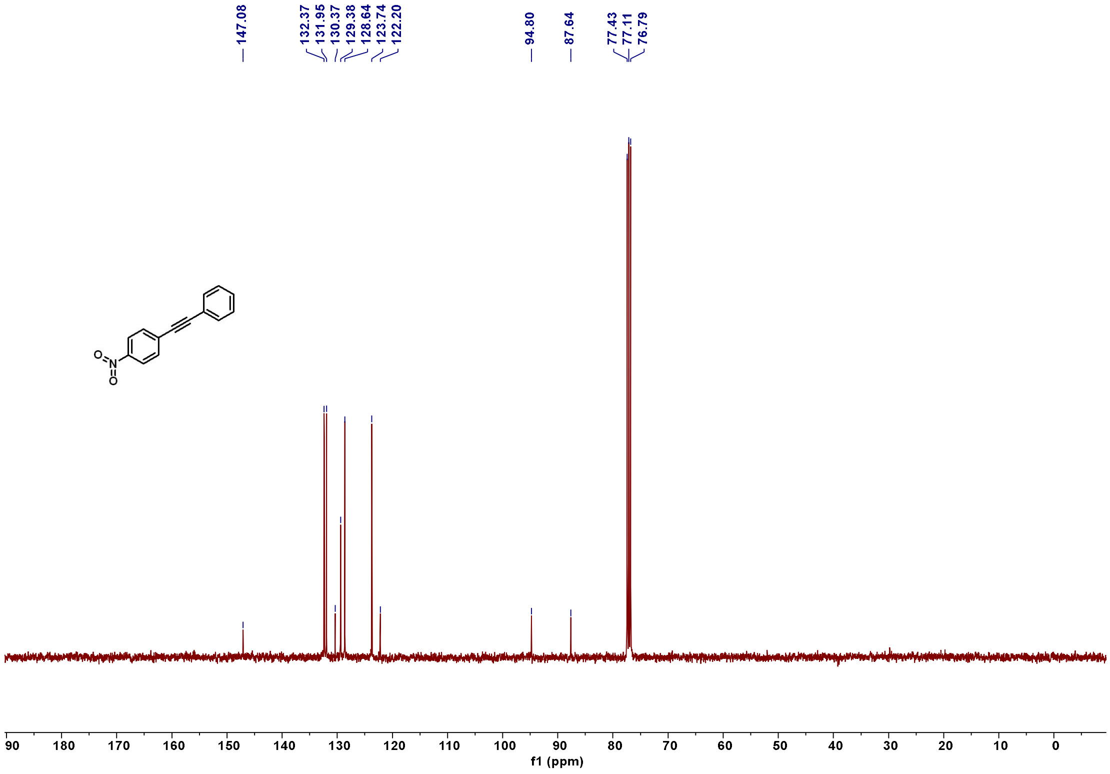

^{13}C NMR (101 MHz, CDCl3) spectrum of 3k

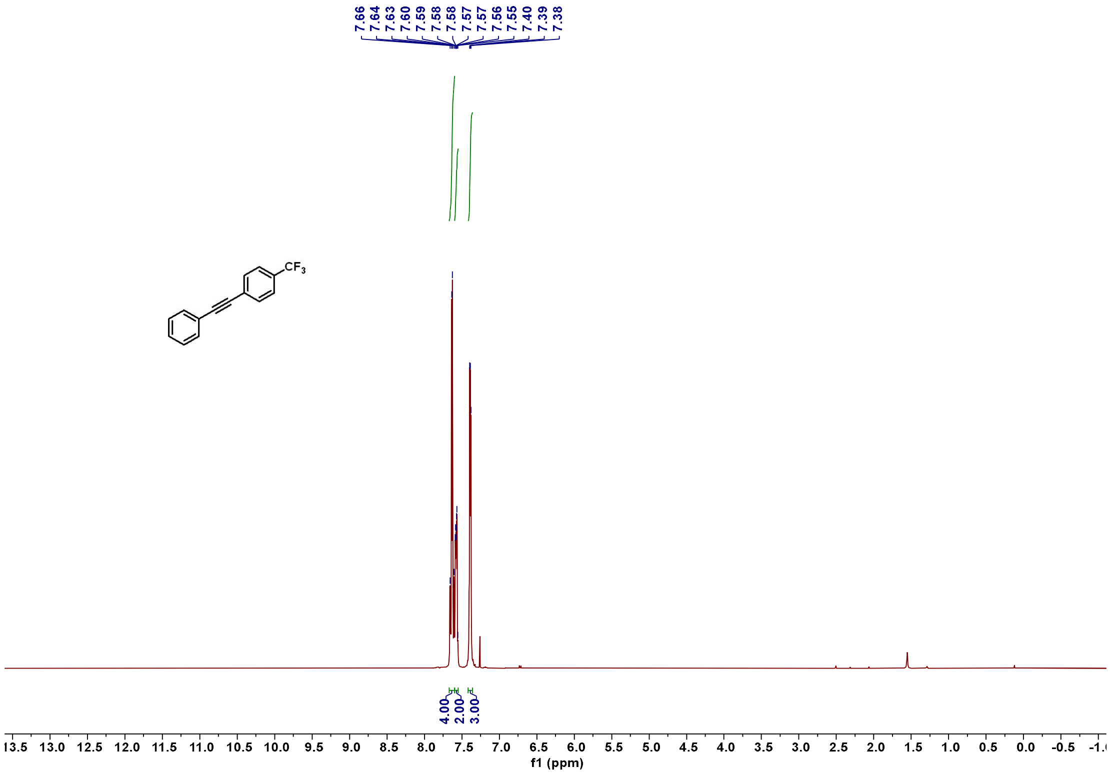

^{1}H NMR (400 MHz, $CDCl_3$) spectrum of 3l

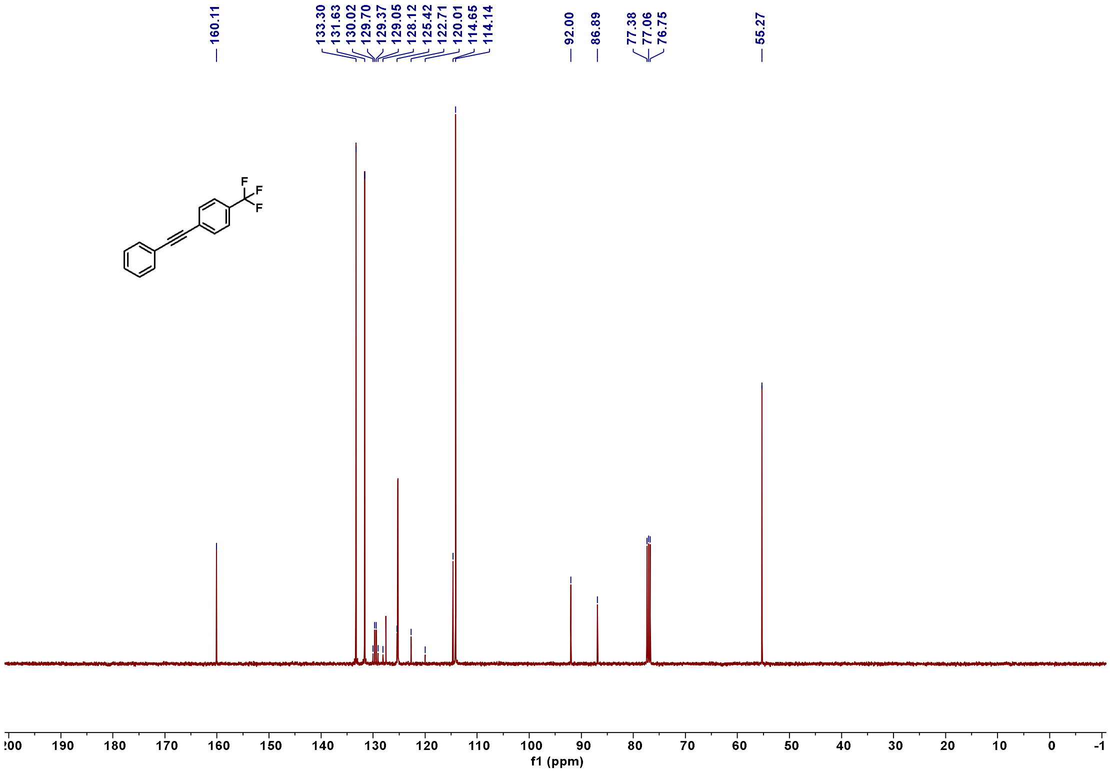

^{13}C NMR (101 MHz, $CDCl_3$) spectrum of 3l

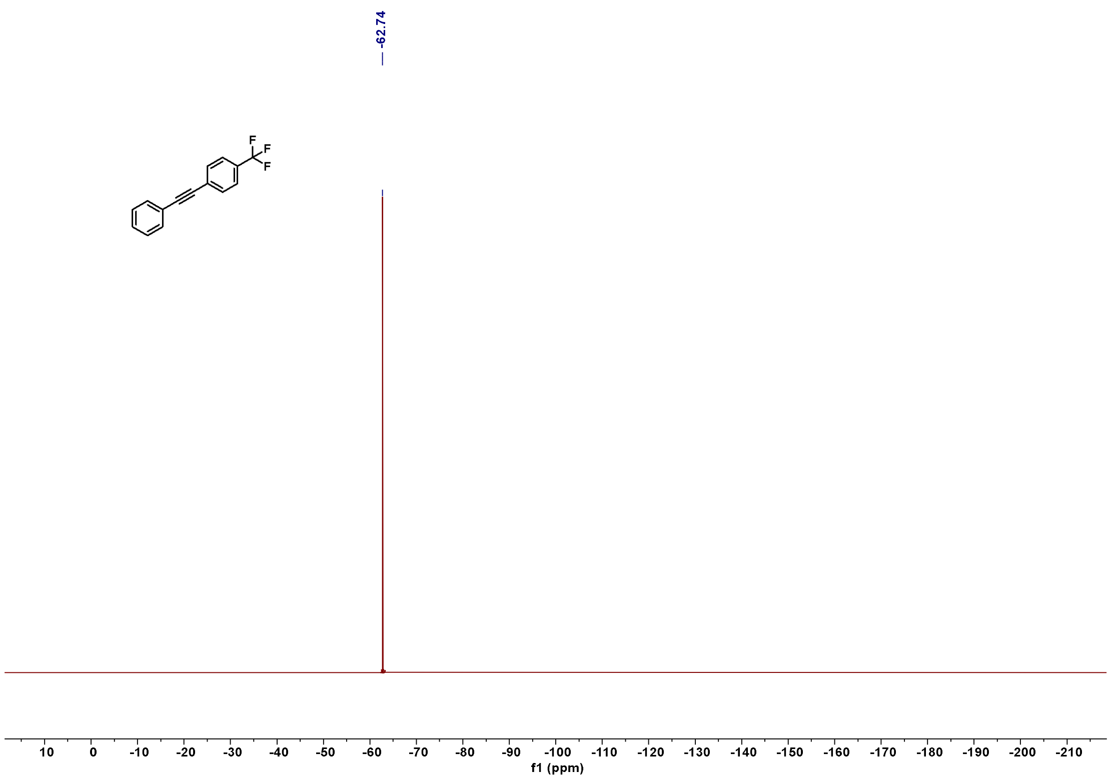

^{19}F NMR (376 MHz, $CDCl_3$) spectrum of 3l

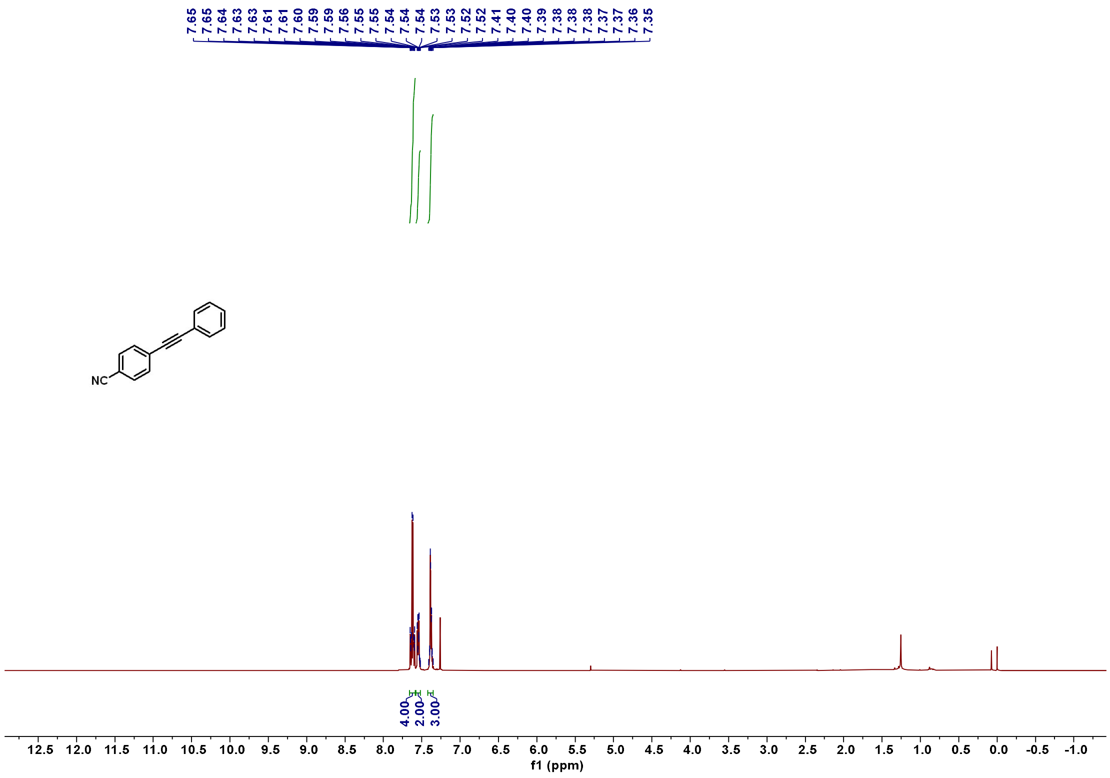

^{1}H NMR (400 MHz, $CDCl_3$) spectrum of 3m

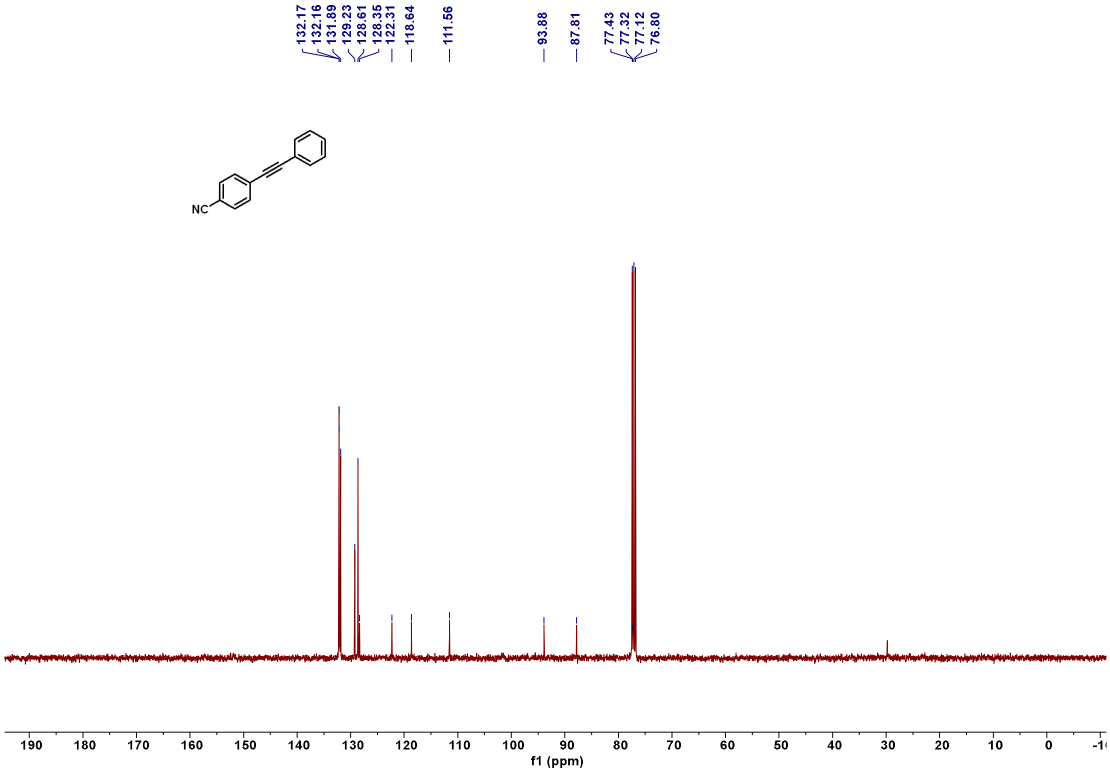

^{13}C NMR (101 MHz, $CDCl_3$) spectrum of 3m

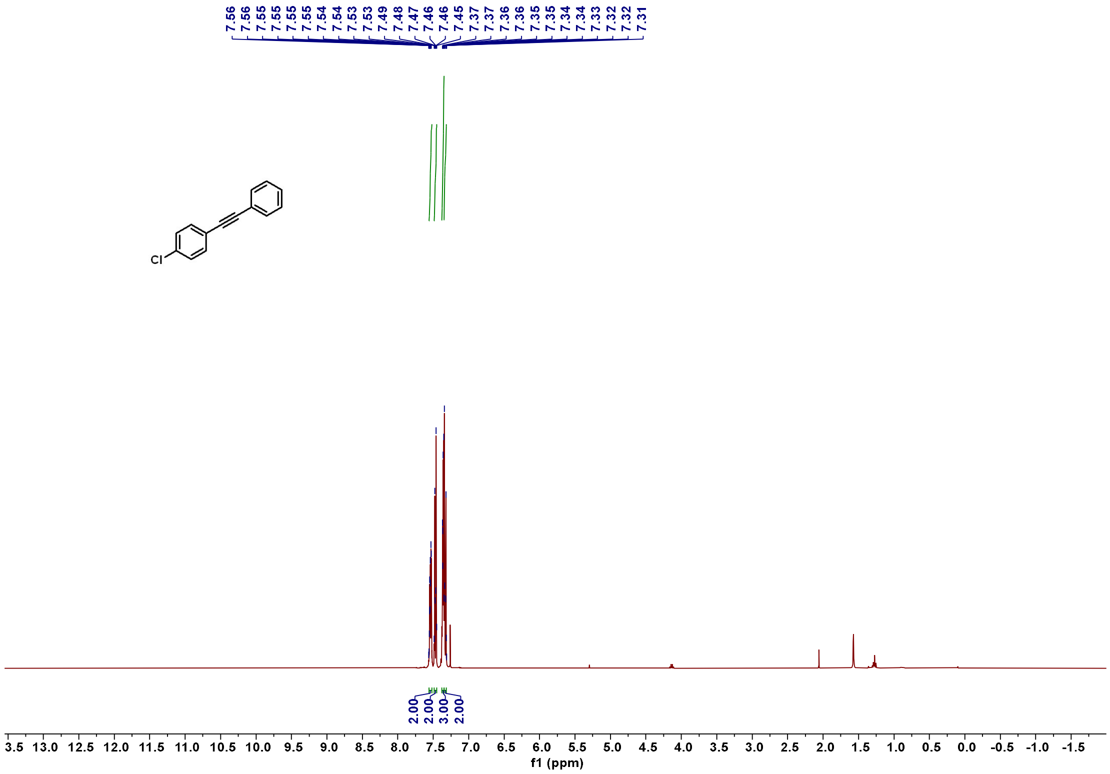

^{1}H NMR (400 MHz, CDCl3) spectrum of 3n

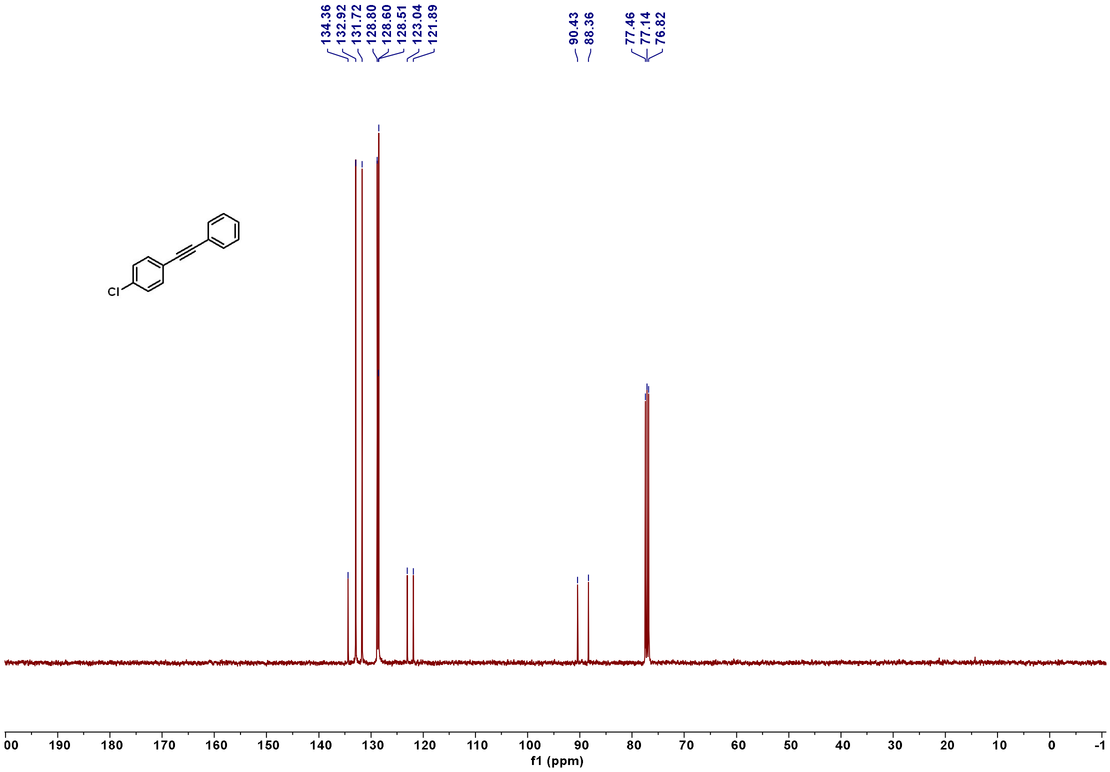

^{13}C NMR (101 MHz, CDCl3) spectrum of 3n

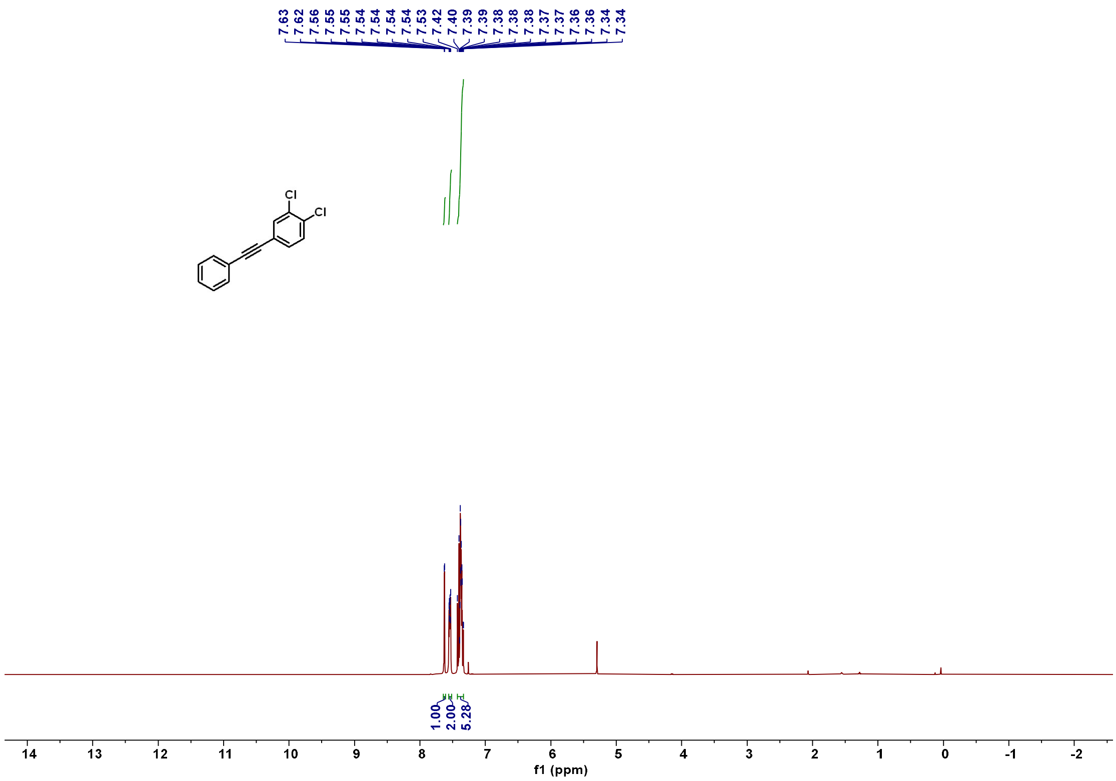

^{1}H NMR (400 MHz, $CDCl_3$) spectrum of 3o

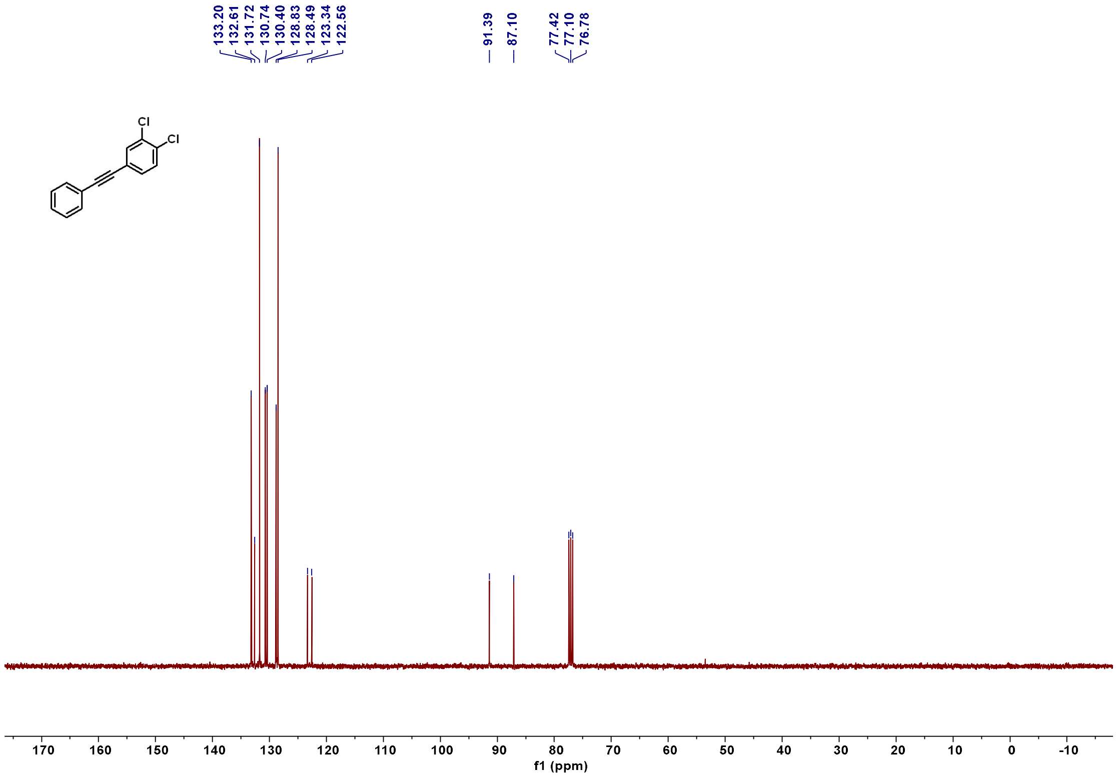

^{13}C NMR (101 MHz, $CDCl_3$) spectrum of 3o

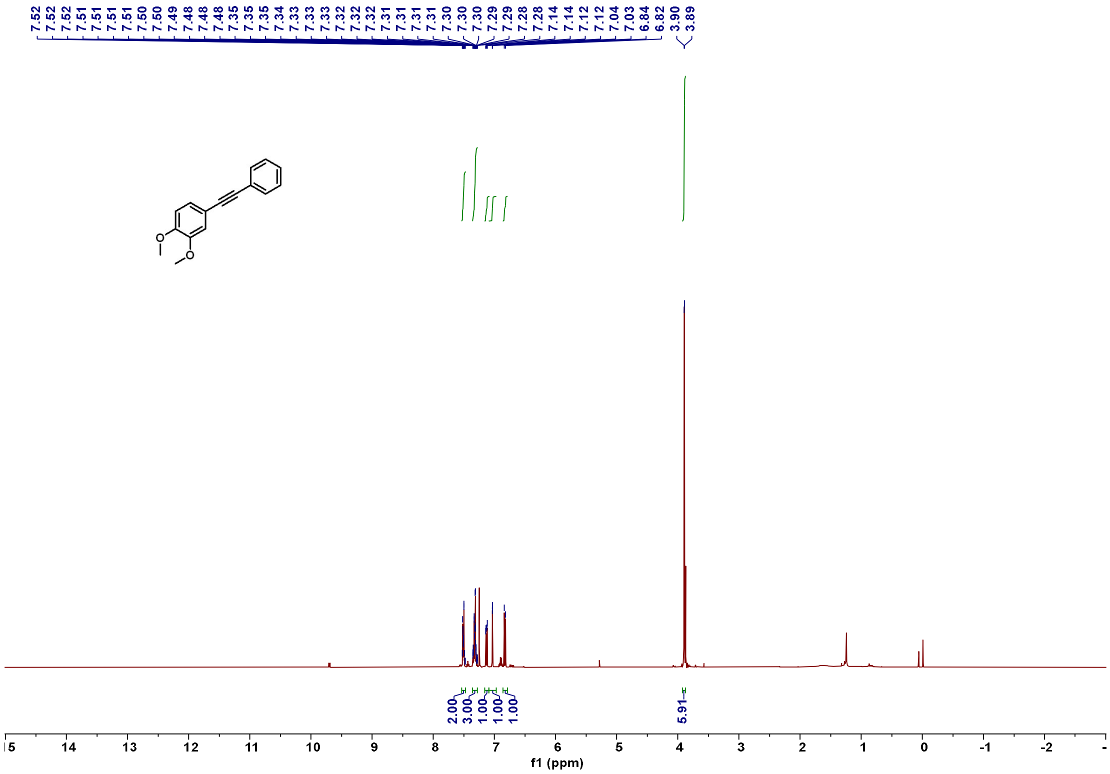

^{1}H NMR (400 MHz, CDCl₃) spectrum of 3p

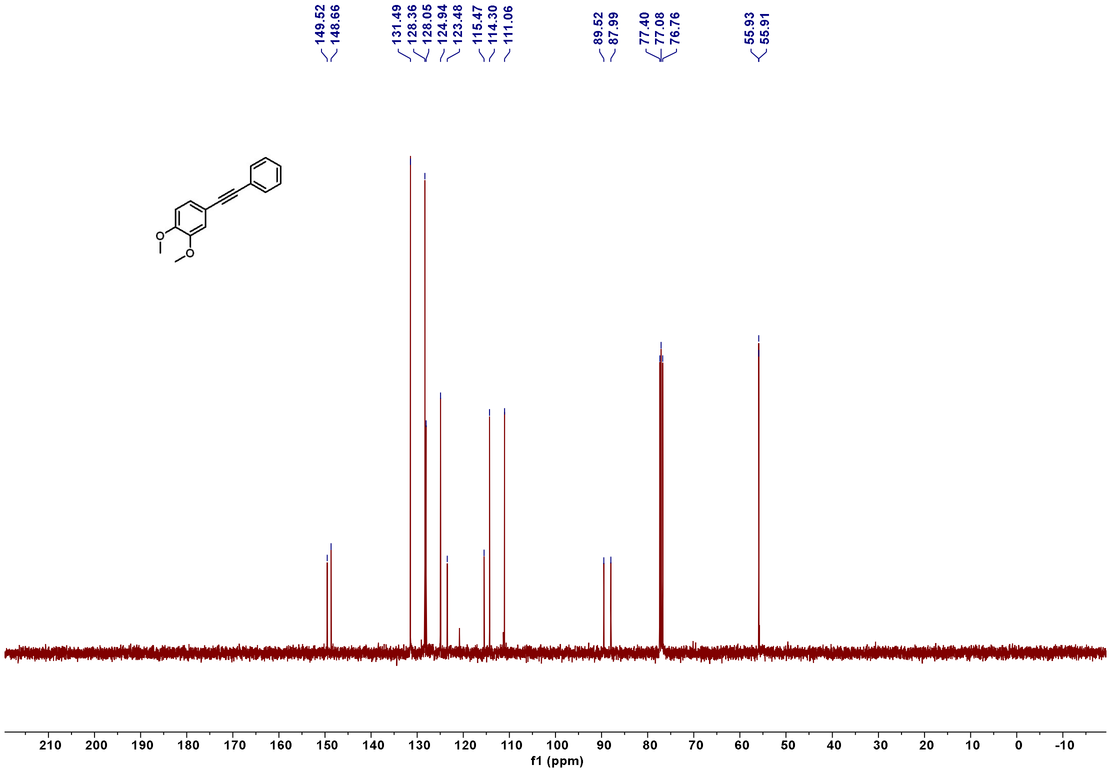

^{13}C NMR (101 MHz, CDCl₃) spectrum of 3p

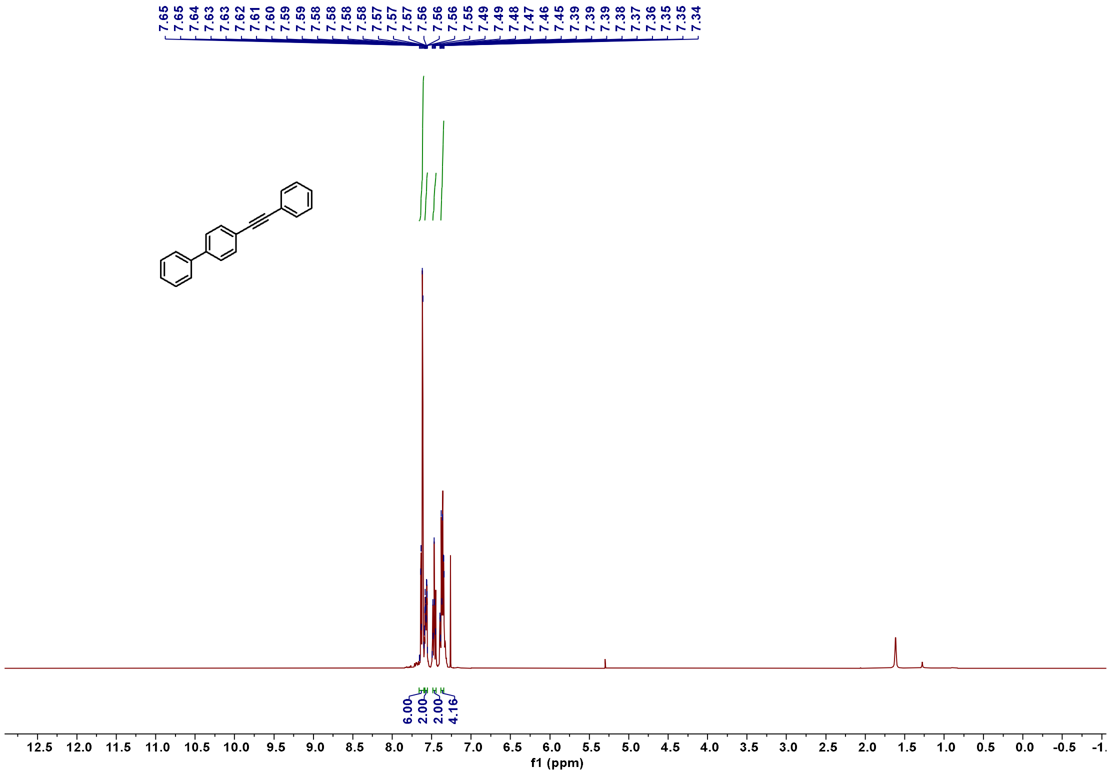

^{1}H NMR (400 MHz, CDCl3) spectrum of 3q

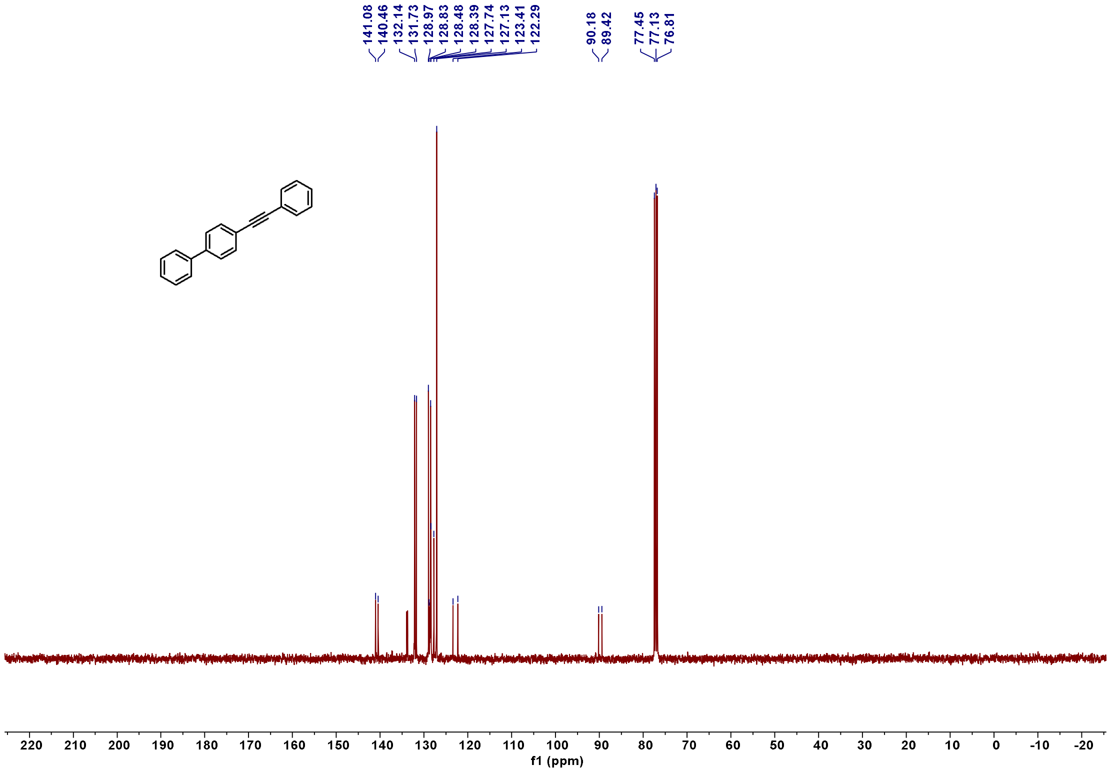

^{13}C NMR (101 MHz, CDCl3) spectrum of 3q

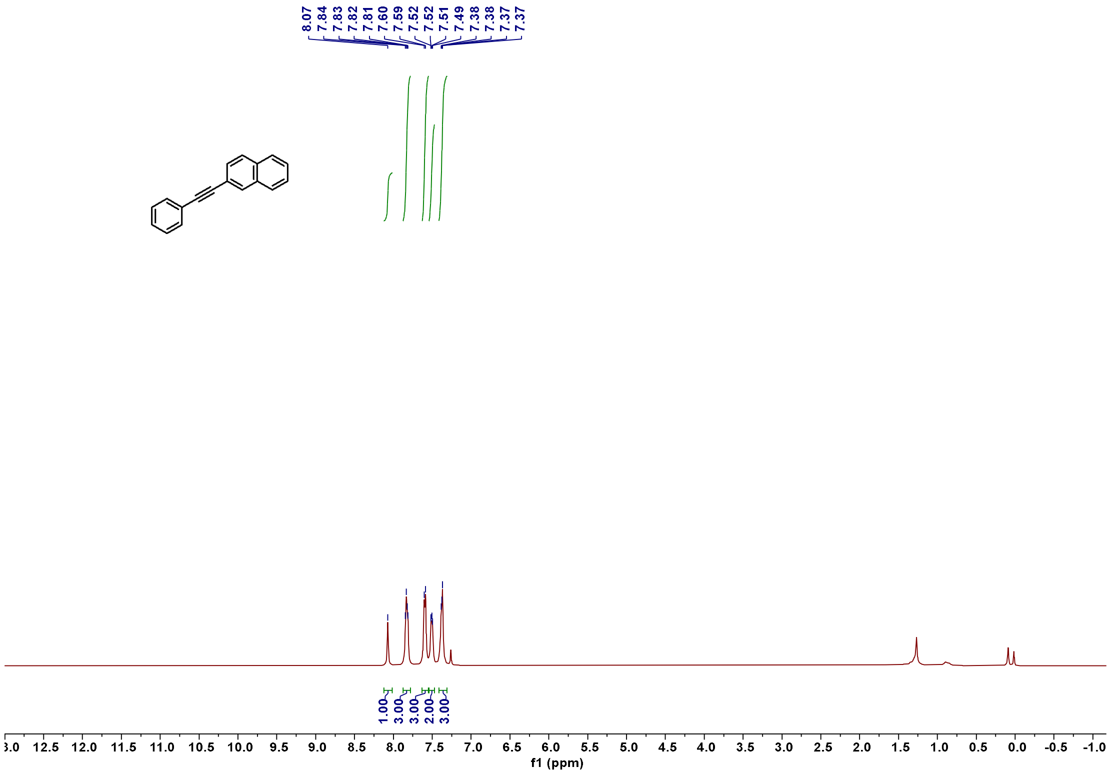

^{1}H NMR (400 MHz, CDCl$_3$) spectrum of 3r

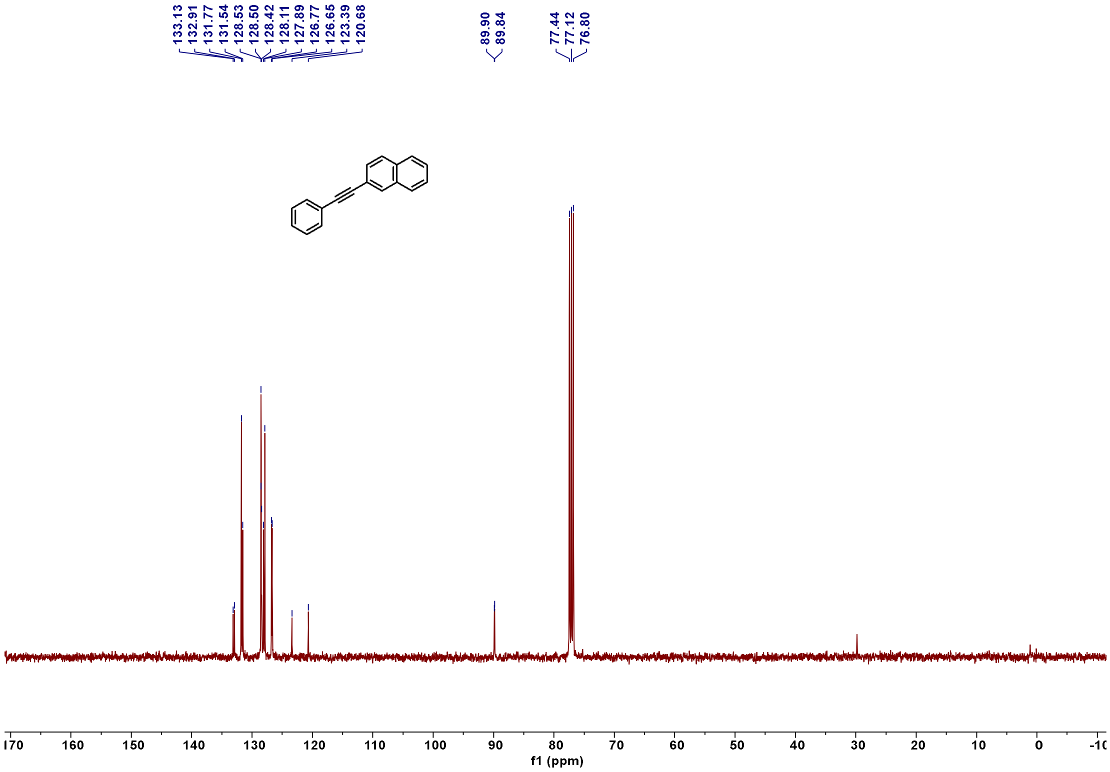

^{13}C NMR (101 MHz, CDCl$_3$) spectrum of 3r

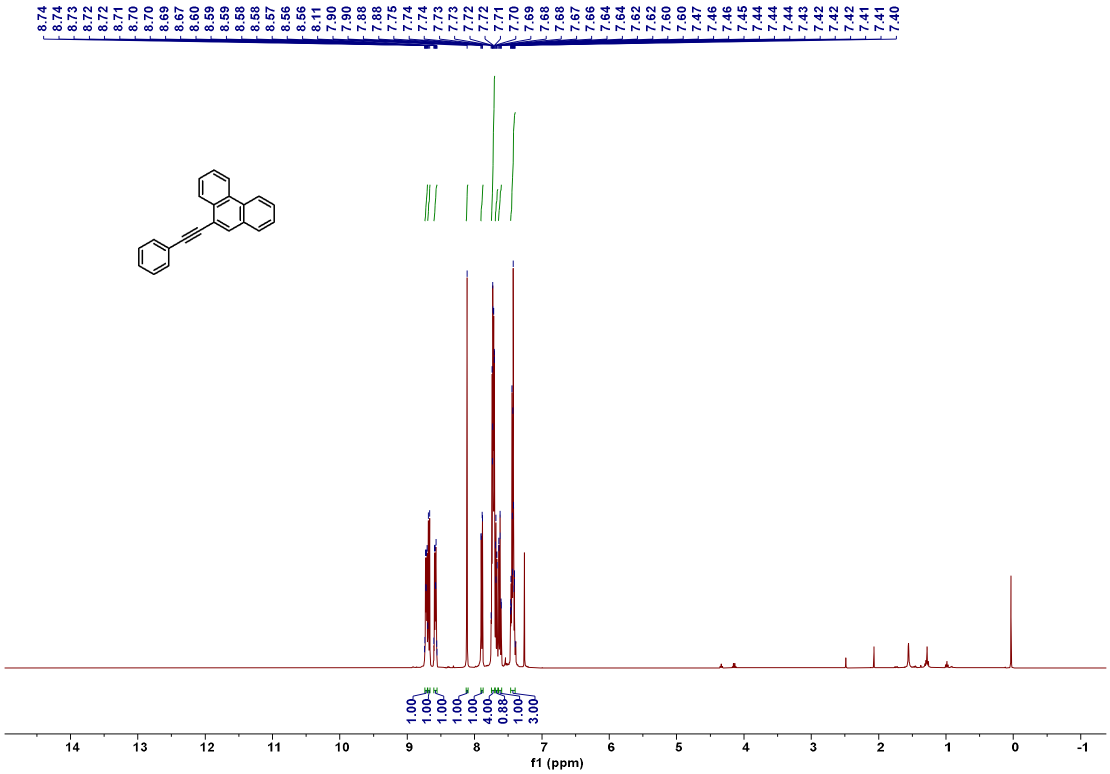

^{1}H NMR (400 MHz, $CDCl_3$) spectrum of 3s

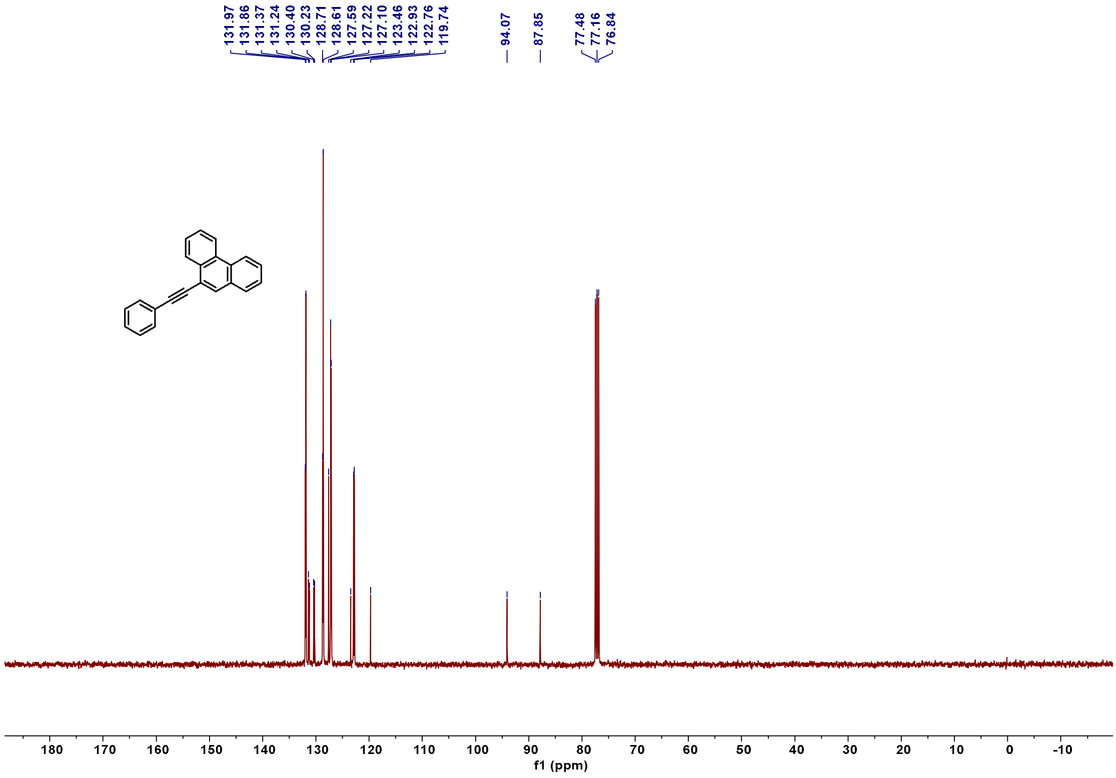

^{13}C NMR (101 MHz, $CDCl_3$) spectrum of 3s

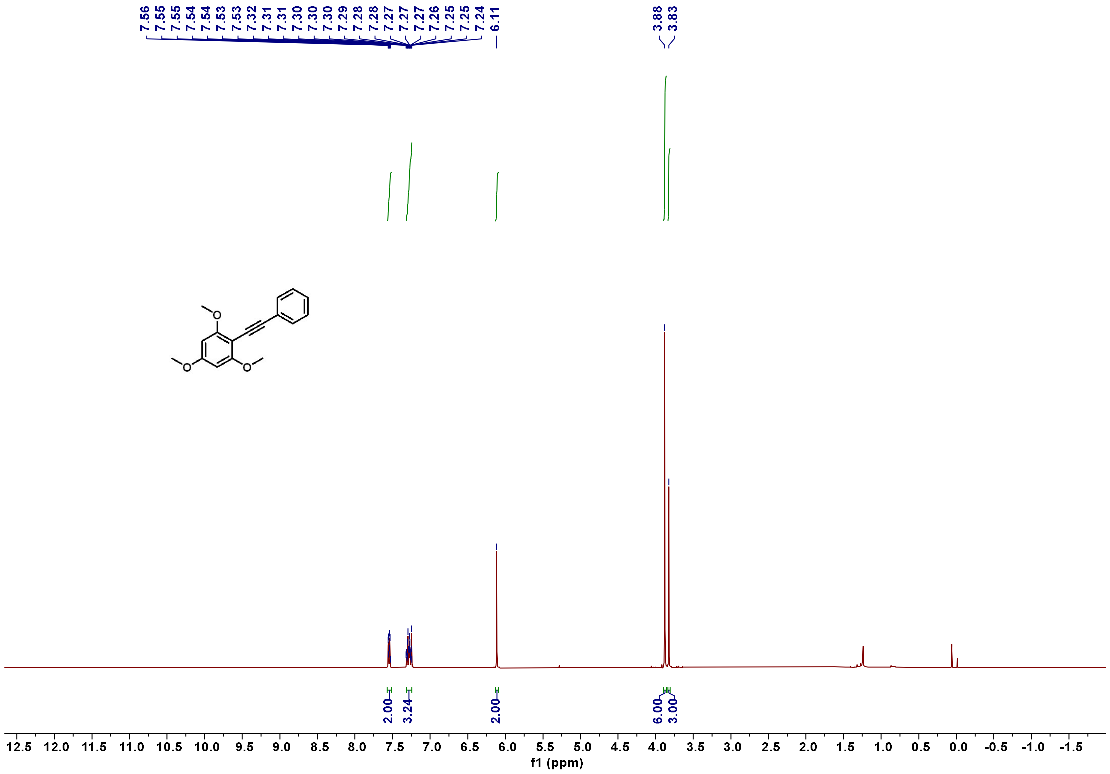

^{1}H NMR (400 MHz, CDCl3) spectrum of 3t

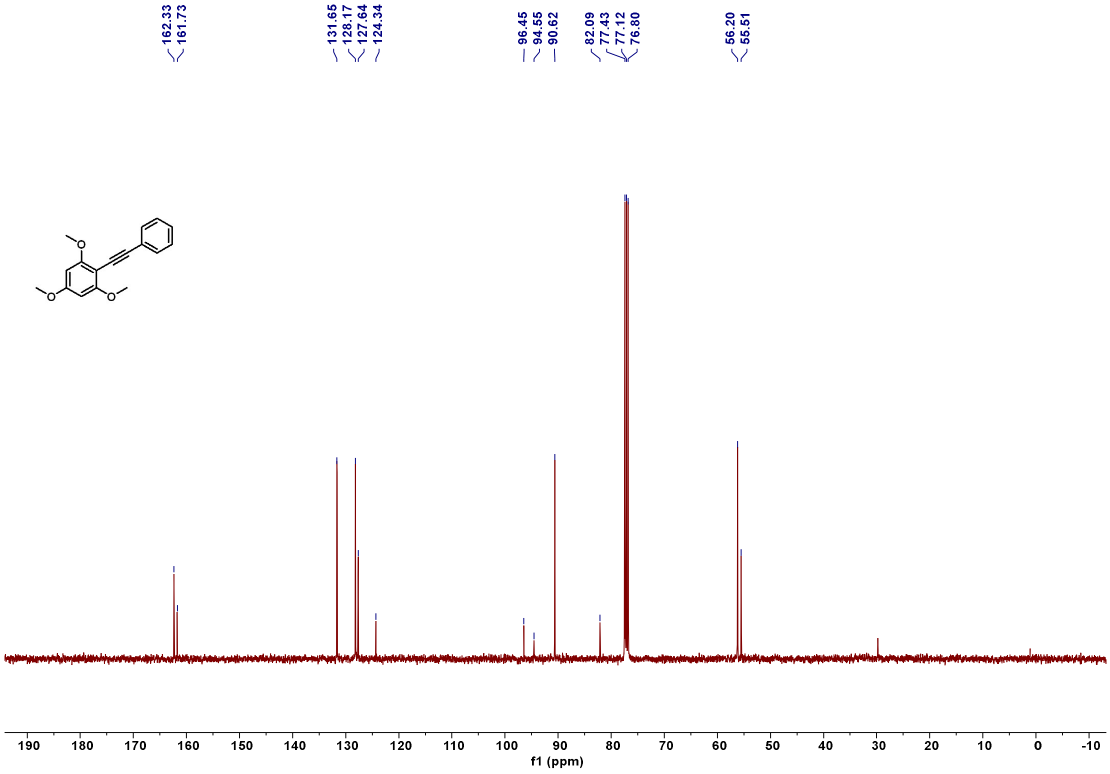

^{13}C NMR (101 MHz, CDCl3) spectrum of 3t

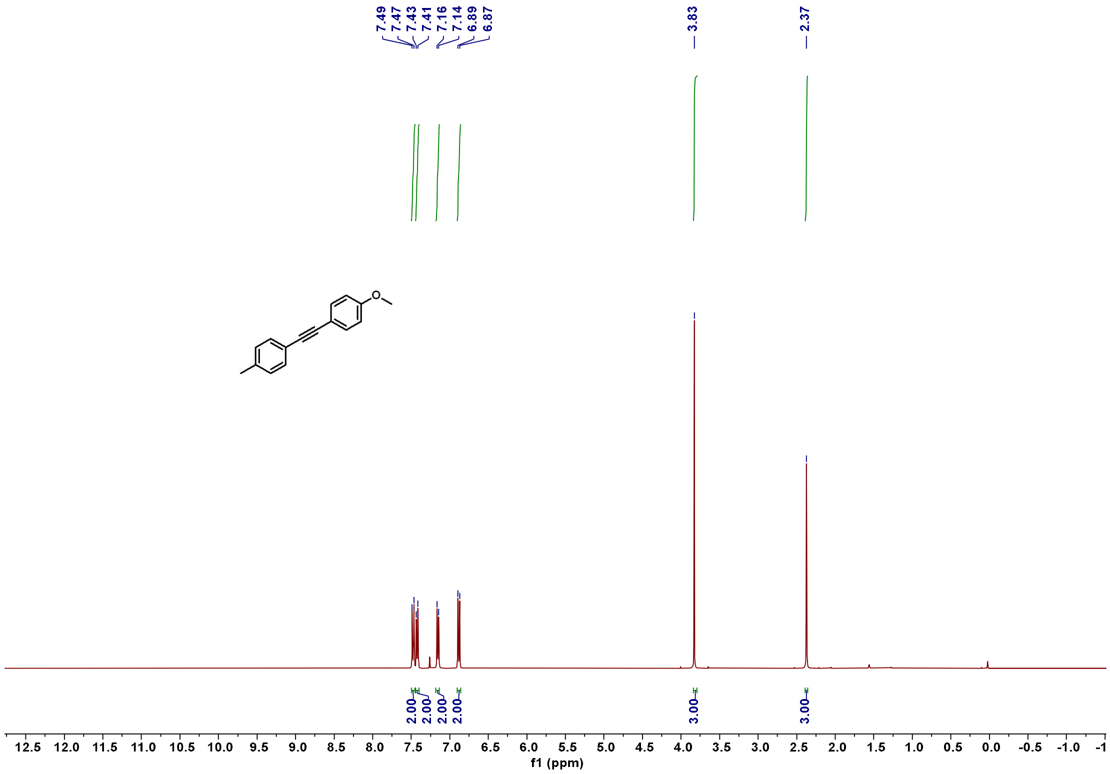

^{1}H NMR (400 MHz, CDCl3) spectrum of 3u

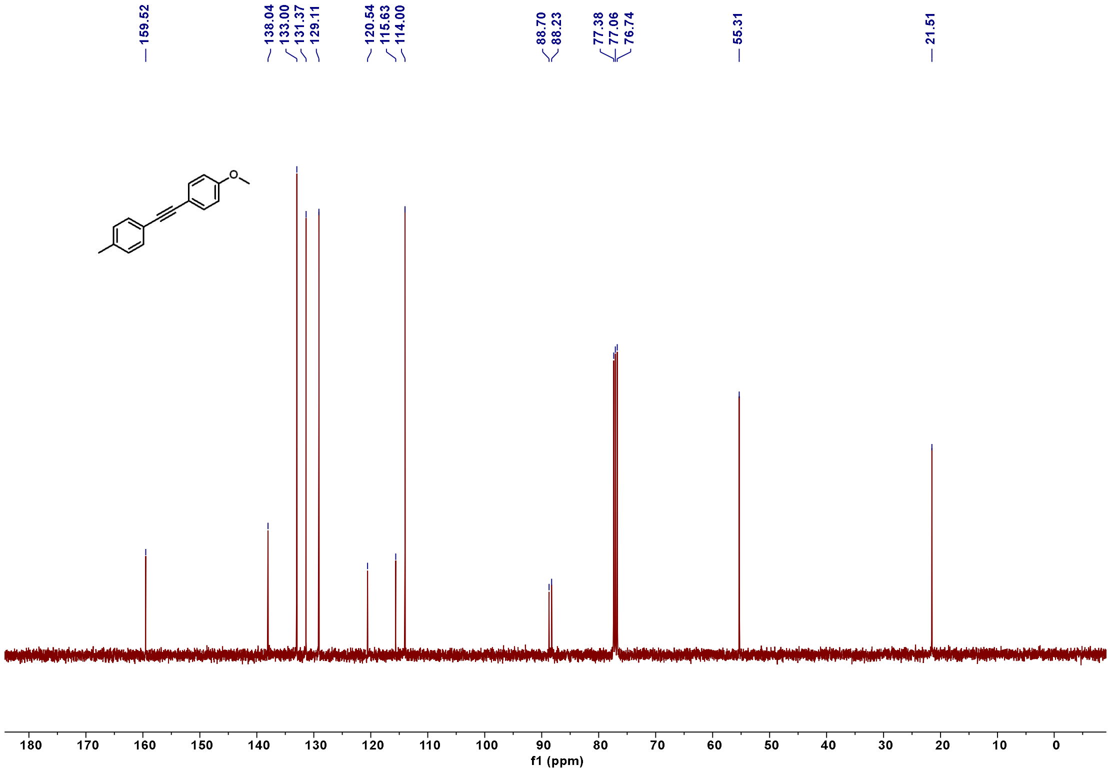

^{13}C NMR (101 MHz, CDCl3) spectrum of 3u

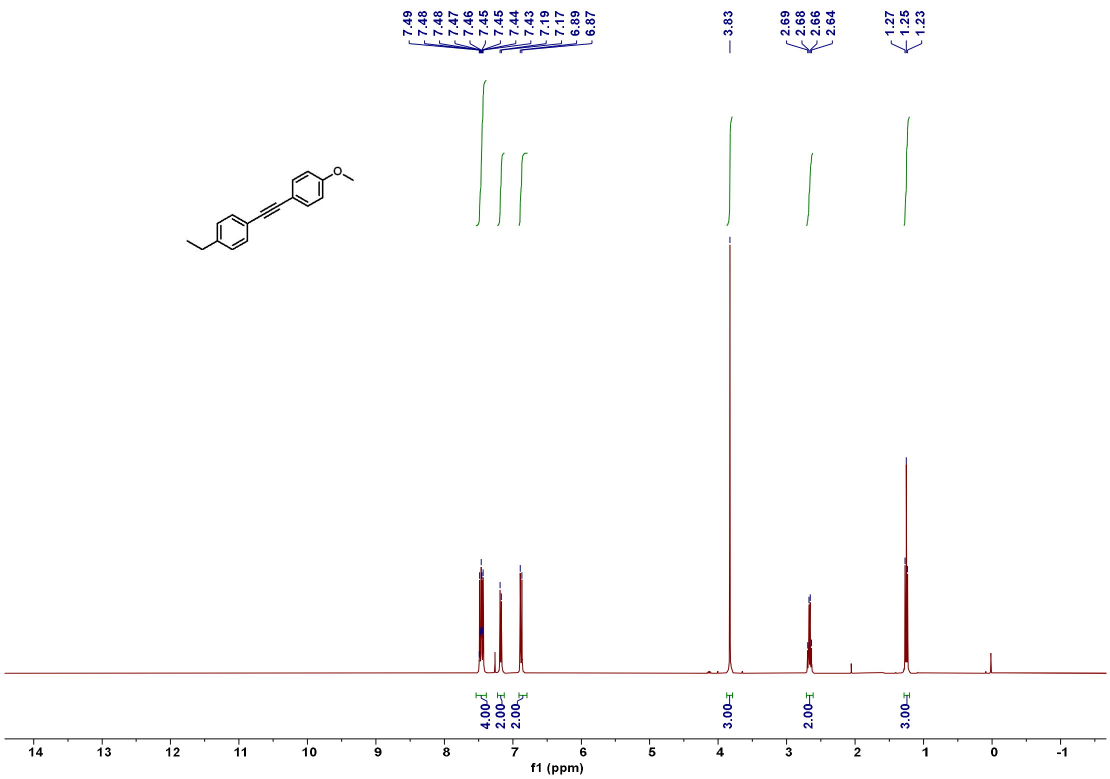

^{1}H NMR (400 MHz, CDCl$_3$) spectrum of 3v

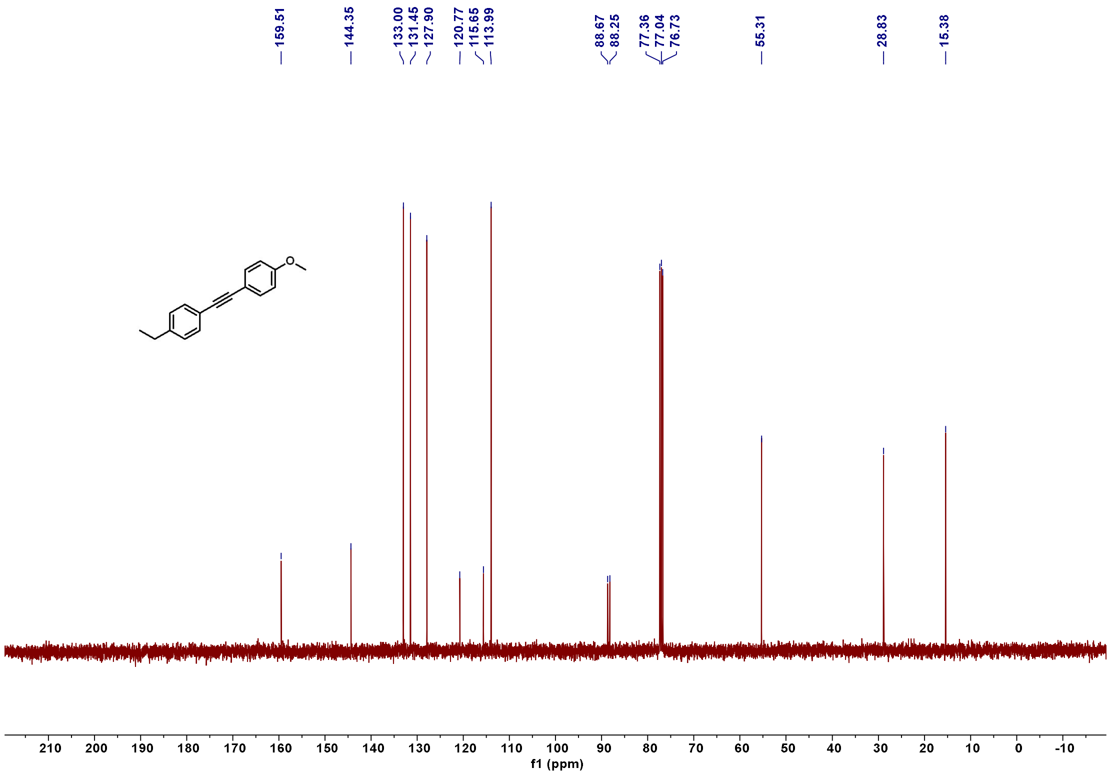

^{13}C NMR (101 MHz, CDCl$_3$) spectrum of 3v

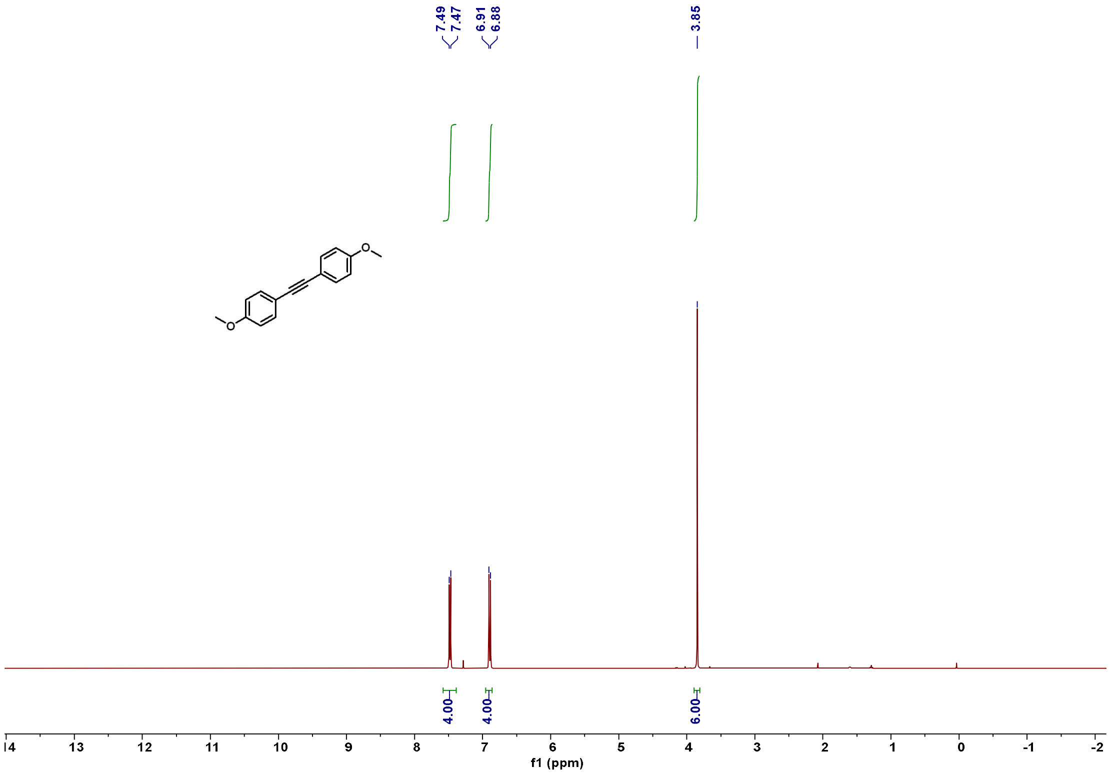

^{1}H NMR (400 MHz, $CDCl_3$) spectrum of 3w

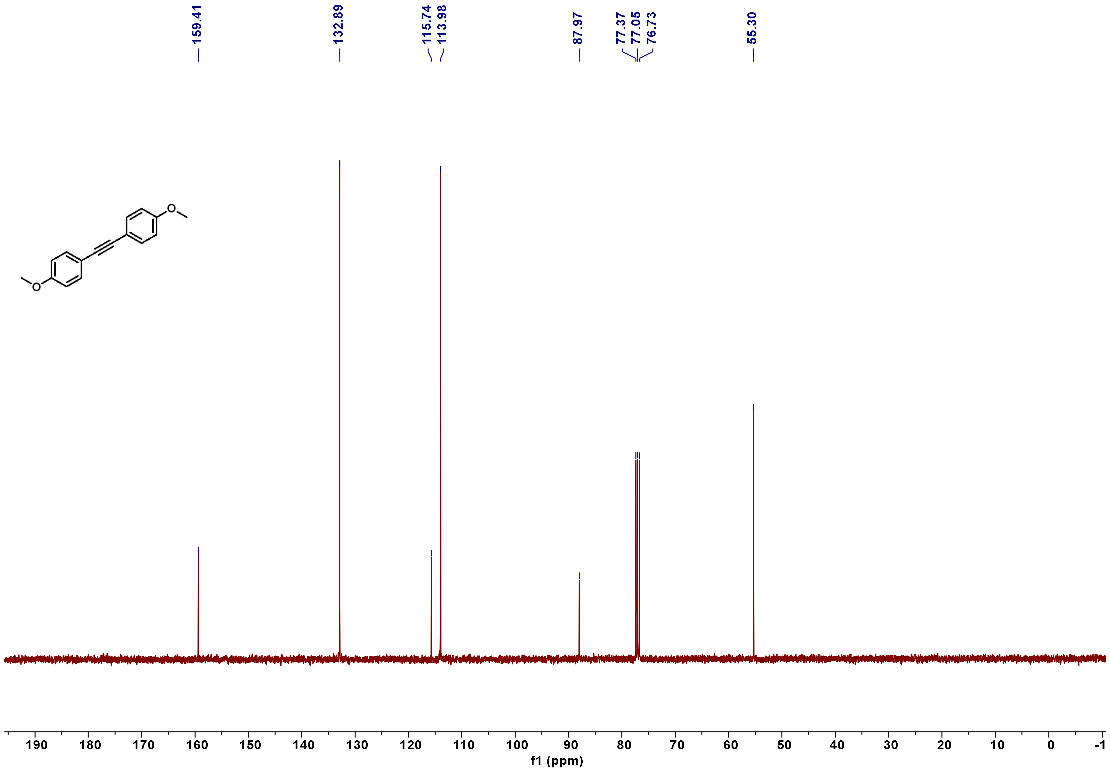

^{13}C NMR (101 MHz, $CDCl_3$) spectrum of 3w

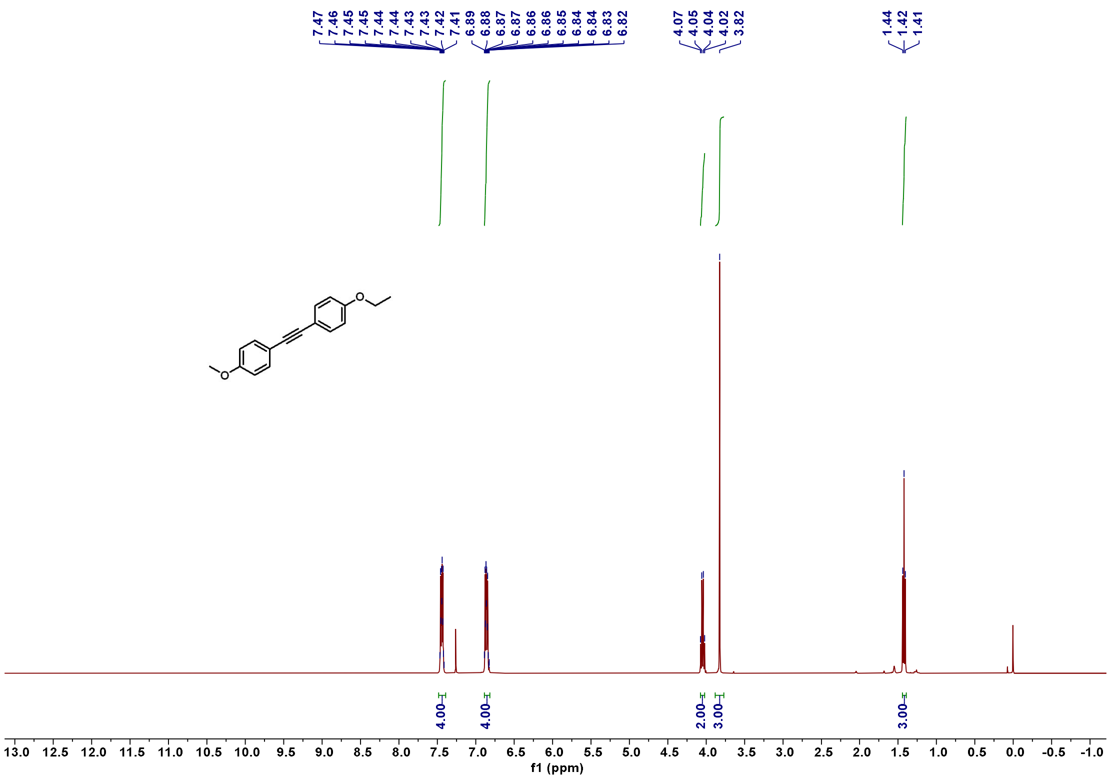

¹H NMR (400 MHz, CDCl₃) spectrum of 3x

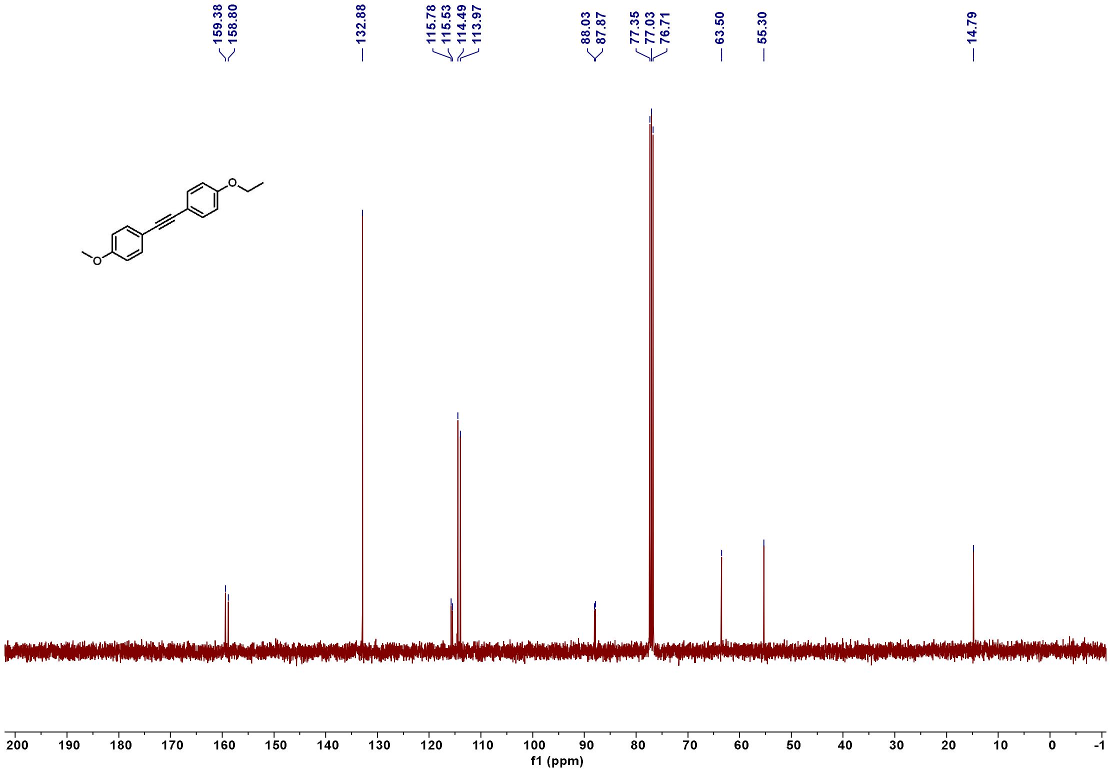

¹³C NMR (101 MHz, CDCl₃) spectrum of 3x

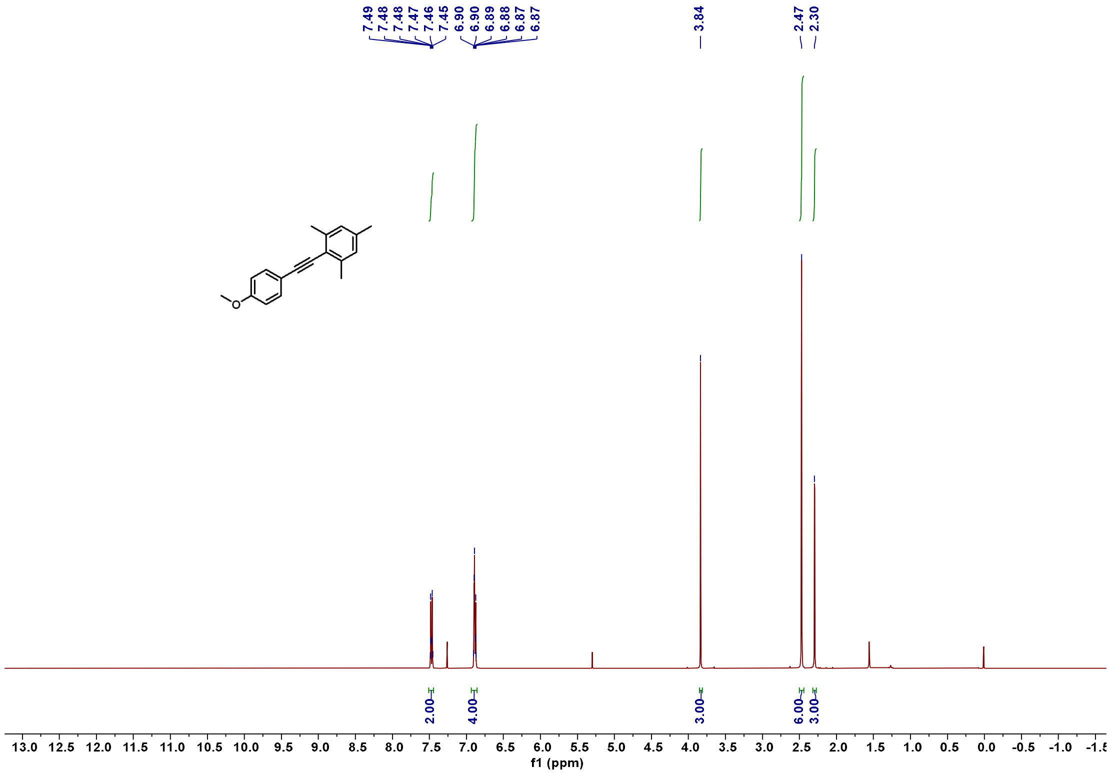

^{1}H NMR (400 MHz, $CDCl_3$) spectrum of 3y

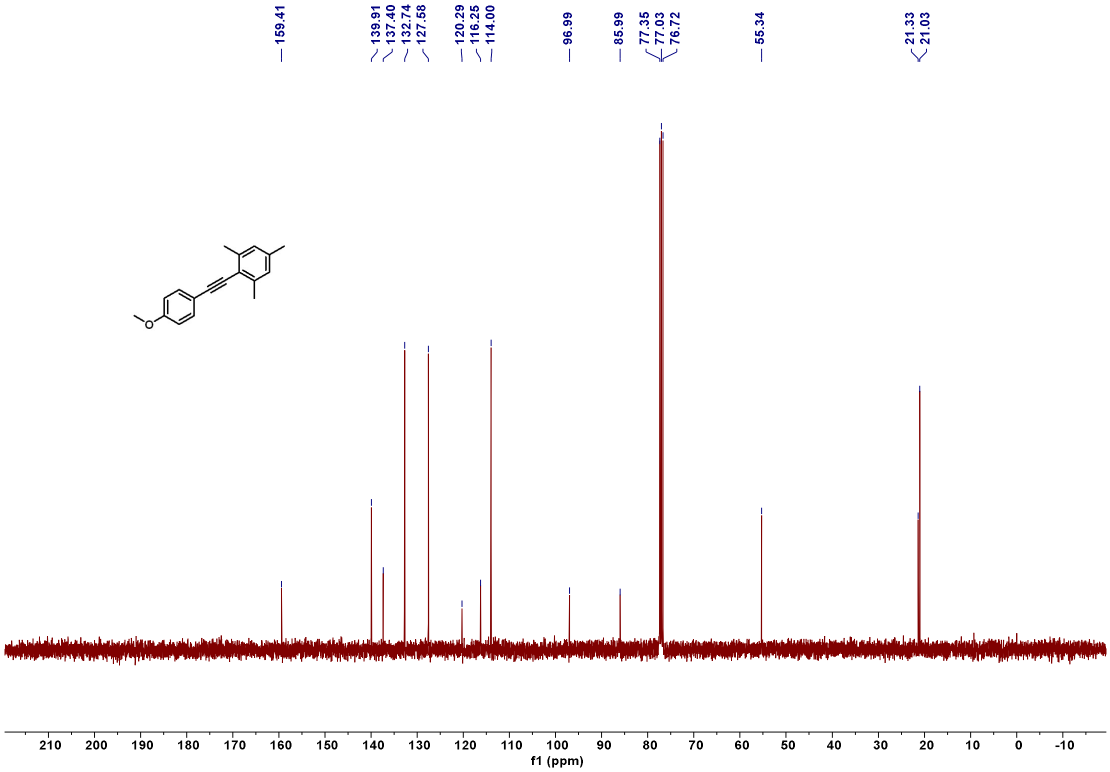

^{13}C NMR (101 MHz, $CDCl_3$) spectrum of 3y

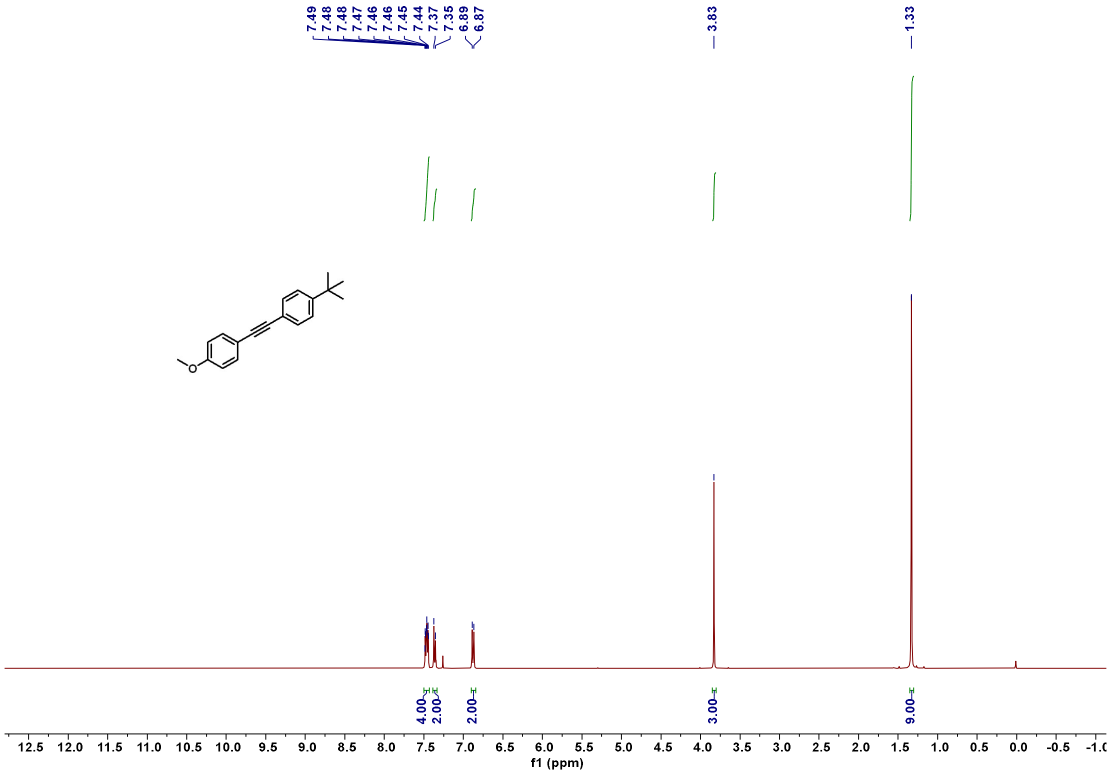

^{1}H NMR (400 MHz, CDCl3) spectrum of 3z

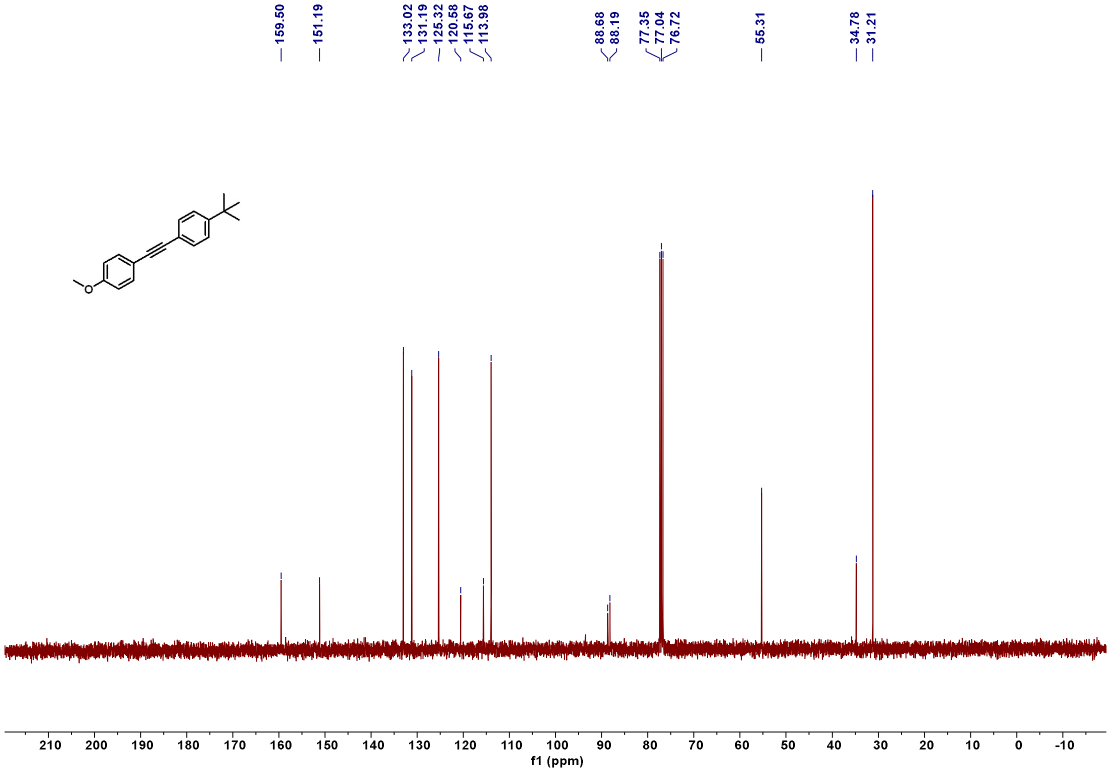

^{13}C NMR (101 MHz, CDCl3) spectrum of 3z

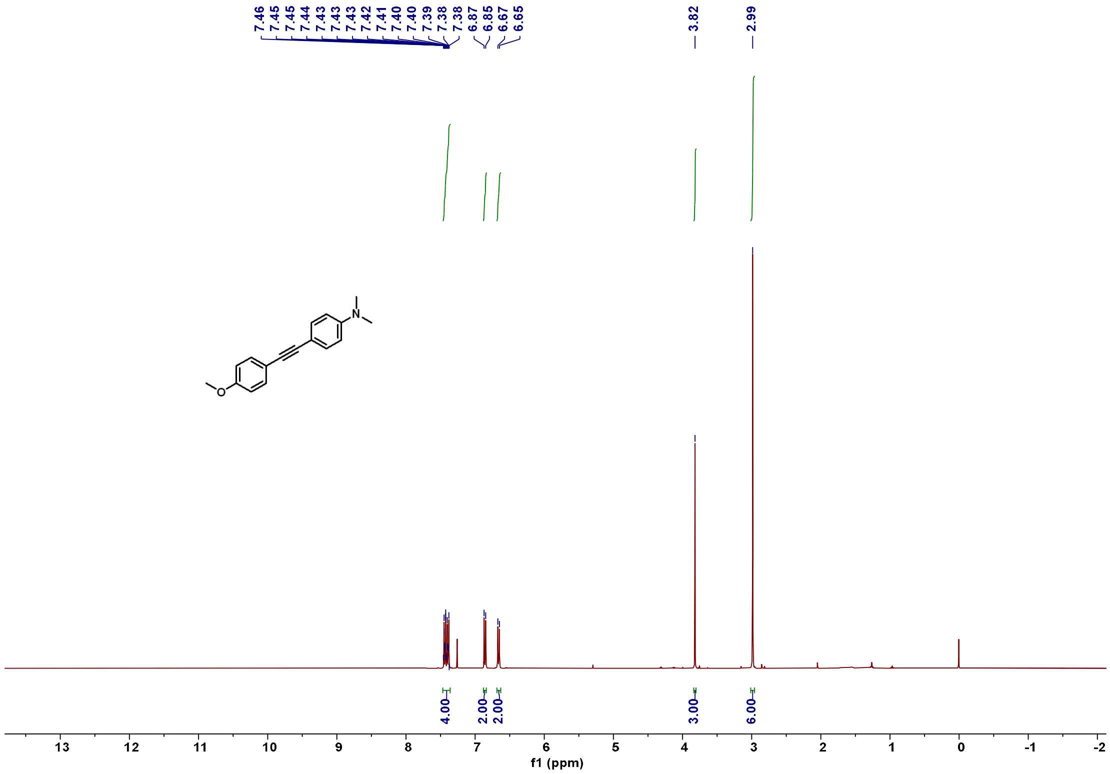

^{1}H NMR (400 MHz, CDCl3) spectrum of 3aa

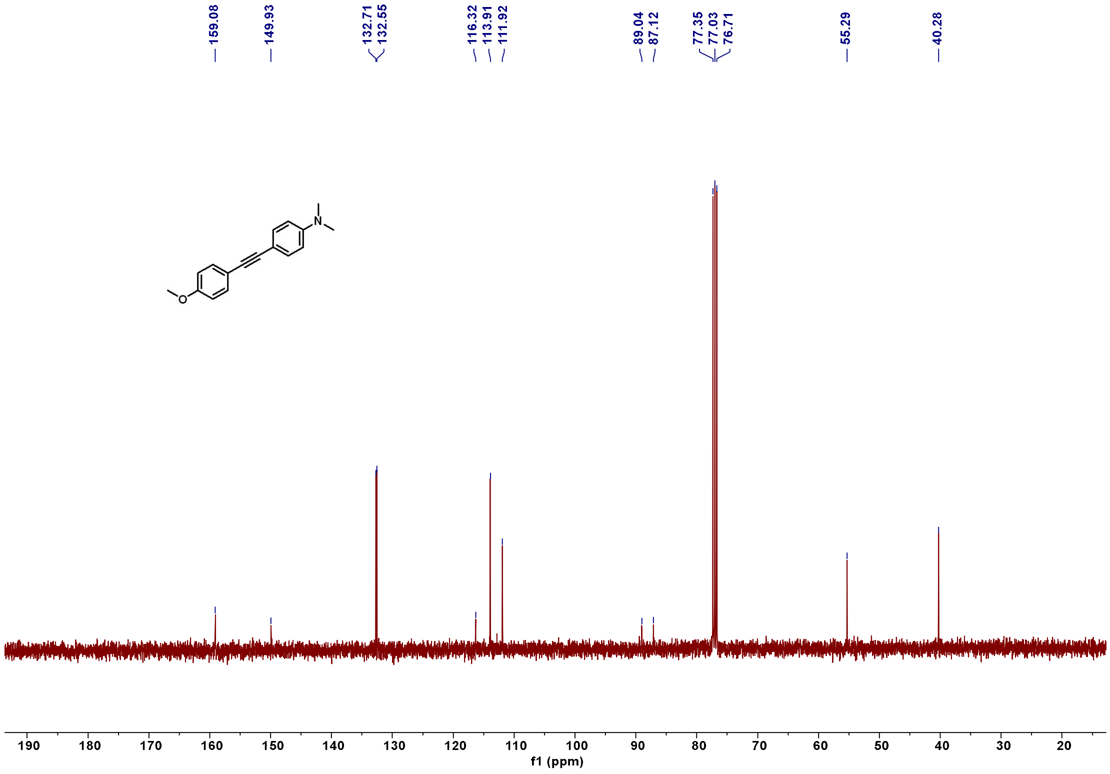

^{13}C NMR (101 MHz, CDCl3) spectrum of 3aa

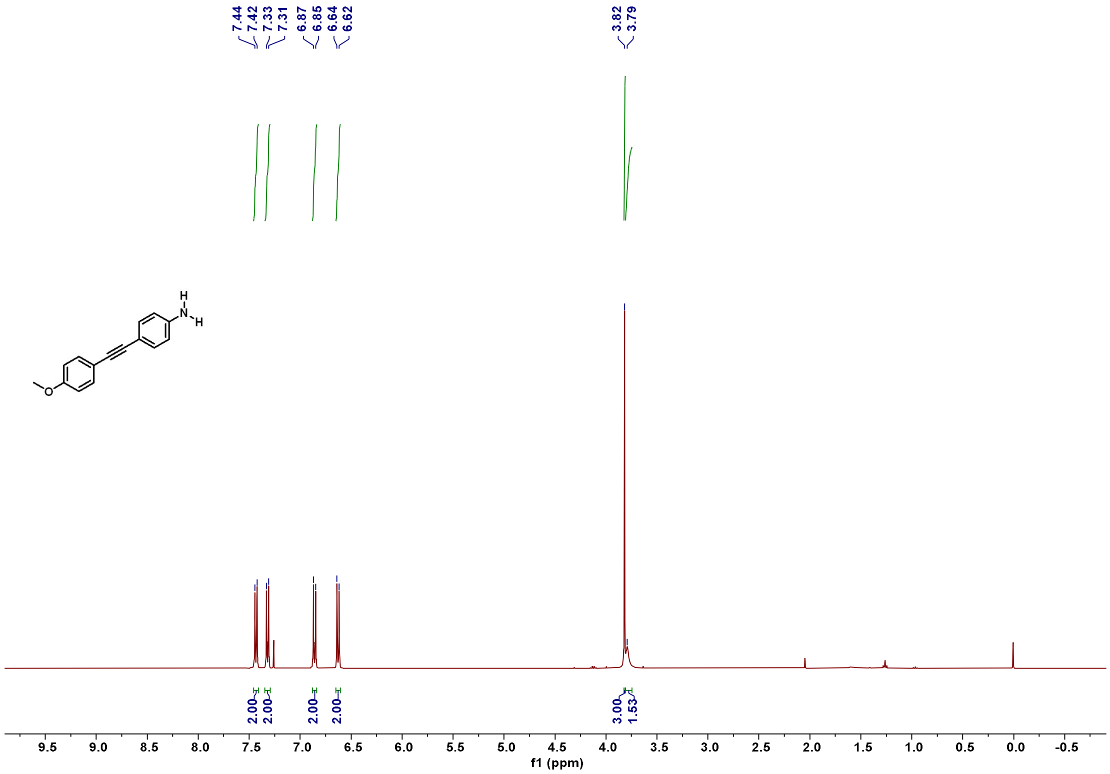

^{1}H NMR (400 MHz, $CDCl_3$) spectrum of 3ab

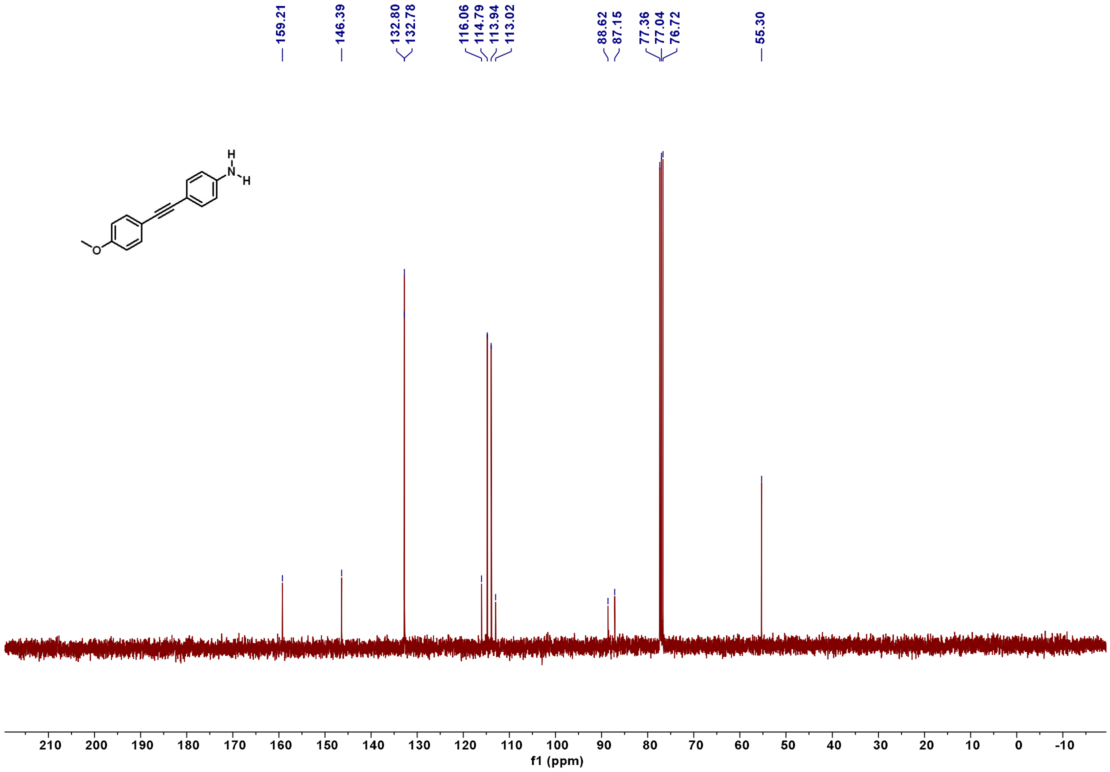

^{13}C NMR (101 MHz, $CDCl_3$) spectrum of 3ab

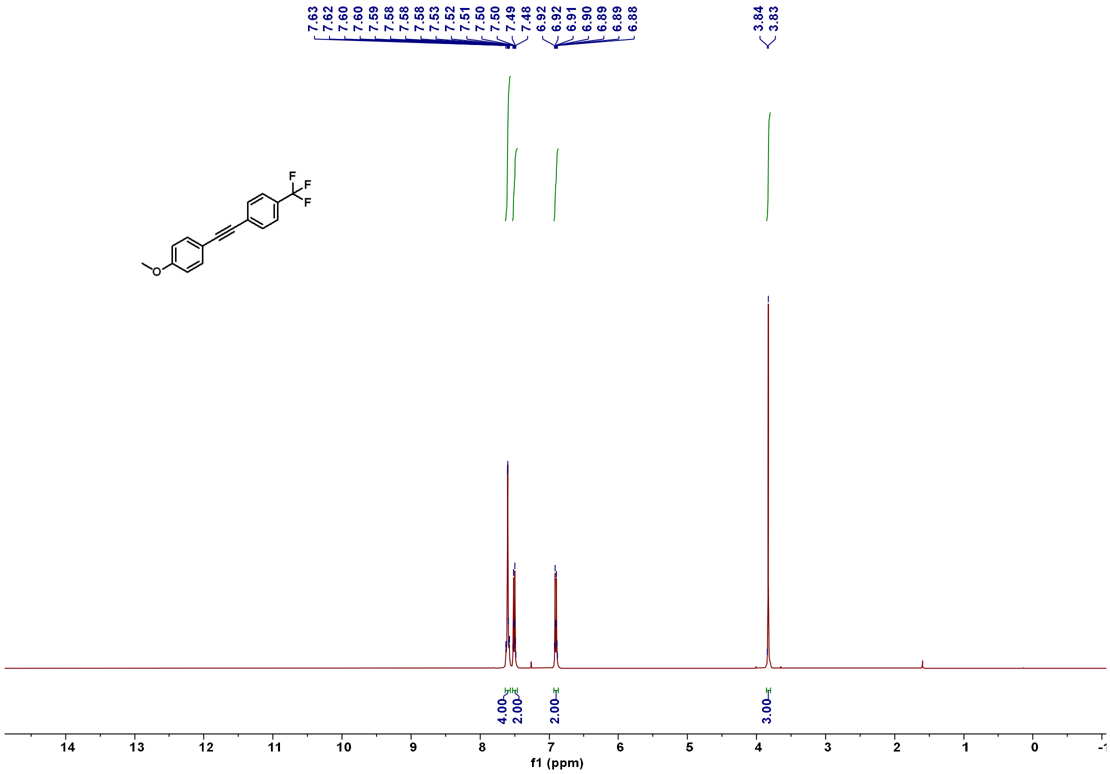

^{1}H NMR (400 MHz, $CDCl_3$) spectrum of 3ac

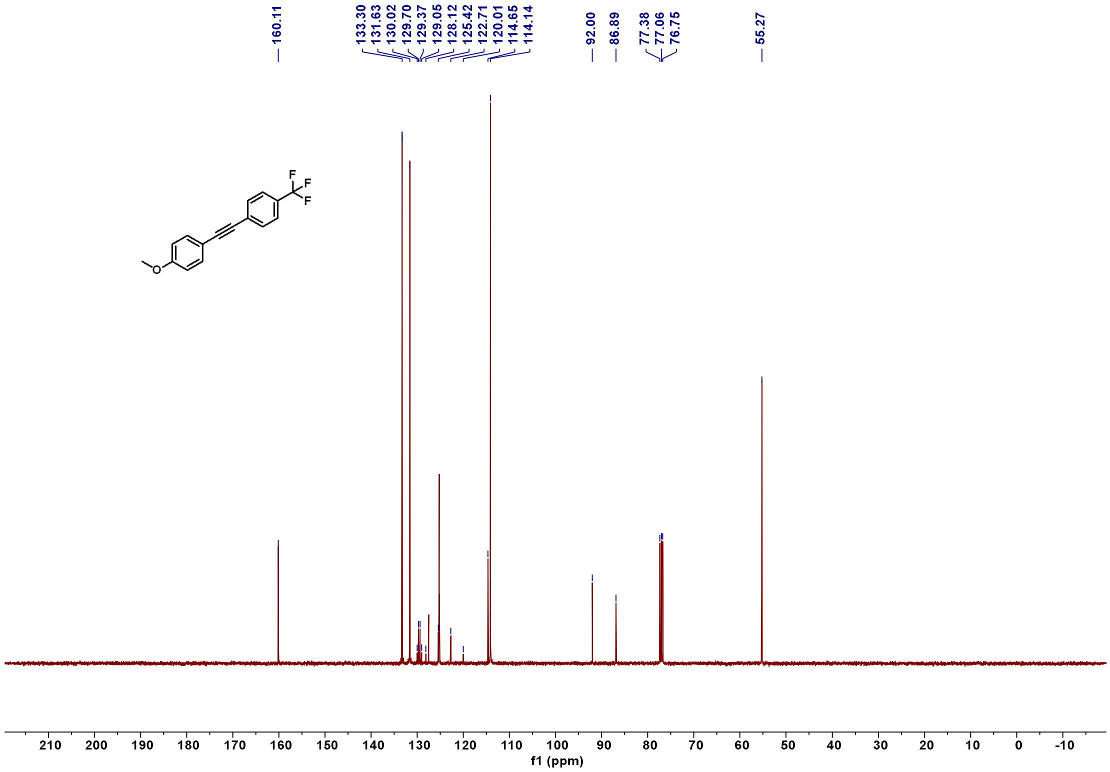

^{13}C NMR (101 MHz, $CDCl_3$) spectrum of 3ac

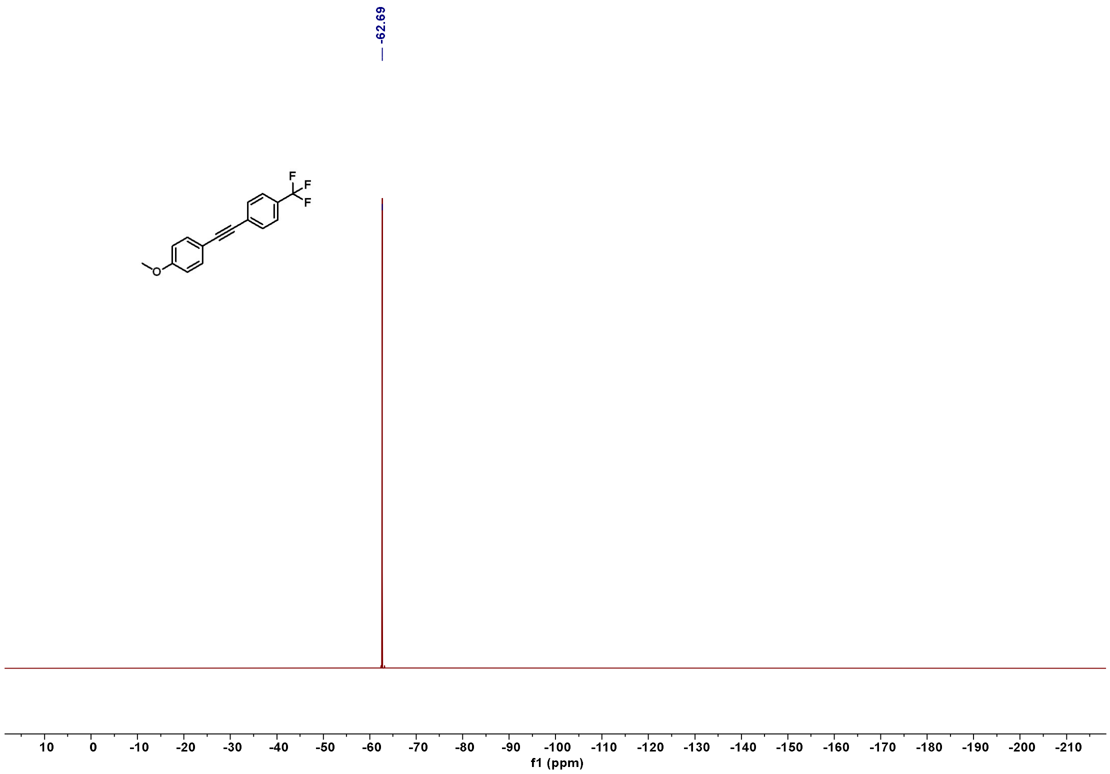

^{19}F NMR (376 MHz, $CDCl_3$) spectrum of 3ac

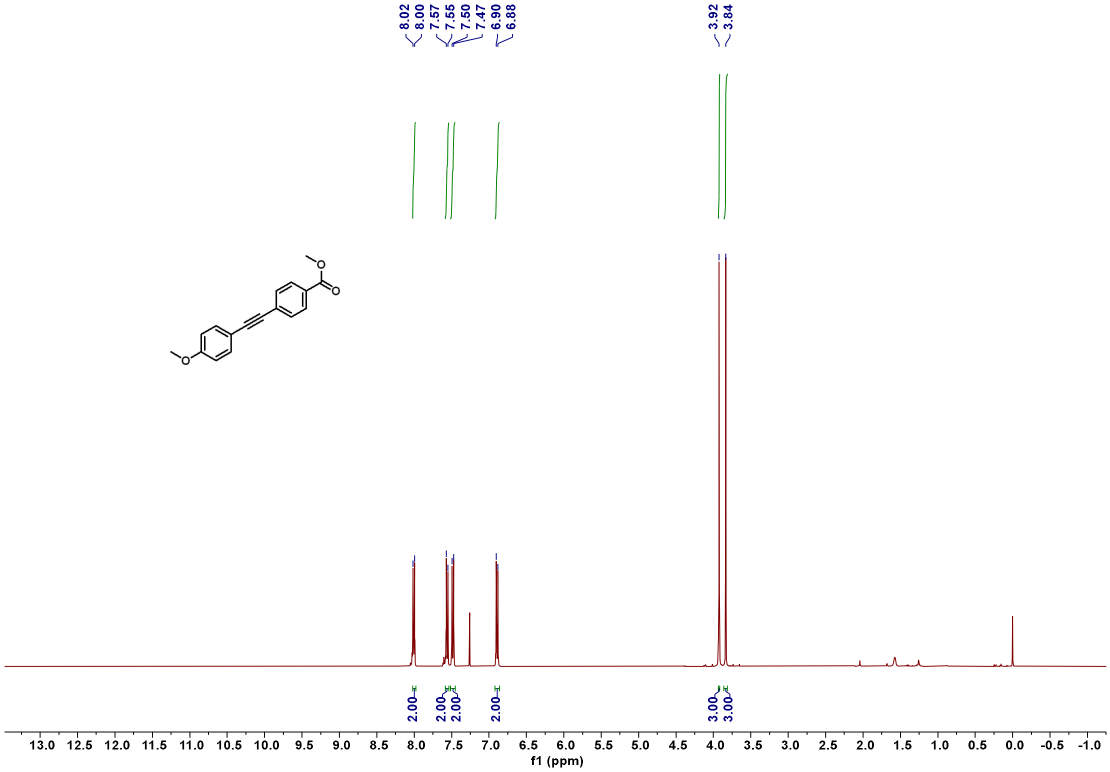

^{1}H NMR (400 MHz, CDCl3) spectrum of 3ad

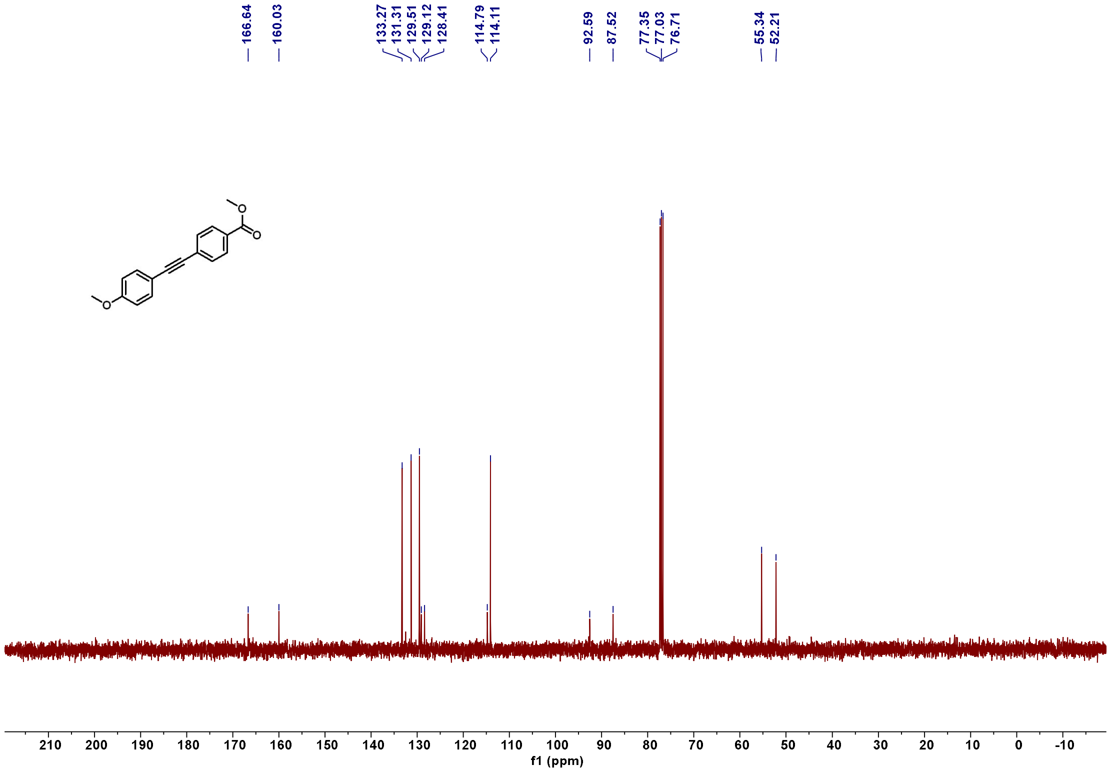

^{13}C NMR (101 MHz, CDCl3) spectrum of 3ad

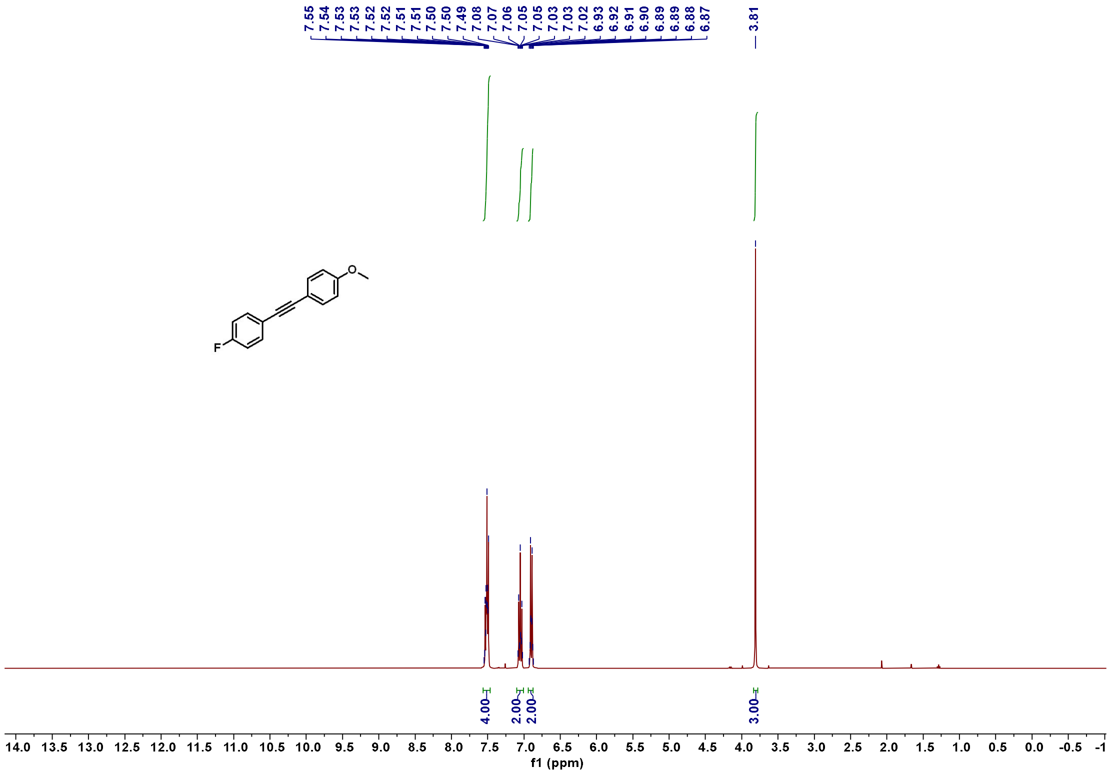

^{1}H NMR (400 MHz, $CDCl_3$) spectrum of 3ae

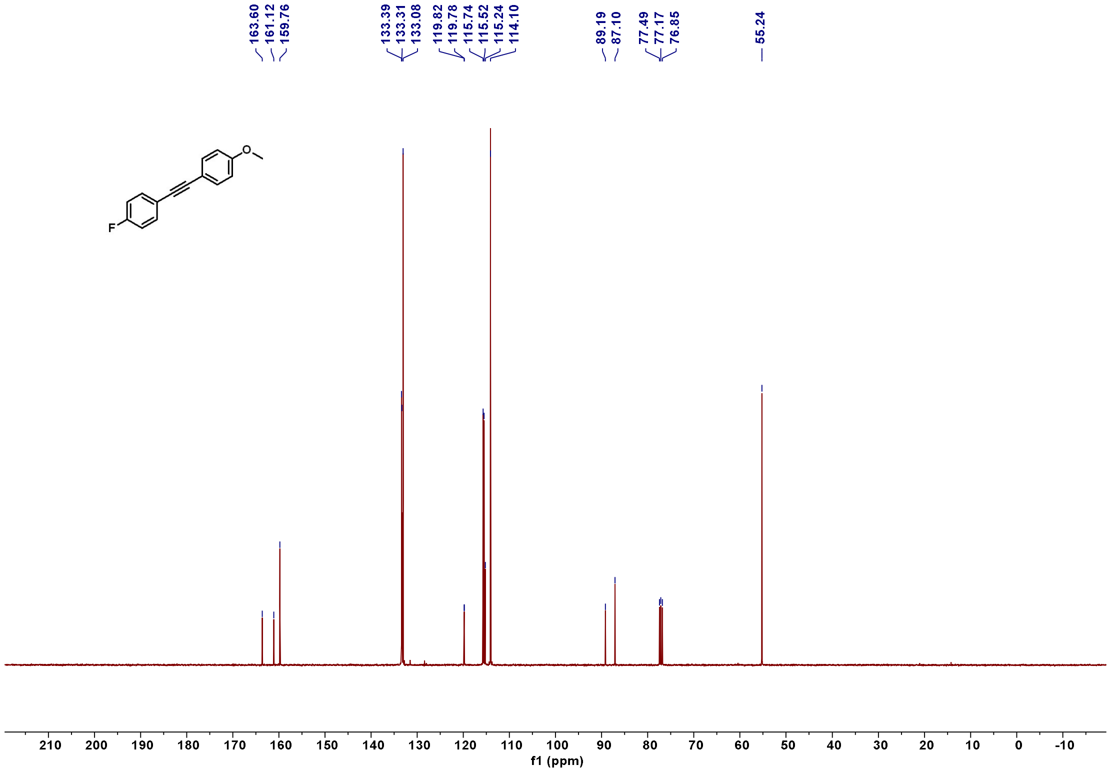

^{13}C NMR (101 MHz, $CDCl_3$) spectrum of 3ae

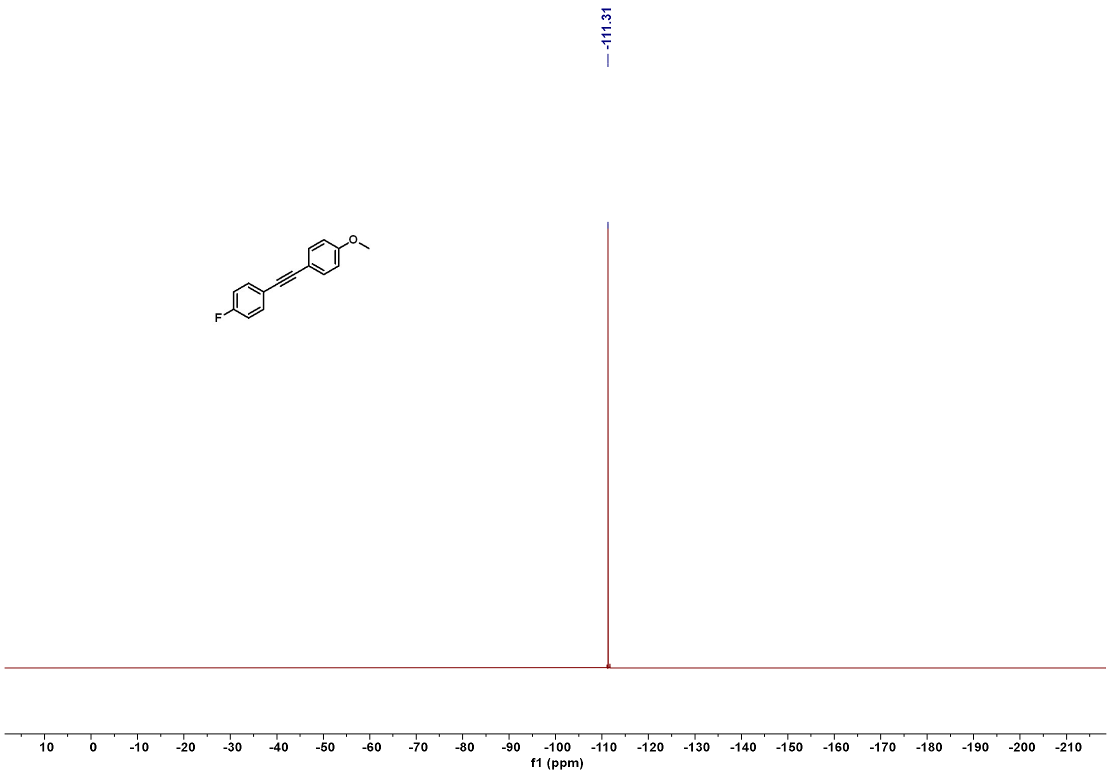

^{19}F NMR (376 MHz, $CDCl_3$) spectrum of 3ae

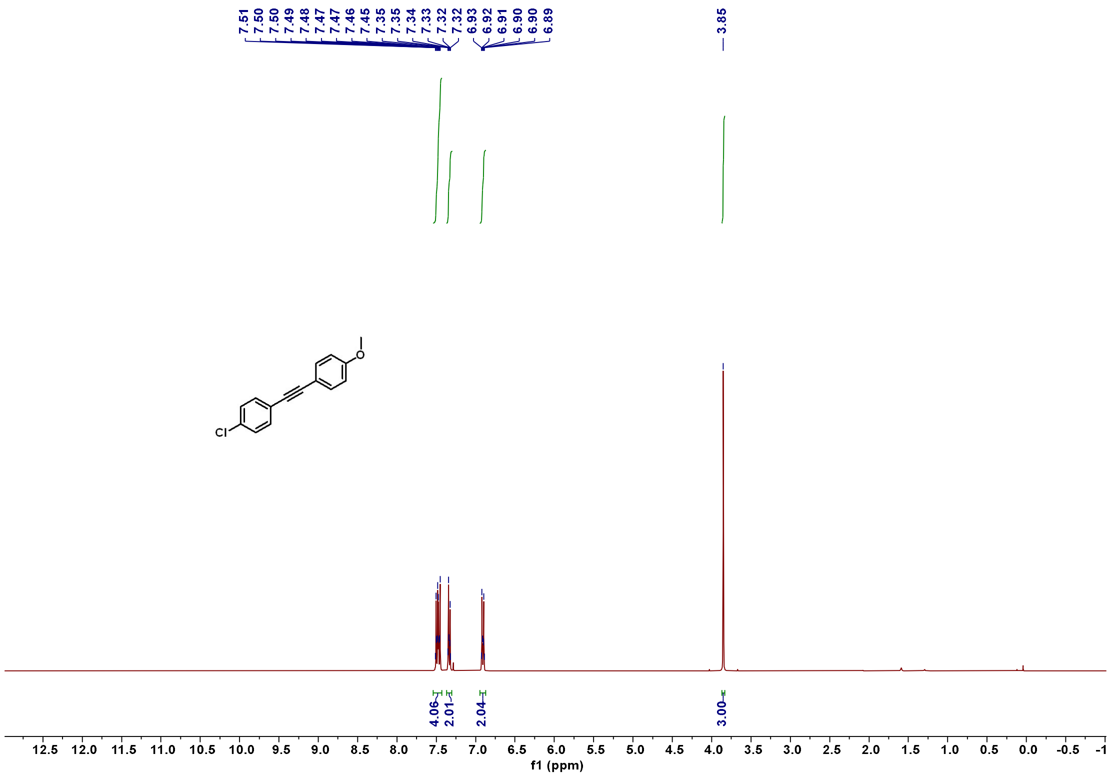

^{1}H NMR (400 MHz, CDCl$_3$) spectrum of 3af

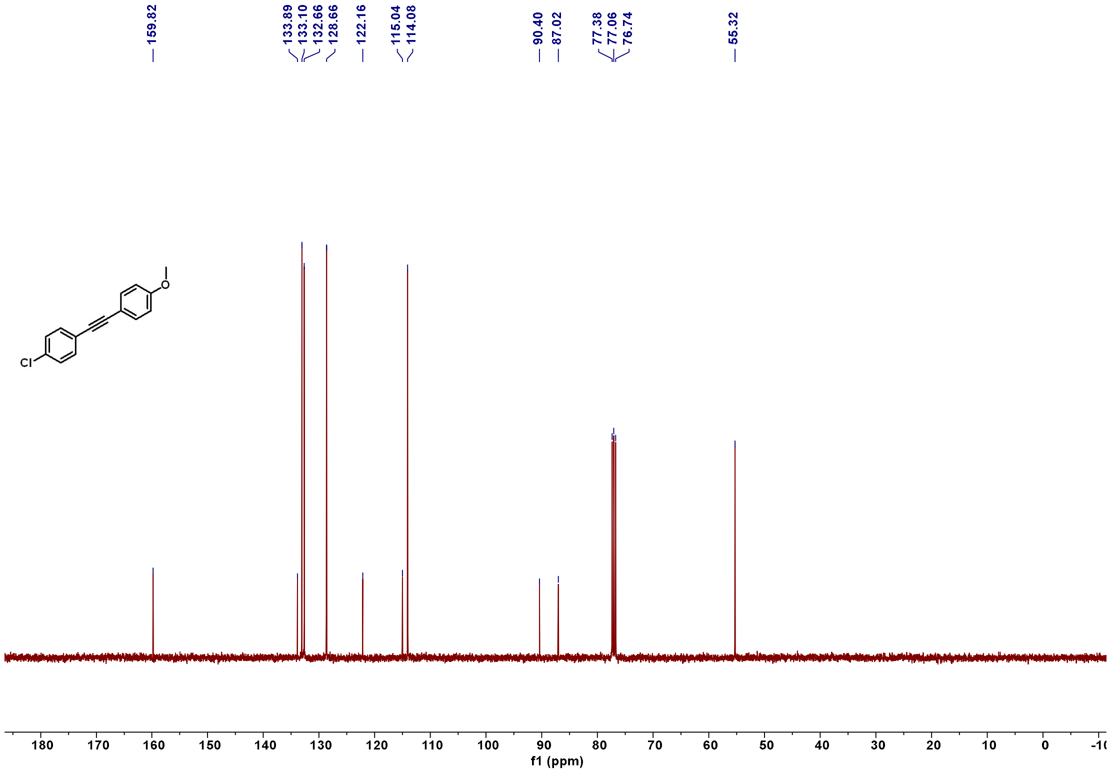

^{13}C NMR (101 MHz, CDCl$_3$) spectrum of 3af

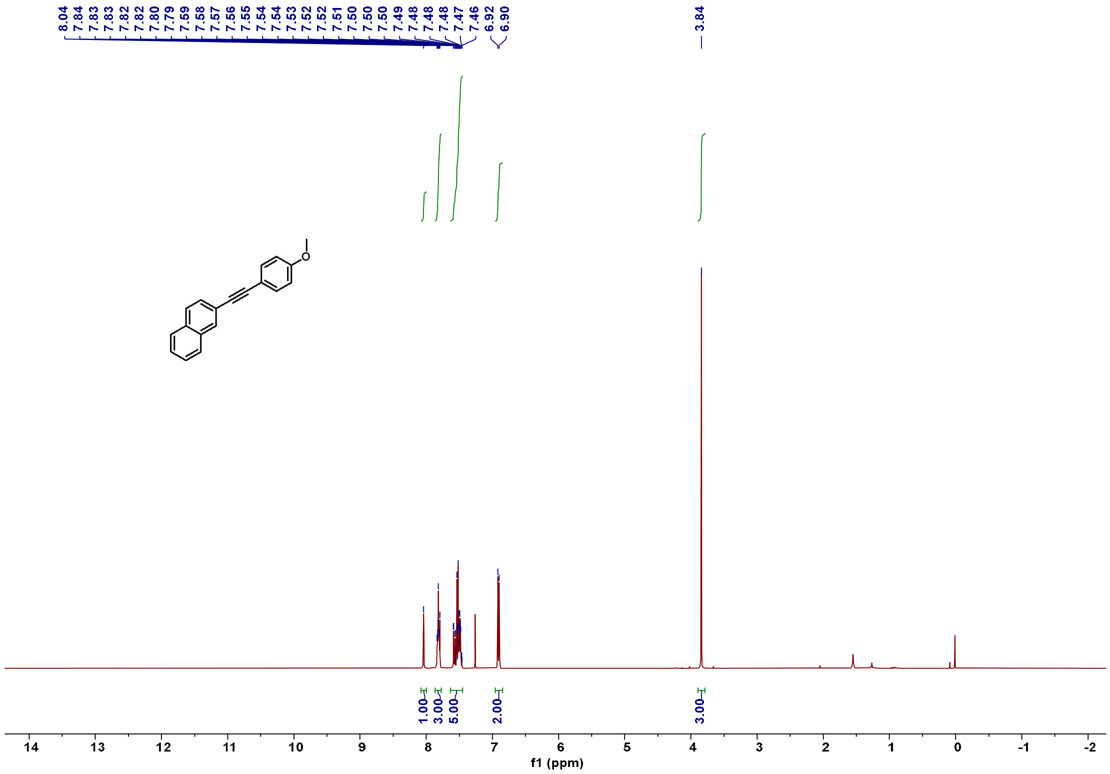

^{1}H NMR (400 MHz, $CDCl_3$) spectrum of 3ag

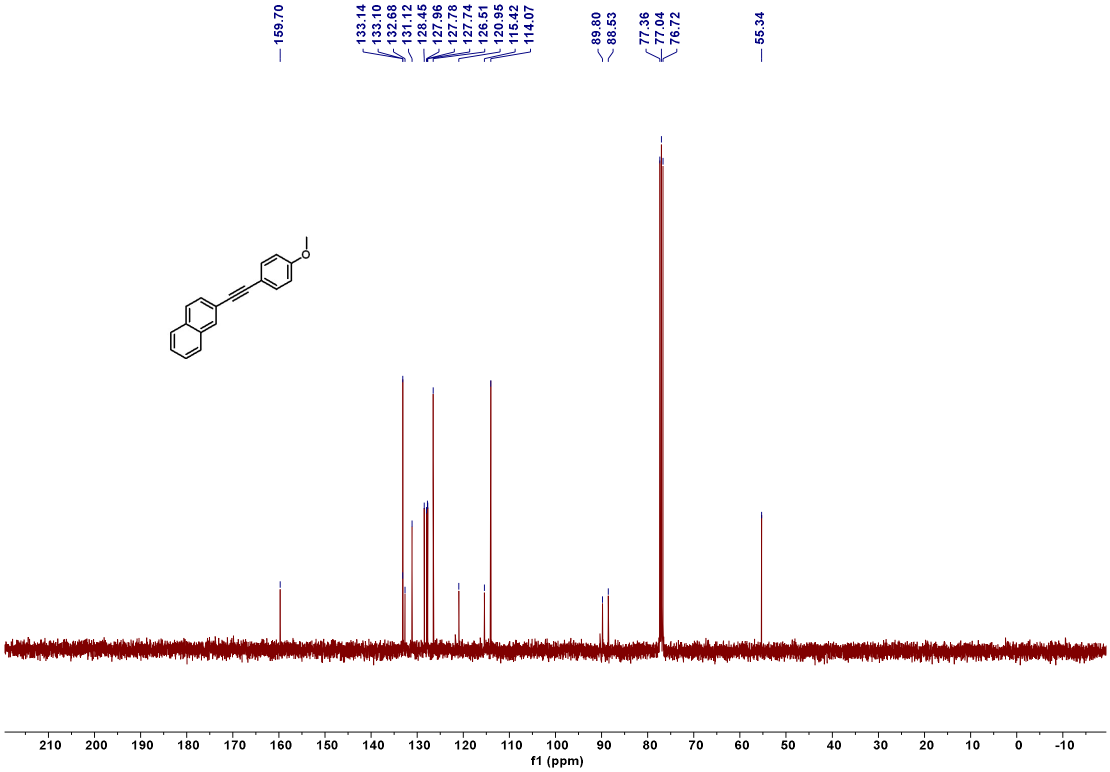

^{13}C NMR (101 MHz, $CDCl_3$) spectrum of 3ag

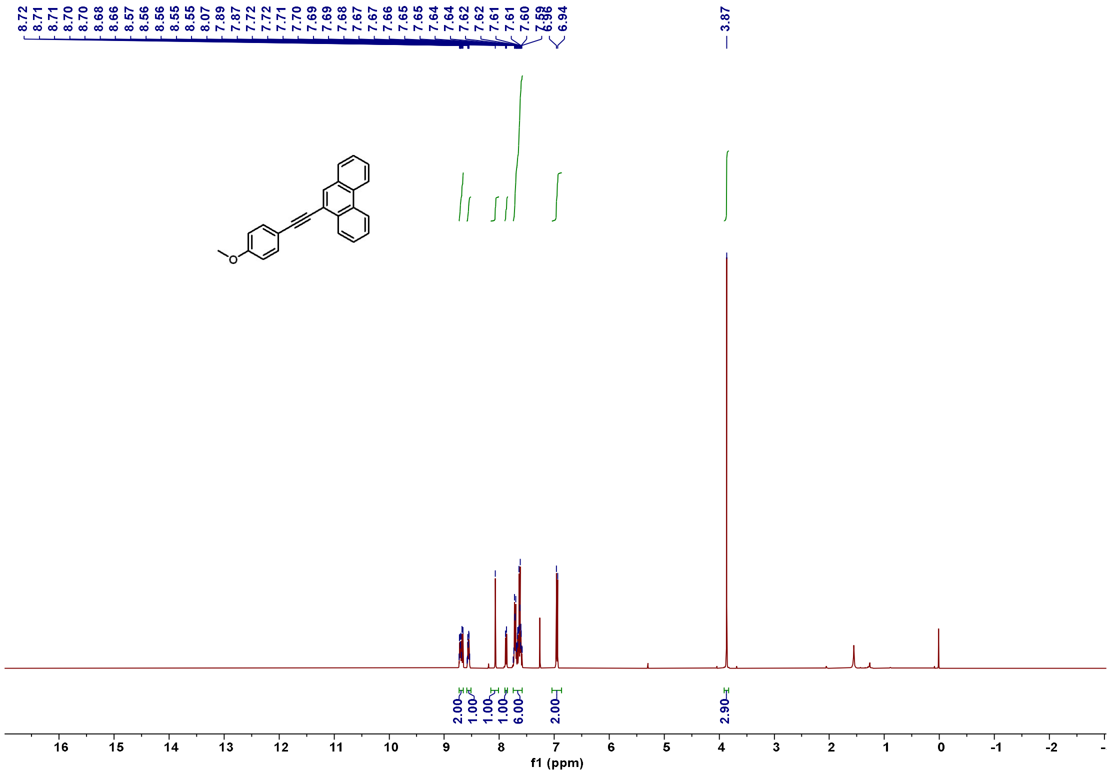

^{1}H NMR (400 MHz, CDCl₃) spectrum of 3ah

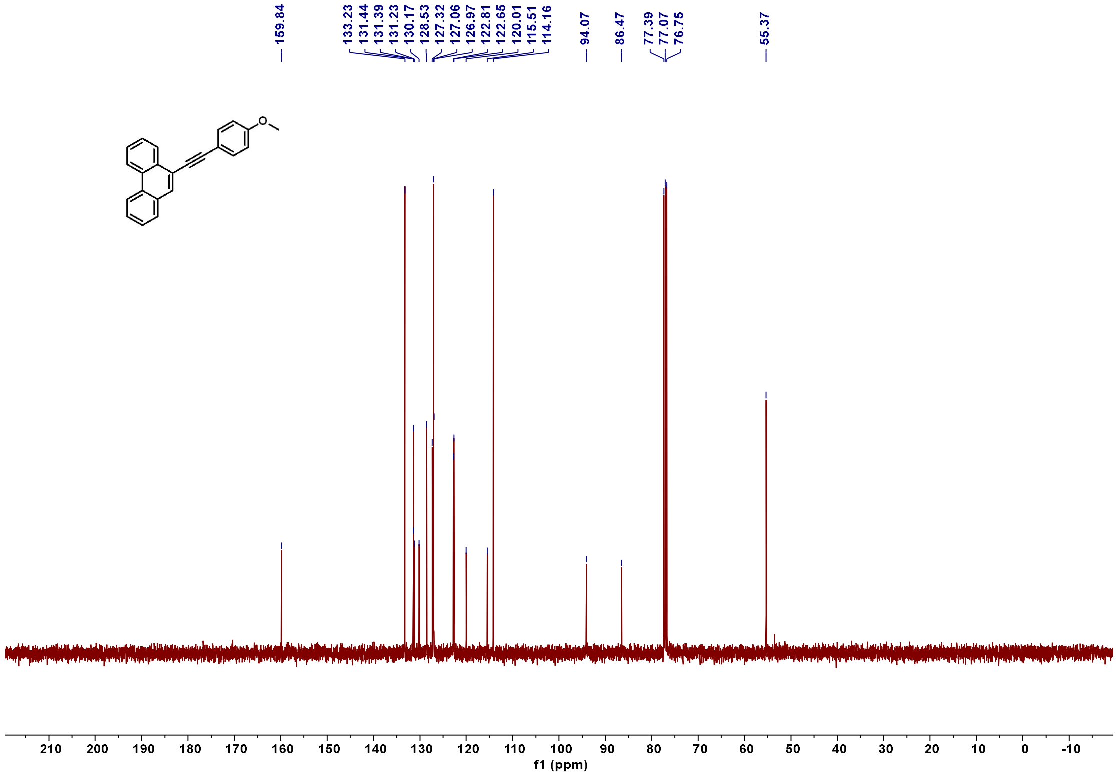

^{13}C NMR (101 MHz, CDCl₃) spectrum of 3ah

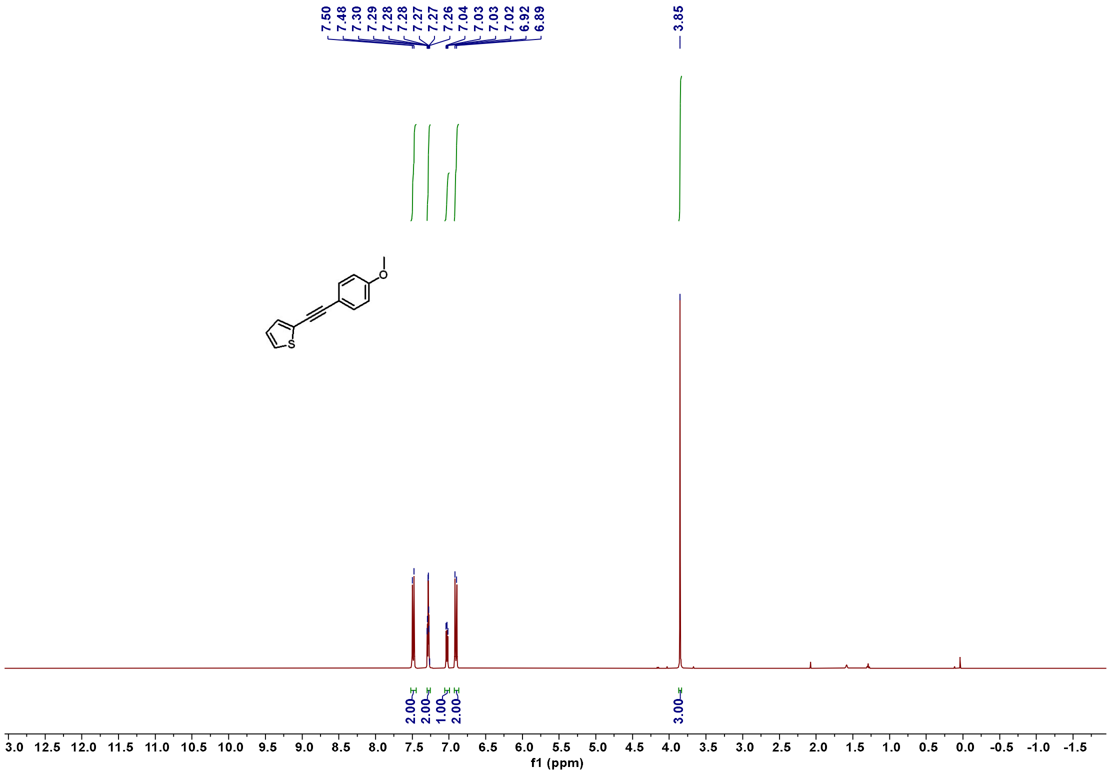

^{1}H NMR (400 MHz, CDCl3) spectrum of 3ai

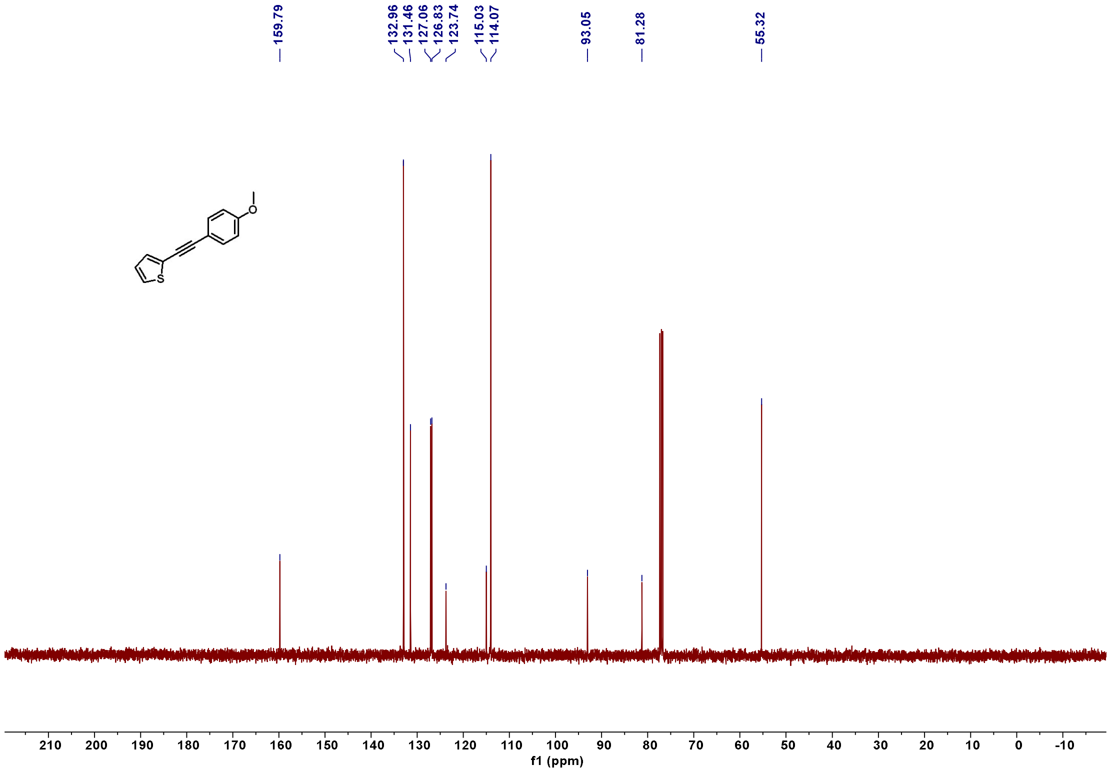

^{13}C NMR (101 MHz, CDCl3) spectrum of 3ai

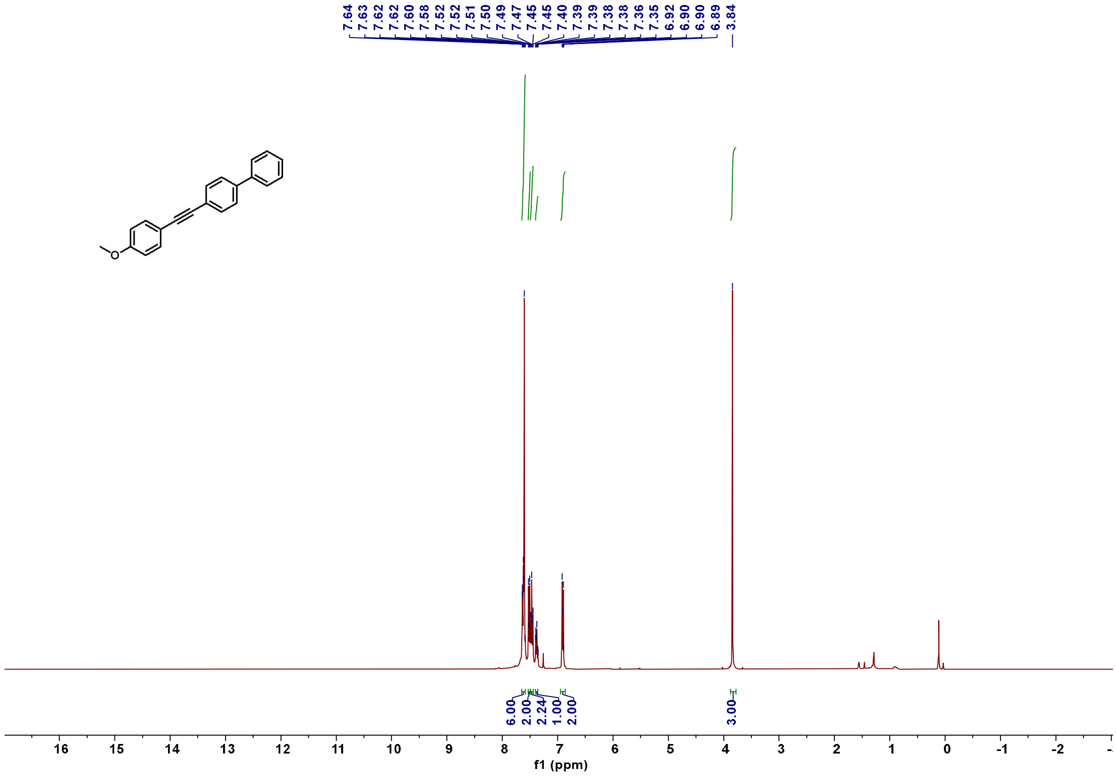

^{1}H NMR (400 MHz, CDCl₃) spectrum of 3aj

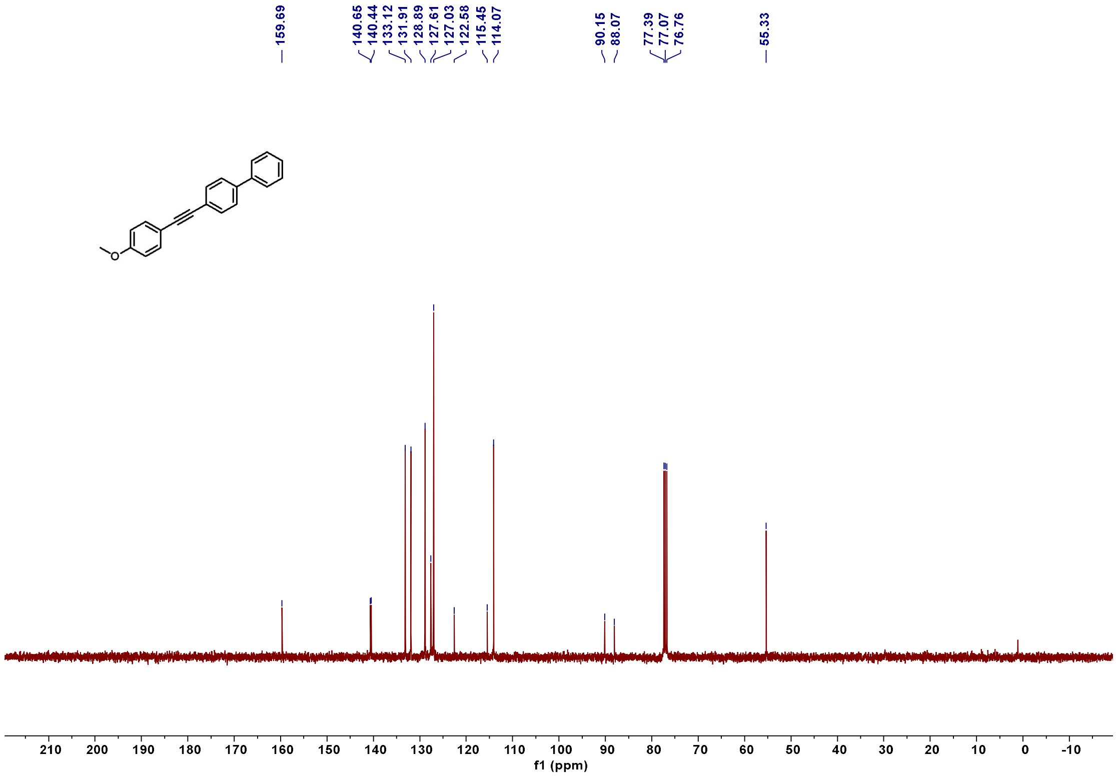

^{13}C NMR (101 MHz, CDCl₃) spectrum of 3aj

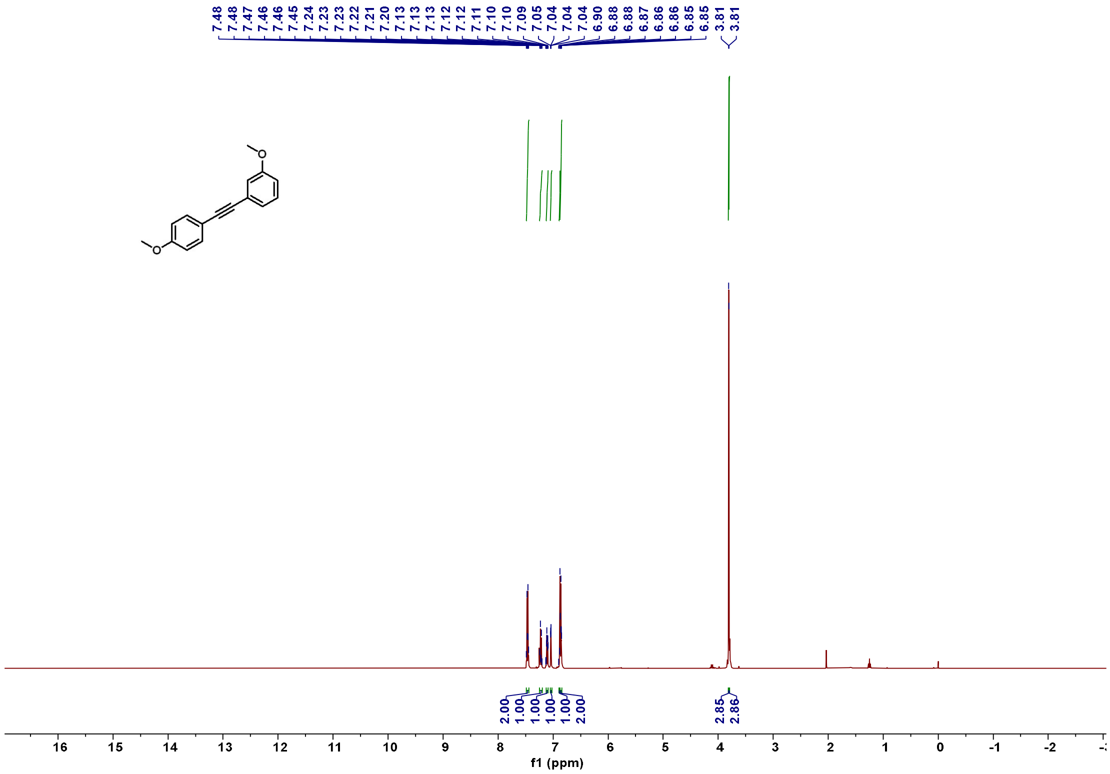

^{1}H NMR (400 MHz, $CDCl_3$) spectrum of 3ak

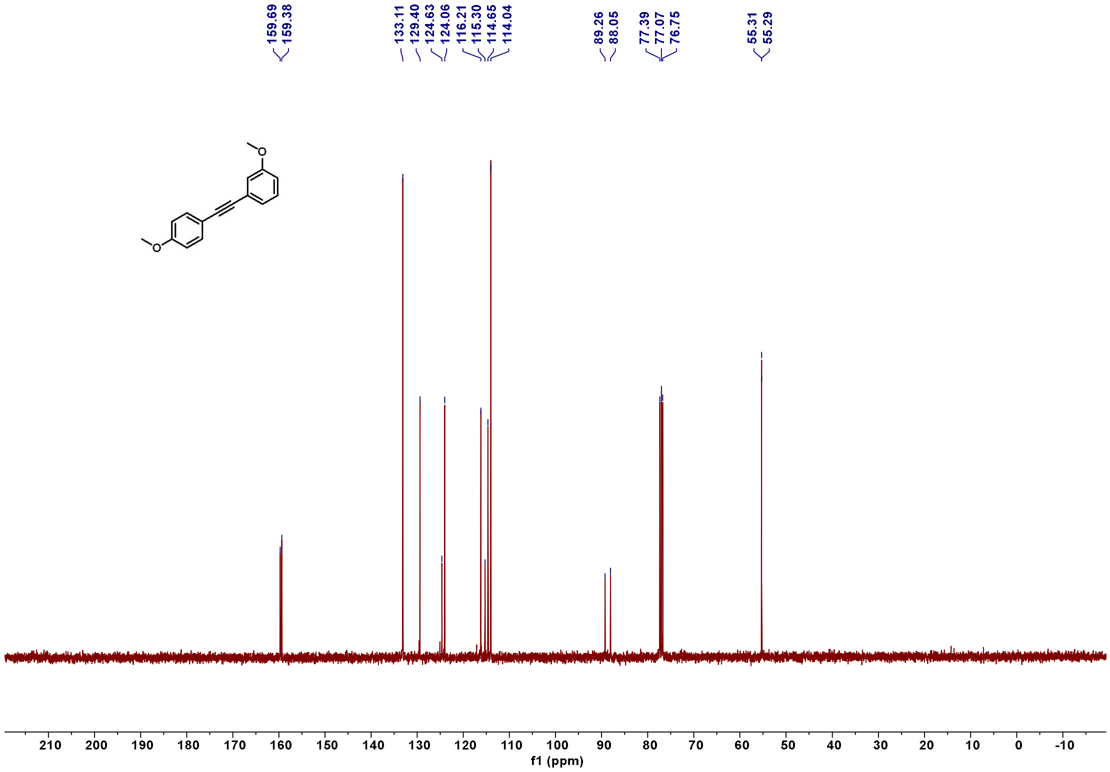

^{13}C NMR (101 MHz, $CDCl_3$) spectrum of 3ak

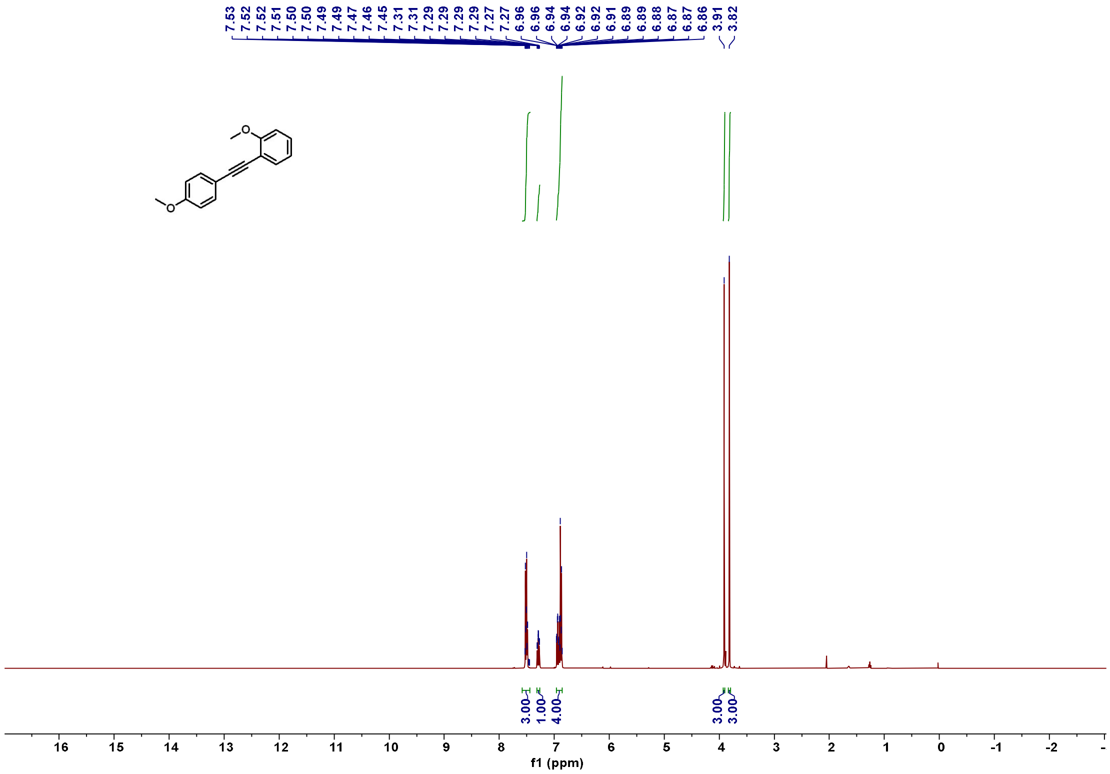

^{1}H NMR (400 MHz, $CDCl_3$) spectrum of 3al

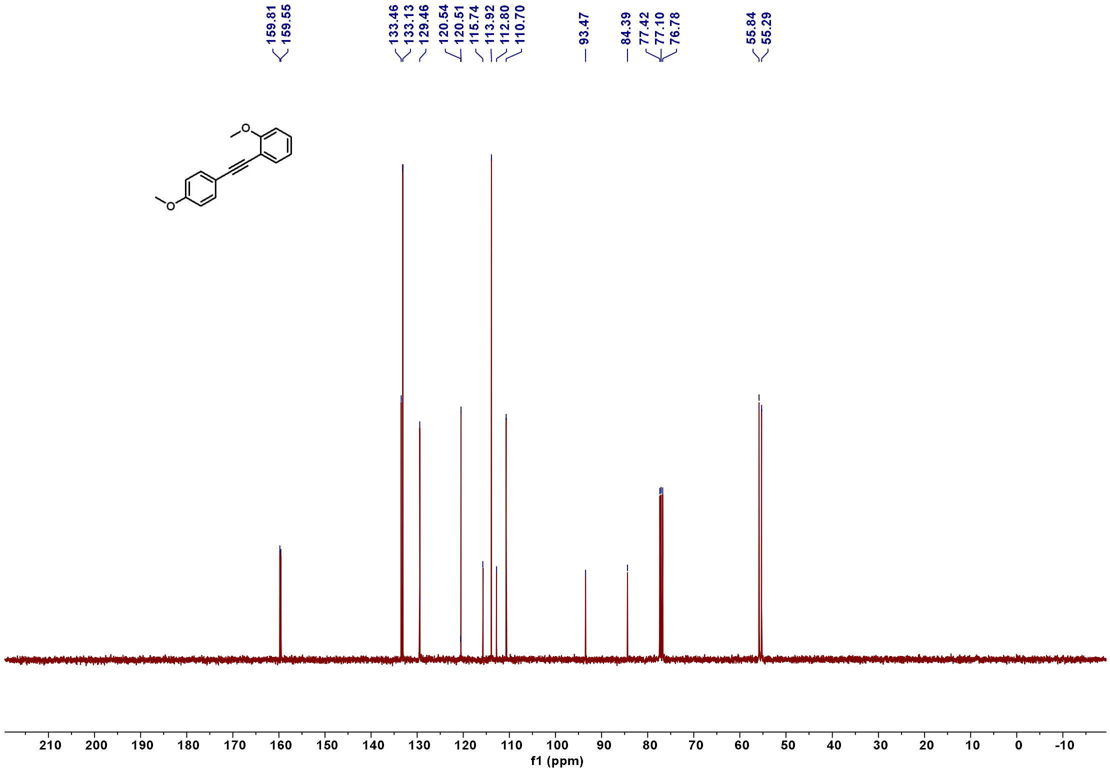

$^{13}C\{^1H\}$ NMR (101 MHz, $CDCl_3$) spectrum of 3al

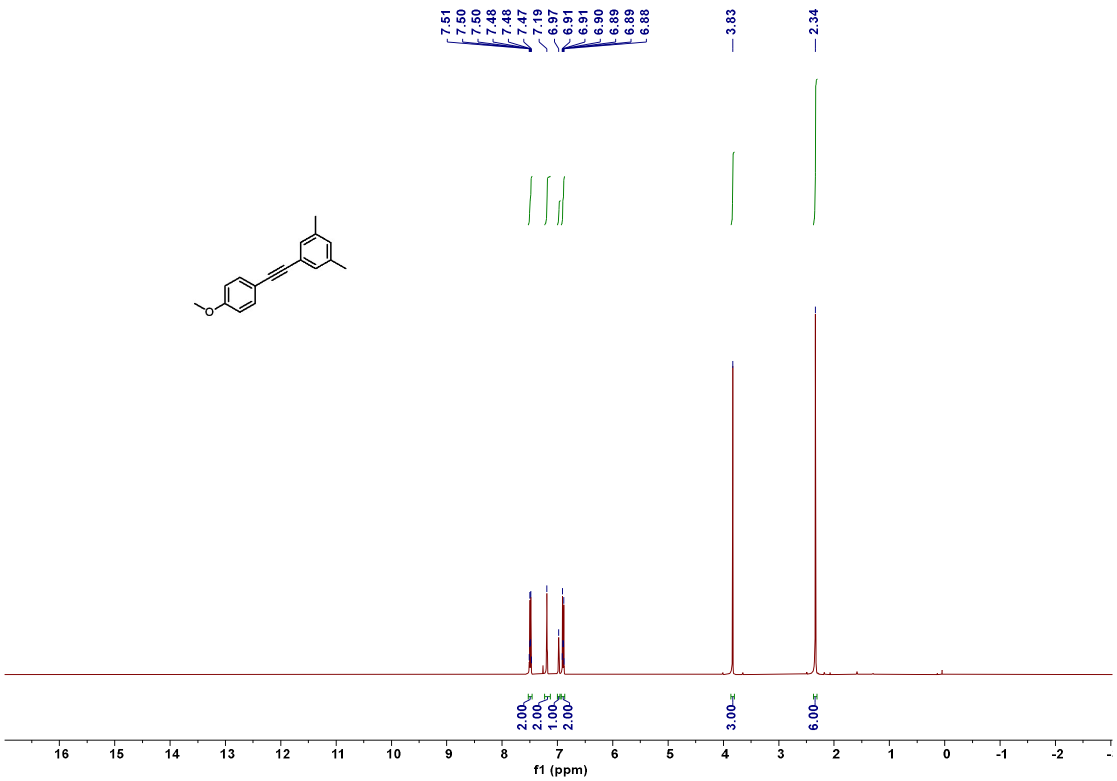

^{1}H NMR (400 MHz, CDCl₃) spectrum of 3am

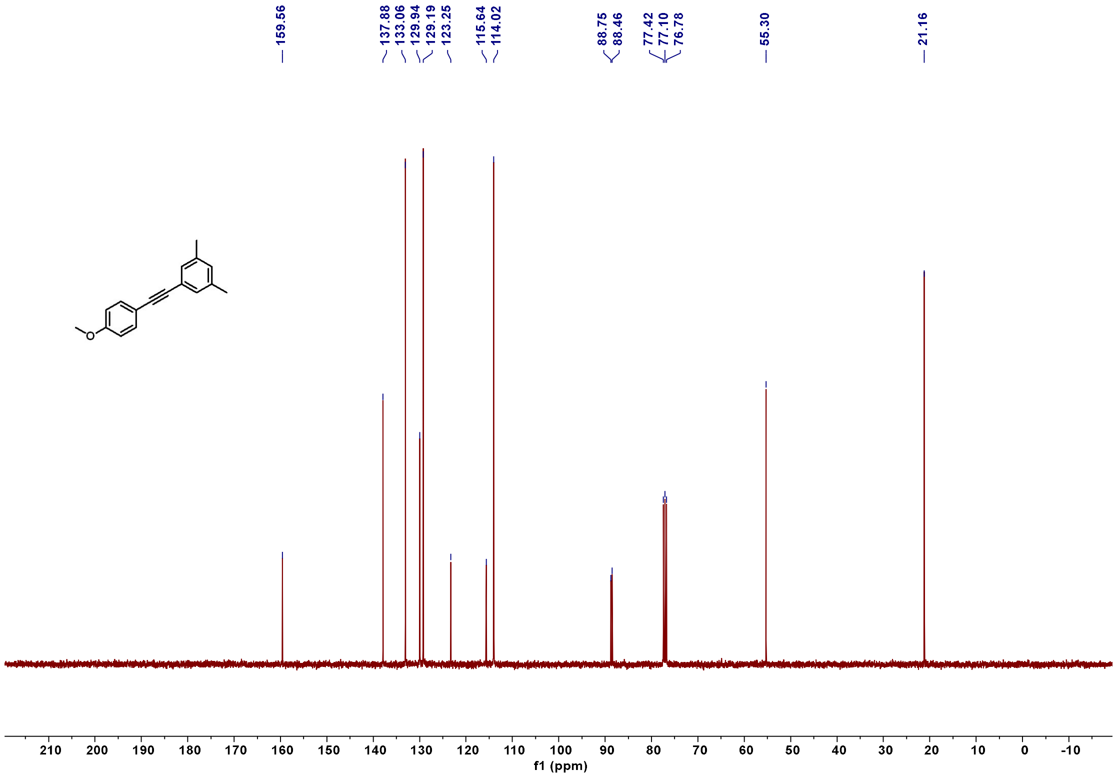

^{13}C NMR (101 MHz, CDCl₃) spectrum of 3am

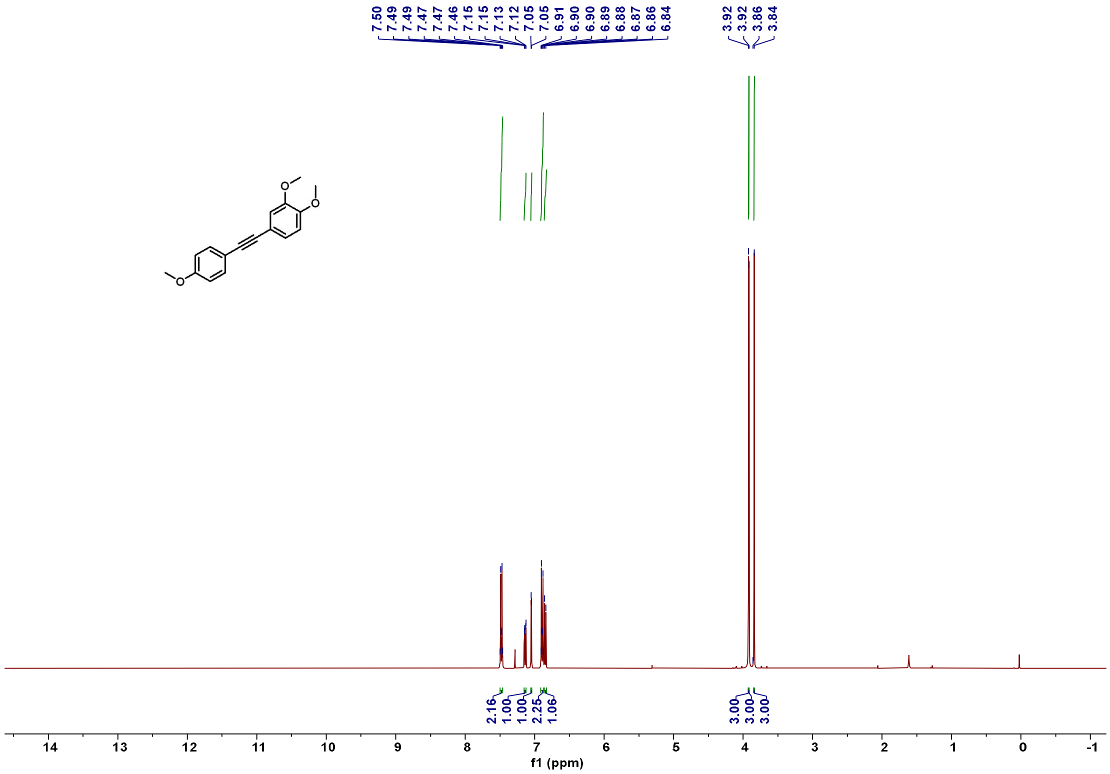

^{1}H NMR (400 MHz, CDCl3) spectrum of 3an

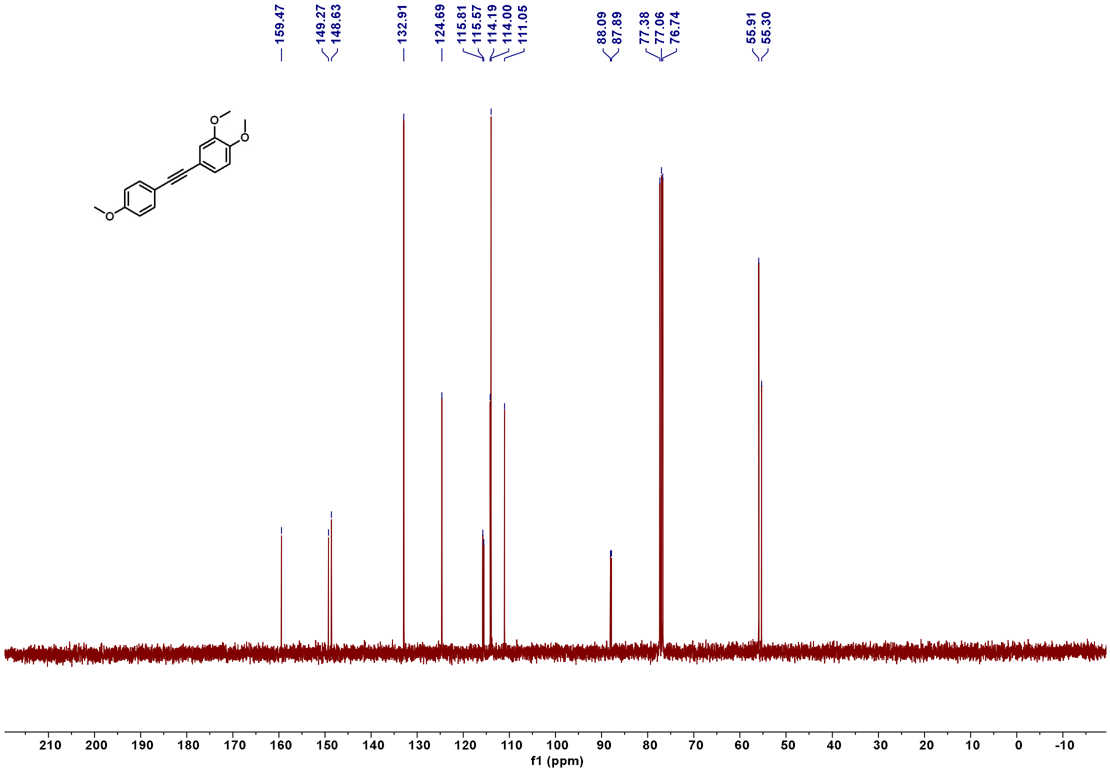

^{13}C NMR (101 MHz, CDCl3) spectrum of 3an

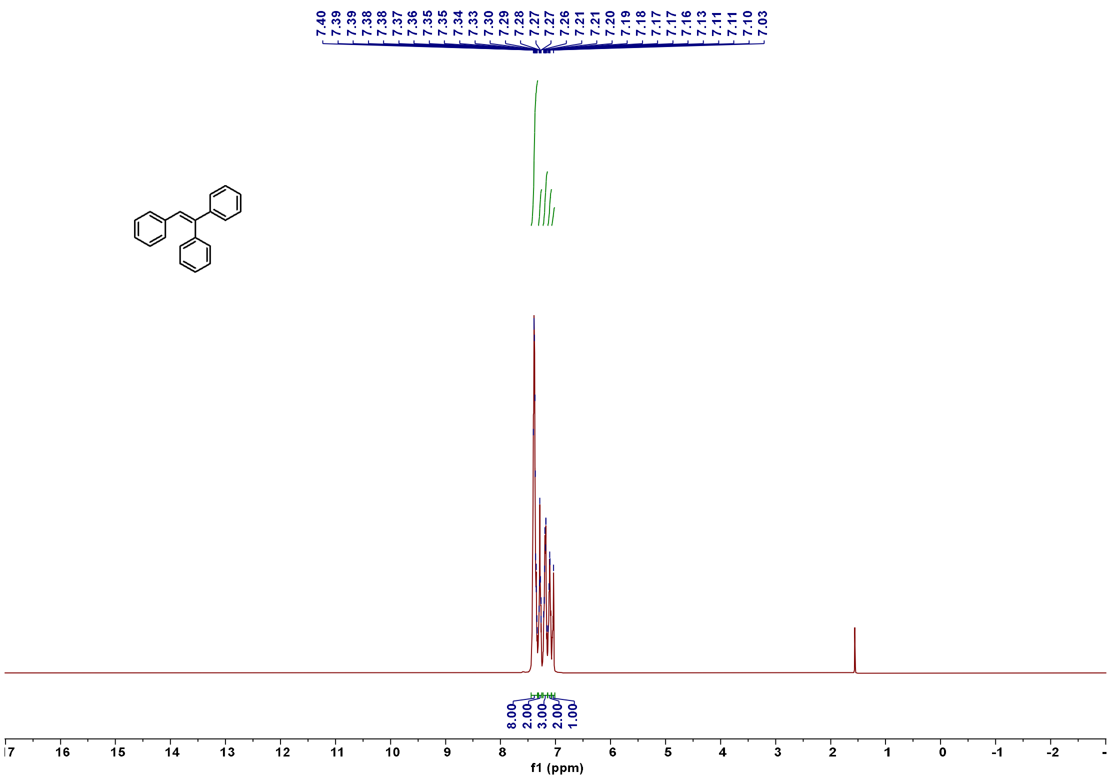

^{1}H NMR (400 MHz, CDCl3) spectrum of 4a

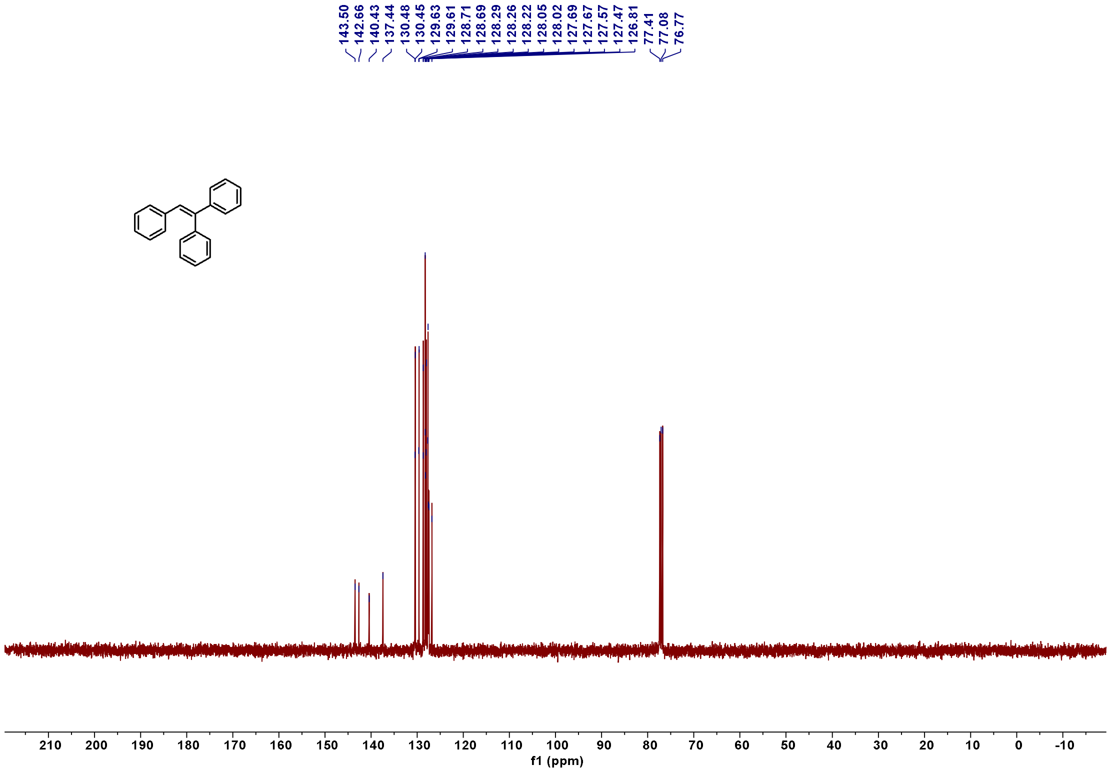

^{13}C NMR (101 MHz, CDCl3) spectrum of 4a

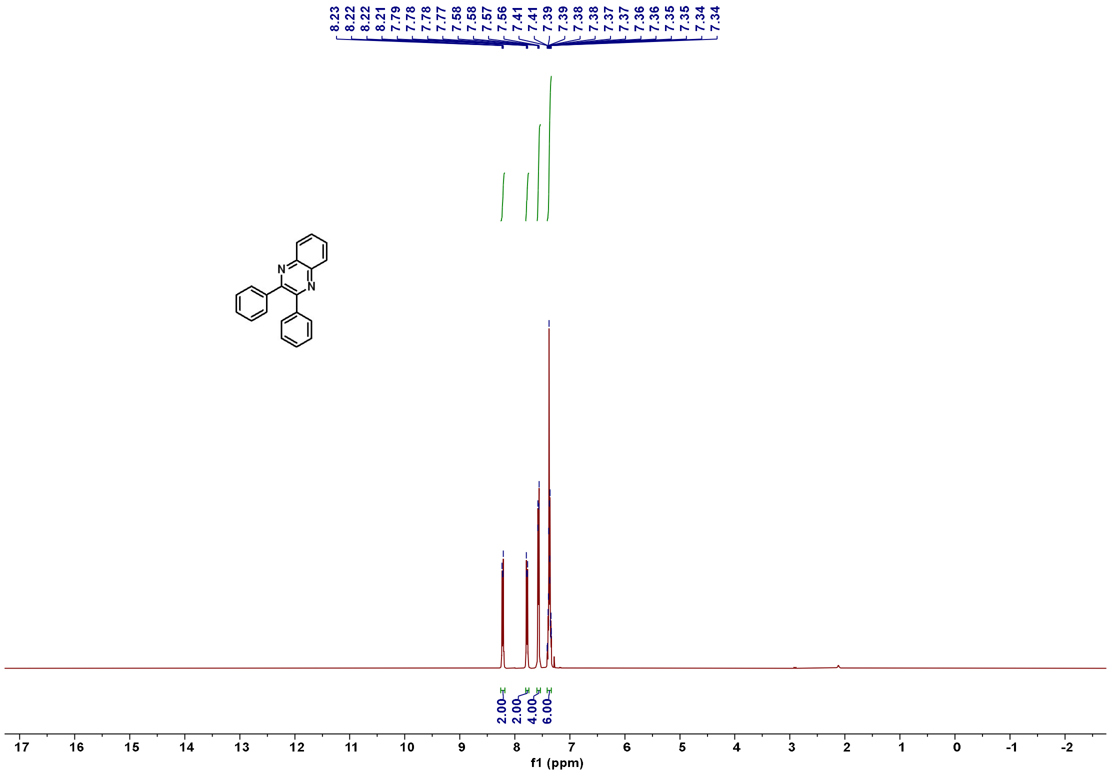

^{1}H NMR (400 MHz, $CDCl_3$) spectrum of 4b

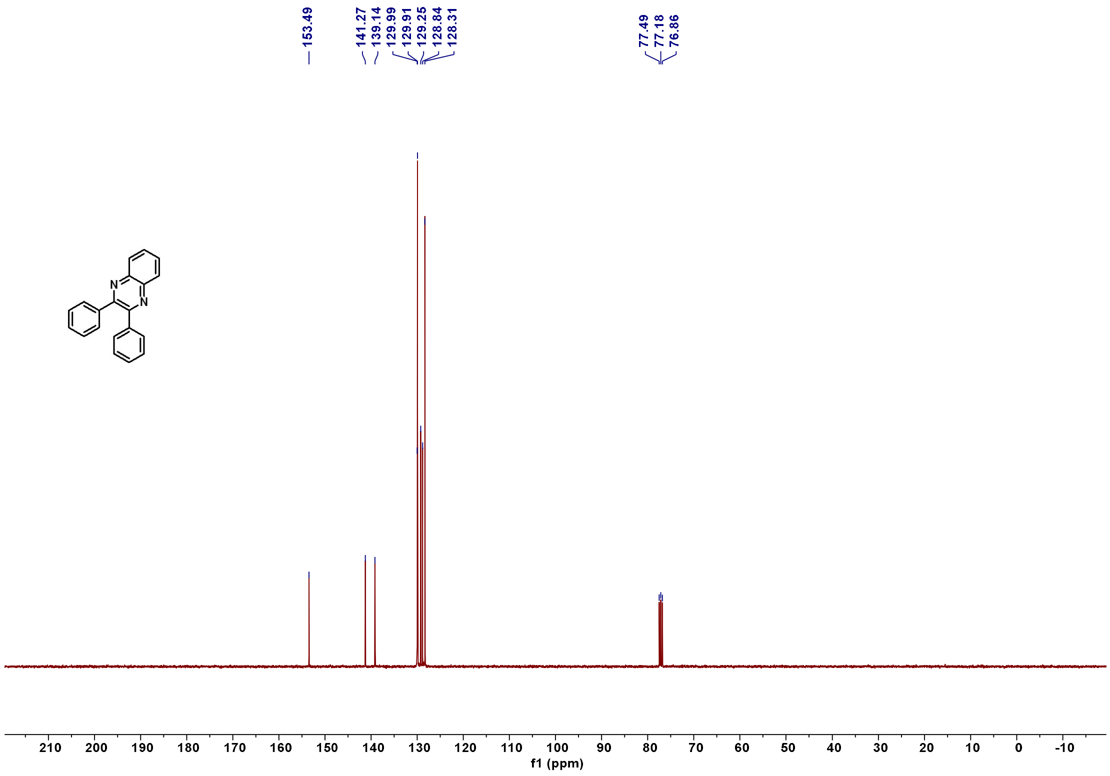

^{13}C NMR (101 MHz, $CDCl_3$) spectrum of 4b

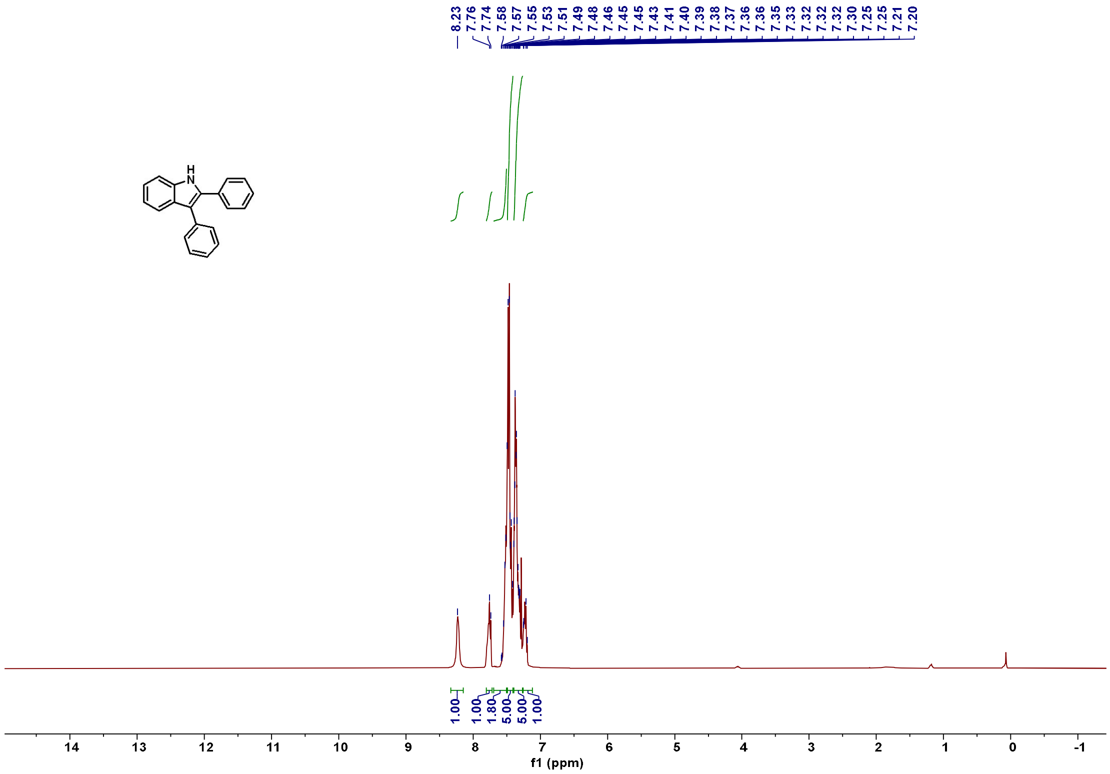

^{1}H NMR (400 MHz, CDCl3) spectrum of 4c

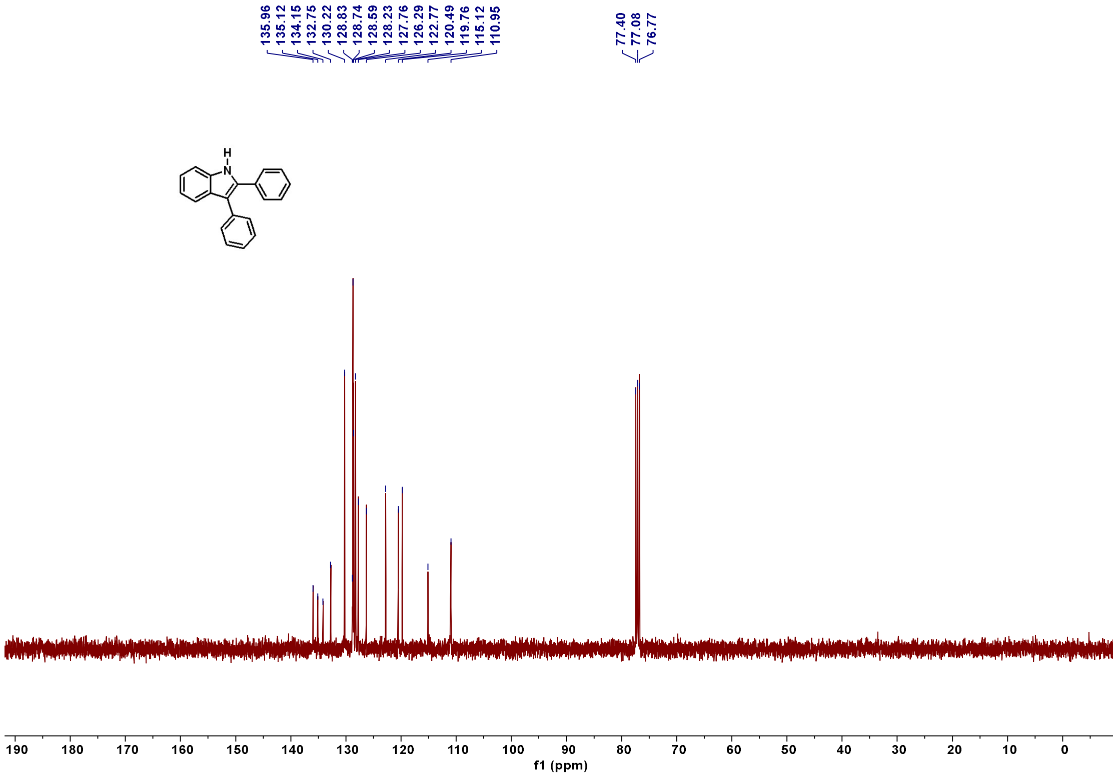

^{13}C NMR (101 MHz, CDCl3) spectrum of 4c

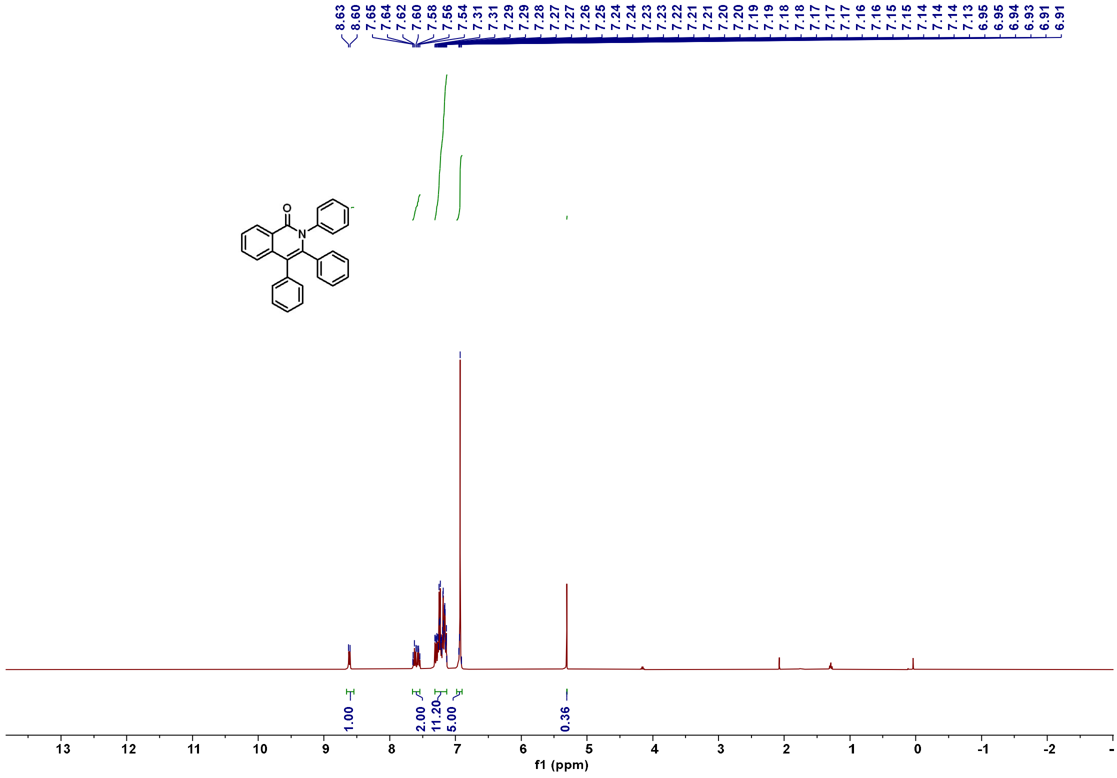

^1H NMR (400 MHz, $CDCl_3$) spectrum of 4d

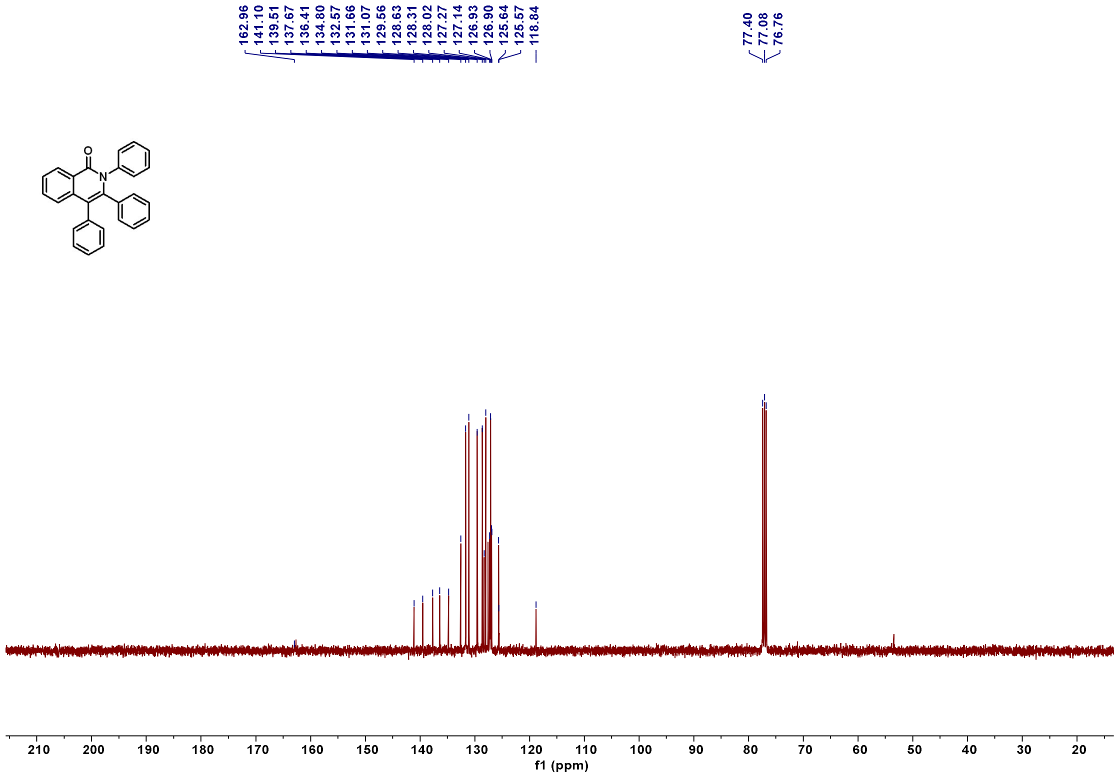

^{13}C NMR (101 MHz, $CDCl_3$) spectrum of 4d

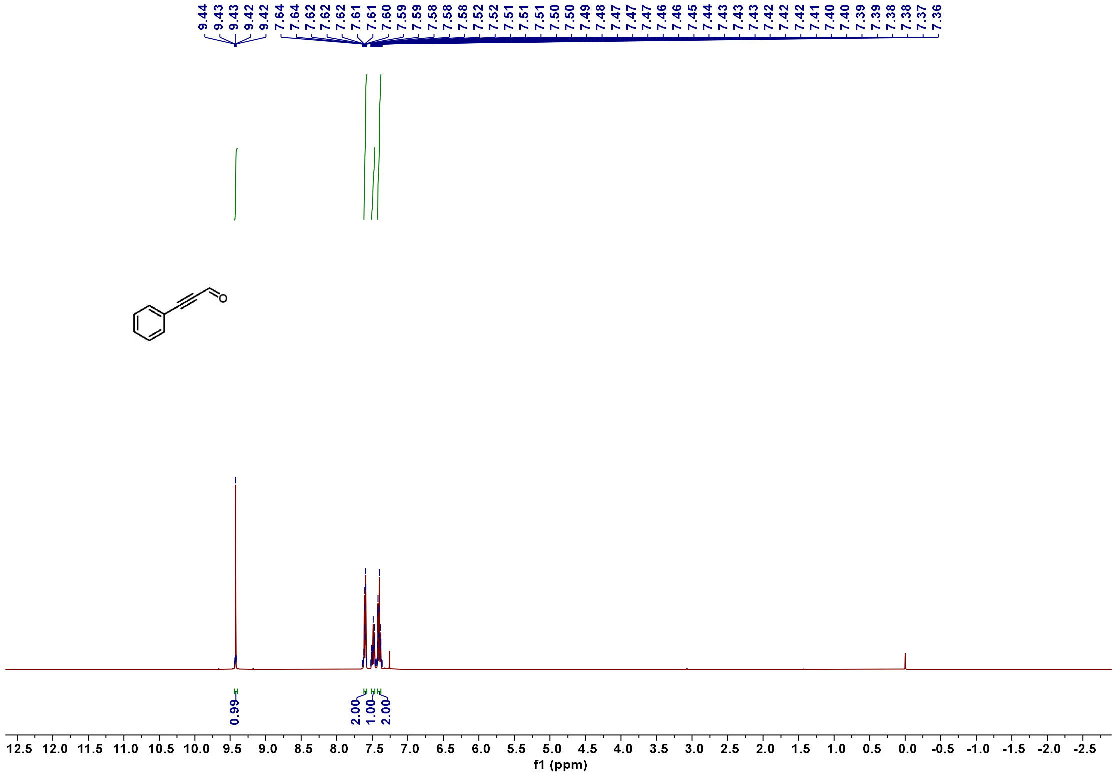

^{1}H NMR (400 MHz, CDCl₃) spectrum of IM-1

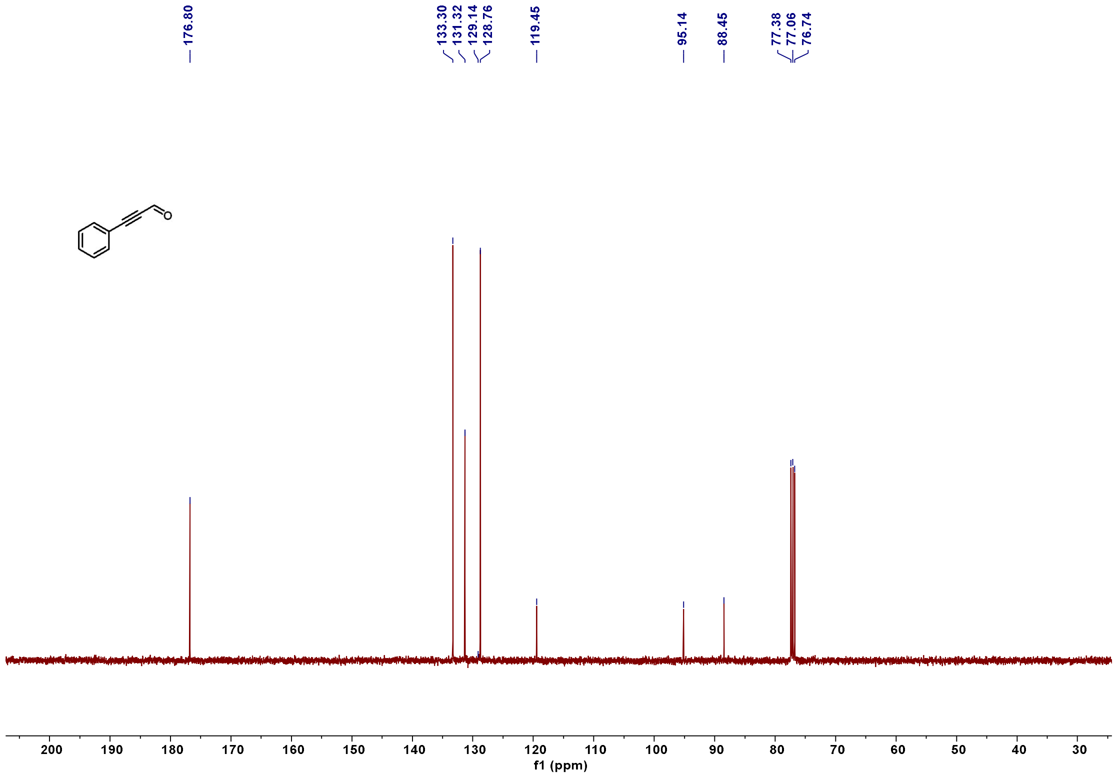

^{13}C NMR (101 MHz, CDCl₃) spectrum of IM-1
